# LAMP5 may promote MM progression by activating p38

**DOI:** 10.3389/pore.2023.1611083

**Published:** 2023-03-22

**Authors:** Yan Chen, Tao Ma

**Affiliations:** Department of Hematology, The Affiliated Hospital of Southwest Medical University, Luzhou, China

**Keywords:** apoptosis, recurrence, prognosis, multiple myeloma, LAMP5

## Abstract

Multiple myeloma (MM) is the second most common tumor of the hematologic system. MM remains incurable at this time. In this study, we used bioinformatics analysis to find key genes in the pathogenesis of MM. We first found that Lysosome associated membrane protein 5 (LAMP5) expression was sequentially increased in healthy donors (HD), monoclonal gammopathy of undetermined significance (MGUS), smoldering multiple myeloma (SMM) and newly diagnosed MM (NDMM), relapsed MM (RMM). We collected bone marrow from patients with NDMM, HD and post-treatment MM (PTMM) and performed qPCR analysis of LAMP5, and found that the expression of LAMP5 is stronger in NDMM than in HD, and decreases after treatment. Western blotting assay also found more expression of LAMP5 in NDMM than in HD. Patients with high LAMP5 expression have a higher DS (Durie-Salmon) stage and worse prognosis. We next verified the expression of LAMP5 in four MM cell lines and silenced LAMP5 expression in RPMI-8226 and AMO-1, and explored the effects of LAMP5 silencing on MM cell apoptosis and cell cycle by flow cytometry and western blotting. Knockdown of LAMP5 promoted apoptosis in MM cells, but had no effect on the cell cycle. Mechanistically, LAMP5 may exert its pro-tumor effects in MM in part through activation of p38 protein. We screened LAMP5 for the first time as a key gene for MM progression and recurrence, and found that LAMP5 may exert its pro-tumor effects in MM through activation of p38 protein.

## Introduction

Multiple myeloma (MM) is the second most common hematologic tumor characterized by an abnormal expansion of monoclonal plasma cells leading to monoclonal protein production and end organ damage (1, 2). Approximately 176,404 people worldwide are diagnosed with MM in 2020 (3). The most prominent clinical manifestations of MM are hypercalcemia, renal failure, anemia, and bone lesions (4). The emergence of treatments such as autologous hematopoietic stem cell transplantation, immunomodulatory drugs, proteasome inhibitors, daratumumab and chimeric antigen receptor T cells has greatly improved the treatment of MM, but MM is still an incurable tumor, and many MM patients will experience disease recurrence after achieving complete remission(5, 6). Abnormal gene expression is one of the main factors in the development and progression of MM (7). Years to decades before being diagnosed with MM, almost all patients can be diagnosed with monoclonal gammopathy of undetermined significance (MGUS) or smoldering multiple myeloma (SMM) (2). Genetic and epigenetic aberrations are not only present in MM but can also be present in MGUS and SMM (8). Single-cell sequencing for newly diagnosed and relapsed MM also showed that some genes (SMAD1 and STMN1, among others) are associated with MM progression and relapse (9).

Lysosome associated membrane protein (LAMP) can affect cellular processes such as phagocytosis, autophagy, lipid transport and senescence, and plays a role in cancers(10). LAMP5, also known as brain and dendritic cell-associated lysosome-associated membrane protein (BAD-LAMP), is a member of the LAMP family (11). LAMP5 expression in mice is confined to neurons, and LAMP5 interneuron deletion causes neural network dysfunction in Alzheimer’s disease (11, 12). In humans, LAMP5 is expressed in the type I IFN-producing plasmacytoid dendritic cells (pDCs), and LAMP5 in pDCs leads to nuclear transcription factor-kappa B (NF-κB) activation and tumor necrosis factor (TNF) production upon deoxyribonucleic acid (DNA) detection (11, 13). We all know that NF-κB activation can lead to the development and progression of MM (14). LAMP5 also plays an essential role in a variety of tumors. In gastric cancer, LAMP5 was upregulated in metastatic tissues, and LAMP5 knockdown significantly inhibited gastric cancer cell proliferation, invasion and migration, and increased apoptosis, cell cycle arrest and cancer stemness (15). LAMP5 also plays an important role in hematologic tumors. The expression of LAMP5 was significantly elevated in blastic plasmacytoid dendritic cell neoplasm (16). LAMP5 is highly and specifically expressed in mixed lineage leukemia-rearranged (MLL-r) leukemias and is associated with poor prognosis, and downregulation of LAMP5 leads to inhibition of NF-kB signaling and increased activation of type 1 interferon signaling downstream of Toll-like receptor/interleukin 1 receptor activation (17, 18).

In this study, we analyzed the GSE6477 dataset to identify differentially expressed genes (DEGs) between newly diagnosed MM (NDMM) and healthy donors (HD) (19, 20), NDMM and relapsed MM (RMM), and found that lysosome associated membrane protein 5 (LAMP5) may have an important role in the occurrence and relapse of MM. Our subsequent study demonstrated that LAMP5 expression was upregulated in NDMM compared to HD and correlated with poor prognosis, while post-treatment MM (PTMM) showed decreased expression of LAMP5 compared to NDMM. Knockdown of LAMP5 induced apoptosis in MM cells, which may act by reducing the expression of P38 protein, but knockdown of LAMP5 had no effect on the cell cycle of MM. Based on these findings, we suggest that LAMP5 is a key gene in the occurrence of MM and plays a tumor-promoting role in MM, with a mechanism of action exerted in part through activation of the P38 protein.

## Materials and methods

### Ethics statement

The Ethics Committees of The Affiliated Hospital of Southwest Medical University have reviewed and certified our study (2022A-87). Written informed consents were obtained from patients prior to participation in this study.

### Database

Expression profiles were downloaded from the Gene Expression Omnibus (GEO) database (www.ncbi.nim.nih.gov/geo/). The screening of DEGs of HD with NDMM and NDMM with RMM were obtained from GSE6477, and the expression of LAMP5 in HD, MGUS, SMM, NDMM, and RMM were obtained from GSE6477 (19). The effect of LAMP5 on MM survival was obtained from GSE4581.

### DEGs identification and survival analysis

We used the GEO2R (http://www.ncbi.nlm.nih.gov/geo/geo2r/) online tool to compare different samples in the GEO database to find DEGs for both datasets. DEGs between NDMM and HD, and between RMM and NDMM samples were screened by GEO2R. The Benjamini-Hochberg false discovery rate method was adopted and the adjusted *p*-value (adj. p) was used to correct the false positive results. The |log fold change (FC)|>1.2 and adj. p< 0.05 were used as cut-off criteria for screening DEGs. We uploaded the expression data of DEGs to GraphPad Prism (v8.0.1) to make heat maps. Survival data of MM patients were obtained from GSE4581, with median values of LAMP5 expression as cut-off, divided into LAMP5 high and low expression groups, and Kaplan-Meier survival analysis was performed using GraphPad Prism (v8.0.1).

### Patient samples

32 NDMM patients, 20 PTMM patients who achieved complete remission (CR) after four courses of chemotherapy were included in this study. 11 HDs were patients with iron deficiency anemia excluding tumors. NDMM, PTMM and HD did not differ in general information such as age and gender. The patients' bone marrow was handled in a manner consistent with our previous study (21). Briefly, mononuclear cells were isolated from the bone marrow of MM patients and HD by density gradient centrifugation and stored in a −80°C refrigerator (Haier, China, DW-86L728J) after adding 1 mL of Trizol reagent (TIANGEN, China, DP424). We studied 32 NDMM patients according to LAMP5 expression, divided into LAMP5^high^ and LAMP5^low^ groups based on the median expression of LAMP5, and analyzed the differences in clinical data between the two groups.

### Cell culture, antibodies and reagents

Human MM cell lines RPMI-8226, U266, H929 and AMO-1 were purchased from Procell Life Science and Technology Co., LTD. (Wuhan, China). Cell lines were cultured in RPMI-1640 medium (Tianjin Hao Yang Hua Ke Biotechnology Co., Ltd., China, HY1640) supplemented by 10% fetal bovine serum (FBS, ABW, Germany, AB-FBS-0060S). Cell lines were kept in 5% CO2 in an incubator at 37°C and passed when the cell density reached 10^6^/mL.

The antibodies used in this study. Rabbit anti-LAMP5 (ImmunoWay Biotechnology, China), Rabbit anti- cyclin-dependent kinase (CDK) 4 (Cell Signaling Technology, USA, 12790), rabbit anti-Cyclin D(CCND) 1 (Proteintech, USA, 26939-1-AP), rabbit anti- Cyclin B (CCNB) 1 (Cell Signaling Technology, USA, 12231), rabbit anti- Phospho- cyclin-dependent kinase 1(P-CDK 1) (Cell Signaling Technology, USA, 4539), mouse anti- B cell lymphoma 2 (BCL2) (Cell Signaling Technology, USA, 15071), rabbit anti-BCL2-Associated X (BAX) (abcam, England, ab32503), rabbit anti-Cleaved Caspase-3 (Cell Signaling Technology, USA, 9664), rabbit anti-extracellular signal-regulated kinase (ERK) 1 (phospho T202) + ERK2 (phospho T185) (abcam, England, ab201015), rabbit anti-phospho- mitogen and stress-activated protein kinase (MSK) 1 (Thr581) (Cell Signaling Technology, USA, 9595), rabbit anti-p38(phospho T180) (abcam, England, ab178867), rabbit anti-NF-κB p65 (phospho S276) (abcam, England, ab183559), rabbit anti-glyceraldehyde-3-phosphate dehydrogenase (GAPDH) (Hangzhou HuaAn Biotechnology Co.,Ltd., China, ET1601-4), Goat Anti-Rabbit IgG (Easybio, China, BE0101-100), Goat Anti-Rabbit IgG (Easybio, China, BE0101-100), mouse anti-β-actin (Beijing zhongshan Jinqiao Biotechnology Co., Ltd., China, 211050604), mouse anti-β-Tubulin(Cell Signaling Technology, USA, 86298).

### RNA isolation and quantitative RT-PCR (qPCR) assay

The methods, reagents and instruments for RNA extraction and qPCR were the same as in our previous study (21). Briefly, total RNA is isolated using Trizol, reverse transcribed into cDNA, primers are added and detected using the ThermoFisher 7500 real time PCR system. With glyceraldehyde-3-phosphate dehydrogenase (GAPDH) serving as the internal control gene, qPCR was performed with triplicates and relative expression of each target gene was calculated with means of 2^−ΔΔCT^ method. The primer sequences for LAMP5 are as follows. Forward (5′–3′): CCC​CTG​ATT​TTG​GGG​CTC​AT, Reverse (5′–3′): TAC​TGG​GAT​CTG​TCC​CGA​GG. The primer sequences for GAPDH are as follows. Forward (5′–3′): GGA​AGC​TTG​TCA​TCA​ATG​GAA​ATC, Reverse (5′–3′): TGA​TGA​CCC​TTT​TGG​CTC​CC.

### Cell transfection

SiRNA targeting LAMP5 (SiLAMP5) and siRNA negative control (SiNC) were acquired from Genepharma (Shanghai, China). Briefly, 3 × 10^5^ cells were plated in 6-well plates, and Lipofectamine 2000 (Invitrogen) was used for siRNA transfection(21). After 24 h of transfection, the medium was replaced, flow cytometry was performed after 48 h of transfection, and western blot was performed after 72 h of transfection. The sequences of SiRNA are in Table 1.

**TABLE 1:** Sequences of SiRNA used in this study.

Name	Sense(5′–3′)	Antisense (5′–3′)
Si1	CUU​UCC​ACU​AAC​CCU​GAA​ATT	UUU​CAG​GGU​UAG​UGG​AAA​GTT
Si2	CAA​ACC​AUU​UCA​CUG​GCC​UTT	AGG​CCA​GUG​AAA​UGG​UUU​GTT
Si3	GUA​AAG​GAA​AGC​CAC​AAC​ATT	UGU​UGU​GGC​UUU​CCU​UUA​CTT
Si4	CUA​CGU​AGA​UCU​GAU​CAC​ATT	UGU​GAU​CAG​AUC​UAC​GUA​GTT
SiNC	UUC​UCC​GAA​CGU​GUC​ACG​UTT	ACG​UGA​CAC​GUU​CGG​AGA​ATT

### Cell apoptosis analysis

Cells were collected 48 h after transfection and washed once with phosphate buffer saline (PBS). Cell suspension was mixed with 5 μL Annexin V-APC and 10 μL 7-AAD (Multi sciences, China, AP105) for 15 min in a dark room (21). Then apoptotic cells were detected by flow cytometry (Cytoflex, Beckman Coulter, USA).

### Cell cycle analysis

Cells were collected 48 h after transfection and washed once with cold PBS, then resuspended and fixed with 70% cold ethanol in 4°C overnight. The cells were washed again with cold PBS, stained with a mixture of 1 mL PI and RNase A (Multi sciences, China, CCS012), and incubated in darkness at room temperature for 30 min (21). Then cell cycle was analyzed by flow cytometry (Cytoflex, Beckman Coulter, USA).

### Western blot assay

Total proteins were extracted 72 h after transfection and used the BCA method to detect protein concentrations. 30 ug protein lysates were separated into 10% SDS-PAGE gels and transferred onto 0.45 μm polyvinylidene fluoride (PVDF) membranes (Millipore, Italy, IPVH00010). Membranes were blocked with QuickBlock^TM^ Western sealed liquid (Beyotime, China, P0252-100 mL), followed by incubation with specific primary antibodies and corresponding second antibodies (21). At last, the enhanced chemiluminescence reagent (Biosharp, China, BL520A) was used to detect the protein expression, and images were obtained by the automatic chemiluminescence image processing system (Tanon 5200 Multi, Shanghai, China)(21). We used ImageJ software to determine the grayscale values of protein expression. Ratio of the gray value of target bands to that of the internal reference β-tubulin or β-actin band represents the relative protein expression.

### Statistical analysis

GraphPad Prism 8.0 software was used for statistical analysis. The figures were drawn in GraphPad Prism 8.0. Values are expressed as mean and standard deviation. Categorical variables were compared using fisher’s exact test. Numerical variables were compared using Student’s t-test. Survival analysis was performed using the Kaplan-Meier method. *p* < 0.05 was considered statistically significant.

## Results

### DEGs identification and heat maps

**FIGURE 1:**
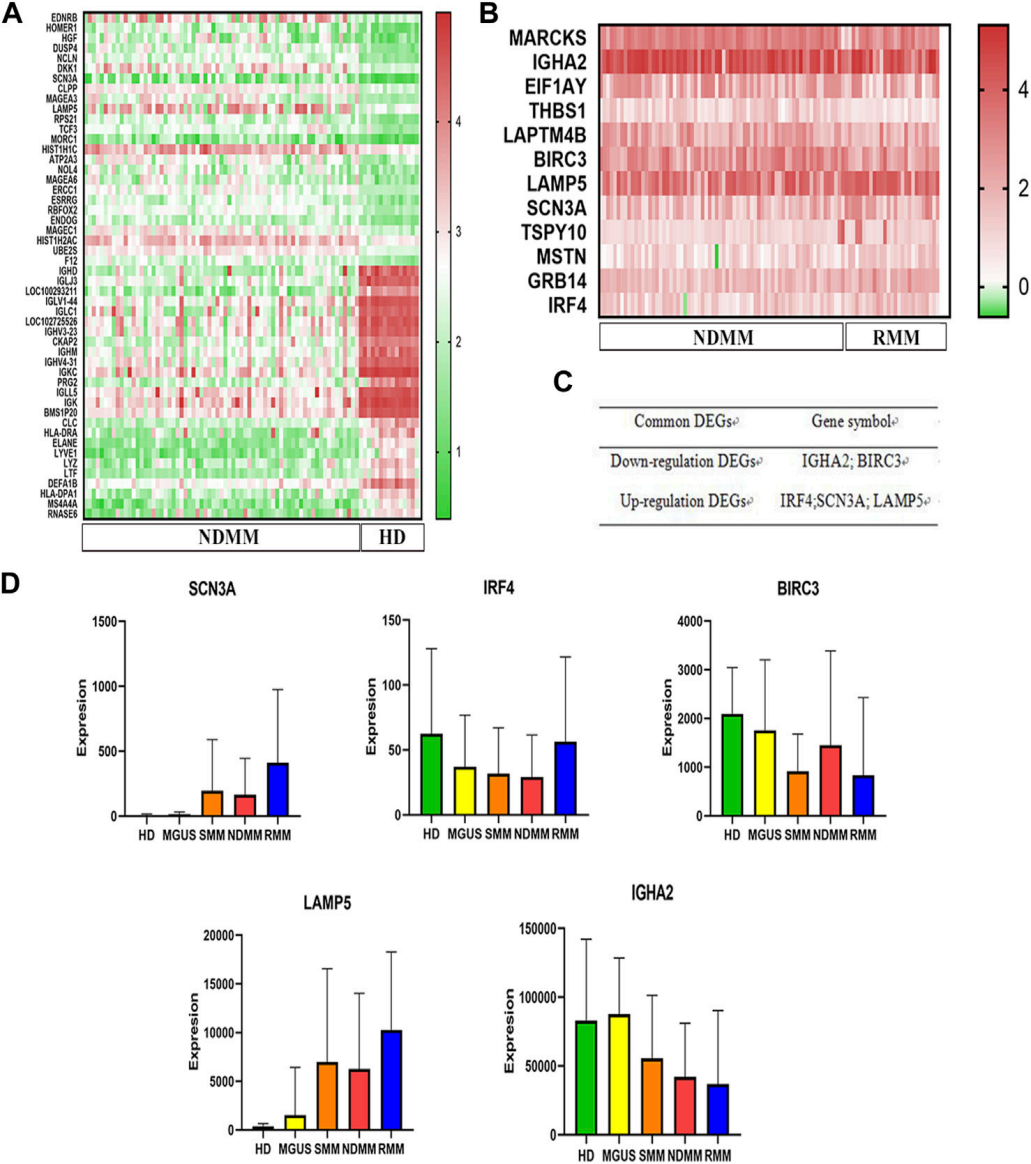
Screening of key genes for MM progression and recurrence. **(A)** The first 50 DEGs between NDMM and HD. **(B)** DEGs between RMM and NDMM. The change in color from green to red indicates a low to high level of gene expression. **(C)** The intersecting genes of the two groups of DEGs. **(D)** Expression of SCN3A, IRF4, BIRC3, LAMP5 and IGHA2 in HD, MGUS, SMM, NDMM, RMM (gene expression data from GSE6477). DEGs: differentially expressed genes, NDMM: newly diagnosed MM, HD: healthy donors, RMM: relapsed MM, MGUS: monoclonal gammopathy of undetermined significance, SMM: smoldering multiple myeloma.

After the analysis of the GSE6477 dataset, we identified 657 downregulated genes and 332 upregulated genes in NDMM vs. HD, and 6 downregulated genes and 6 upregulated genes in RMM vs. NDMM. We produced heat maps of the top50 DEGs for NDMM vs. HD and the full DEGs for RMM vs. NDMM (Figures 1A, B). The intersection genes of the two groups of DEGs are in Figure 1C. The downregulated intersection genes of the two groups of DEGs were immunoglobulin heavy chain alpha 2 (IGHA2) and baculoviral IAP repeat containing 3 (BIRC3), and the upregulated intersection genes were interferon regulatory factor (IRF) 4, sodium voltage-gated channel alpha subunit 3 (SCN3A) and LAMP5. Figure 1D shows the expression of SCN3A, IRF4, BIRC3, LAMP5 and IGHA2 in HD, MGUS, SMM, NDMM and RMM. LAMP5 shows the highest overall expression level and is more expressed in MGUS than HD, more expressed in SMM and NDMM than MGUS, and more expressed in RMM than NDMM, so we chose LAMP5 for the subsequent validation experiments.

### LAMP5 expression is upregulated in MM and correlates with poor prognosis

**FIGURE 2:**
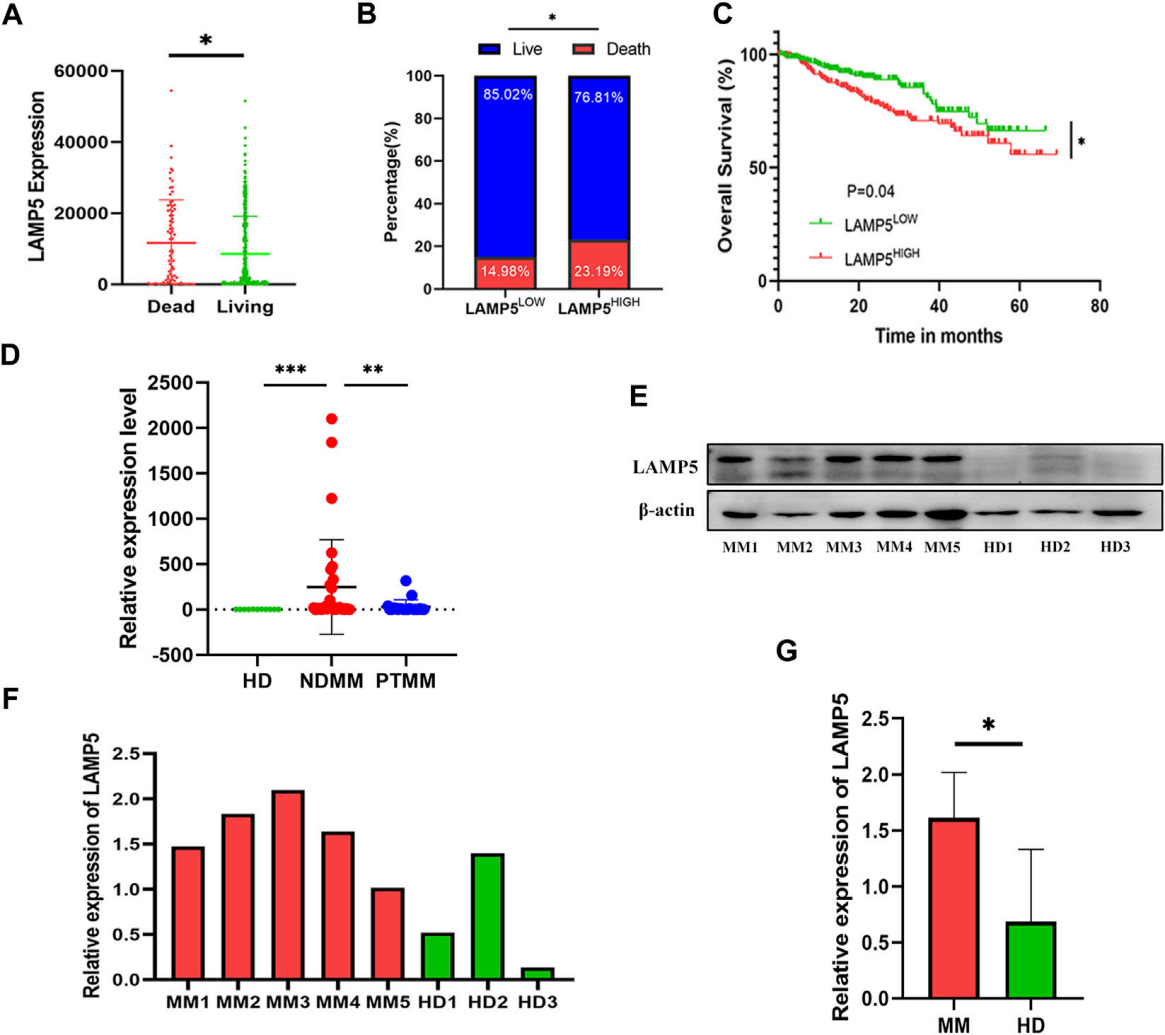
LAMP5 is highly expressed in MM and associated with poor prognosis. **(A)** The expression of LAMP5 was higher in patients in the dead group than in those in the living group. **(B)** Mortality in patients with high and low LAMP5 expression groups. **(C)** Survival analysis showed that patients in the LAMP5 high expression group had a worse prognosis. The above gene expression and survival data of MM patients were obtained from GSE4581. **(D)** qPCR showed the expression of LAMP5 in HD, NDMM, and PTMM. **(E)** Western blot assay to detect LAMP5 expression in NDMM and HD. **(F)** Histogram of relative expression of LAMP5 in NDMM versus HD patients. **(G)** The difference in expression of LAMP5 between NDMM and HD was statistically significant. *p < 0.05, **p < 0.01, ***p < 0.0005. HD: healthy donors, NDMM: newly diagnosed MM, PTMM: post-treatment MM.

We first analyzed the effect of LAMP5 in GSE4581 on the survival of NDMM patients, and divided NDMM into live and dead groups, and patients in the dead group had higher expression of LAMP5, and the difference was statistically significant (Figure 2A). We divided NDMM into LAMP5 high expression group and LAMP5 low expression group, and the mortality rate of LAMP5 high expression group and LAMP5 low expression group were 23.19% and 14.98%, respectively, and the difference was statistically significant (Figure 2B). Survival analysis was performed and we found that LAMP5 high expression group had worse prognosis, and the difference was statistically significant (Figure 2C). We next verified the expression of LAMP5 in HD, NDMM, and PTMM using qPCR. We found that the expression of LAMP5 was higher in NDMM than in HD, whereas the expression of LAMP5 was lower in PTMM than in NDMM, and the difference was statistically significant (Figure 2D). The general clinical data of the three groups are as follows (Table 2).

**TABLE 2:** Comparison of the general information of NDMM, PTMM and HD.

Characteristics	HD (*n* = 11)	NDMM (*n* = 32)	PTMM (*n* = 20)
Age(years)	58.64 ± 13.83	65.75 ± 9.56	65.70 ± 8.40
Sex(Male/Female)	5/6	21/11	16/4
WBC (×10^9^/L)	8.44 ± 3.57	5.08 ± 1.98	5.74 ± 1.33
HGB (g/L)	56.91 ± 12.76	83.34 ± 22.84	100.7 ± 19.56
PLT (×10^9^/L)	318.91 ± 102.66	165.13 ± 98.84	176.45 ± 64.22
ALB(g/L)	39.20 ± 3.33	34.41 ± 7.41	37.86 ± 3.40
Crea(umol/L)	64.69 ± 22.80	134.59 ± 134.31	89.13 ± 59.83
Ca^2+^(mmol/L)	2.16 ± 0.12	2.34 ± 0.40	2.21 ± 0.13
β2-MG(mg/L)	-	8.93 ± 8.95	3.85 ± 2.06
M protein type			
IgG	-	18	10
IgA	-	8	5
IgD	-	2	1
Light chain	-	4	4
DS Staging System			
I	-	2	1
II	-	6	4
III	-	24	15
ISS			
I	-	6	5
II	-	9	5
III	-	17	10

WBC, White blood cells; HGB, Hemoglobin; PLT, Platelets; ALB, Albumin; Crea, Creatinine; β2-MG, β2 microglobulin; DS, Durie-Salmon; ISS, International Staging System.

We summarized the correlation between high and low expression of LAMP5 gene and clinicopathological characteristics of MM patients (Table 3). With the exception of DS stage, there was no significant difference for any of the parameters.

**TABLE 3:** Correlation of LAMP5 expression with clinicopathological characteristics of MM patients in the validation cohort.

Characteristics	LAMP5 ^low^ (*n* = 16)	LAMP5 ^high^ (*n* = 16)	*p*-value
Age(years)	64.31 ± 11.12	67.19 ± 7.81	0.4729
Sex(Male/Female)	9/7	12/4	0.4578
WBC (×10^9^/L)	5.38 ± 2.40	4.78 ± 1.47	0.6689
HGB (g/L)	90.13 ± 26.60	76.56 ± 16.52	0.2201
PLT (×10^9^/L)	186.31 ± 116.52	143.94 ± 75.22	0.2240
ALB(g/L)	35.99 ± 7.69	32.83 ± 7.01	0.2133
Crea(umol/L)	117.08 ± 97.41	152.10 ± 164.73	0.7879
Ca^2+^(mmol/L)	2.40 ± 0.30	2.29 ± 0.48	0.1457
β2-MG(mg/L)	7.94 ± 6.56	9.92 ± 10.98	0.6420
M protein type			
IgG	8	10	0.7244
IgA	5	3	0.6851
IgD	1	1	>0.9999
Light chain	2	2	>0.9999
DS Staging System			
I	2	0	0.4839
II	5	1	0.1719
III	9	15	0.0373
ISS			
I	4	2	0.6539
II	5	4	>0.9999
III	7	10	0.4795

WBC, White blood cells; HGB, Hemoglobin; PLT, Platelets; ALB, Albumin; Crea, Creatinine; β2-MG, β2 microglobulin; DS, Durie-Salmon; ISS, International Staging System.

We next examined the expression of LAMP5 protein in 5 NDMM and 3 HD using western blot and found that the expression of LAMP5 protein was also higher in NDMM than in HD (Figures 2E–G).

### Expression of LAMP5 in MM cell lines and knockdown of LAMP5

**FIGURE 3:**
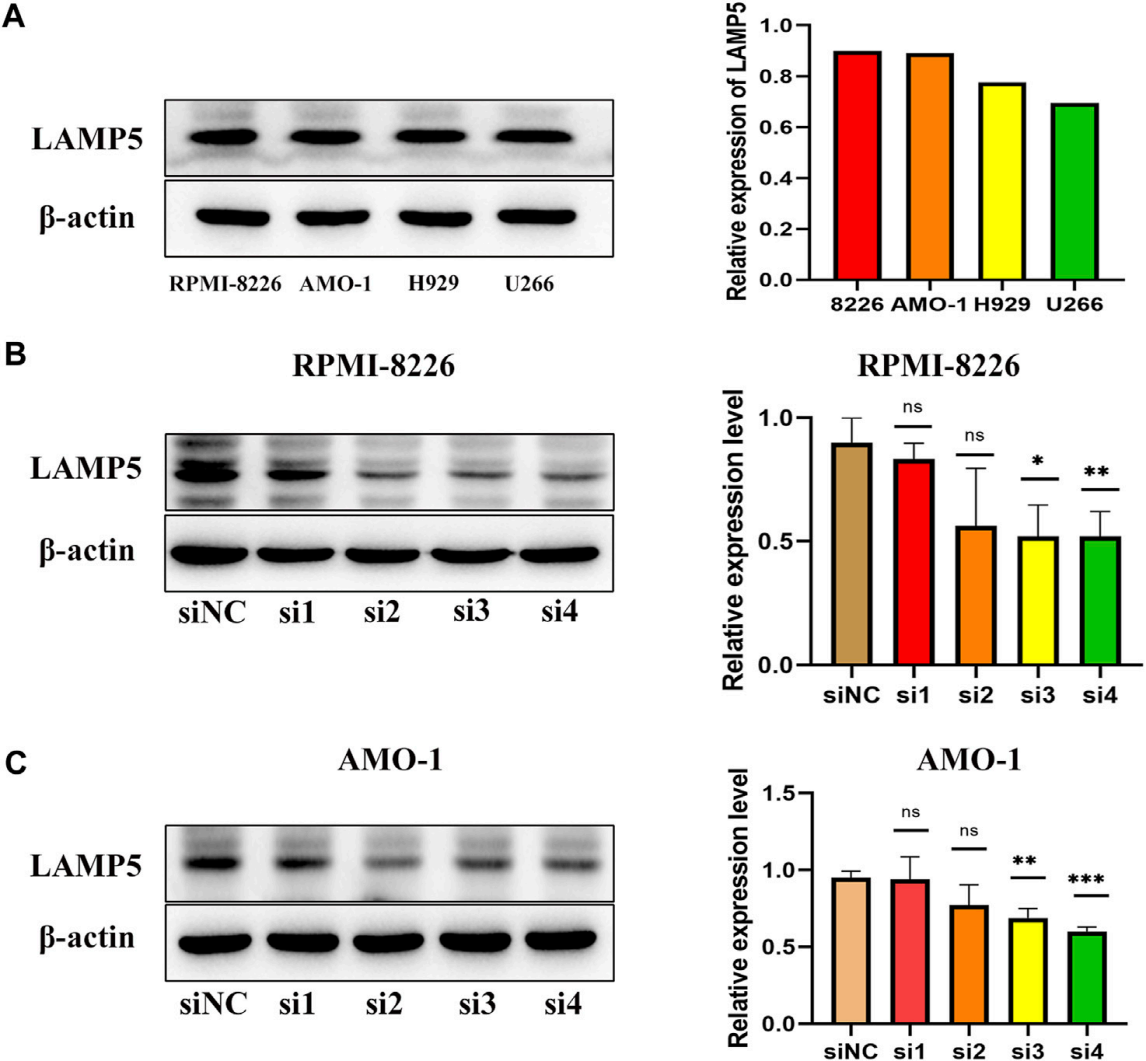
Expression of LAMP5 in MM cell lines and screening of siRNA for knockdown of LAMP5. **(A)** Expression of LAMP5 in RPMI-8226, AMO-1, H929 and U266. **(B,C)** We used 4 different siRNA sequences to interfere with LAMP5 expression in MM cell lines and found that si3 and si4 were the most efficient. *p < 0.05, **p < 0.01, ***p < 0.0005, ns: no significance.

We next examined the expression of LAMP5 in four MM cell lines, RPMI-8226, AMO-1, H929 and U266, and found that LAMP5 expression was highest in RPMI-8226 and AMO-1, followed by H929 and lowest in U266 (Figure 3A). We selected RPMI-8226 and AMO-1 for the follow-up experiments. We used four different siRNA sequences for cell transfection and found that si3 and si4 were the most efficient in reducing LAMP5 expression, so we selected these two sequences for the next experiments (Figures 3B, C).

### Knockdown of LAMP5 gene promotes apoptosis in MM cells

**FIGURE 4:**
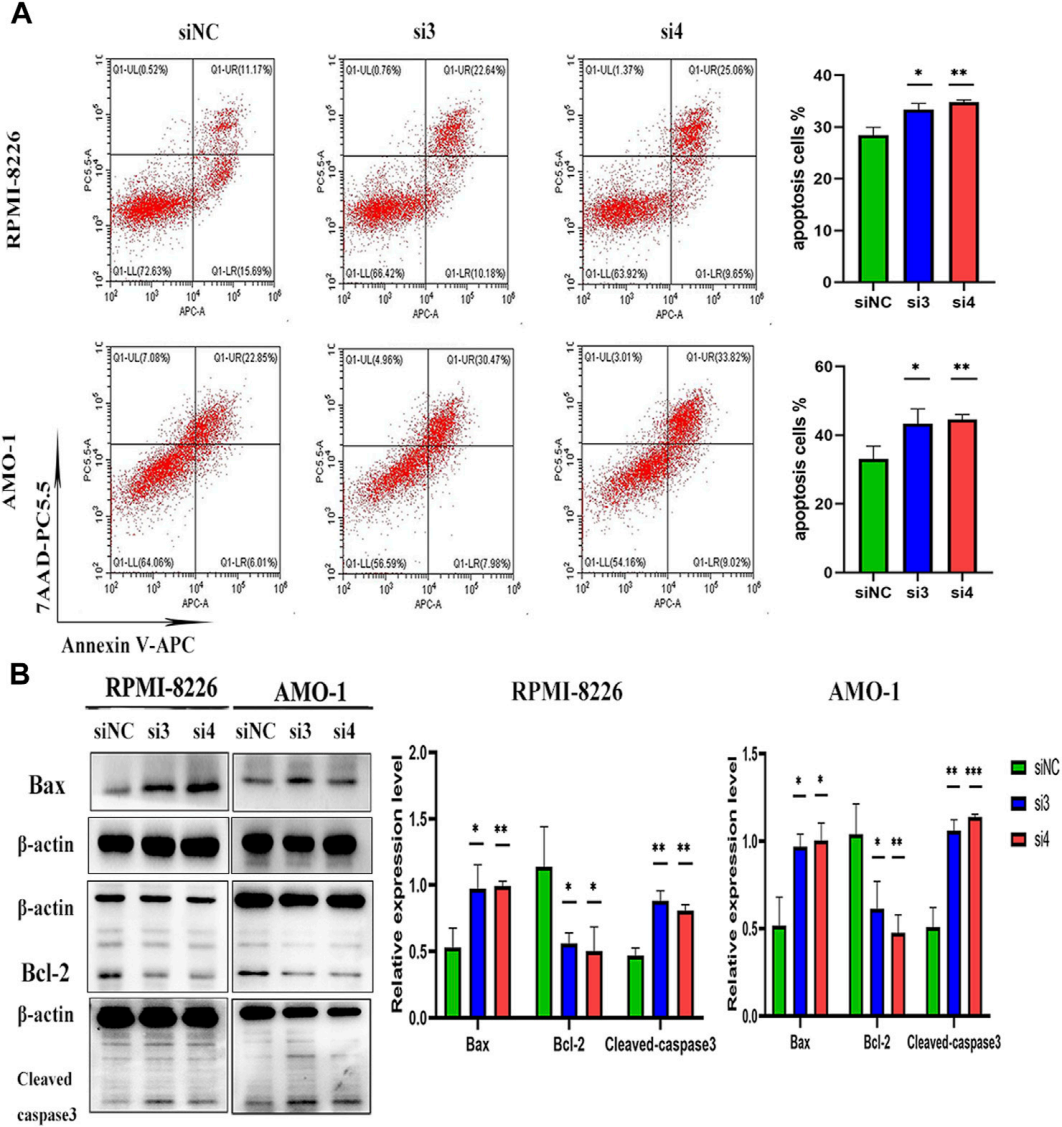
Knockdown of LAMP5 in MM promotes apoptosis. **(A)** Apoptosis rates were detected using flow cytometry and were increased in the si3 and si4 groups compared to siNC. **(B)** The expression of apoptosis-related proteins was detected using western blot, and the expression of Bax and cleaved-caspase3 proteins was increased and Bcl-2 protein expression was decreased in the si3 and si4 groups compared to siNC. *p < 0.05, **p < 0.01, ***p < 0.0005.

We interfered with the expression of LAMP5 gene in RPMI-8226 and AMO-1 and then detected apoptosis using flow cytometry, and the apoptosis rate was increased in both si3 and si4 groups in both cell lines compared to siNC. In RPMI-8226, the apoptosis rate was 28.44% ± 1.53% for siNC and 33.39% ± 1.17% and 34.80% ± 0.44% for si3 and si4, respectively, while in AMO-1, the apoptosis rates were 33.05% ± 3.74%, 43.34% ± 4.27% and 44.52% ± 1.47% for siNC, si3 and si4, respectively (Figure 4A). Our experiment was repeated three times. The differences were statistically significant. In both cell lines, knockdown of LAMP5 increased the expression of the pro-apoptotic protein bax, downregulated the expression of the anti-apoptotic protein bcl-2, and upregulated the expression of cleaved caspase3 (Figure 4B). Therefore, we concluded that knockdown of LAMP5 gene could lead to apoptosis in MM cells.

### Knockdown of LAMP5 gene has no effect on the cell cycle of MM cells

**FIGURE 5:**
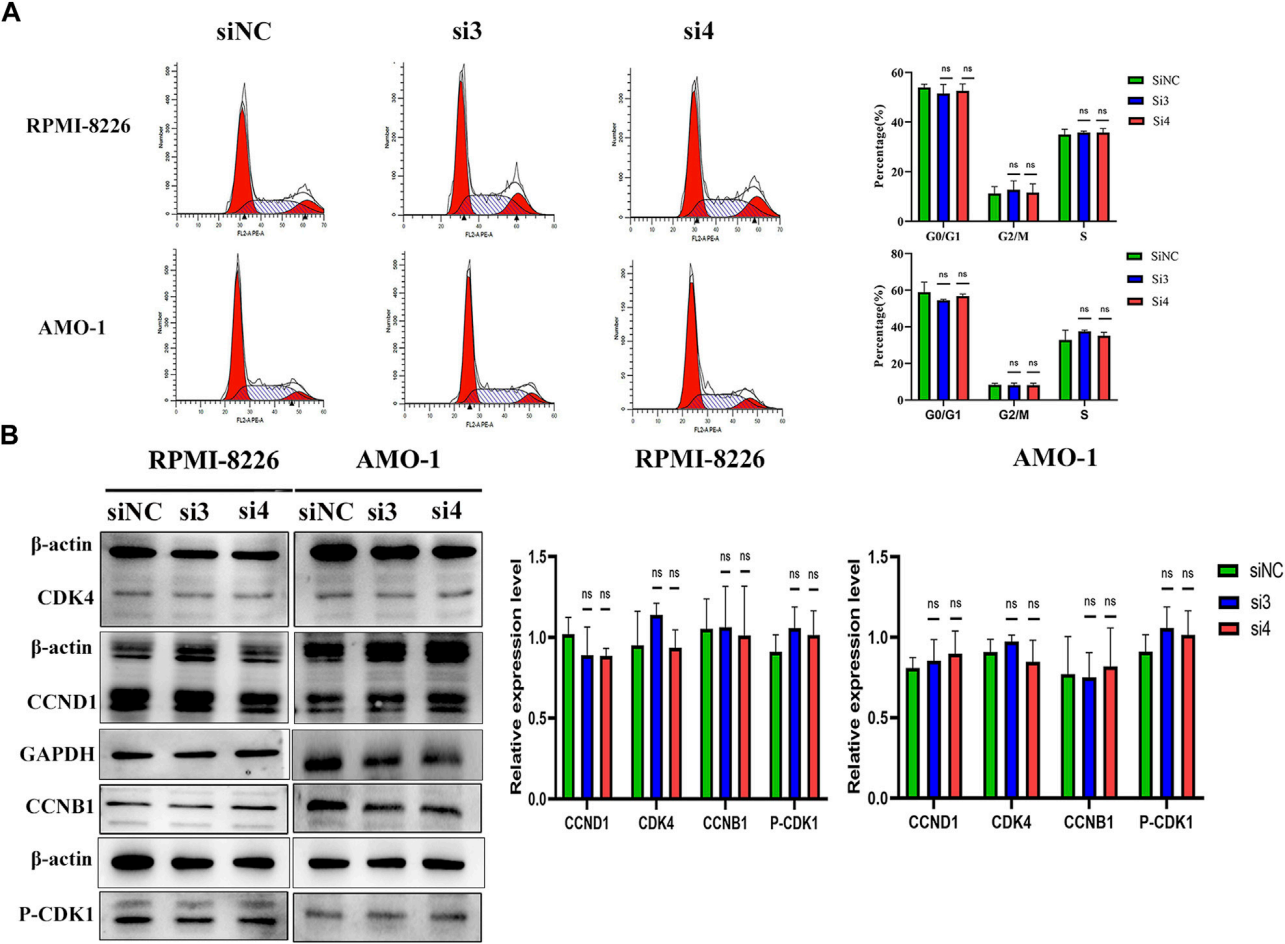
Knockdown of LAMP5 expression had no effect on the MM cell cycle. **(A)** Flow cytometry was used to examine the cell cycle and found no significant changes in the proportion of cells in each cell cycle phase for SiNC, si3 and si4. **(B)** The expression of cell cycle-associated proteins was detected using western blot. The expression of cell cycle-related proteins CCND1, CDK4, CCNB1 and p-CDK1 did not change significantly in the three groups. ns: no significance.

We next explored the effect of knocking down the LAMP5 gene on the MM cell cycle. We first performed flow cytometry to detect cell cycle and we found that the proportion of cells in each cell cycle phase did not change much in both si3 and si4 groups compared to siNC. In RPMI-8226, the proportions of G0/G1 phase, G2/M phase and S phase cells were 53.94% ± 1.36%, 11.12% ± 2.87% and 34.93% ± 2.17% in the siNC group, 51.53% ± 3.67%, 12.65% ± 3.68% and 35.82% ± 0.55% in the si3 group, respectively, while 52.67% ± 2.71%, 11.54% ± 3.56% and 35.78% ± 1.66% in the si4 group, respectively (Figure 5A). In AMO-1, the proportions of G0/G1, G2/M and S-phase cells were 59.00% ± 5.43%, 8.20% ± 0.88% and 32.79% ± 5.41% in the siNC group, 54.43% ± 0.53%, 8.07% ± 1.15% and 37.51% ± 0.71% in the si3 group, respectively, while 56.85% ± 1.03%, 8.07% ± 1.09% and 35.07% ± 1.93% in the si4 group, respectively (Figure 5A). We next examined the expression of key cell cycle proteins CCND1 and CDK4 in G0/G1 phase and CCNB1 and CDK1 in G2/M phase and found that the four cell cycle proteins were also not statistically different in each of the 3 groups (Figure 5B). Knockdown of the LAMP5 gene had no effect on the MM cell cycle.

### Knockdown of LAMP5 reduces P38 protein expression in MM

**FIGURE 6:**
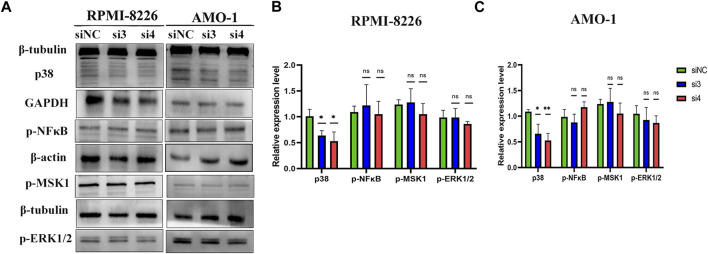
LAMP5 may exert its pro-tumor effects partly through P38 protein. **(A–C)** Knockdown of LAMP5 expression in MM cell lines showed a decrease in p38 protein expression as well, but p-NFκB, p-msk1 and p-ERK1/2 protein expression did not show statistically significant changes in the three groups. *p < 0.05, **p < 0.01, ns: no significance.

To explore the mechanism of LAMP5 promoting apoptosis in MM cells, we examined the expression of four proteins, p-ERK1/2, p38, p-MSK1, and p-NF-κB. We found that knockdown of LAMP5 in RPMI-8226 and AMO-1 decreased the expression of p38 protein, and the difference was statistically significant (Figure 6). However, knockdown of LAMP5 had no effect on the expression of three proteins, p-ERK1/2, p-MSK1, and p-NF-κB (Figure 6). Knockdown of LAMP5 may promote apoptosis in MM cells by reducing the expression of p38 protein.

## Discussion and conclusion

Almost 200 years after MM was first reported in 1844, awareness of MM has gradually increased (22). MM is a progressive disease in which genetic alterations occurring in the germinal centers are not sufficient to cause myeloma, but rather give cell proliferative potential to normally non-dividing plasma cells, a pre-malignant state known as MGUS, an asymptomatic stage, followed by an intermediate stage, SMM, and eventually progressing to symptomatic NDMM (22). MGUS is present in approximately 2% of people over 50 years of age, and MGUS progresses to MM at a rate of 1% per year (23, 24). Globally, cases of incident and death from MM have more than doubled in the last 30 years (25). At the beginning of this century, the average overall survival of MM patients was about 3 years, when the main therapeutic agents were bortezomib, lenalidomide and thalidomide (26). The subsequent application of treatments including daratumumab, chimeric antigen receptor T cells, autologous hematopoietic stem cell transplantation, and bispecific T-cell engagers has greatly improved the survival of MM patients (27, 28, 29). However, regardless of the improvement in treatment modalities, MM is still an incurable tumor and it is inevitable that patients will experience recurrence. Genetic alterations leading to malignant cloning of plasma cells are one of the main pathogenic mechanisms of MM, and the treatment of MM patients needs to be individualized based on molecular characteristics (22, 30).

In this study, we tried to find DEGs in NDMM compared to HD, and also in RMM compared to NDMM, where alterations in these genes may lead to MM progression and recurrence. We finally identified 2 downregulated intersectional DEGs, IGHA2 and BIRC3, and 3 upregulated intersectional DEGs, IRF4, SCN3A, LAMP5. BIRC3, IRF4 have been studied in MM (31, 32). LAMP5 expression was the highest among these genes and was progressively higher as MM progressed. MGUS had higher LAMP5 expression than HD, SMM and NDMM had higher LAMP5 expression than MGUS, while RMM had the highest LAMP5 expression. LAMP5 has not been studied in MM, so we chose LAMP5 for the follow-up study. We collected bone marrow from patients with NDMM, HD and PTMM and performed qPCR analysis of LAMP5, and found that the expression of LAMP5 was higher in NDMM than in HD, and decreased after treatment. Western blotting assay also found more expression of LAMP5 in NDMM than in HD. This is also consistent with the expression of the GSE6477 data. Combined with the survival data of MM in GSE4581, we found that the survival of patients with high expression of LAMP5 was lower than that of the low expression group. We summarized the correlation between high and low expression of LAMP5 gene and clinicopathological characteristics of MM patients. With the exception of DS stage, there was no significant difference for any of the parameters. LAMP5 may be one of the key genes in the development of MM as well as its recurrence.

Our next experiments showed that interference with LAMP5 gene expression in MM cell lines promoted apoptosis in MM cells, but had no effect on the cell cycle. The mechanism by which silencing LAMP5 in MM cells leads to increased apoptosis is by increasing the expression of the pro-apoptotic protein Bax and decreasing the expression of the anti-apoptotic protein BCL-2, suggesting that silencing LAMP5 inhibits MM cell apoptosis in part through the mitochondrial pathway of apoptosis. So what is the mechanism by which LAMP5 promotes MM progression? In MLL leukemia, LAMP5 is associated with poor prognosis, and interference with LAMP5 expression reduces the expression of p38 and p-NF-κB (17, 18). NF-κB signaling pathway is closely related to the pathogenesis of MM, and p38, MSK1, and ERK1/2 proteins are associated with NFκB expression (14, 33–35). Therefore, we selected p38, p-MSK1, p-NFκB, and p-ERK1/2 for the assay to determine whether knockdown of LAMP5 in MM would act through the above proteins. We found that in MM LAMP5 exerts its pro-oncogenic effect probably through activation of p38, but p-MSK1, p-NF-κB, and p-ERK1/2 did not show changes in expression in our study. In MM, P38 plays a pro-cancer role (36, 37). P38 regulates osteoclast and osteoblast activity in MM and induces bone destruction, and inhibition of p38 expression reduces MM bone disease (38).

In conclusion, we have explored for the first time the role and mechanism of action of LAMP5 in MM. The expression of LAMP5 gradually increased with the progression of MGUS to SMM, NDMM, and RMM, and high expression of LAMP5 was associated with poor prognosis of MM. Knockdown of LAMP5 induces apoptosis in MM cells, which may act in part by decreasing the expression of p38 protein, and LAMP5 may become a new therapeutic target for MM.

## Data Availability

The original contributions presented in the study are included in the article/Supplementary Material, further inquiries can be directed to the corresponding author.
