# Supplementary material for: LAMP5 may promote MM progression by activating p38
Source: Pathol Oncol Res. 2023 Mar 22;29:1611083. doi: 10.3389/pore.2023.1611083 (PMC10073510; doi:10.3389/pore.2023.1611083)
Supplement: Supplementary file 1 [file DataSheet3.ZIP › AMO1 cell cycle/1/amo1 nc 1 ╖╓╬÷.pdf]

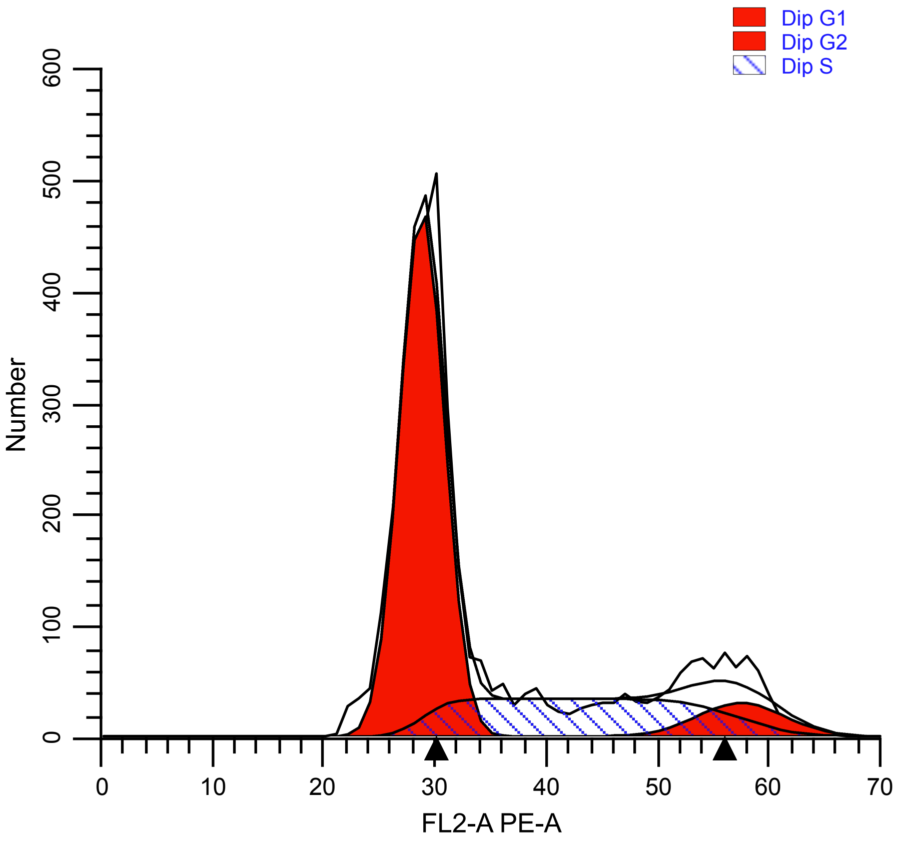

File analyzed: AMO1 NC 1.fcs
Date analyzed: 10-Oct-2022
Model: 1nn0n_DSD
Analysis type: Manual analysis
Auto Linearity: No

Ploidy Mode: First cycle is diploid

Diploid: 100.00 %
Dip G1: 65.23 % at 28.69
Dip G2: 8.22 % at 57.38
Dip S: 26.55 % G2/G1: 2.00
%CV: 6.91

Total S-Phase: 26.55 %
Total B.A.D.: 0.00 % no debris no aggs

Debris: %
Aggregates: %
Modeled events: 3644
All cycle events: 3644
Cycle events per channel: 123
RCS: 3.488

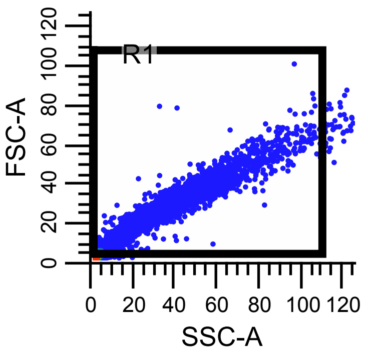

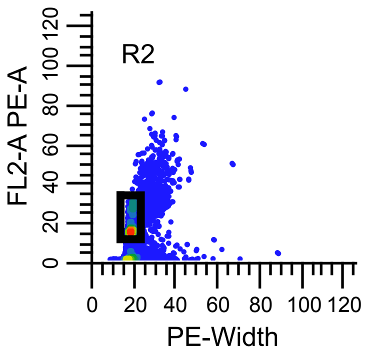

ModFit LT V4.1.7(Mac)
