# Supplementary material for: LAMP5 may promote MM progression by activating p38
Source: Pathol Oncol Res. 2023 Mar 22;29:1611083. doi: 10.3389/pore.2023.1611083 (PMC10073510; doi:10.3389/pore.2023.1611083)
Supplement: Supplementary file 1 [file DataSheet3.ZIP › AMO1 cell cycle/1/amo1 si3 1 ╖╓╬÷.pdf]

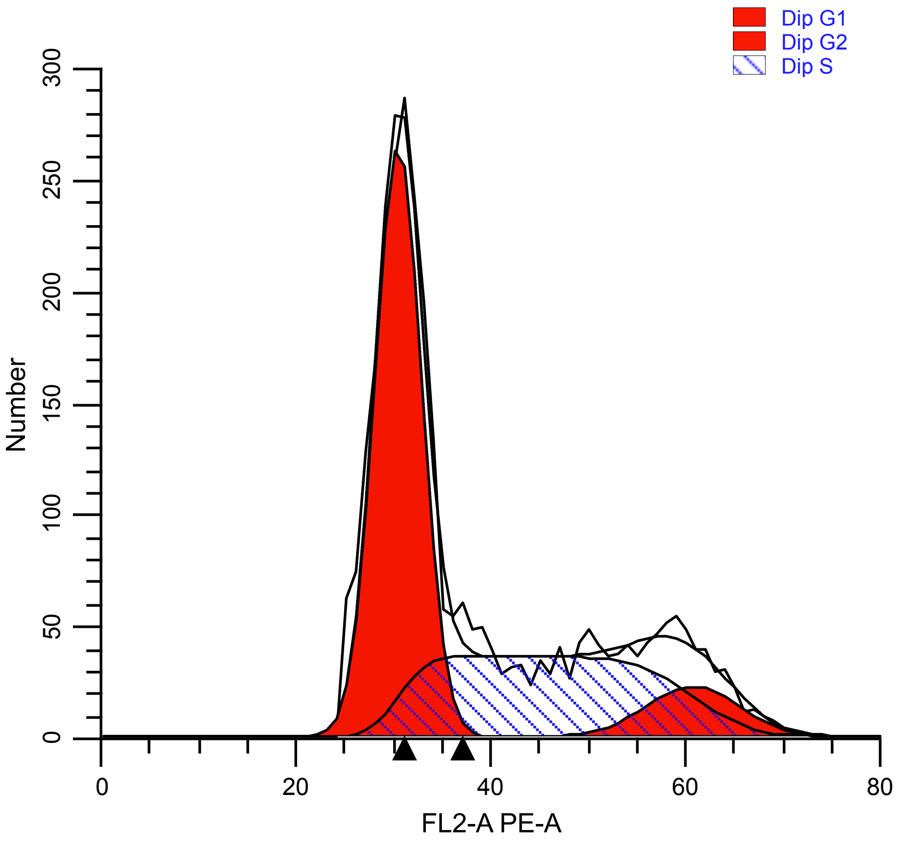


File analyzed: AMO1 SI3  1.fcs
Date analyzed: 10-Oct-2022
Model: 1nn0n_DSD
Analysis type: Manual analysis
Auto Linearity: No

Ploidy Mode: First cycle is diploid

Diploid: 100.00 %
Dip G1: 54.17 % at 30.34
Dip G2: 9.11 % at 60.68
Dip S: 36.73 %   G2/G1: 2.00
%CV: 7.88

Total S-Phase: 36.73 %
Total B.A.D.: 0.00 %   no debris no aggs

Debris:  %
Aggregates:  %
Modeled events: 2962
All cycle events: 2962
Cycle events per channel: 95
RCS: 3.253

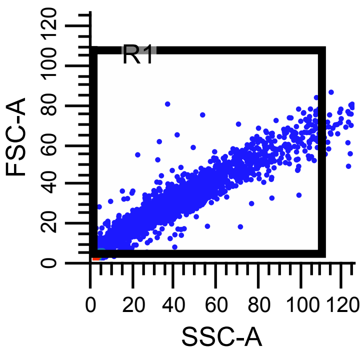


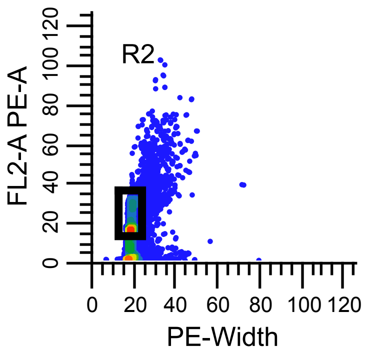


ModFit LT V4.1.7(Mac)
