# Supplementary material for: LAMP5 may promote MM progression by activating p38
Source: Pathol Oncol Res. 2023 Mar 22;29:1611083. doi: 10.3389/pore.2023.1611083 (PMC10073510; doi:10.3389/pore.2023.1611083)

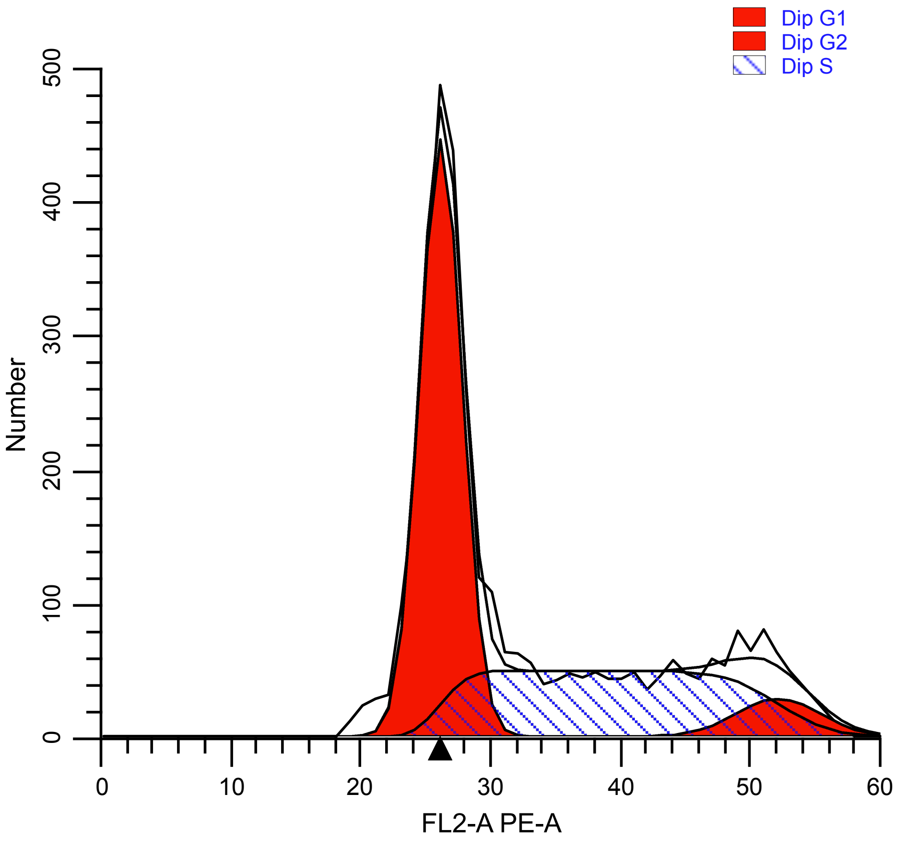

File analyzed: AMO1 si3 5.fcs
Date analyzed: 10-Oct-2022
Model: 1nn0n_DSD
Analysis type: Manual analysis
Auto Linearity: No

Ploidy Mode: First cycle is diploid

Diploid: 100.00 %
Dip G1: 55.04 % at 26.05
Dip G2: 6.82 % at 52.09
Dip S: 38.14 %   G2/G1: 2.00
%CV: 6.20

Total S-Phase: 38.14 %
Total B.A.D.: 0.00 %   no debris no aggs

Debris: %
Aggregates: %
Modeled events: 3338
All cycle events: 3338
Cycle events per channel: 123
RCS: 3.640

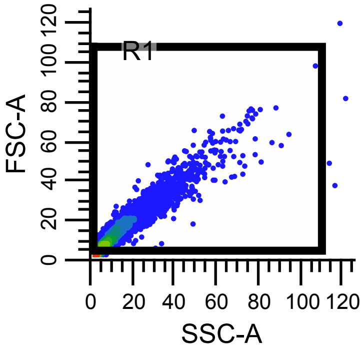

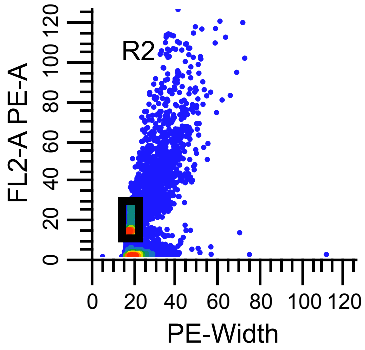

Supplement: Supplementary file 1 [file DataSheet3.ZIP › AMO1 cell cycle/2/amo si3 5 ╖╓╬÷.pdf]
