# Supplementary material for: LAMP5 may promote MM progression by activating p38
Source: Pathol Oncol Res. 2023 Mar 22;29:1611083. doi: 10.3389/pore.2023.1611083 (PMC10073510; doi:10.3389/pore.2023.1611083)

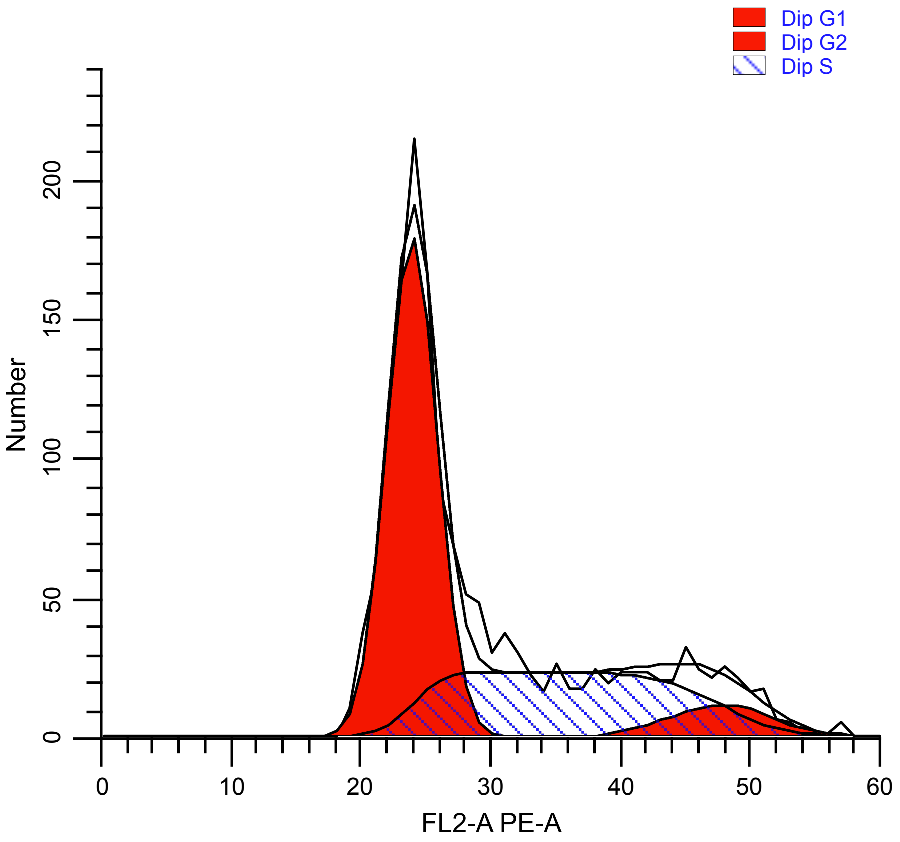

File analyzed: AMO1 si4 5.fcs
Date analyzed: 10-Oct-2022
Model: 1nn0n_DSD
Analysis type: Manual analysis
Auto Linearity: No

Ploidy Mode: First cycle is diploid

Diploid: 100.00 %
Dip G1: 57.01 % at 23.82
Dip G2: 7.23 % at 47.64
Dip S: 35.76 %   G2/G1: 2.00
%CV: 8.05

Total S-Phase: 35.76 %
Total B.A.D.: 0.00 %   no debris no aggs

Debris: %
Aggregates: %
Modeled events: 1532
All cycle events: 1532
Cycle events per channel: 62
RCS: 1.132

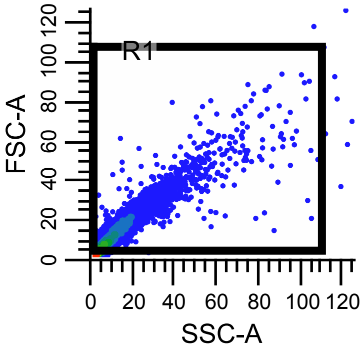

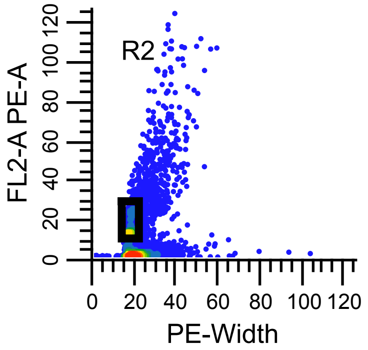

Supplement: Supplementary file 1 [file DataSheet3.ZIP › AMO1 cell cycle/2/amo1 si4 5 ╖╓╬÷.pdf]
