# Supplementary material for: LAMP5 may promote MM progression by activating p38
Source: Pathol Oncol Res. 2023 Mar 22;29:1611083. doi: 10.3389/pore.2023.1611083 (PMC10073510; doi:10.3389/pore.2023.1611083)
Supplement: Supplementary file 1 [file DataSheet3.ZIP › AMO1 cell cycle/3/amo1 si3 4 ╖╓╬÷.pdf]

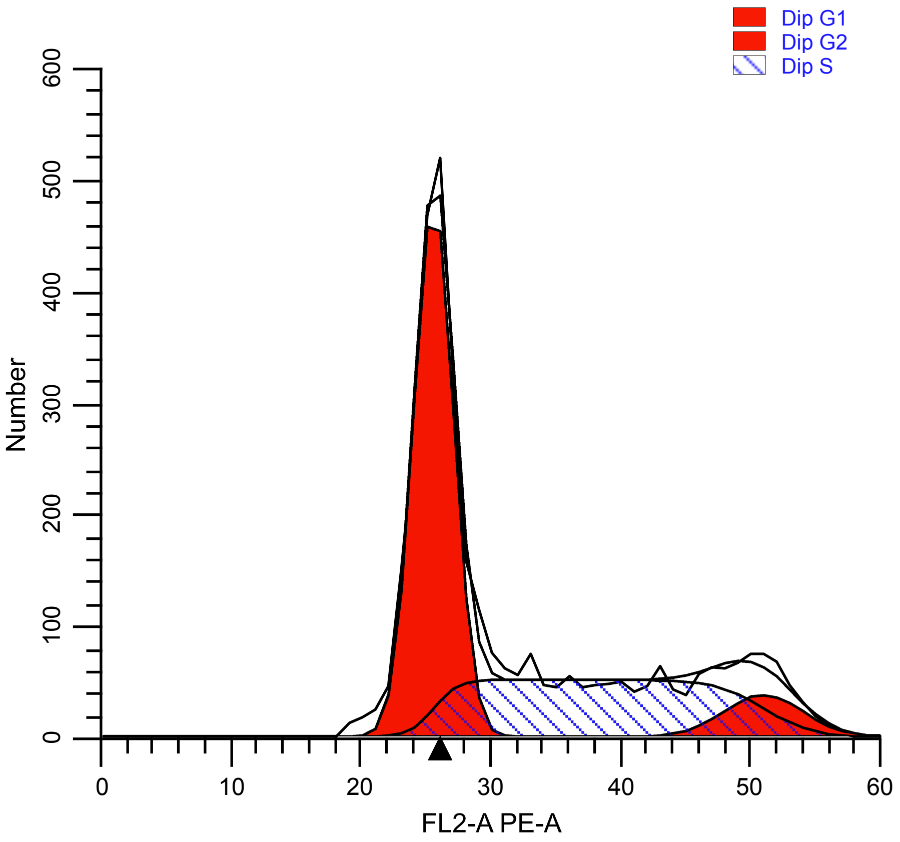

File analyzed: AMO1 si3 4.fcs
Date analyzed: 10-Oct-2022
Model: 1nn0n_DSD
Analysis type: Manual analysis
Auto Linearity: No

Ploidy Mode: First cycle is diploid

Diploid: 100.00 %
Dip G1: 54.08 % at 25.48
Dip G2: 8.28 % at 50.95
Dip S: 37.65 % G2/G1: 2.00
%CV: 5.93

Total S-Phase: 37.65 %
Total B.A.D.: 0.00 % no debris no aggs

Debris: %
Aggregates: %
Modeled events: 3426
All cycle events: 3426
Cycle events per channel: 129
RCS: 2.109

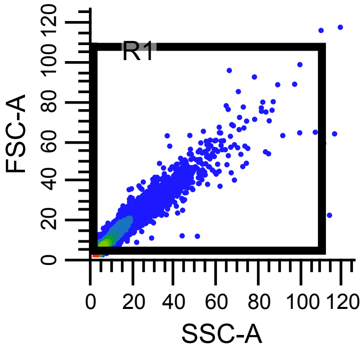

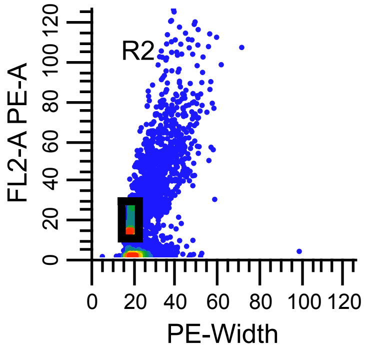

ModFit LT V4.1.7(Mac)
