# Supplementary figures and images for: LAMP5 may promote MM progression by activating p38 (part 1 of 2)
Source: Pathol Oncol Res. 2023 Mar 22;29:1611083. doi: 10.3389/pore.2023.1611083 (PMC10073510; doi:10.3389/pore.2023.1611083)

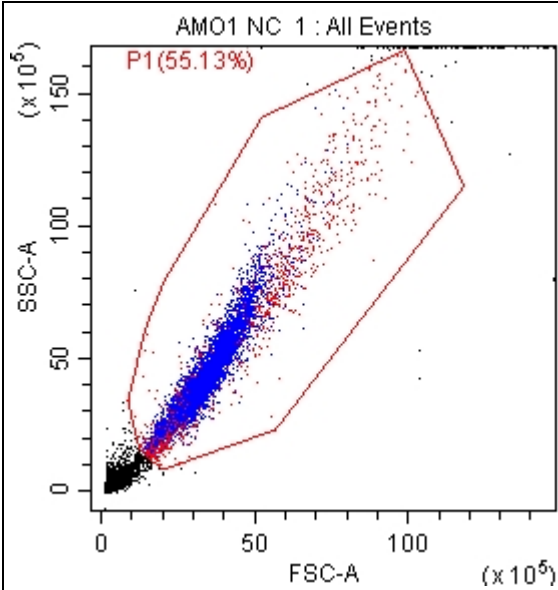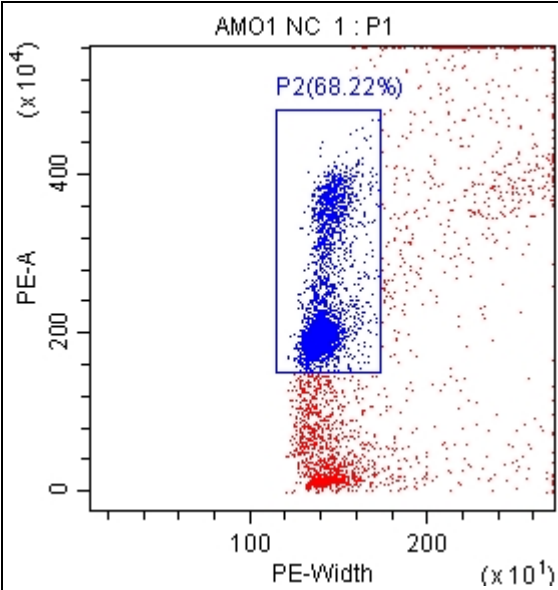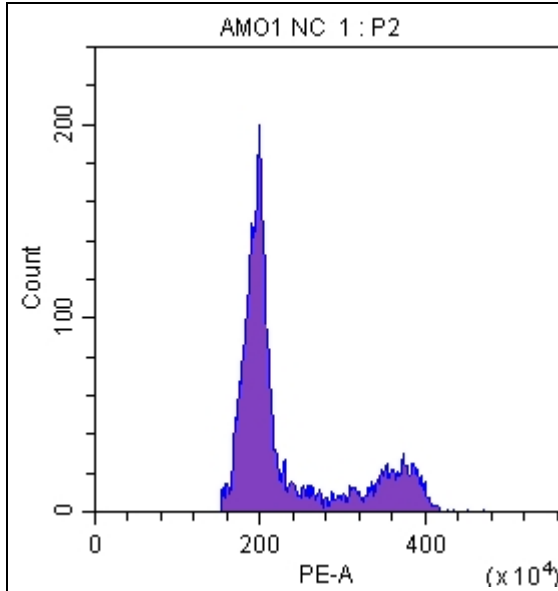

试管名称: AMO1 NC 1

样本ID:

| 群体             | 颗粒数   | %总数     | %父群     |
|----------------|-------|---------|---------|
| ▼ ● All Events | 10000 | 100.00% | 100.00% |
| ▼ ● P1         | 5513  | 55.13%  | 55.13%  |
| ● P2           | 3761  | 37.61%  | 68.22%  |

Supplement: Supplementary file 1 [file DataSheet3.ZIP › AMO1 cell cycle/1/AMO1 NC 1.pdf]

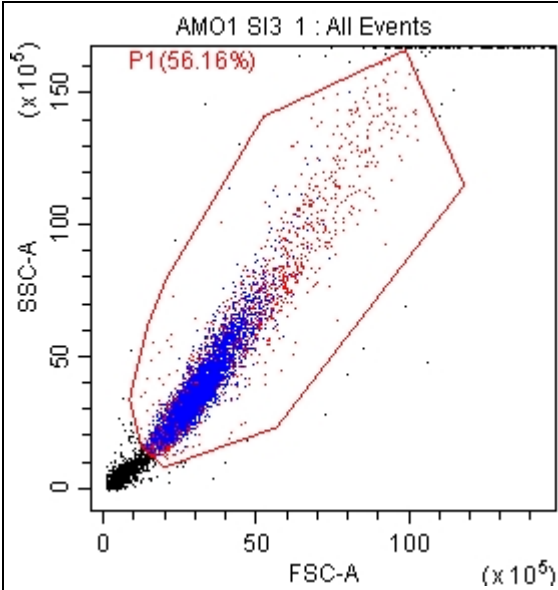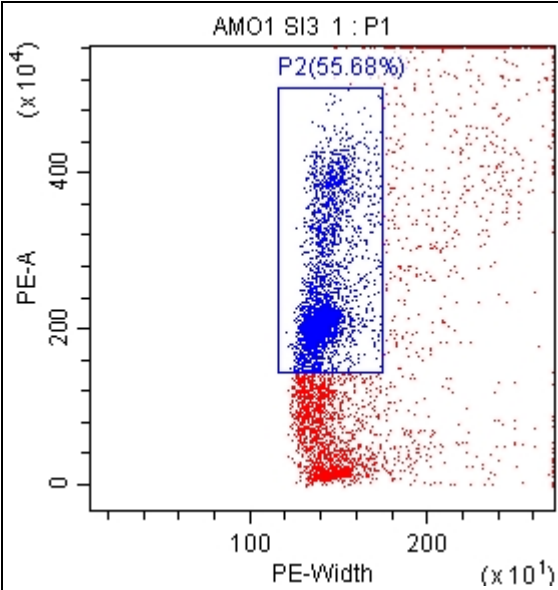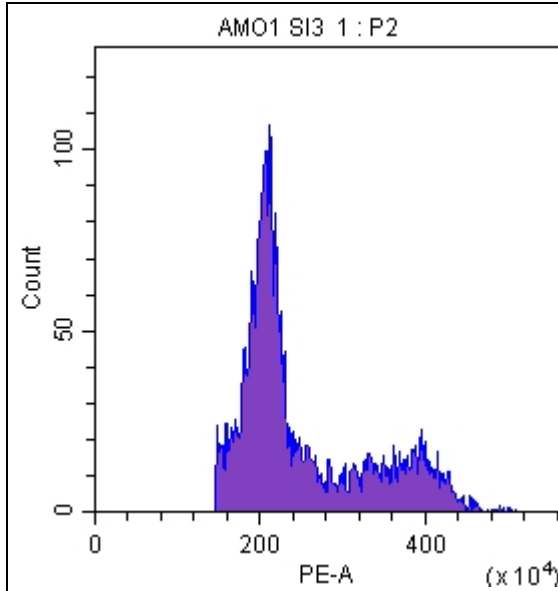

试管名称: AMO1 SI3 1

样本ID:

| 群体             | 颗粒数   | %总数     | %父群     |
|----------------|-------|---------|---------|
| ▼ ● All Events | 10000 | 100.00% | 100.00% |
| ▼ ● P1         | 5616  | 56.16%  | 56.16%  |
| ● P2           | 3127  | 31.27%  | 55.68%  |

Supplement: Supplementary file 1 [file DataSheet3.ZIP › AMO1 cell cycle/1/AMO1 SI3 1.pdf]

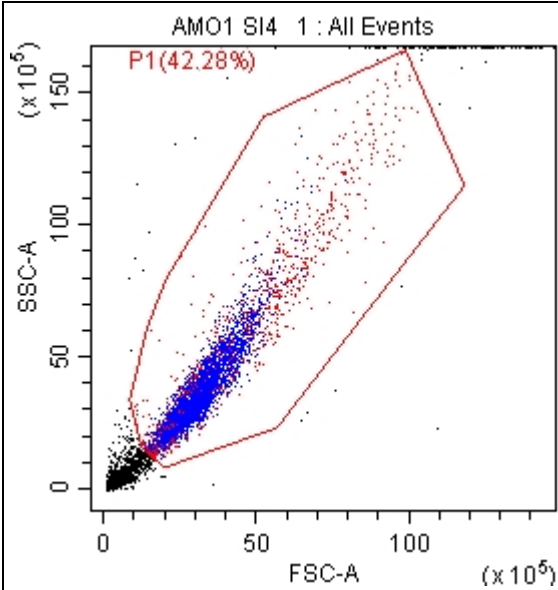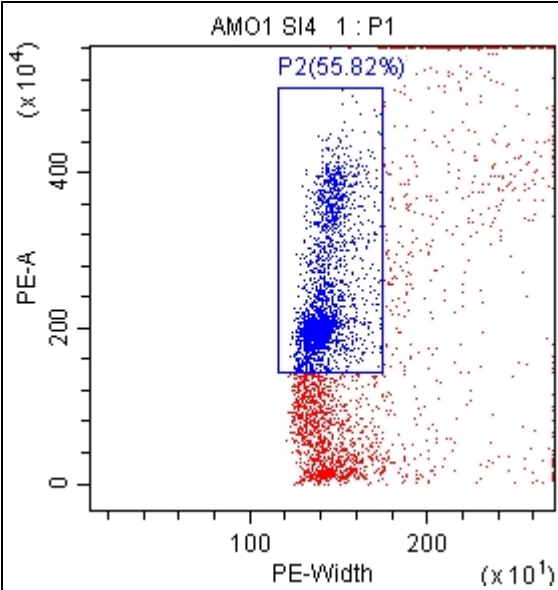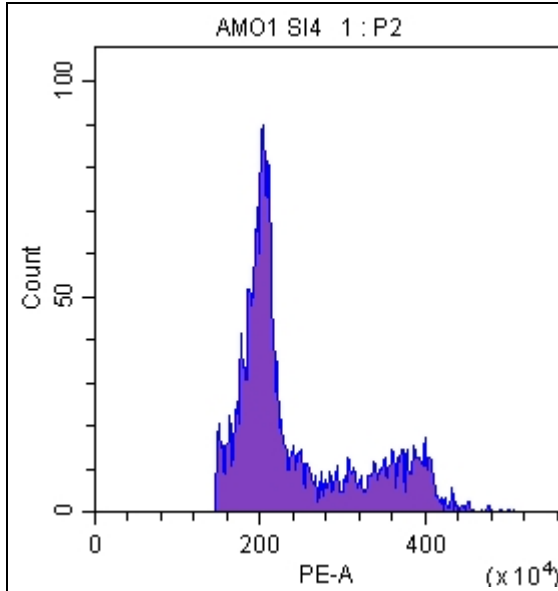

试管名称: AMO1 SI4 1

样本ID:

| 群体             | 颗粒数   | %总数     | %父群     |
|----------------|-------|---------|---------|
| ▼ ● All Events | 10000 | 100.00% | 100.00% |
| ▼ ● P1         | 4228  | 42.28%  | 42.28%  |
| ● P2           | 2360  | 23.60%  | 55.82%  |

Supplement: Supplementary file 1 [file DataSheet3.ZIP › AMO1 cell cycle/1/AMO1 SI4 1.pdf]

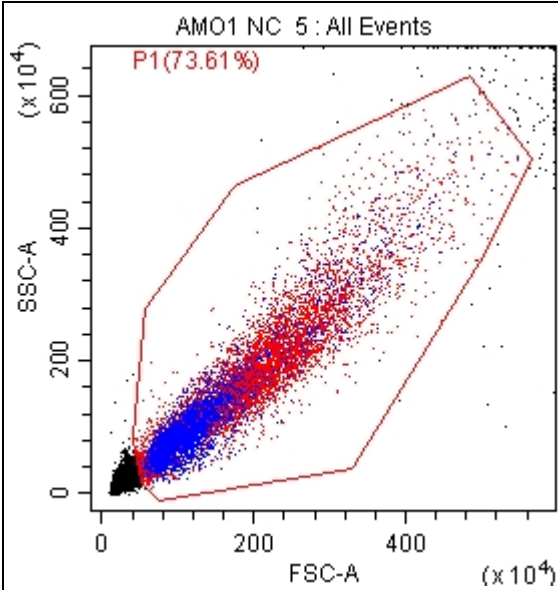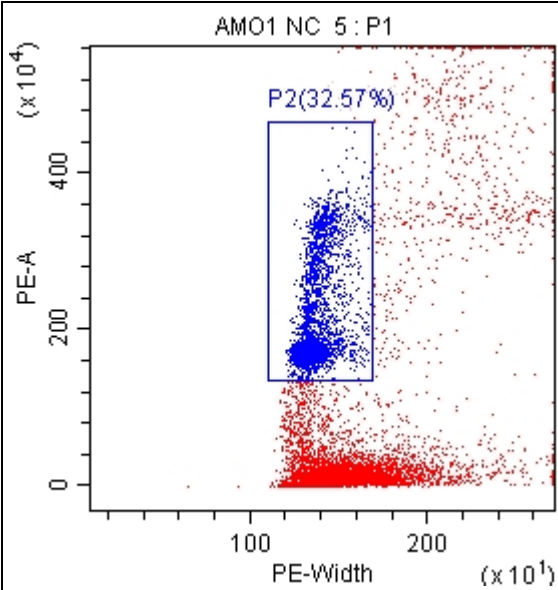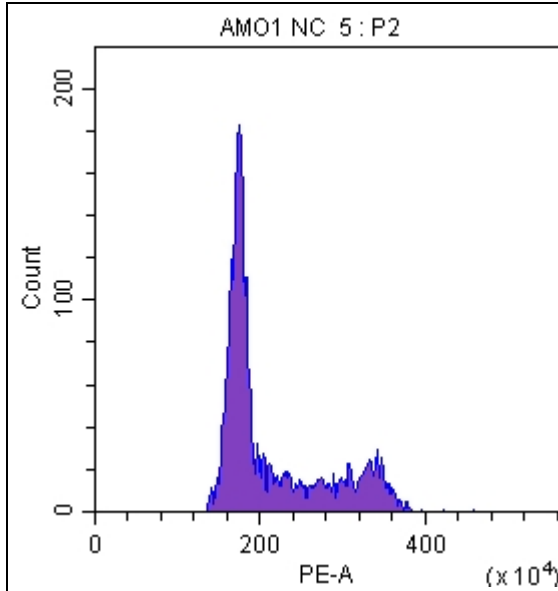

试管名称: AMO1 NC 5

样本ID:

| 群体             | 颗粒数   | %总数     | %父群     |
|----------------|-------|---------|---------|
| ▼ ● All Events | 13586 | 100.00% | 100.00% |
| ▼ ● P1         | 10000 | 73.61%  | 73.61%  |
| ● P2           | 3257  | 23.97%  | 32.57%  |

Supplement: Supplementary file 1 [file DataSheet3.ZIP › AMO1 cell cycle/2/AMO1 NC 5.pdf]

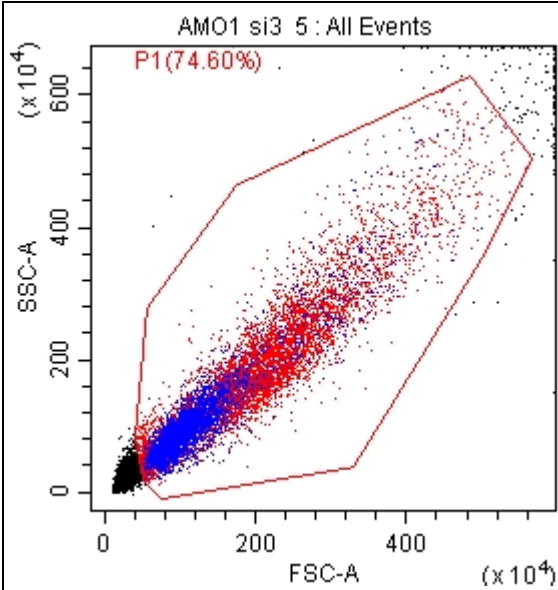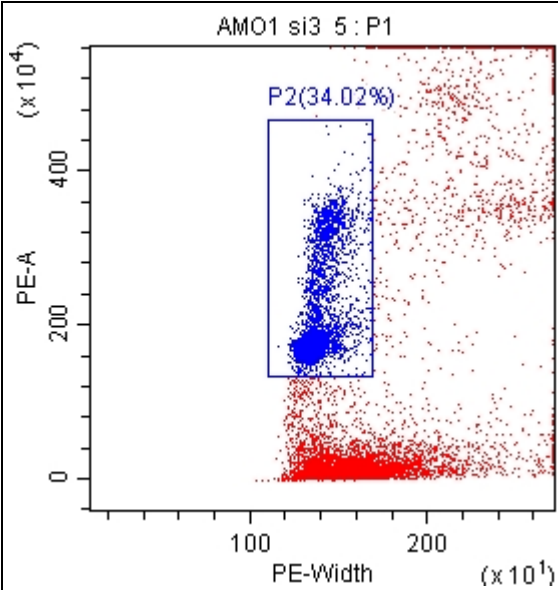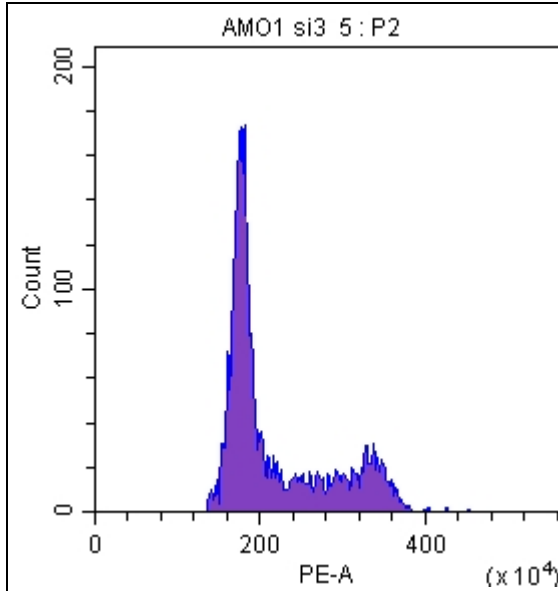

试管名称: AM01 si3 5

样本ID:

| 群体             | 颗粒数   | %总数     | %父群     |
|----------------|-------|---------|---------|
| ▼ ● All Events | 13404 | 100.00% | 100.00% |
| ▼ ● P1         | 10000 | 74.60%  | 74.60%  |
| ● P2           | 3402  | 25.38%  | 34.02%  |

Supplement: Supplementary file 1 [file DataSheet3.ZIP › AMO1 cell cycle/2/AMO1 si3 5.pdf]

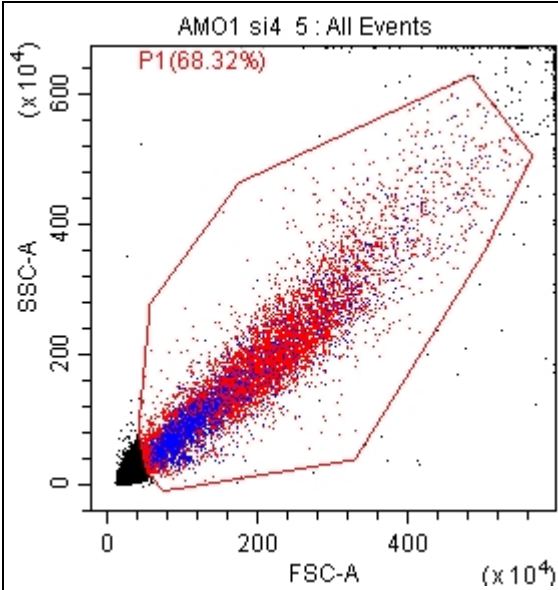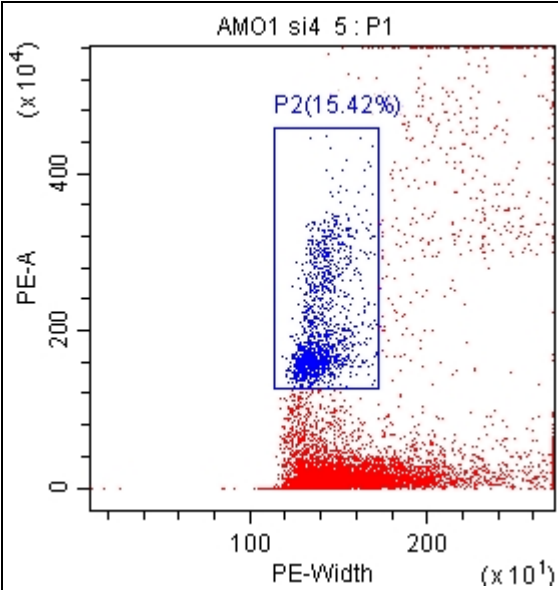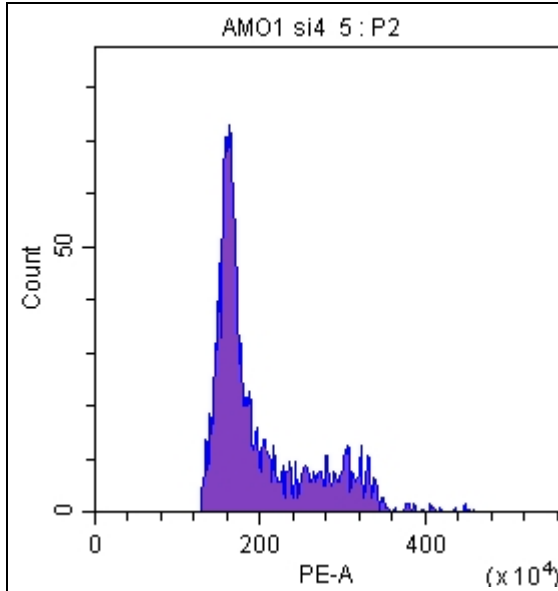

试管名称: AM01 si4 5

样本ID:

| 群体             | 颗粒数   | %总数     | %父群     |
|----------------|-------|---------|---------|
| ▼ ● All Events | 14636 | 100.00% | 100.00% |
| ▼ ● P1         | 10000 | 68.32%  | 68.32%  |
| ● P2           | 1542  | 10.54%  | 15.42%  |

Supplement: Supplementary file 1 [file DataSheet3.ZIP › AMO1 cell cycle/2/AMO1 si4 5.pdf]

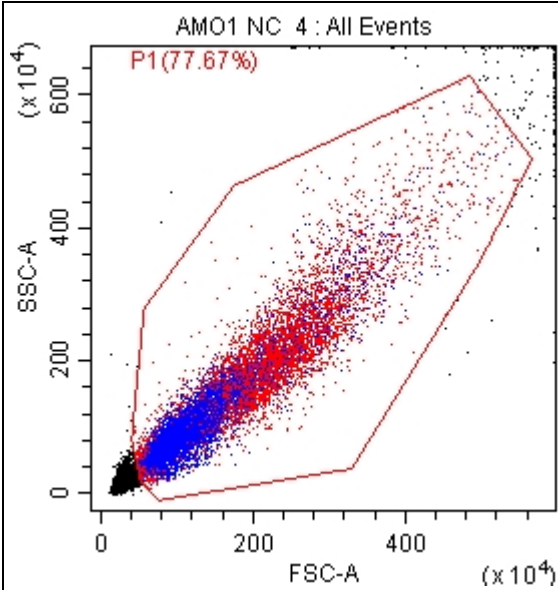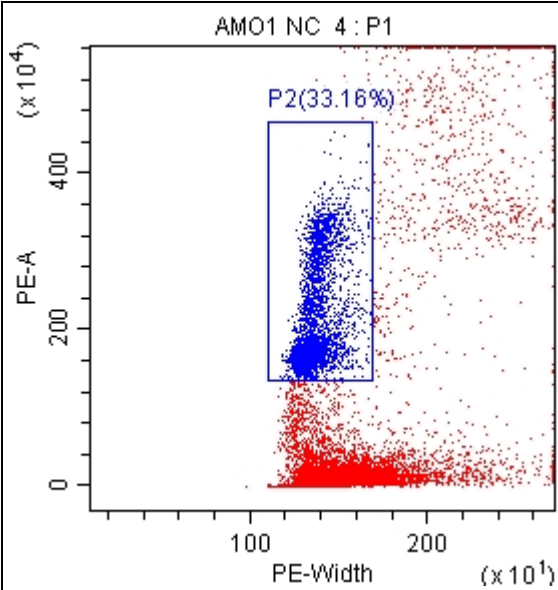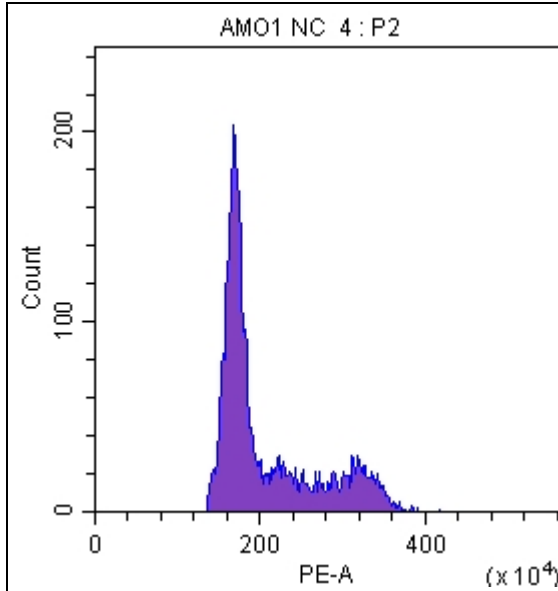

试管名称: AMO1 NC 4

样本ID:

| 群体             | 颗粒数   | %总数     | %父群     |
|----------------|-------|---------|---------|
| ▼ ● All Events | 14786 | 100.00% | 100.00% |
| ▼ ● P1         | 11484 | 77.67%  | 77.67%  |
| ● P2           | 3808  | 25.75%  | 33.16%  |

Supplement: Supplementary file 1 [file DataSheet3.ZIP › AMO1 cell cycle/3/AMO1 NC 4.pdf]

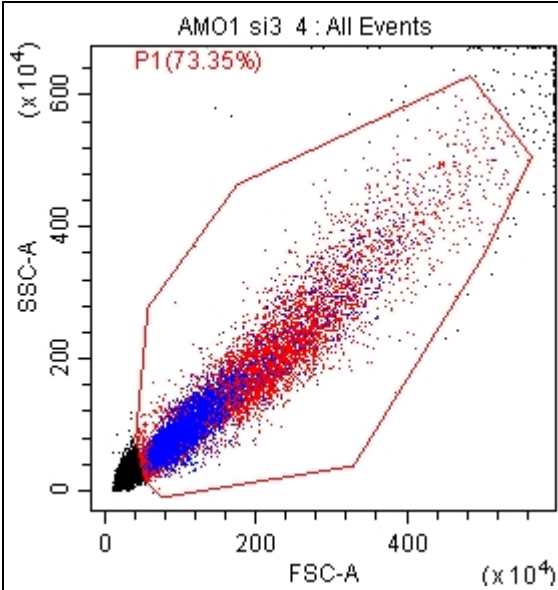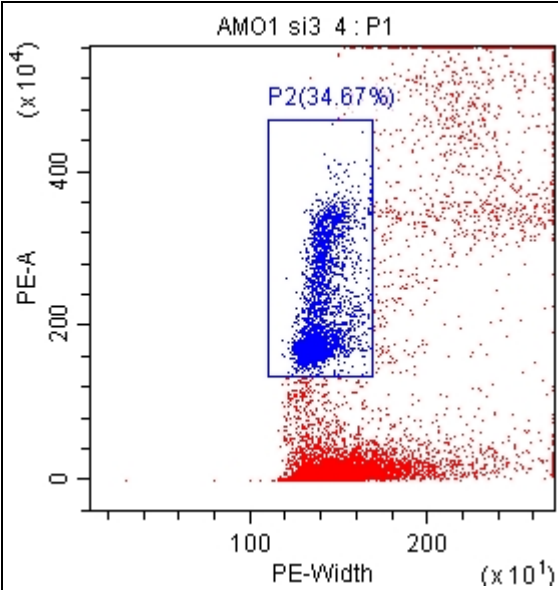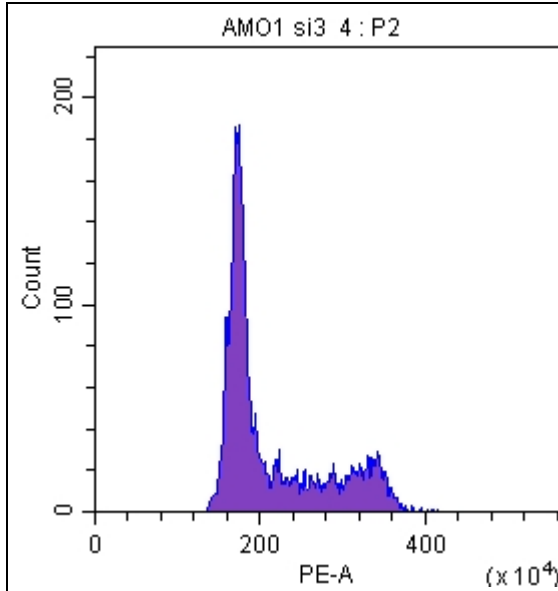

试管名称: AM01 si3 4

样本ID:

| 群体             | 颗粒数   | %总数     | %父群     |
|----------------|-------|---------|---------|
| ▼ ● All Events | 13633 | 100.00% | 100.00% |
| ▼ ● P1         | 10000 | 73.35%  | 73.35%  |
| ● P2           | 3467  | 25.43%  | 34.67%  |

Supplement: Supplementary file 1 [file DataSheet3.ZIP › AMO1 cell cycle/3/AMO1 si3 4.pdf]

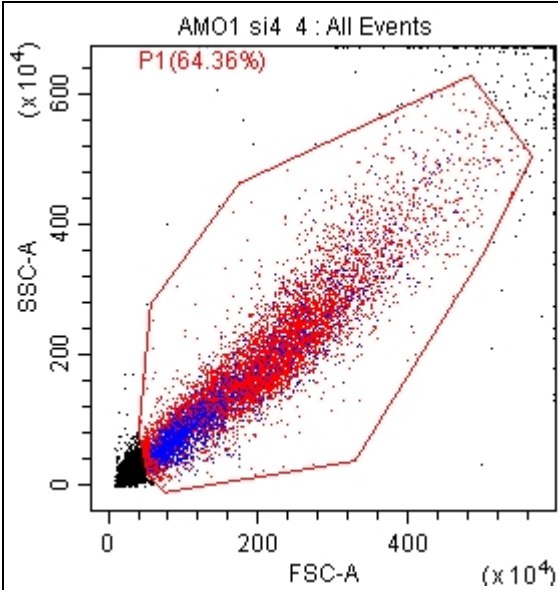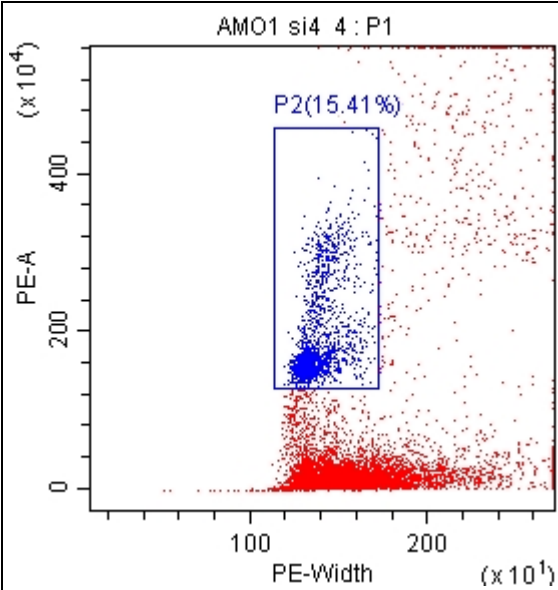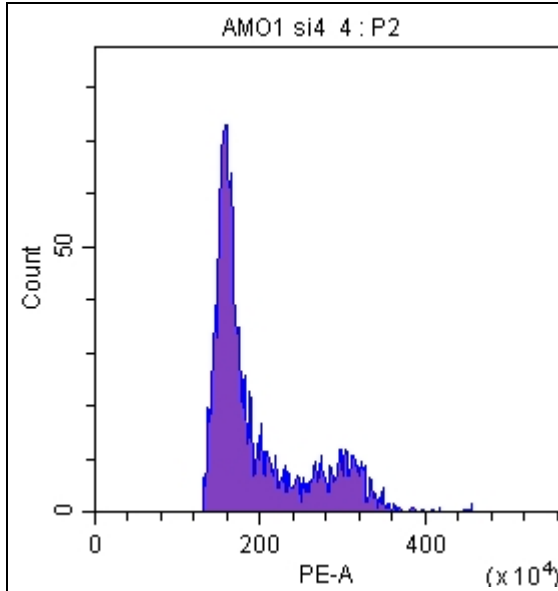

试管名称: AM01 si4 4

样本ID:

| 群体             | 颗粒数   | %总数     | %父群     |
|----------------|-------|---------|---------|
| ▼ ● All Events | 15538 | 100.00% | 100.00% |
| ▼ ● P1         | 10000 | 64.36%  | 64.36%  |
| ● P2           | 1541  | 9.92%   | 15.41%  |

Supplement: Supplementary file 1 [file DataSheet3.ZIP › AMO1 cell cycle/3/AMO1 si4 4.pdf]

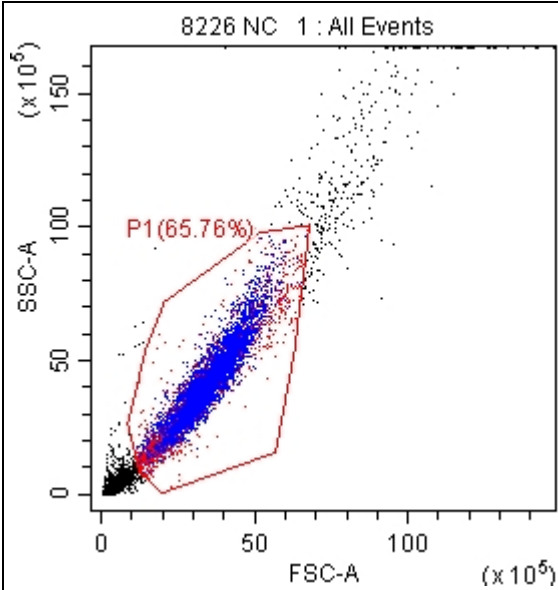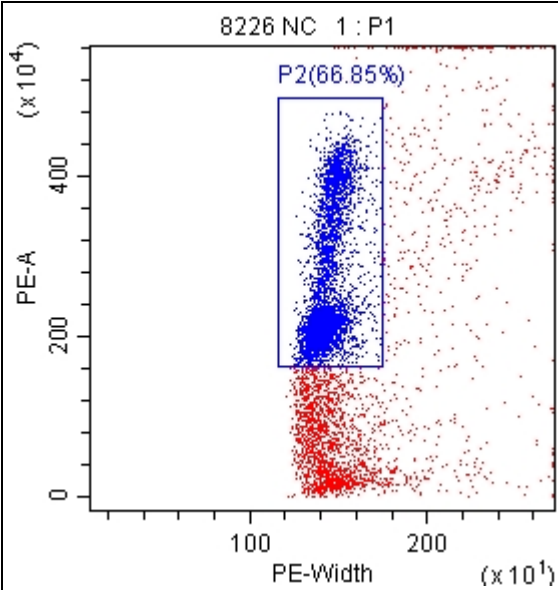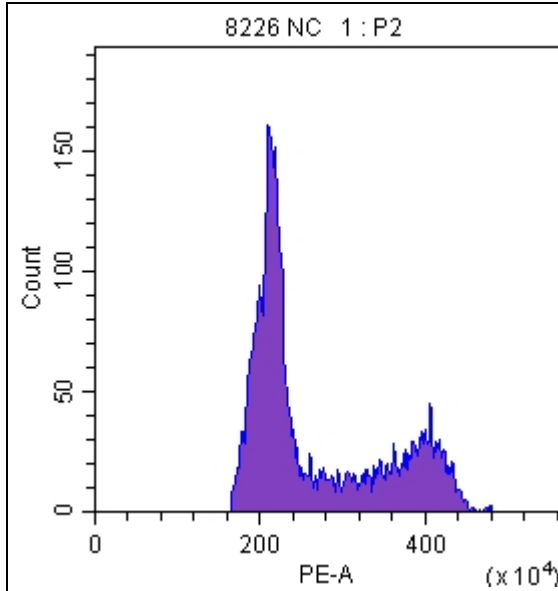

试管名称: 8226 NC 1

样本ID:

| 群体             | 颗粒数   | %总数     | %父群     |
|----------------|-------|---------|---------|
| ▼ ● All Events | 10000 | 100.00% | 100.00% |
| ▼ ● P1         | 6576  | 65.76%  | 65.76%  |
| ● P2           | 4396  | 43.96%  | 66.85%  |

Supplement: Supplementary file 2 [file DataSheet4.ZIP › 8226 cell cycle/1/8226 NC 1.pdf]

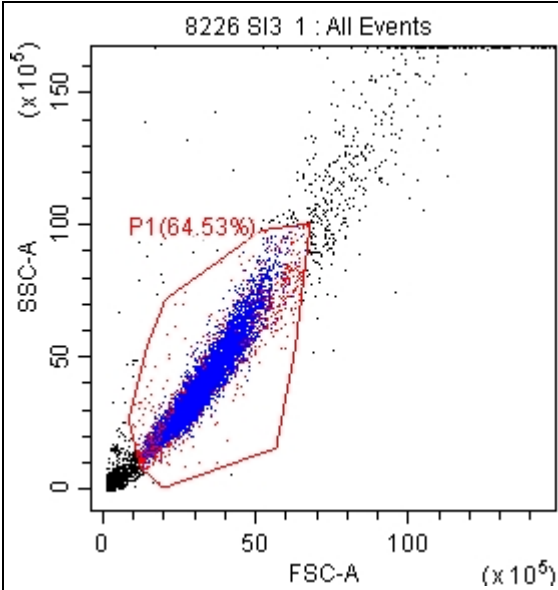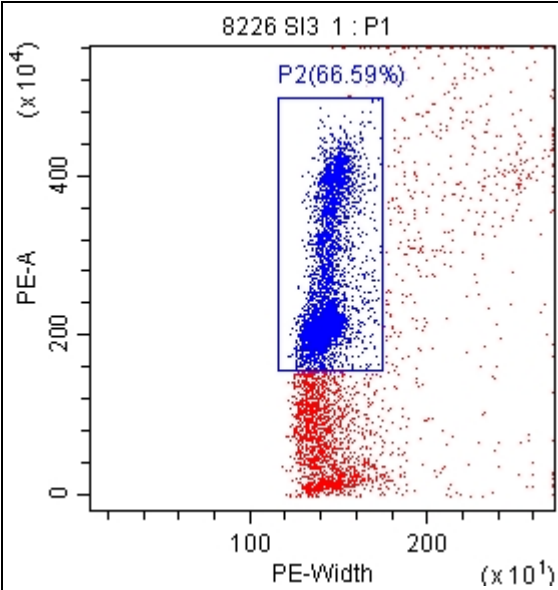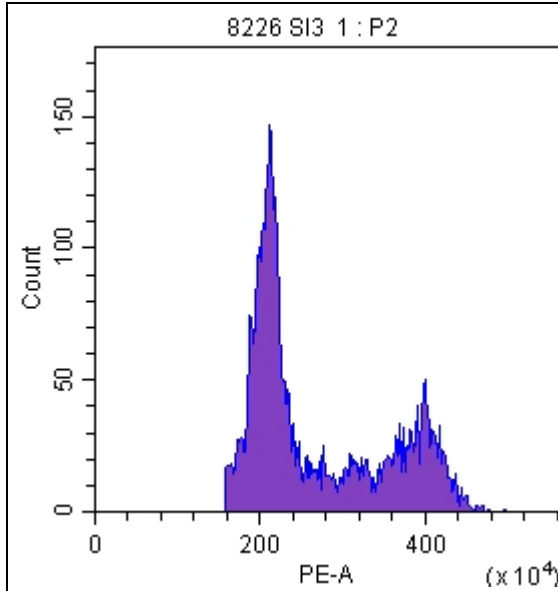

试管名称: 8226 SI3 1

样本ID:

| 群体             | 颗粒数   | %总数     | %父群     |
|----------------|-------|---------|---------|
| ▼ ● All Events | 10000 | 100.00% | 100.00% |
| ▼ ● P1         | 6453  | 64.53%  | 64.53%  |
| ● P2           | 4297  | 42.97%  | 66.59%  |

Supplement: Supplementary file 2 [file DataSheet4.ZIP › 8226 cell cycle/1/8226 SI3 1.pdf]

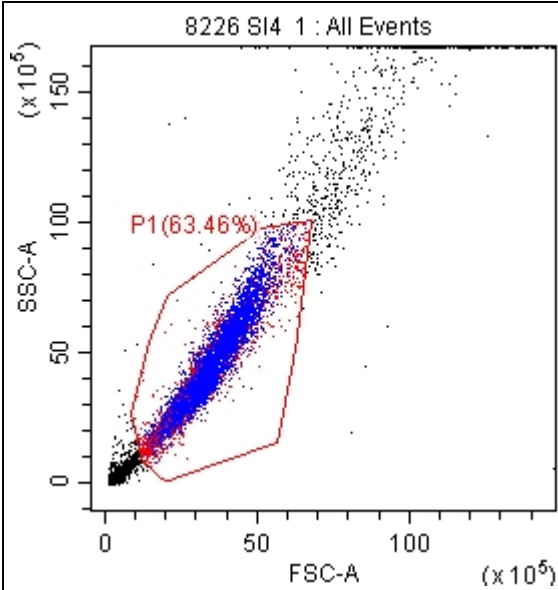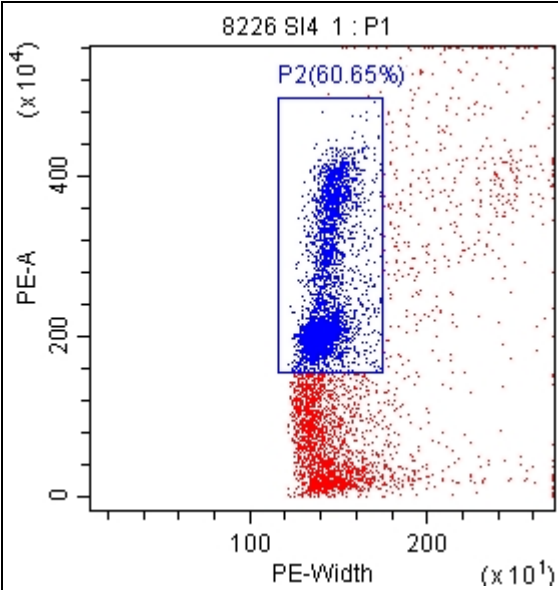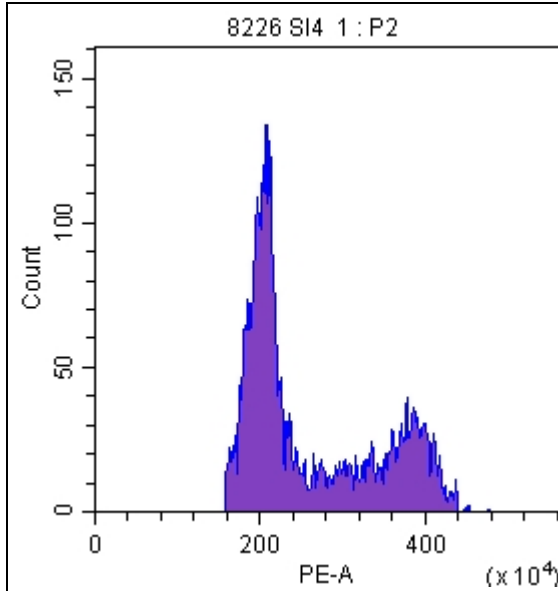

试管名称: 8226 SI4 1

样本ID:

| 群体             | 颗粒数   | %总数     | %父群     |
|----------------|-------|---------|---------|
| ▼ ● All Events | 10000 | 100.00% | 100.00% |
| ▼ ● P1         | 6346  | 63.46%  | 63.46%  |
| ● P2           | 3849  | 38.49%  | 60.65%  |

Supplement: Supplementary file 2 [file DataSheet4.ZIP › 8226 cell cycle/1/8226 SI4 1.pdf]

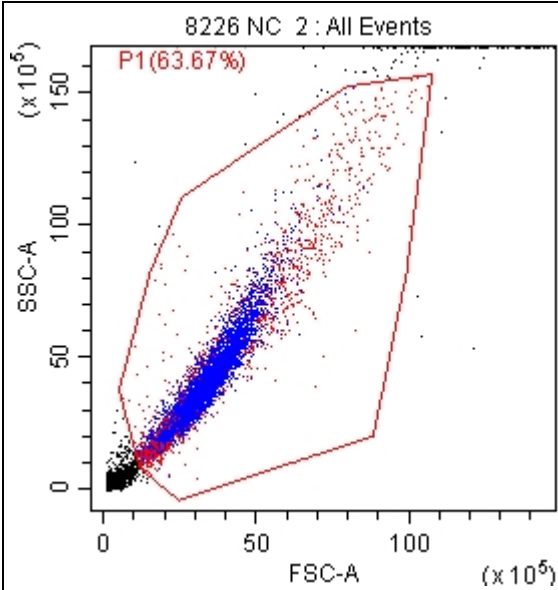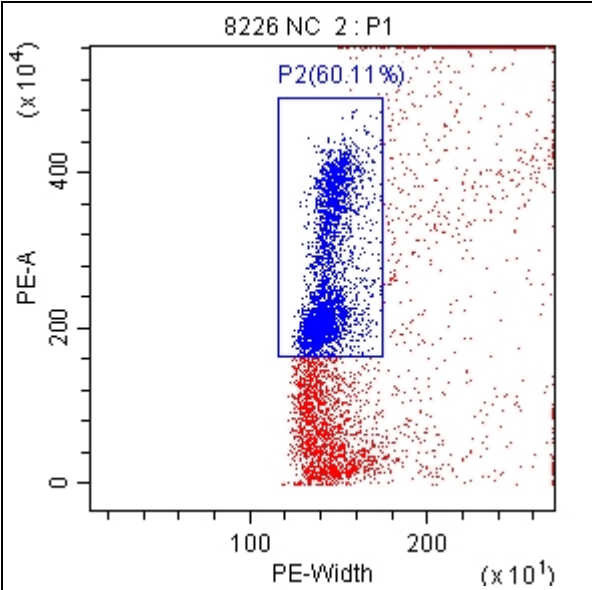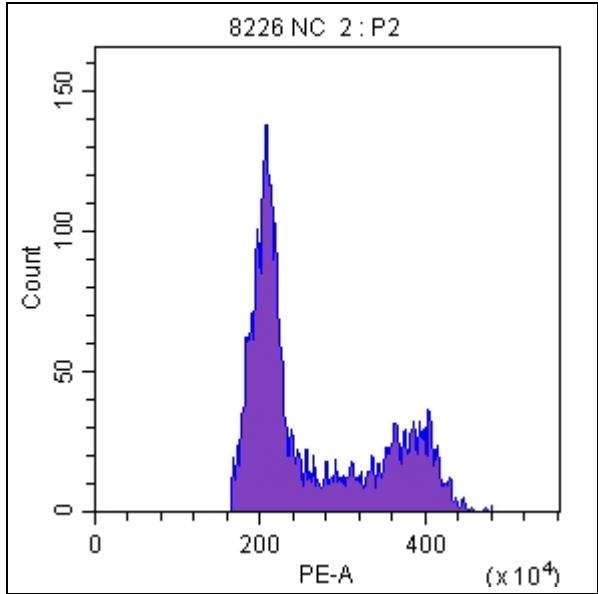

试管名称: 8226 NC 2

样本ID:

| 群体             | 颗粒数   | %总数     | %父群     |
|----------------|-------|---------|---------|
| ▼ ● All Events | 10000 | 100.00% | 100.00% |
| ▼ ● P1         | 6367  | 63.67%  | 63.67%  |
| ● P2           | 3827  | 38.27%  | 60.11%  |

Supplement: Supplementary file 2 [file DataSheet4.ZIP › 8226 cell cycle/2/8226 NC 2.pdf]

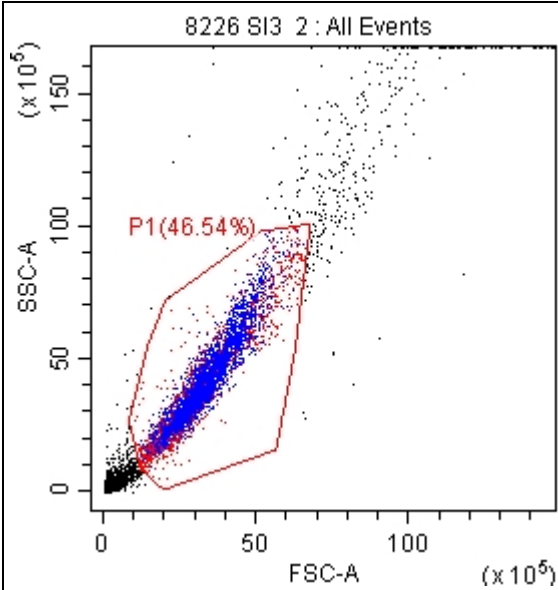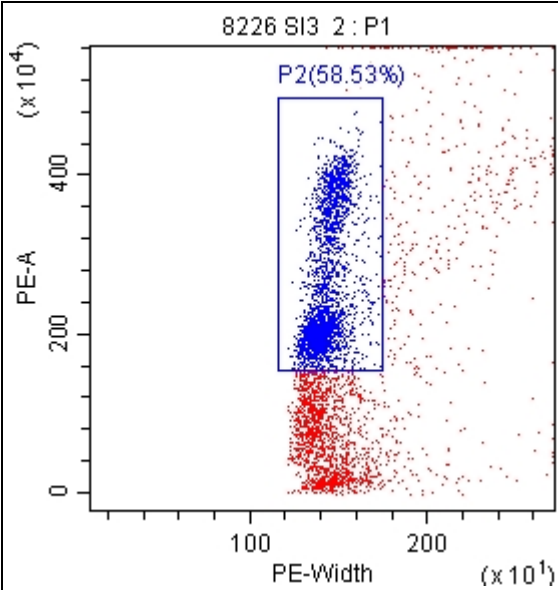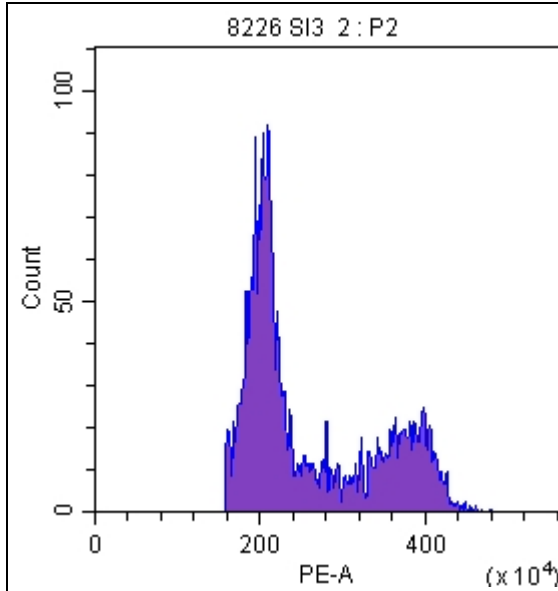

试管名称: 8226 SI3 2

样本ID:

| 群体            | 颗粒数   | %总数     | %父群     |
|---------------|-------|---------|---------|
| ▼  All Events | 10000 | 100.00% | 100.00% |
| ▼  P1         | 4654  | 46.54%  | 46.54%  |
| P2            | 2724  | 27.24%  | 58.53%  |

Supplement: Supplementary file 2 [file DataSheet4.ZIP › 8226 cell cycle/2/8226 SI3 2.pdf]

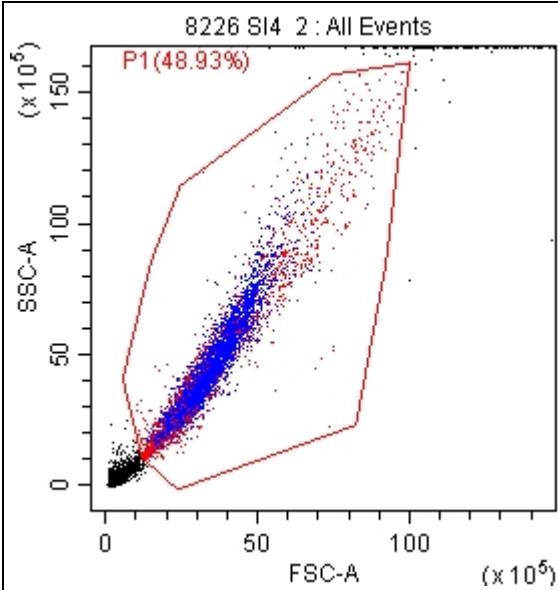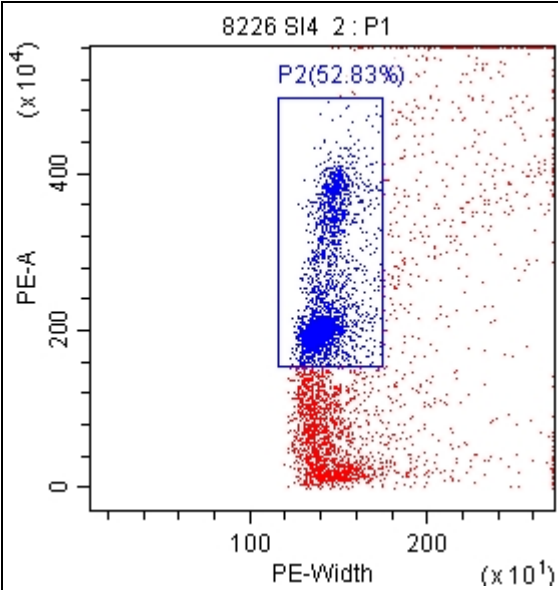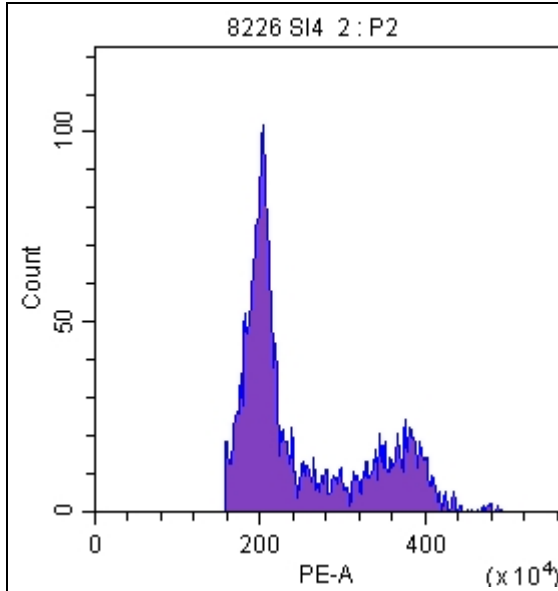

试管名称: 8226 SI4 2

样本ID:

| 群体             | 颗粒数   | %总数     | %父群     |
|----------------|-------|---------|---------|
| ▼ ● All Events | 10000 | 100.00% | 100.00% |
| ▼ ● P1         | 4893  | 48.93%  | 48.93%  |
| ● P2           | 2585  | 25.85%  | 52.83%  |

Supplement: Supplementary file 2 [file DataSheet4.ZIP › 8226 cell cycle/2/8226 SI4 2.pdf]

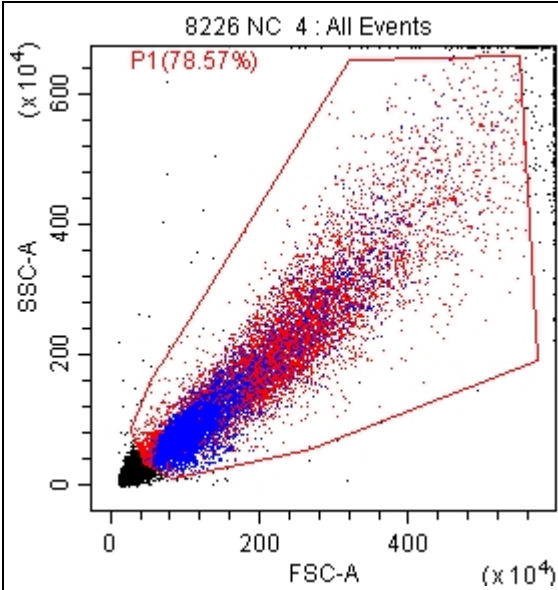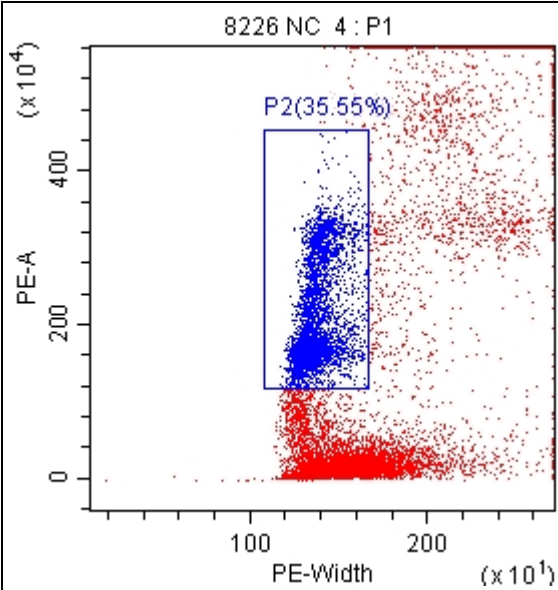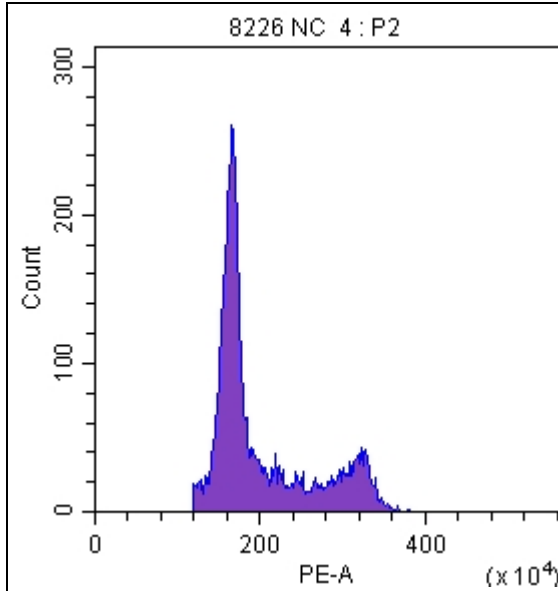

试管名称: 8226 NC 4

样本ID:

| 群体             | 颗粒数   | %总数     | %父群     |
|----------------|-------|---------|---------|
| ▼ ● All Events | 17669 | 100.00% | 100.00% |
| ▼ ● P1         | 13882 | 78.57%  | 78.57%  |
| ● P2           | 4935  | 27.93%  | 35.55%  |

Supplement: Supplementary file 2 [file DataSheet4.ZIP › 8226 cell cycle/3/8226 NC 4.pdf]

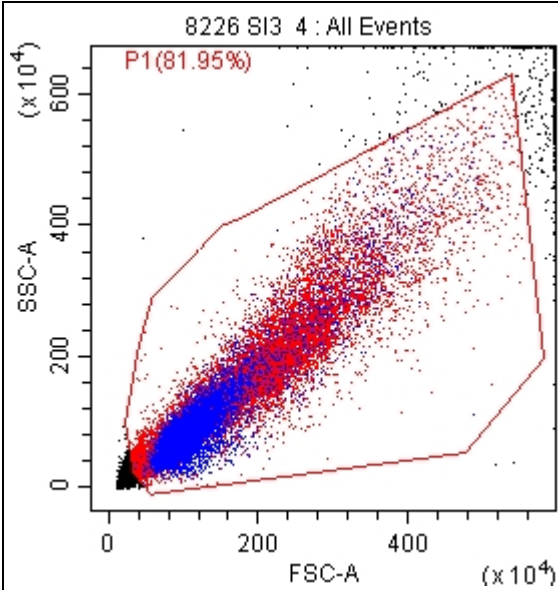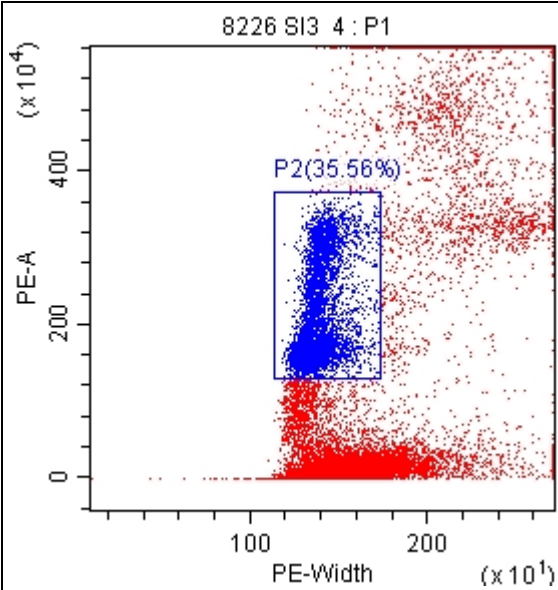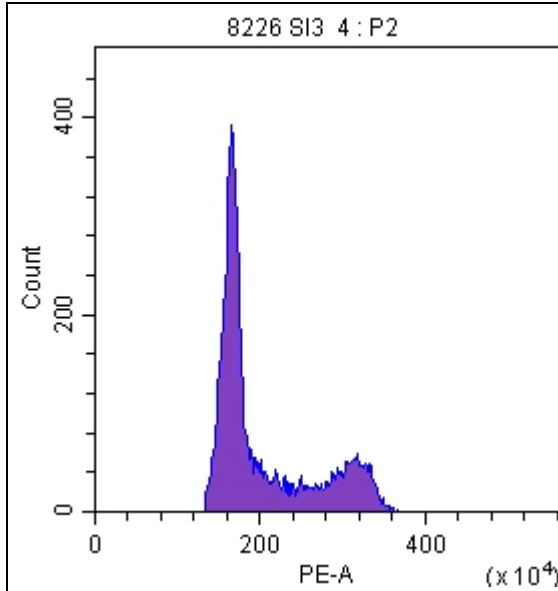

试管名称: 8226 SI3 4

样本ID:

| 群体             | 颗粒数   | %总数     | %父群     |
|----------------|-------|---------|---------|
| ▼ ● All Events | 23897 | 100.00% | 100.00% |
| ▼ ● P1         | 19584 | 81.95%  | 81.95%  |
| ● P2           | 6964  | 29.14%  | 35.56%  |

Supplement: Supplementary file 2 [file DataSheet4.ZIP › 8226 cell cycle/3/8226 SI3 4.pdf]

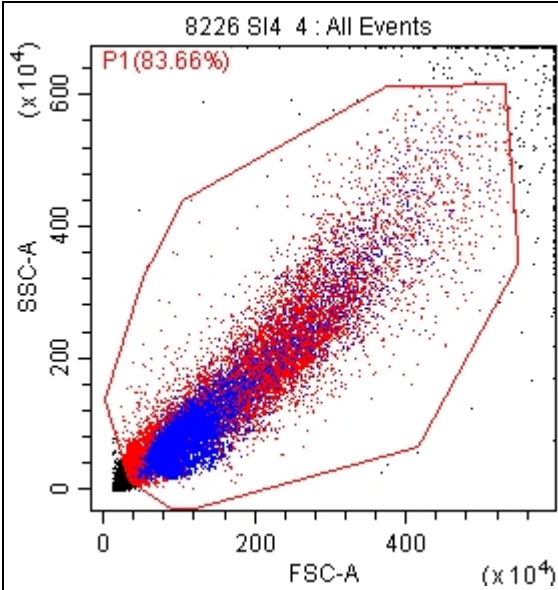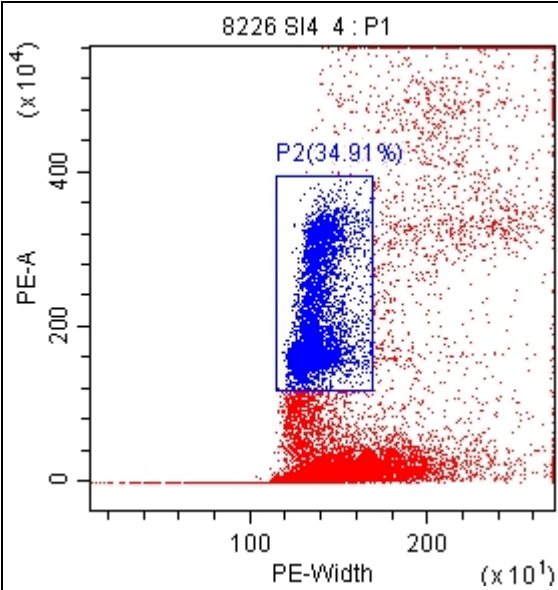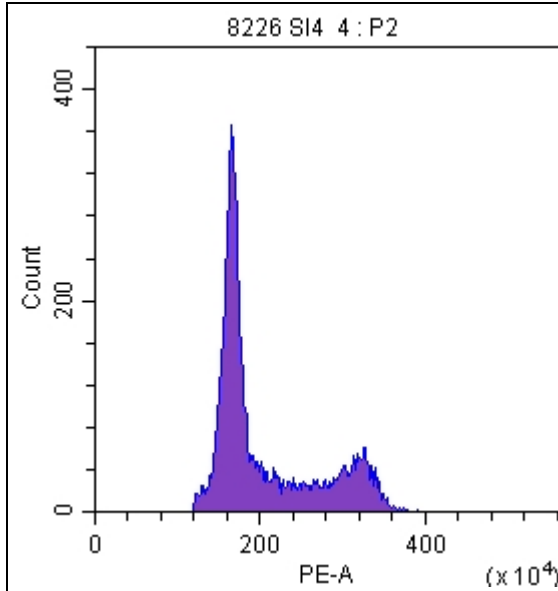

试管名称: 8226 SI4 4

样本ID:

| 群体             | 颗粒数   | %总数     | %父群     |
|----------------|-------|---------|---------|
| ▼ ● All Events | 22730 | 100.00% | 100.00% |
| ▼ ● P1         | 19017 | 83.66%  | 83.66%  |
| ● P2           | 6638  | 29.20%  | 34.91%  |

Supplement: Supplementary file 2 [file DataSheet4.ZIP › 8226 cell cycle/3/8226 SI4 4.pdf]

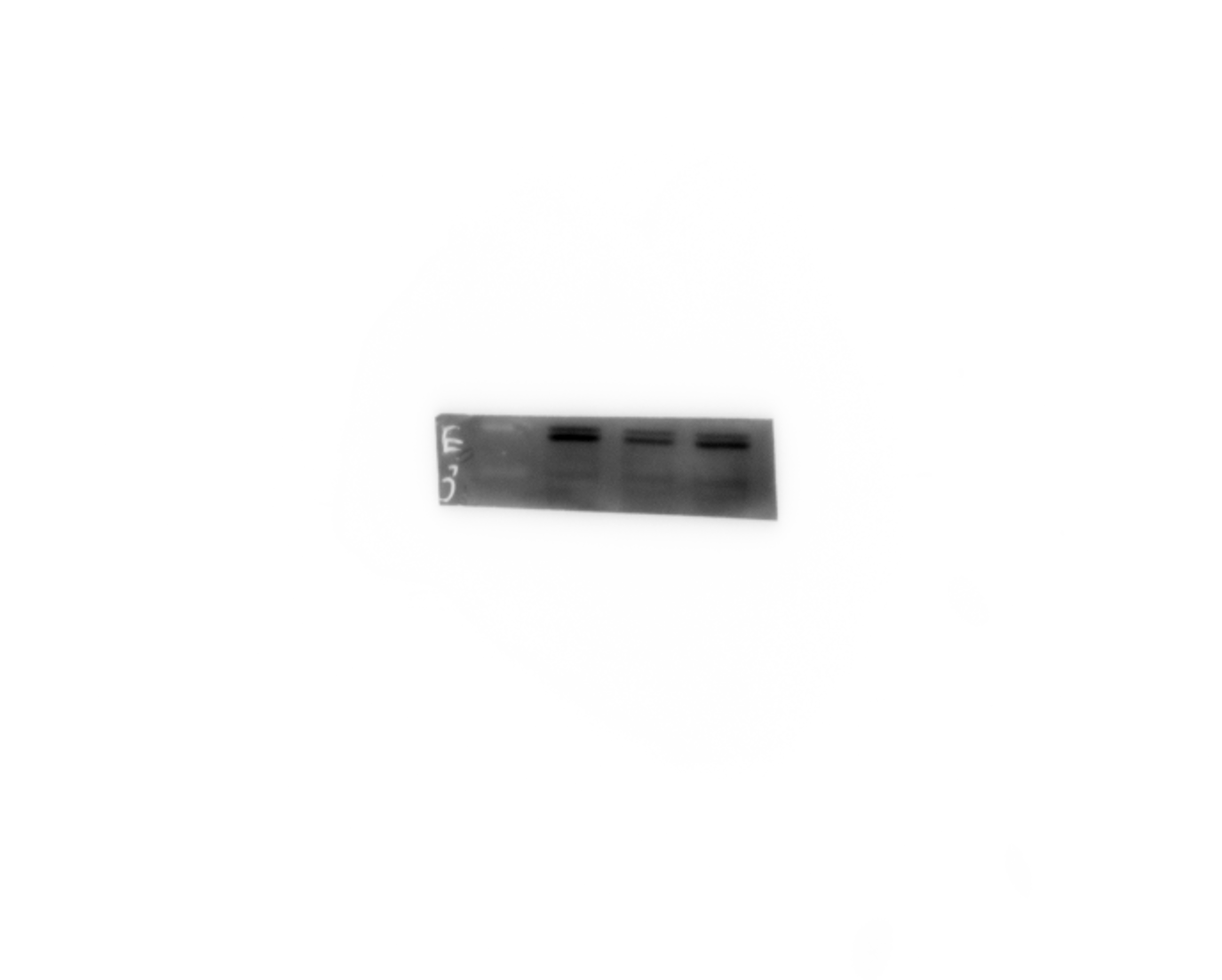

Supplement: Supplementary file 3 [file DataSheet1.ZIP › WB/8226/10 p-erk12/10.10/E.Tif]

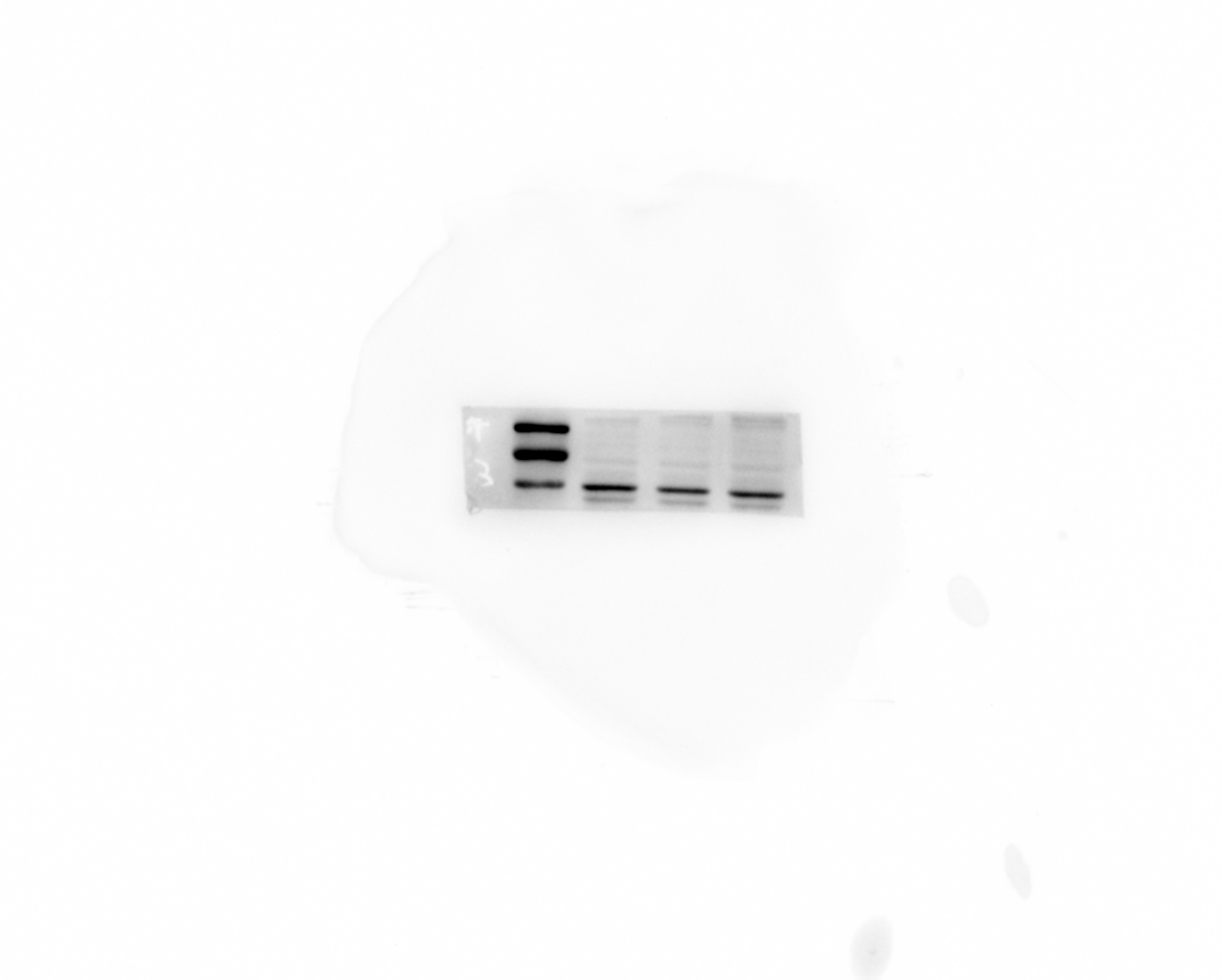

Supplement: Supplementary file 3 [file DataSheet1.ZIP › WB/8226/10 p-erk12/10.10/a┬-Tubulin.Tif]

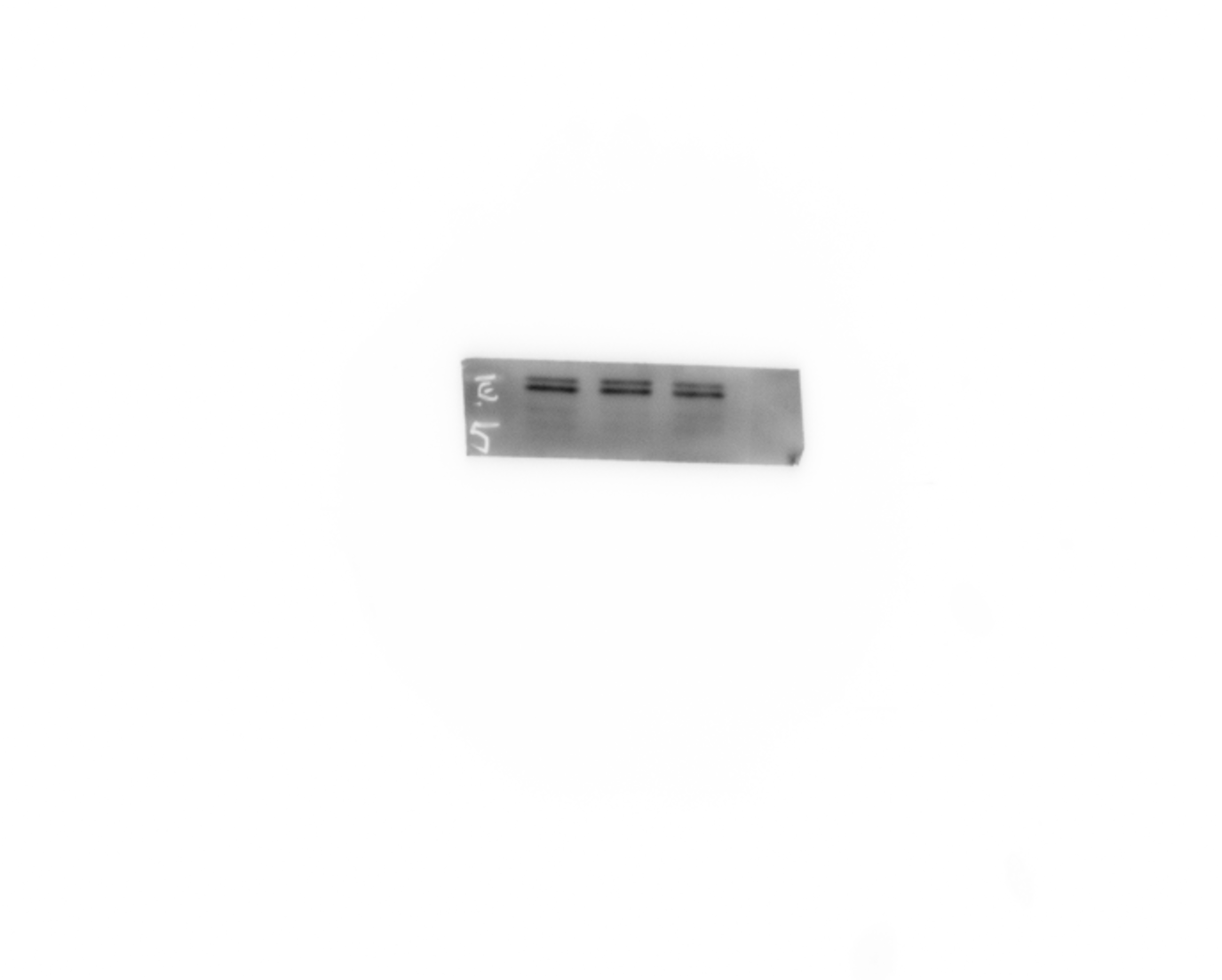

Supplement: Supplementary file 3 [file DataSheet1.ZIP › WB/8226/10 p-erk12/10.11/8 E.Tif]

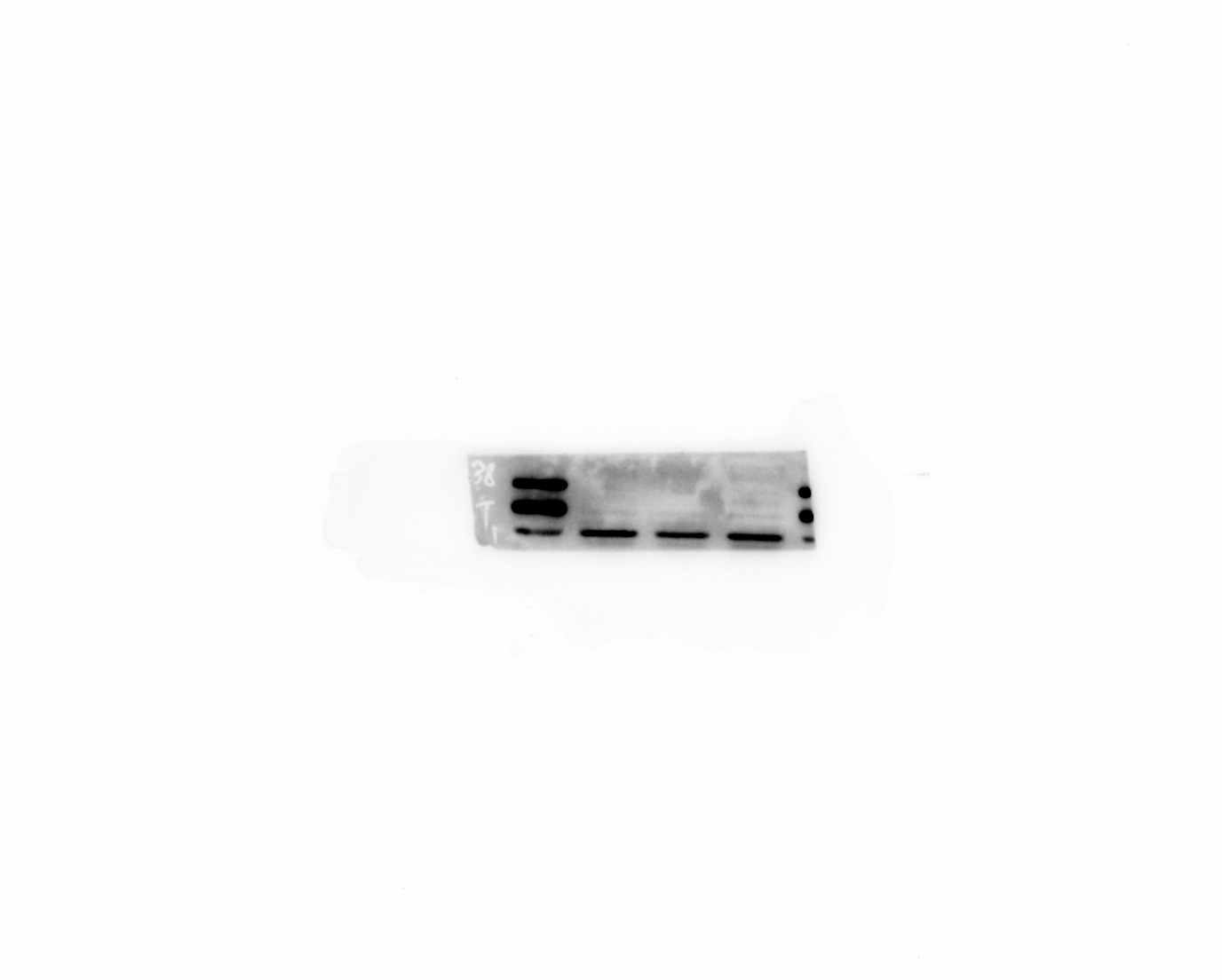

Supplement: Supplementary file 3 [file DataSheet1.ZIP › WB/8226/10 p-erk12/10.11/a┬-Tubulin.Tif]

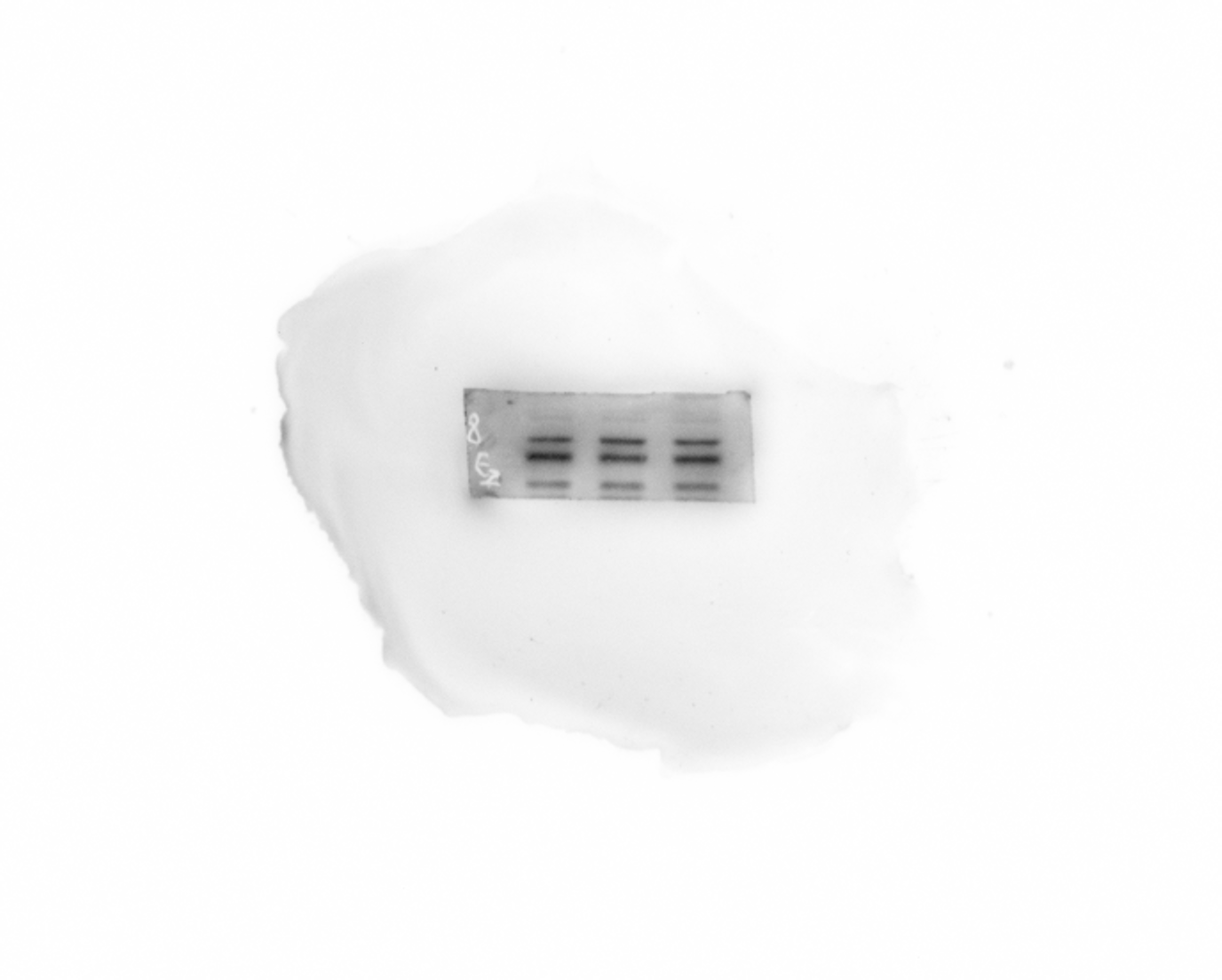

Supplement: Supplementary file 3 [file DataSheet1.ZIP › WB/8226/10 p-erk12/10.9/8E 2.Tif]

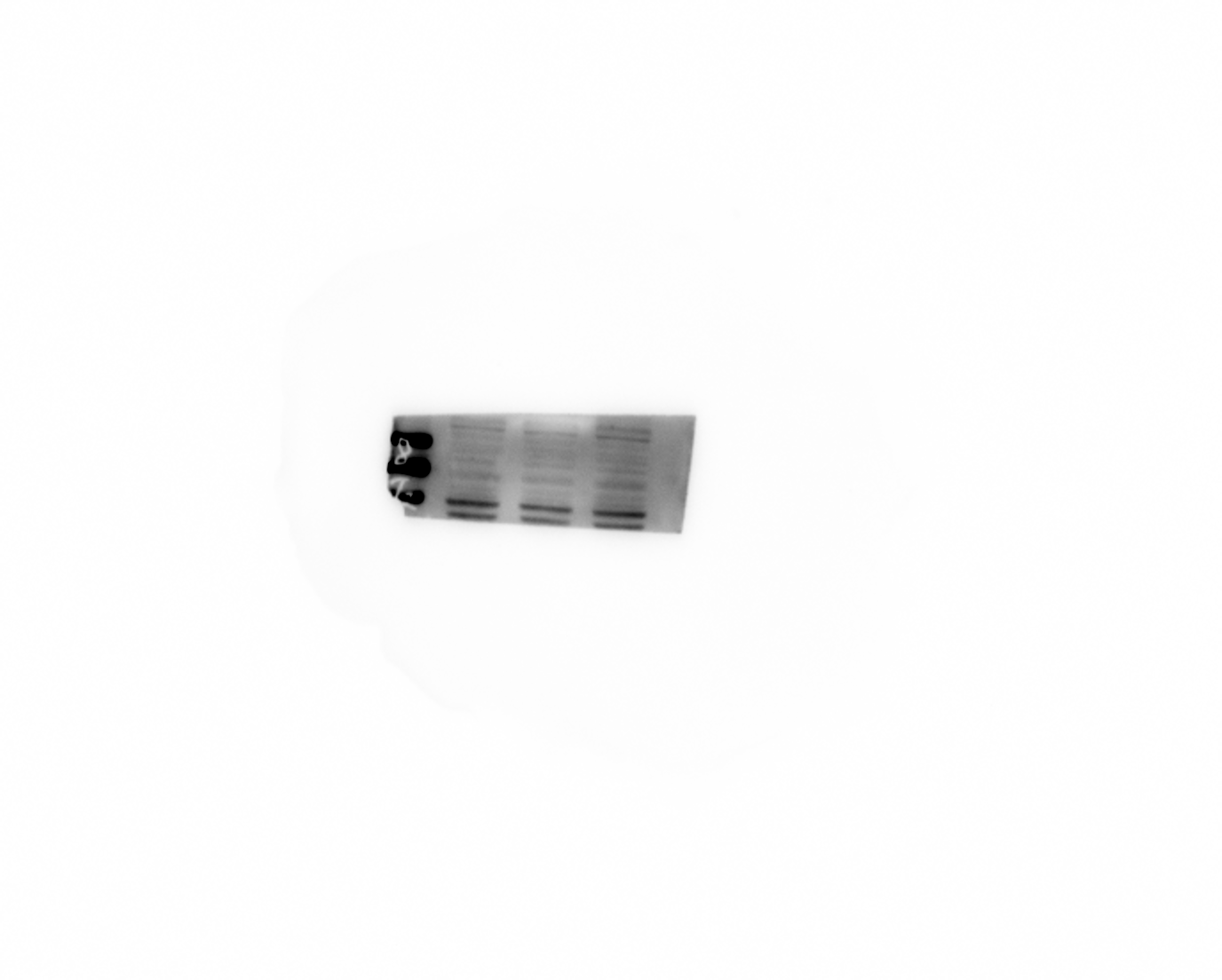

Supplement: Supplementary file 3 [file DataSheet1.ZIP › WB/8226/10 p-erk12/10.9/8a┬-Tubulin.Tif]

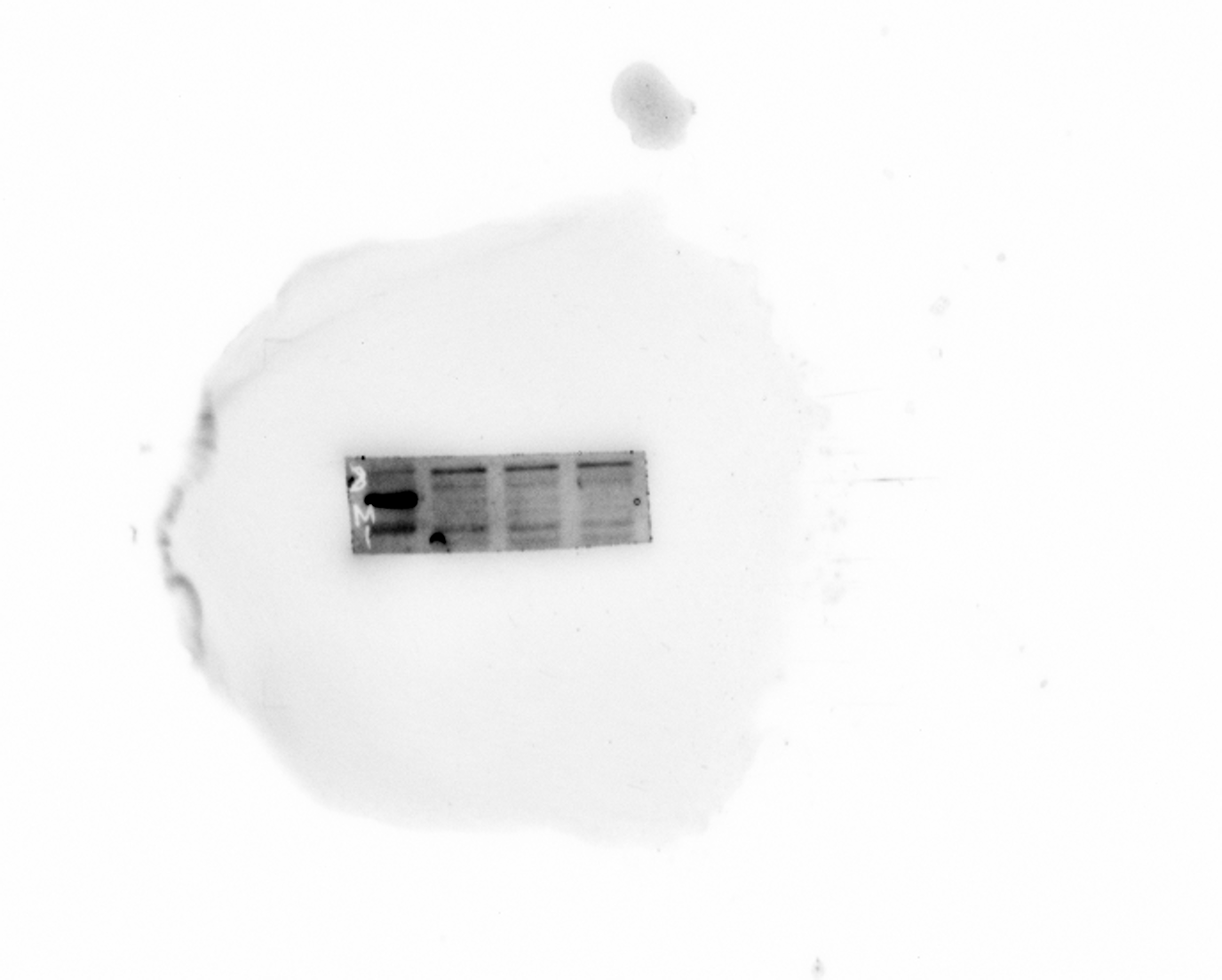

Supplement: Supplementary file 3 [file DataSheet1.ZIP › WB/8226/11 p-msk1/10.4/8 PMSk1.Tif]

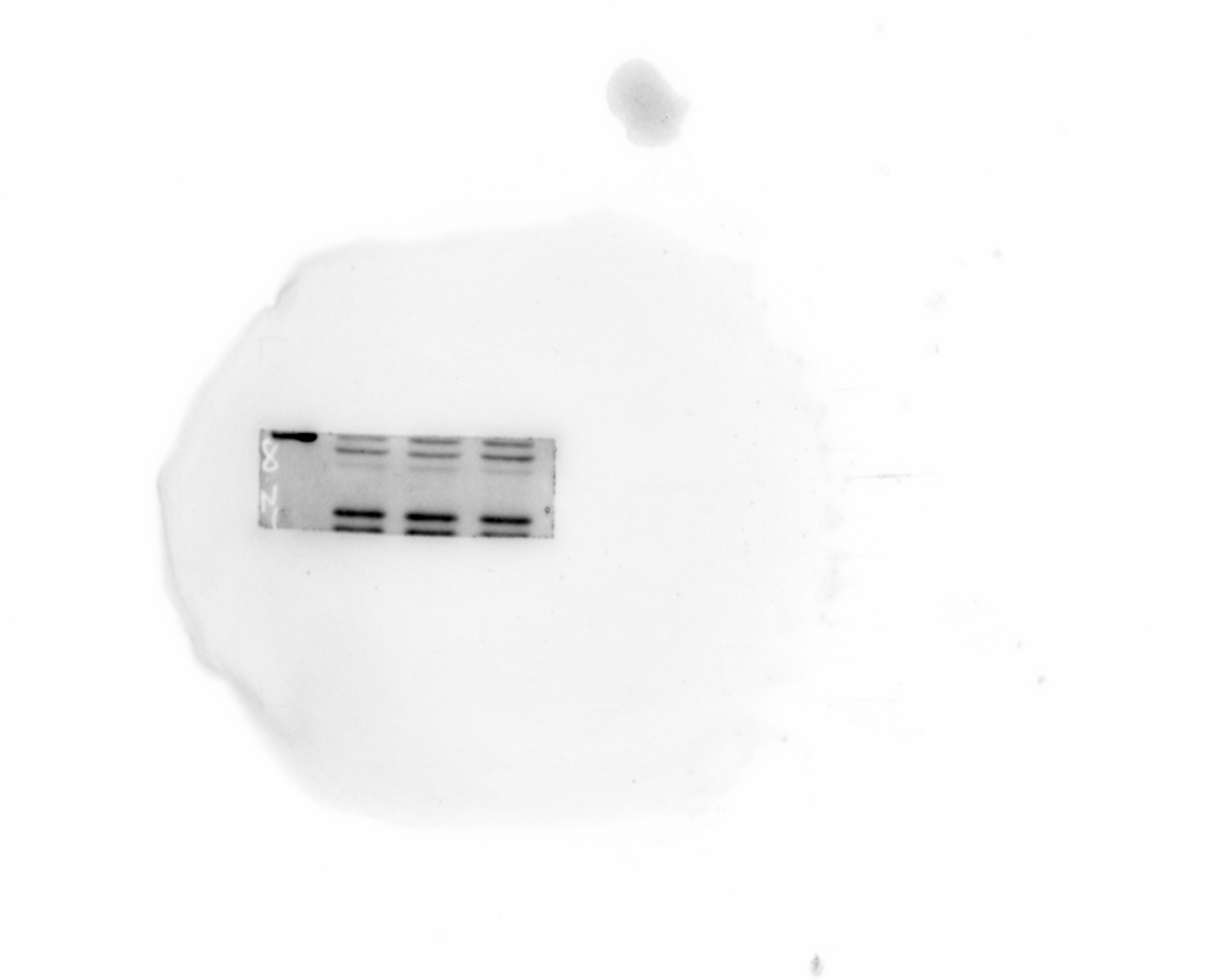

Supplement: Supplementary file 3 [file DataSheet1.ZIP › WB/8226/11 p-msk1/10.4/8a┬-actin.Tif]

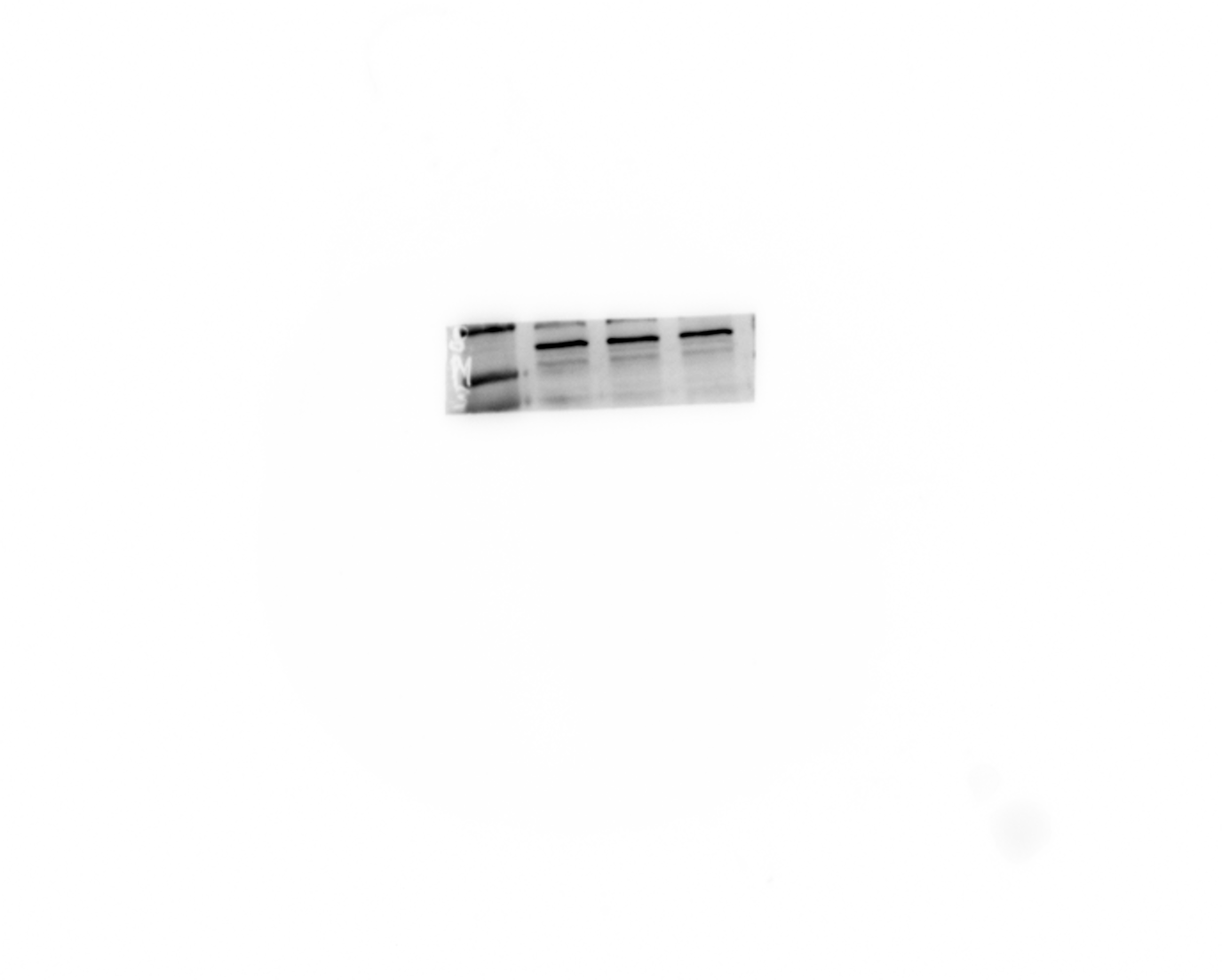

Supplement: Supplementary file 3 [file DataSheet1.ZIP › WB/8226/11 p-msk1/10.5/8 pmsk.Tif]

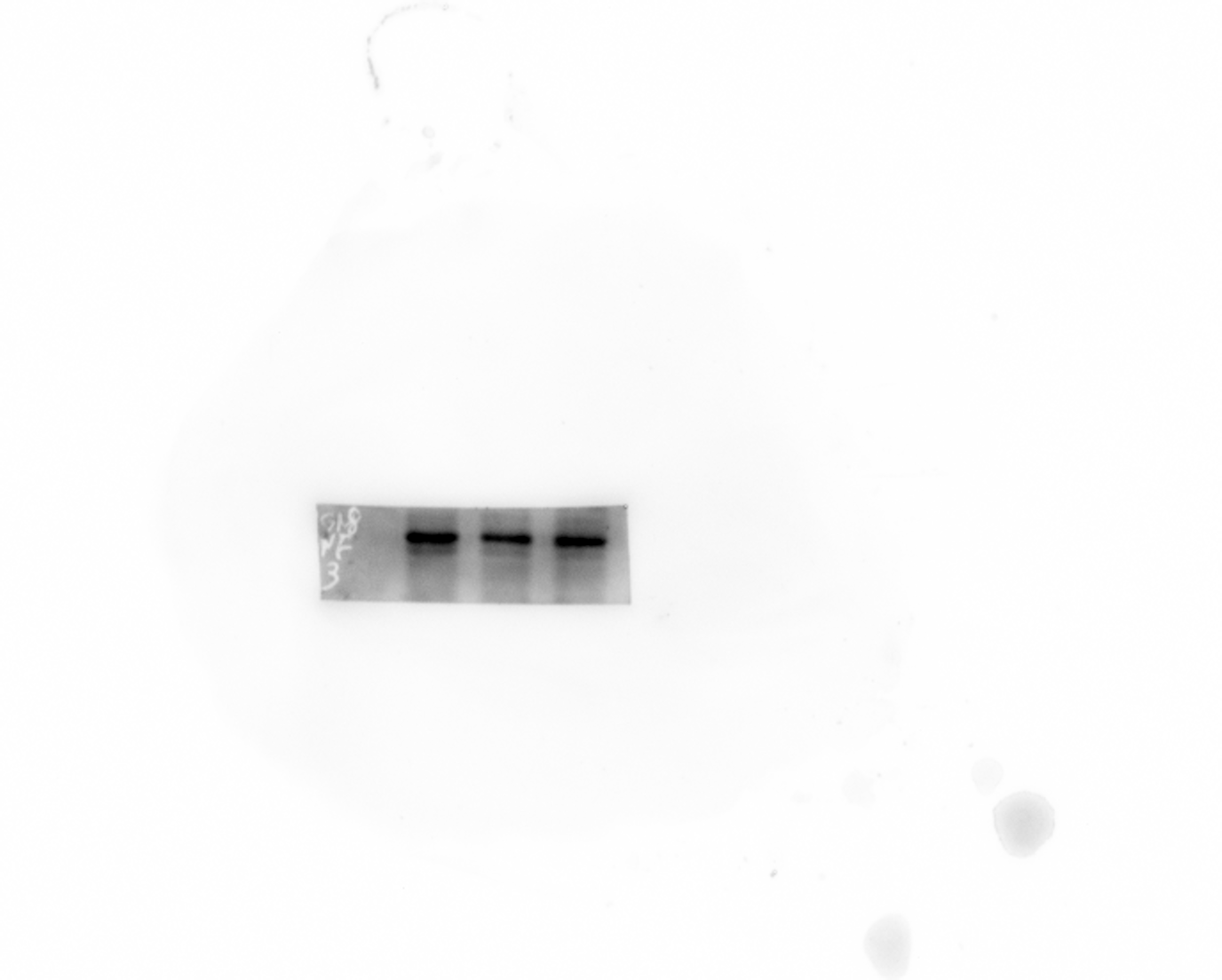

Supplement: Supplementary file 3 [file DataSheet1.ZIP › WB/8226/11 p-msk1/10.5/8 a┬-actin.Tif]

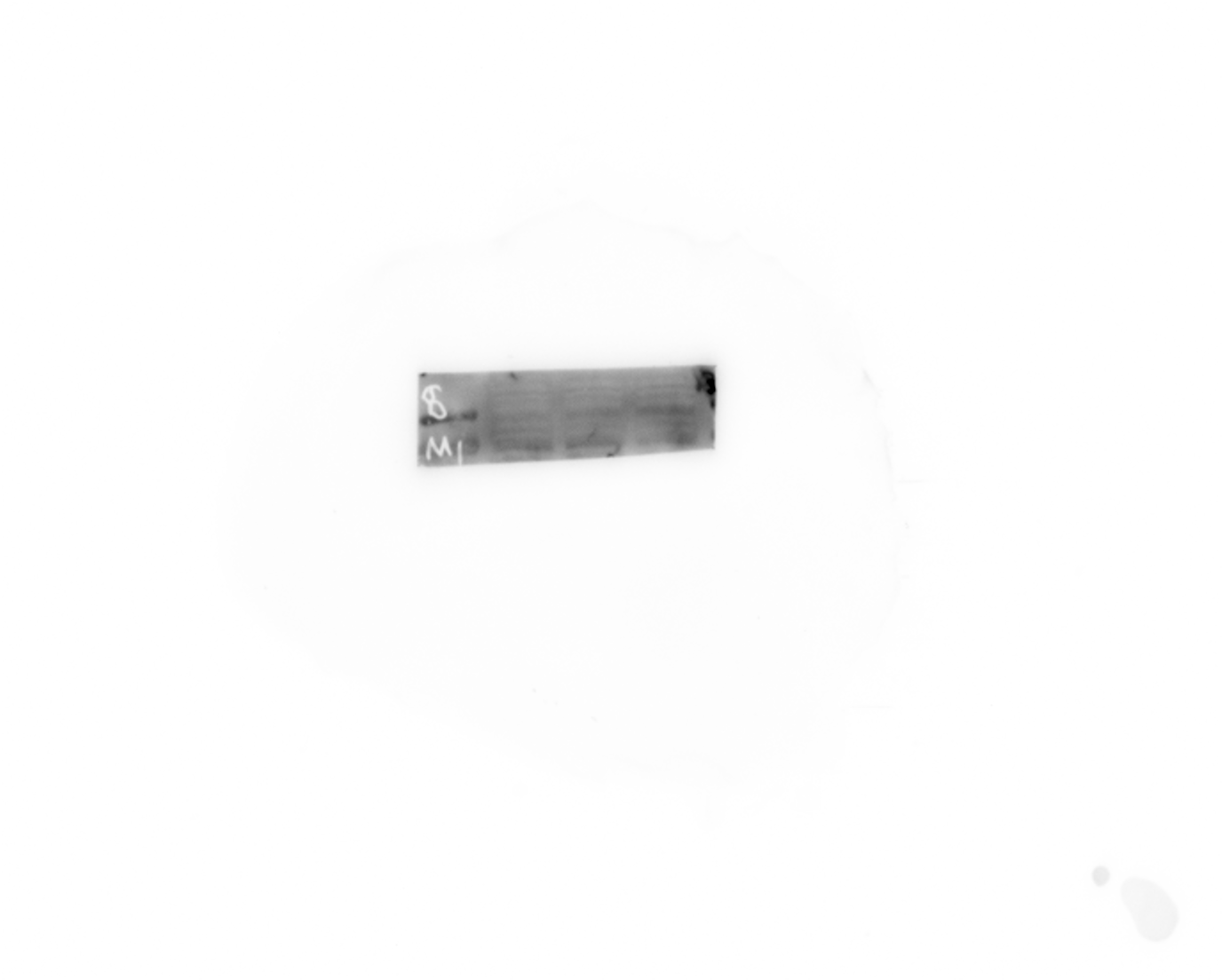

Supplement: Supplementary file 3 [file DataSheet1.ZIP › WB/8226/11 p-msk1/10.7/8M1.Tif]

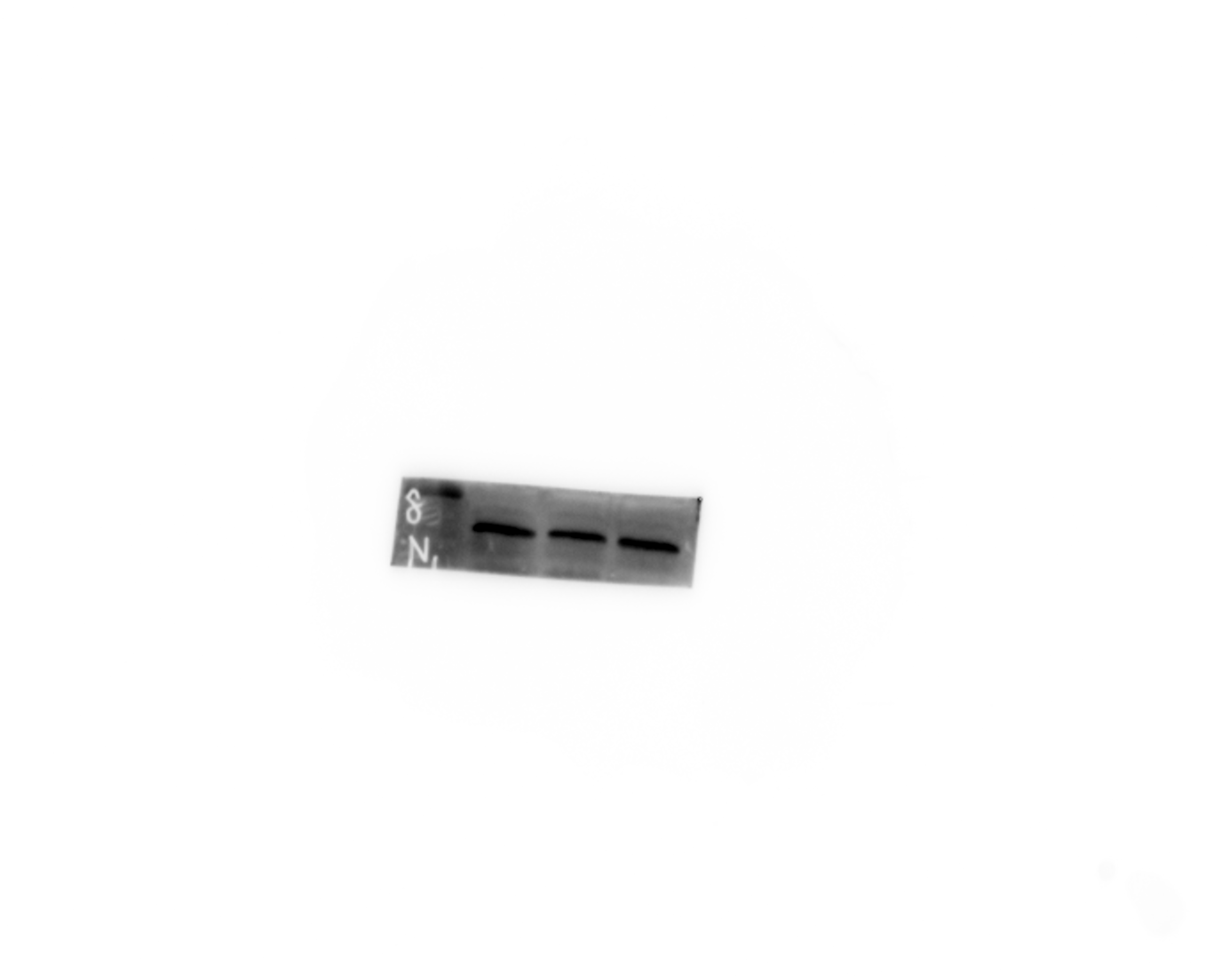

Supplement: Supplementary file 3 [file DataSheet1.ZIP › WB/8226/11 p-msk1/10.7/8a┬-actin.Tif]

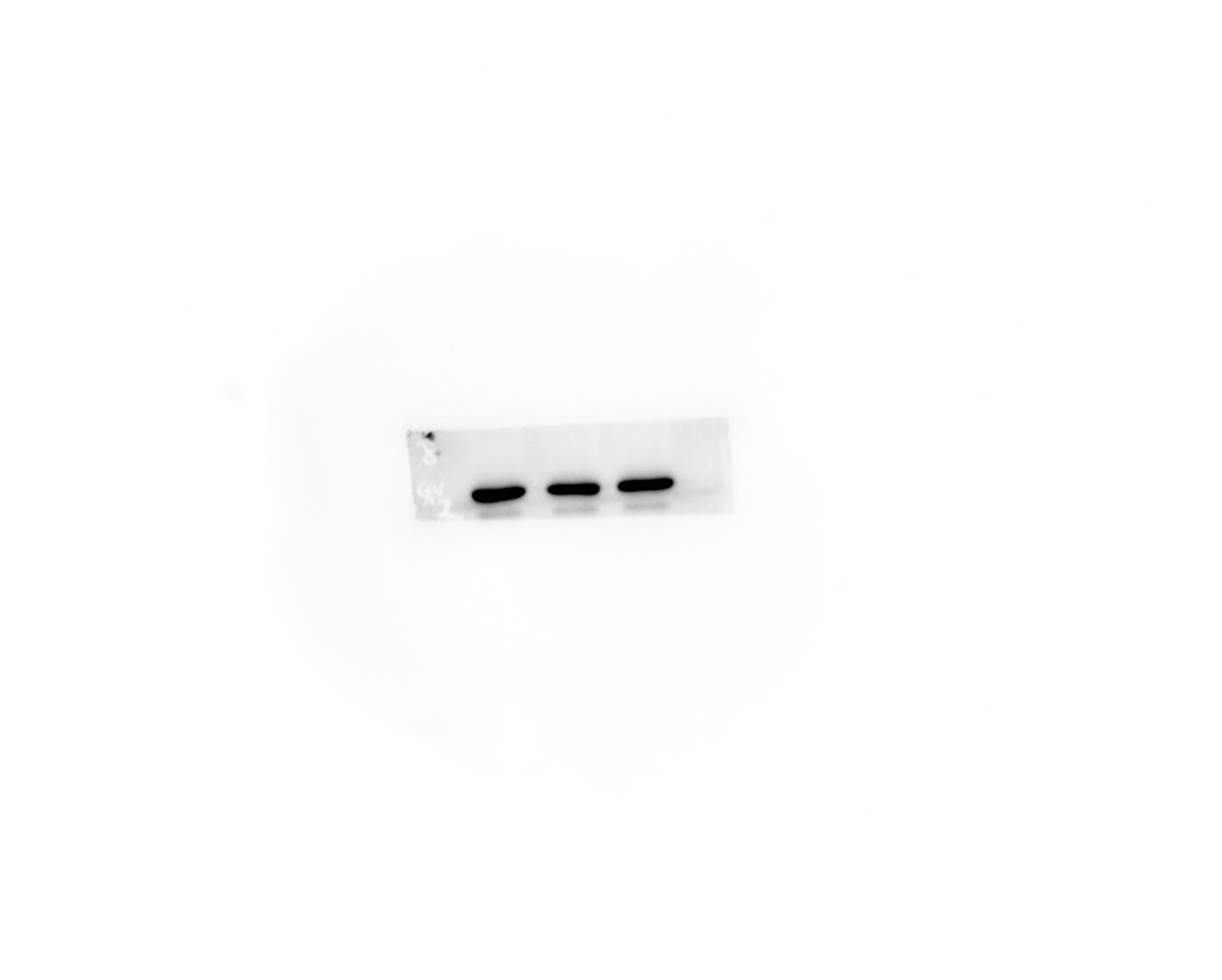

Supplement: Supplementary file 3 [file DataSheet1.ZIP › WB/8226/12p-nfkb/10.2/8GAPDH.Tif]

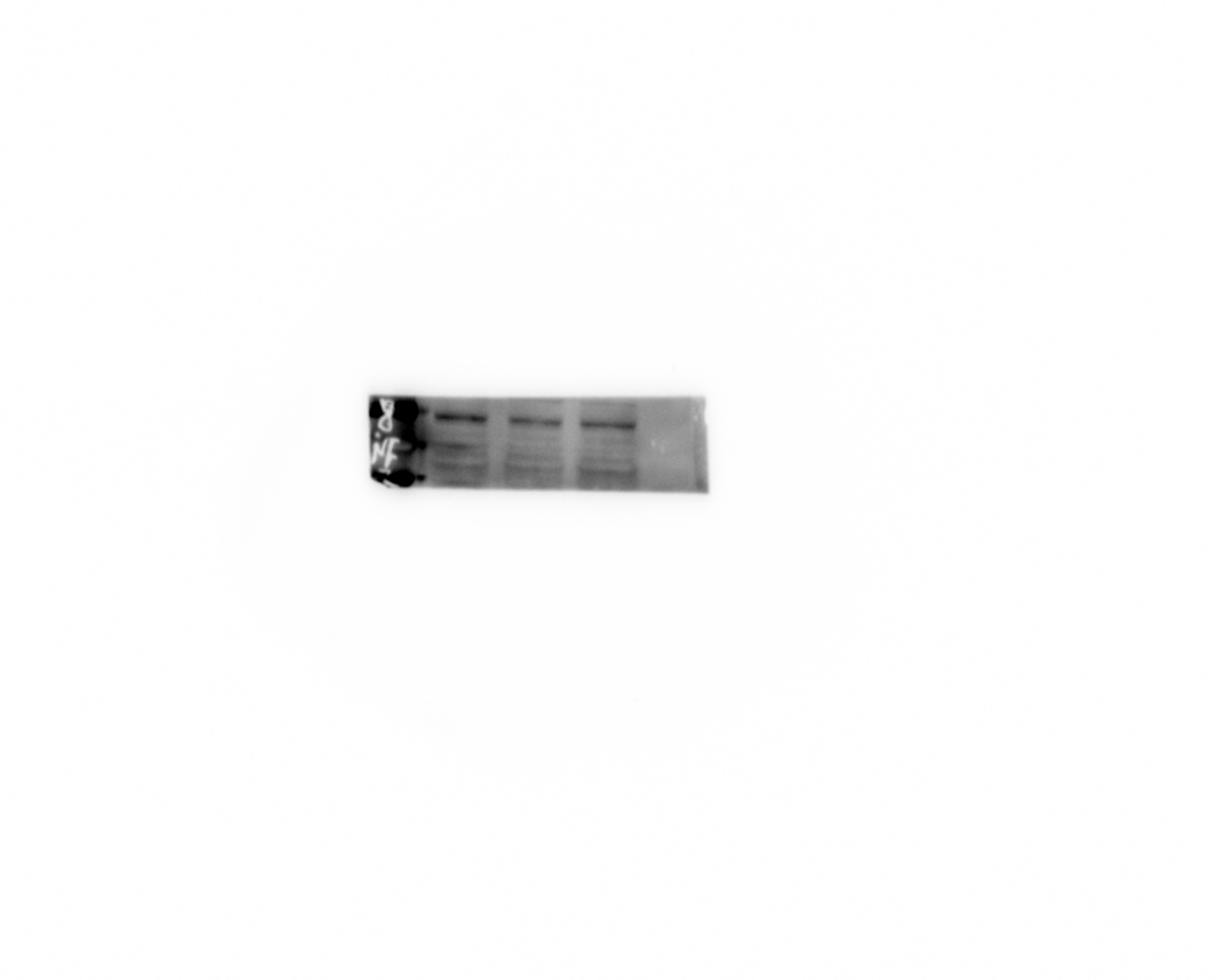

Supplement: Supplementary file 3 [file DataSheet1.ZIP › WB/8226/12p-nfkb/10.2/8P- NF KB.Tif]

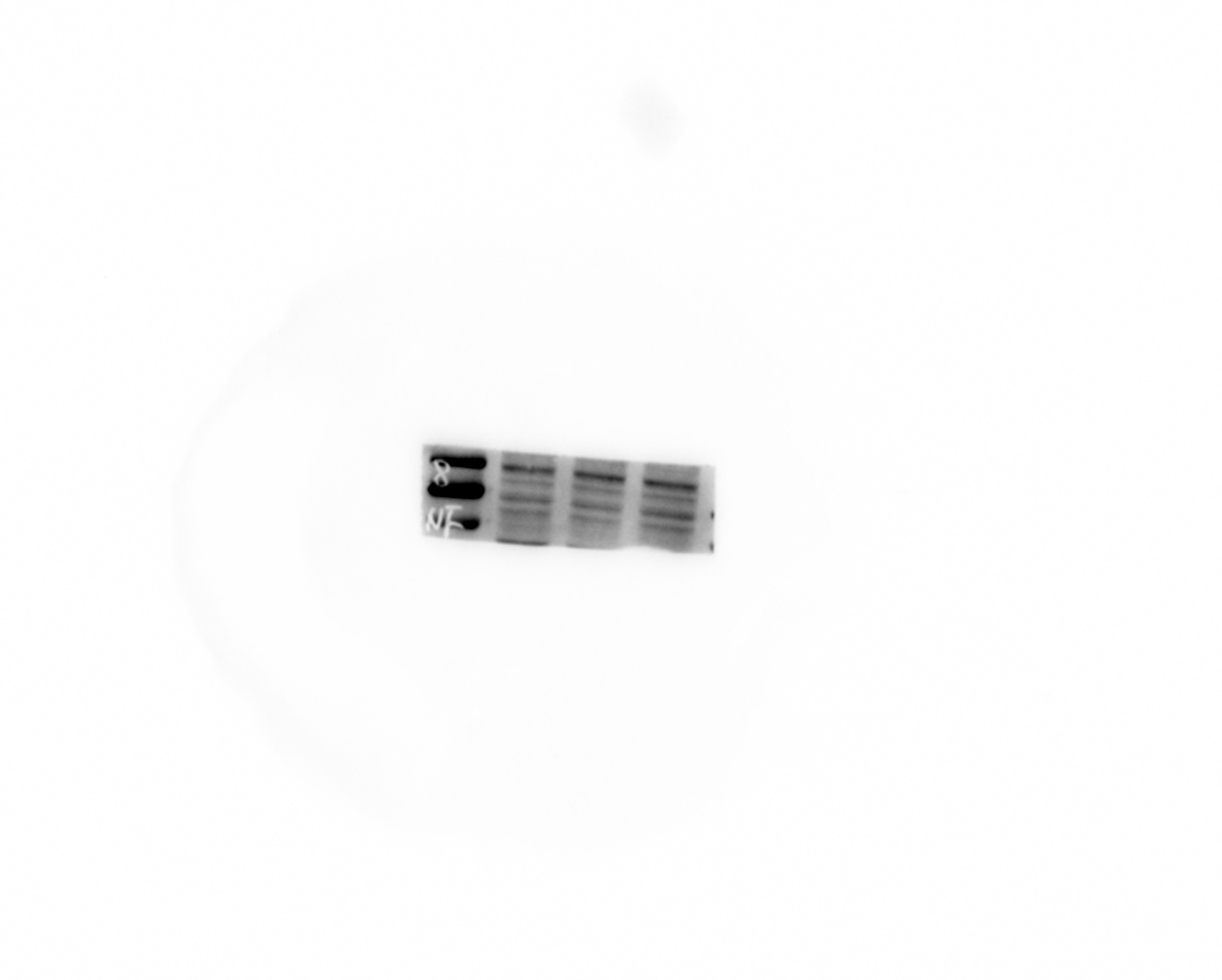

Supplement: Supplementary file 3 [file DataSheet1.ZIP › WB/8226/12p-nfkb/10.4/8 NF.Tif]

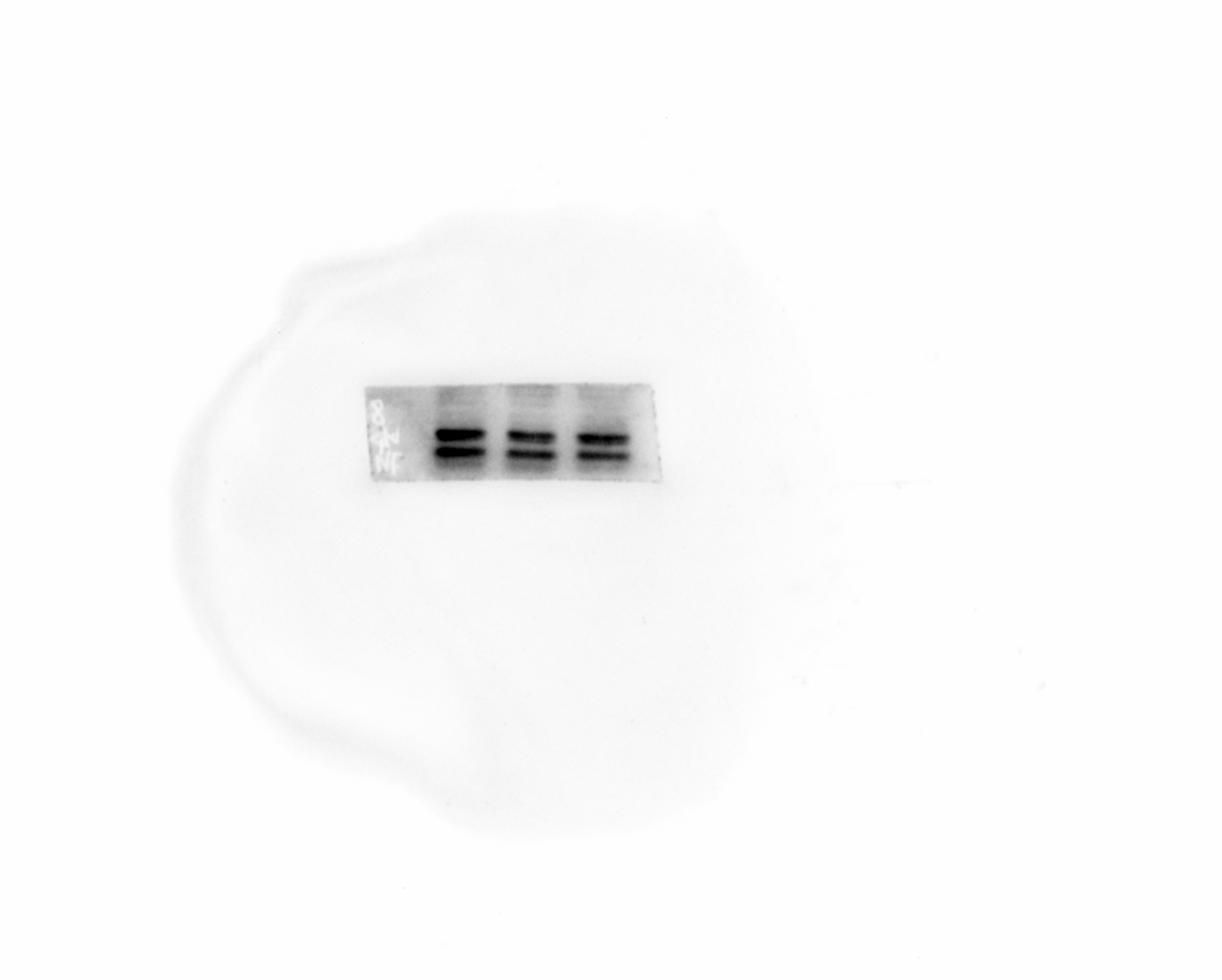

Supplement: Supplementary file 3 [file DataSheet1.ZIP › WB/8226/12p-nfkb/10.4/8GAPDH.Tif]

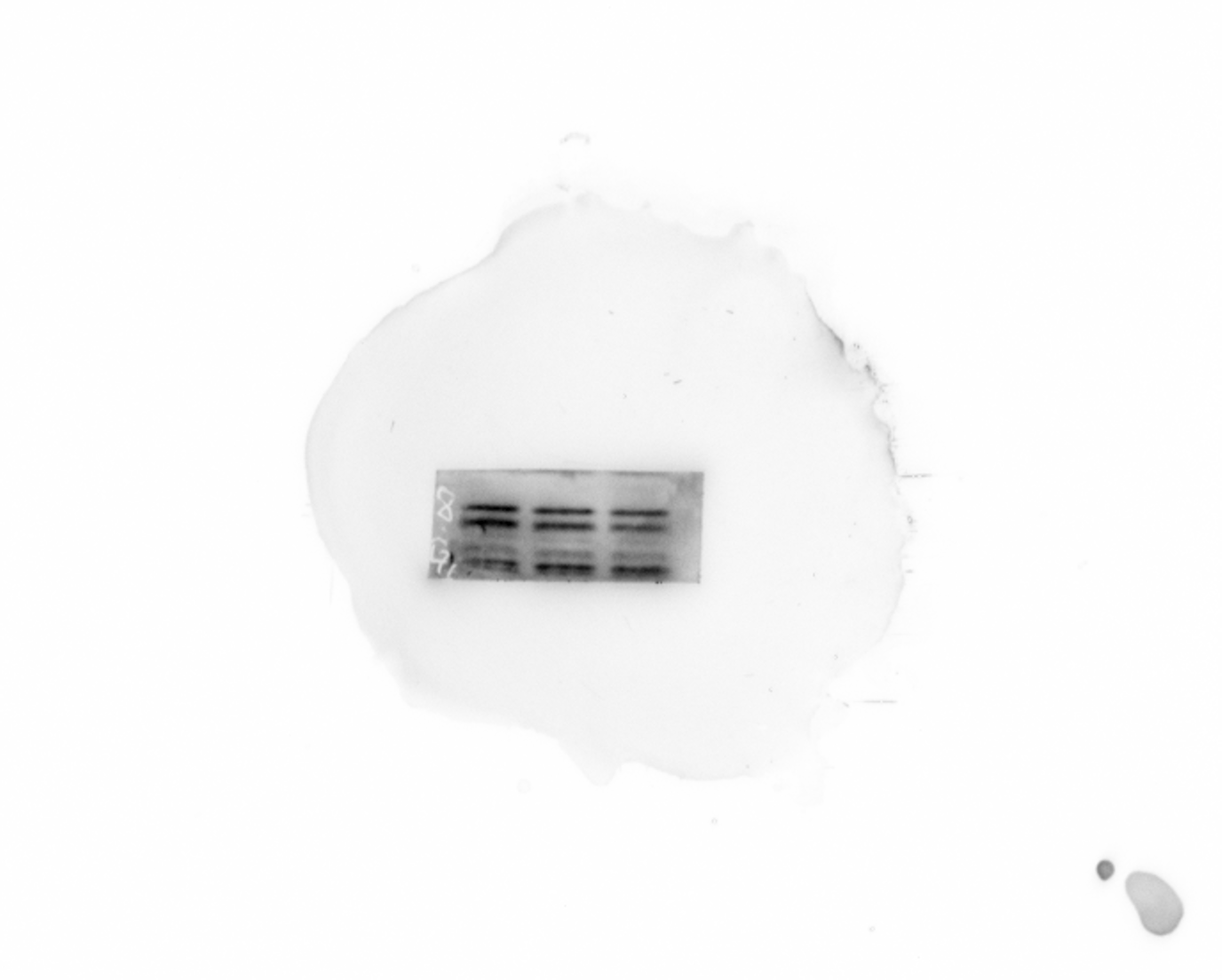

Supplement: Supplementary file 3 [file DataSheet1.ZIP › WB/8226/12p-nfkb/10.7/8GAPDH.Tif]

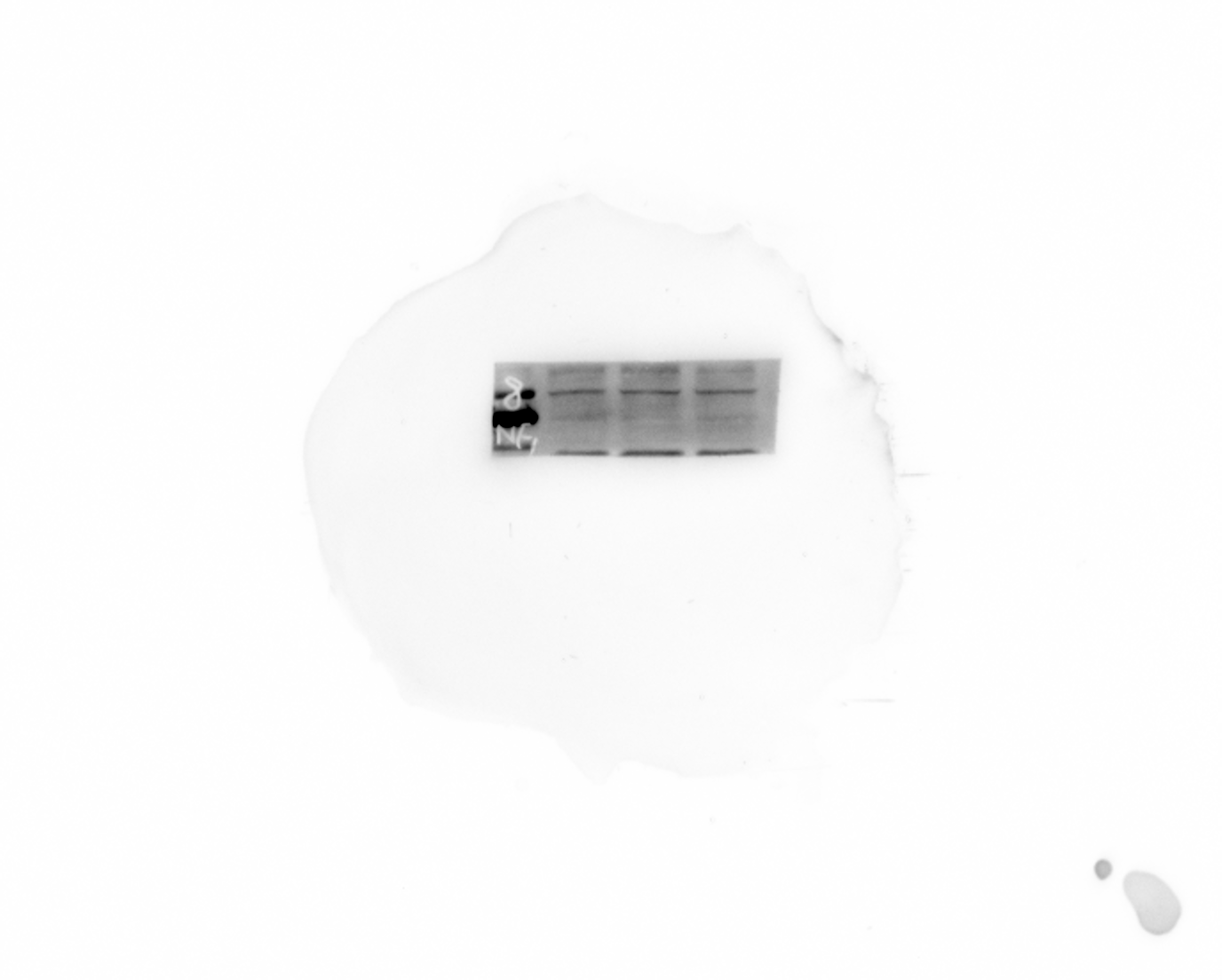

Supplement: Supplementary file 3 [file DataSheet1.ZIP › WB/8226/12p-nfkb/10.7/8NF1.Tif]

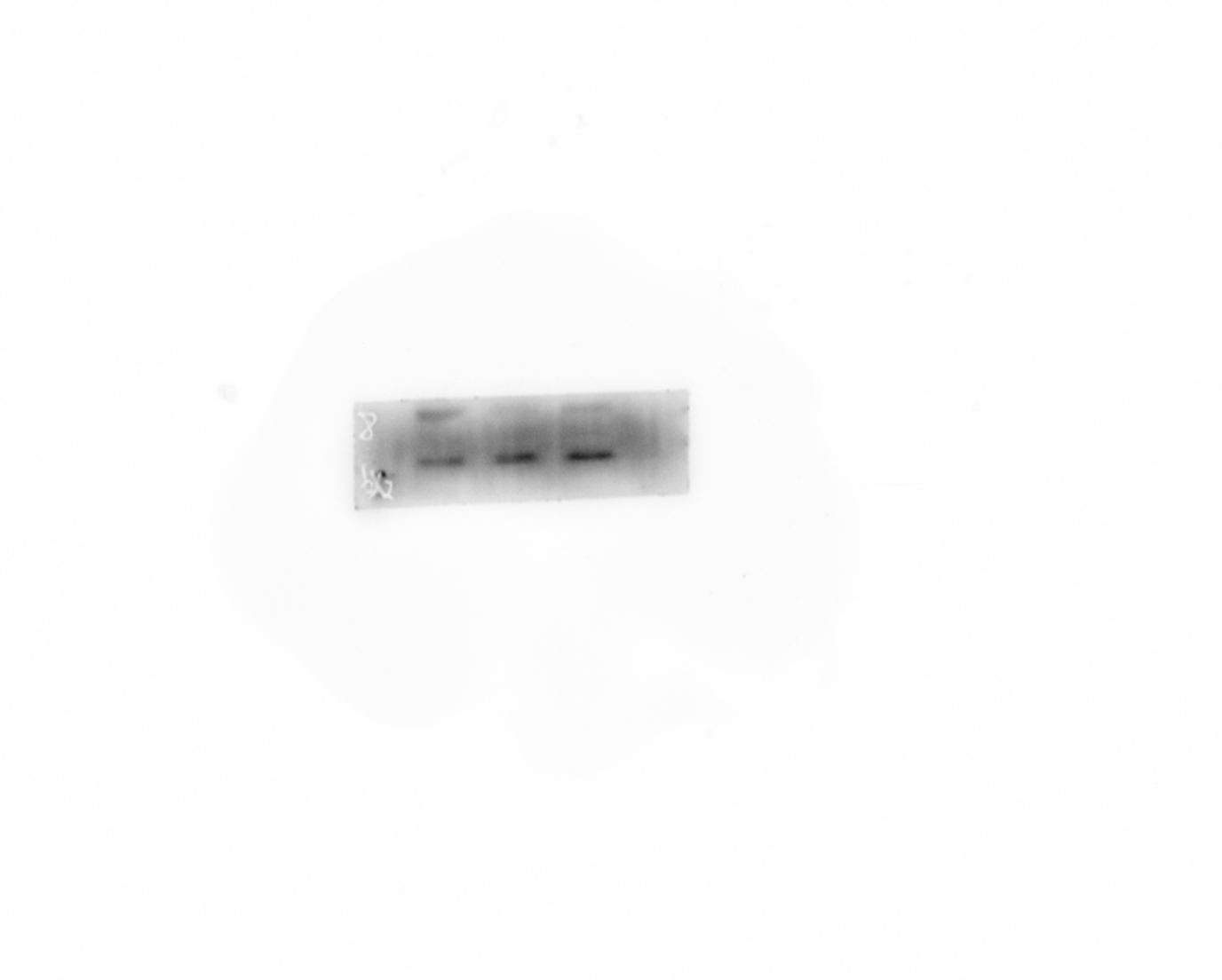

Supplement: Supplementary file 3 [file DataSheet1.ZIP › WB/8226/1BAX/10.2/8BAX 2.Tif]

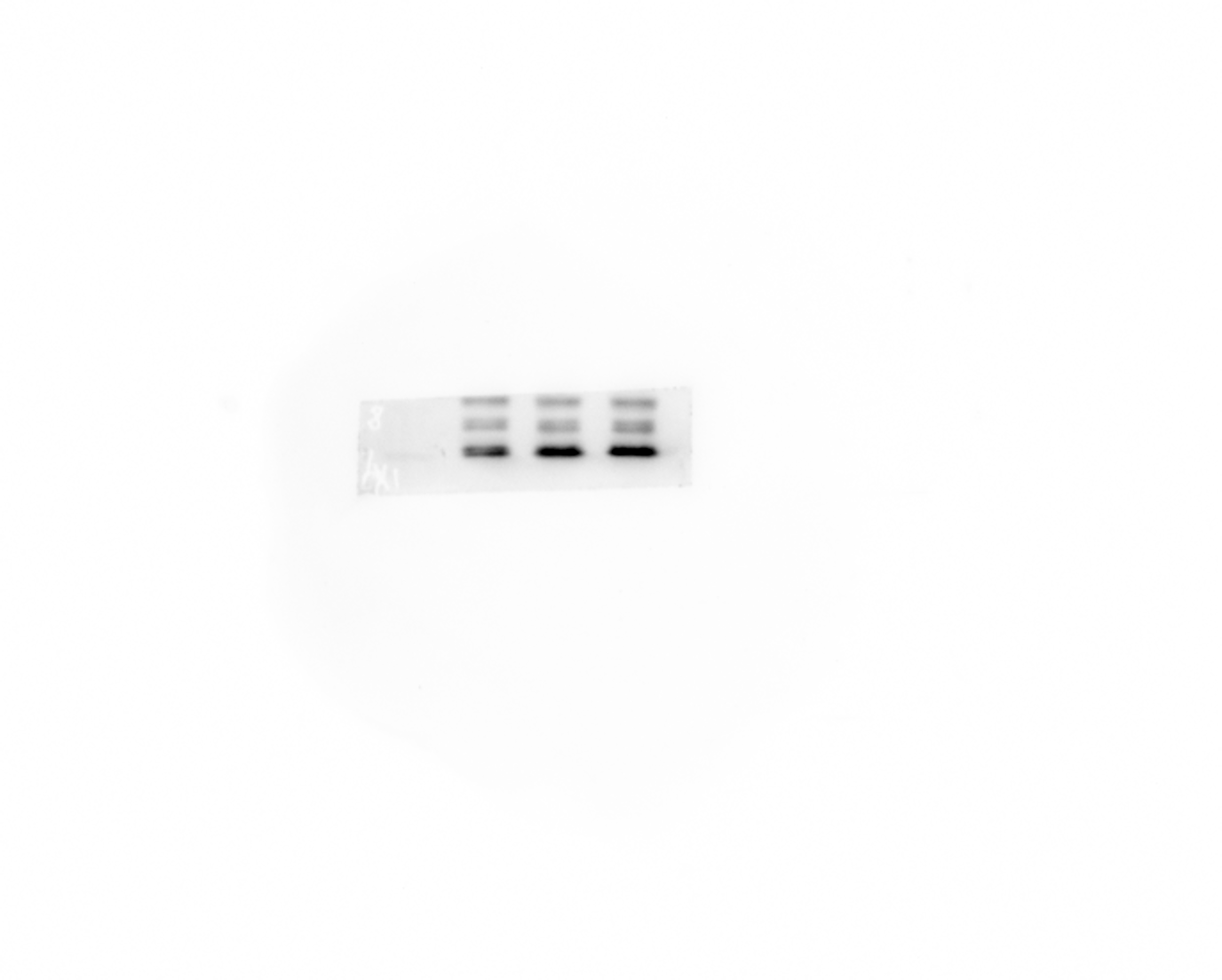

Supplement: Supplementary file 3 [file DataSheet1.ZIP › WB/8226/1BAX/9.24/8 BAX 1.Tif]

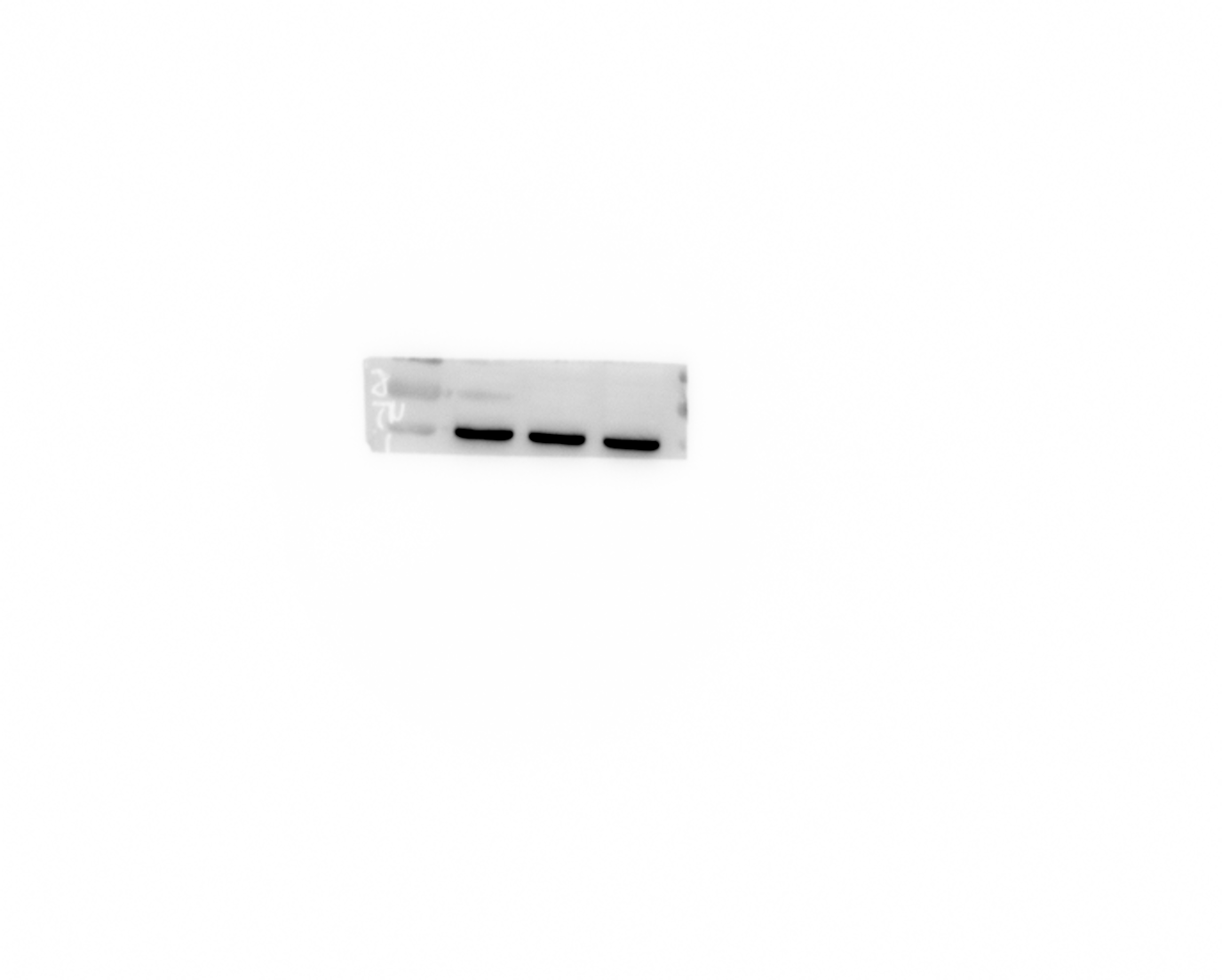

Supplement: Supplementary file 3 [file DataSheet1.ZIP › WB/8226/1BAX/9.24/8226 a┬-actin.Tif]

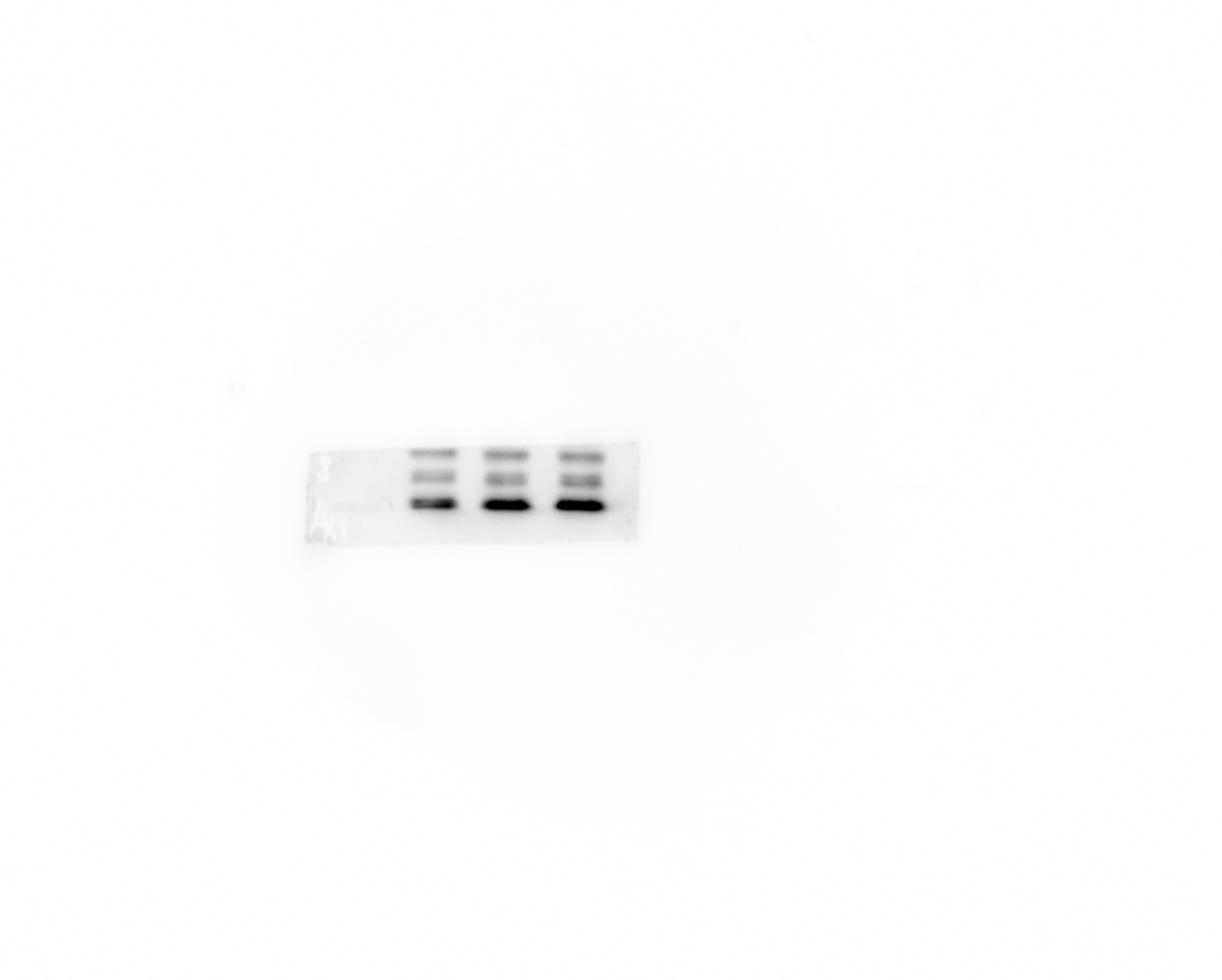

Supplement: Supplementary file 3 [file DataSheet1.ZIP › WB/8226/1BAX/9.24/8BAX 1.Tif]

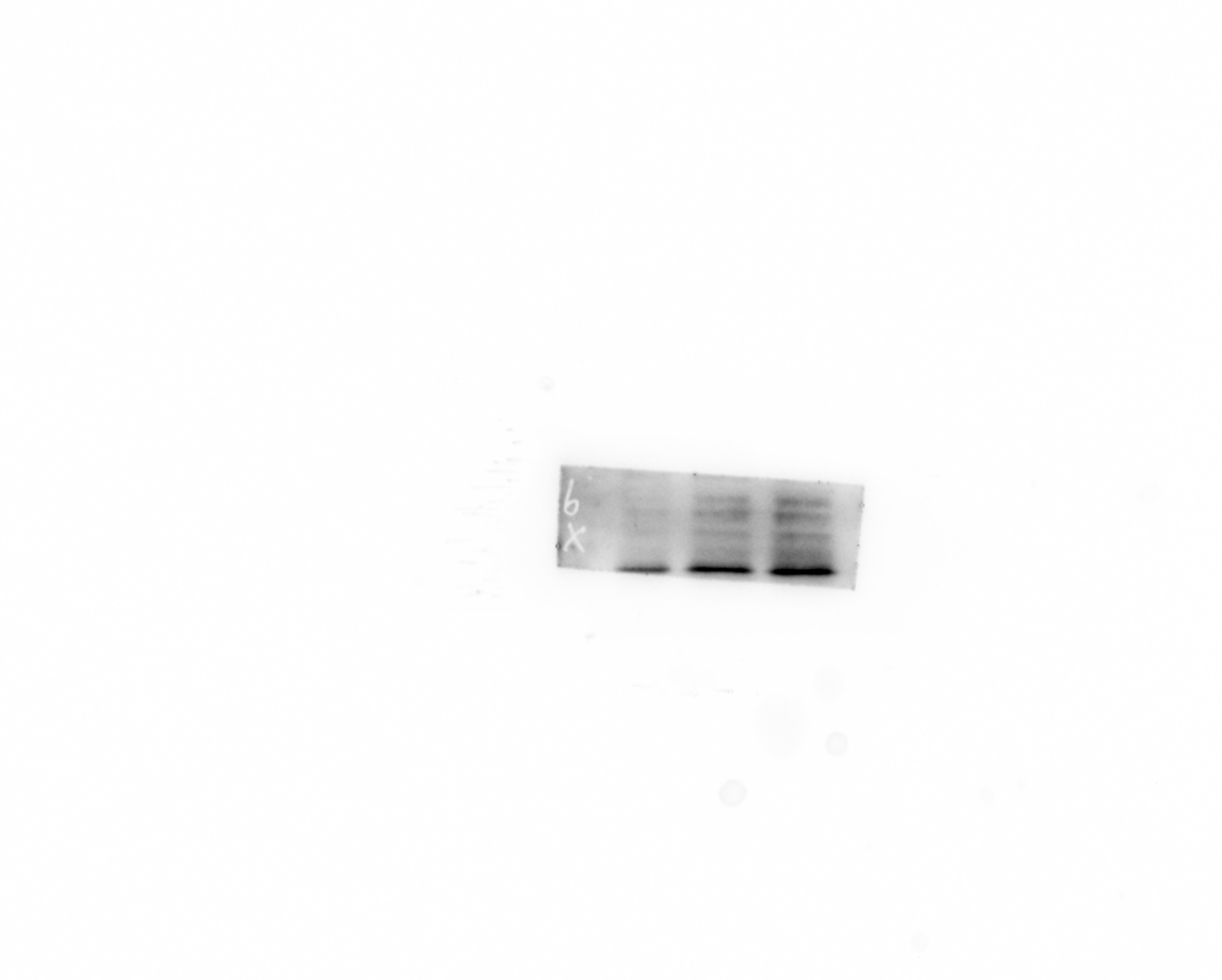

Supplement: Supplementary file 3 [file DataSheet1.ZIP › WB/8226/1BAX/9.29/8226 BAX.Tif]

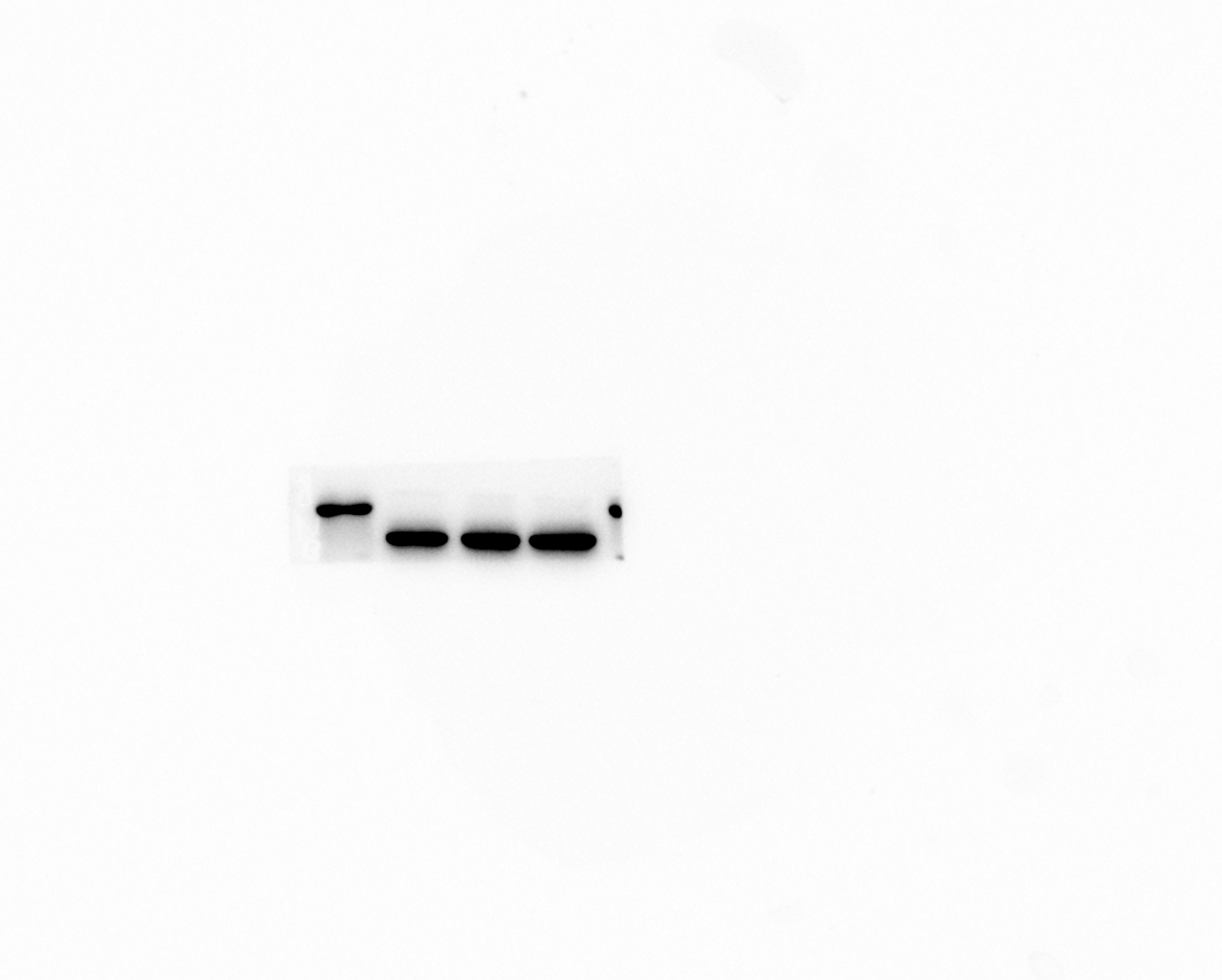

Supplement: Supplementary file 3 [file DataSheet1.ZIP › WB/8226/1BAX/9.29/8226 a┬-actin.Tif]

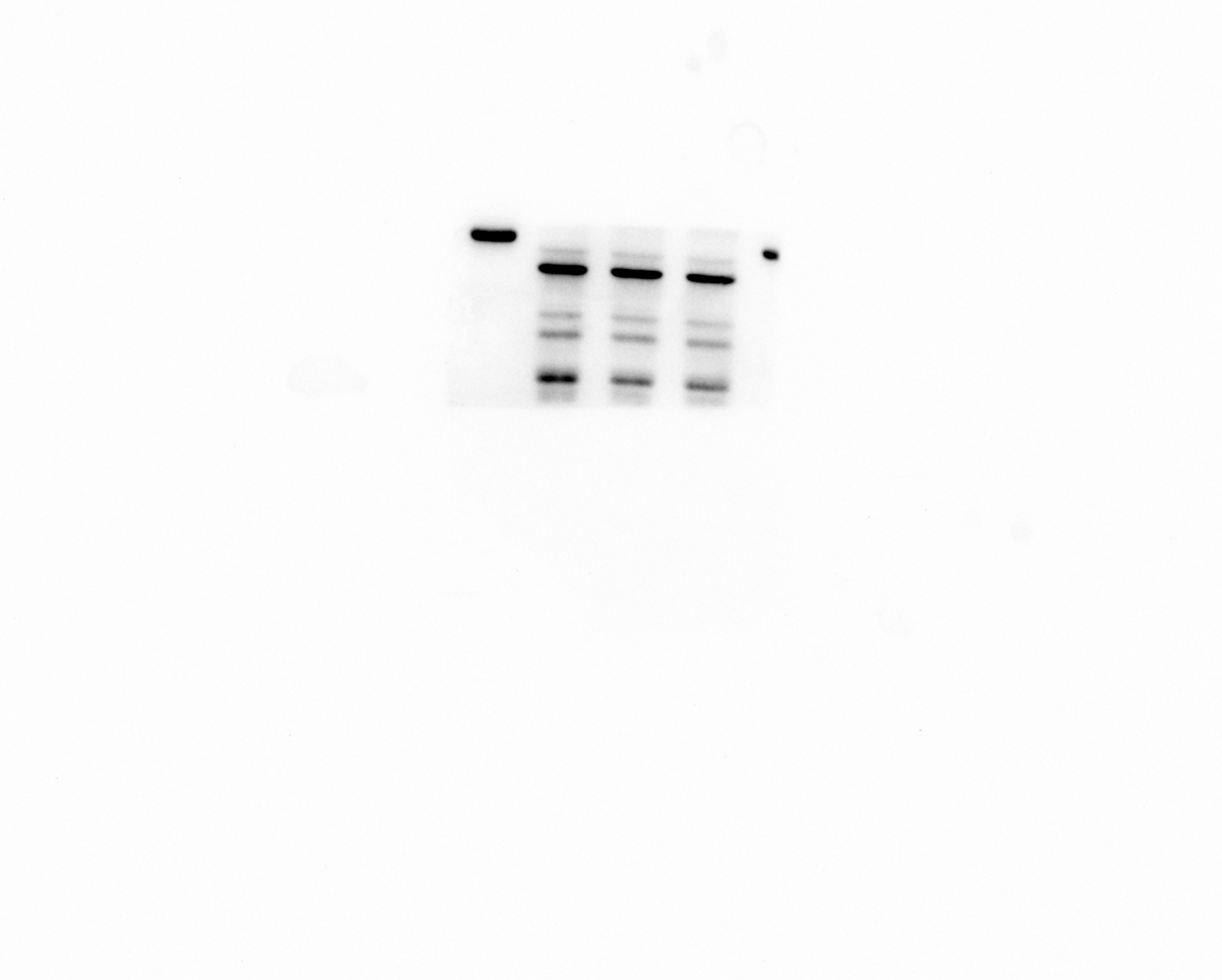

Supplement: Supplementary file 3 [file DataSheet1.ZIP › WB/8226/2bcl2/9.24/8226 BCL2.Tif]

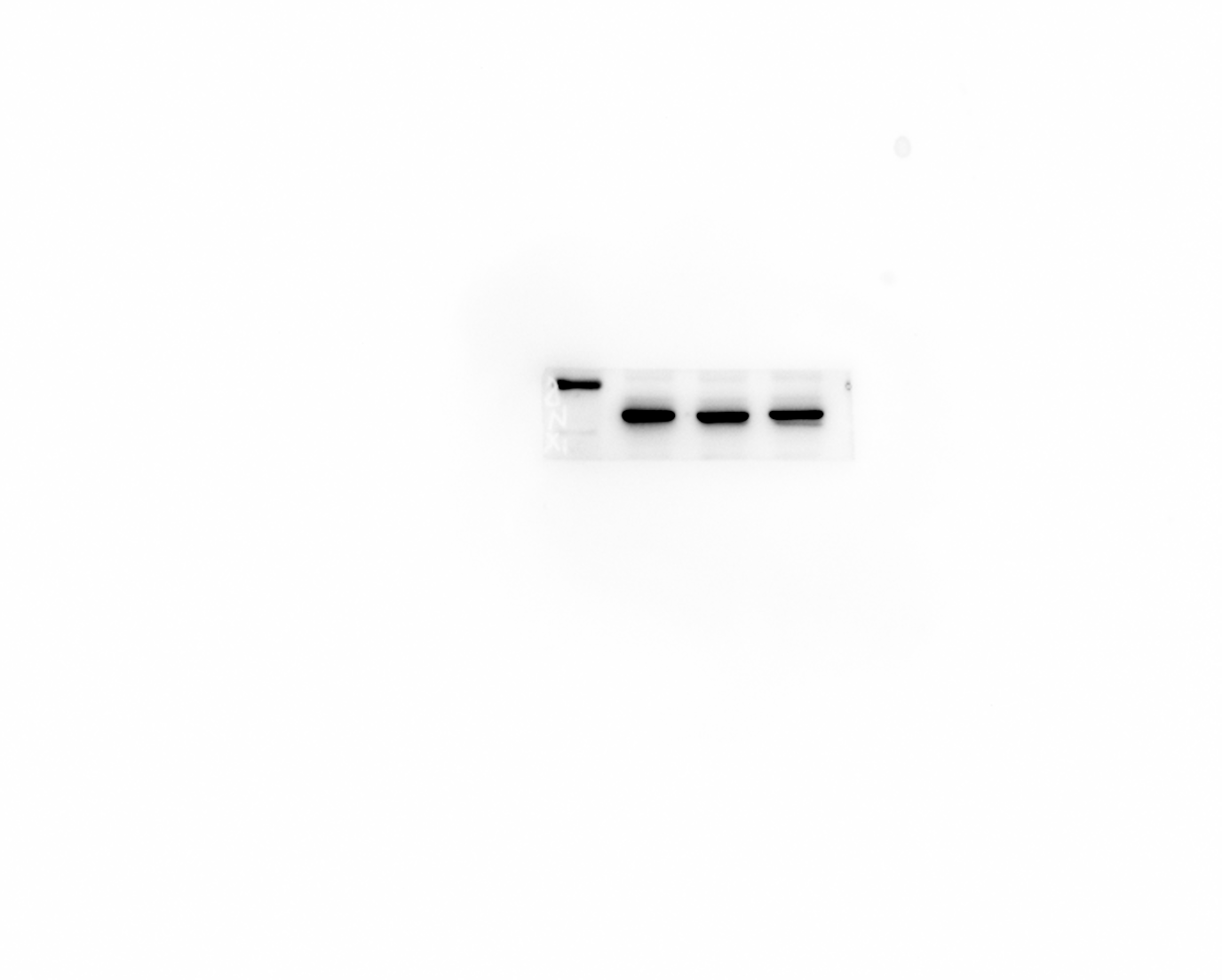

Supplement: Supplementary file 3 [file DataSheet1.ZIP › WB/8226/2bcl2/9.25/8a┬-actin.Tif]

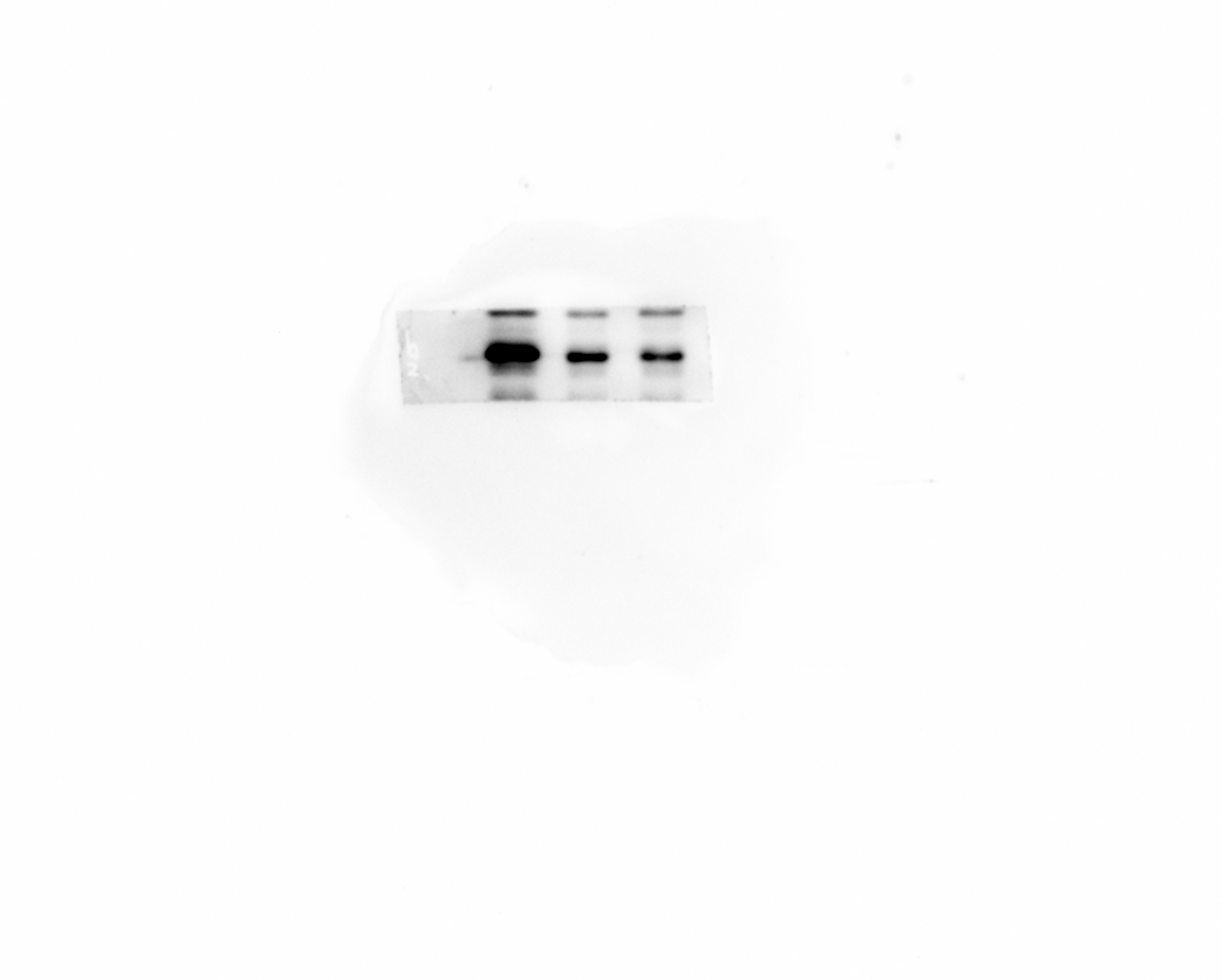

Supplement: Supplementary file 3 [file DataSheet1.ZIP › WB/8226/2bcl2/9.25/BCL2 1.Tif]

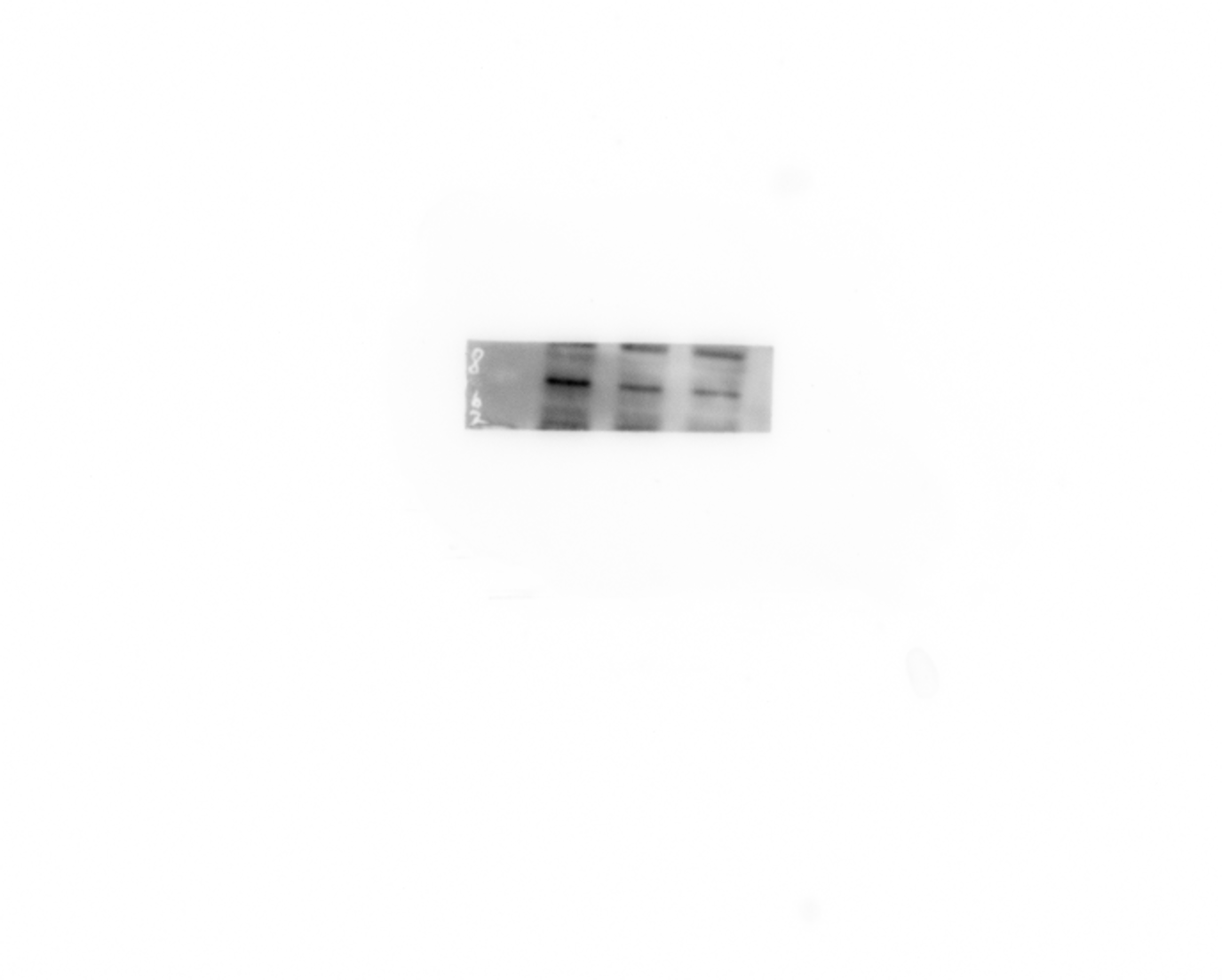

Supplement: Supplementary file 3 [file DataSheet1.ZIP › WB/8226/2bcl2/9.26/8 Bcl 2.Tif]

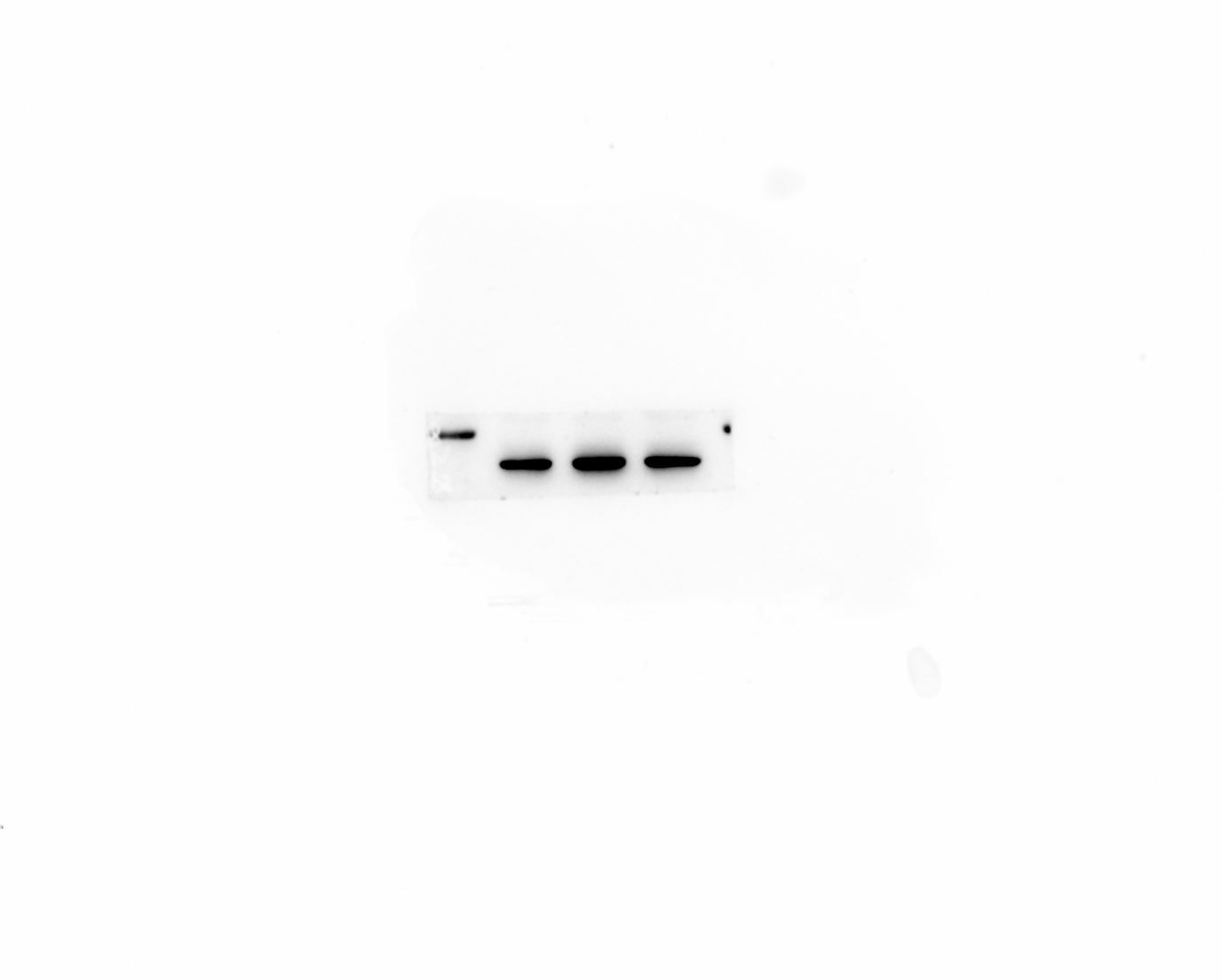

Supplement: Supplementary file 3 [file DataSheet1.ZIP › WB/8226/2bcl2/9.26/8a┬-actin.Tif]

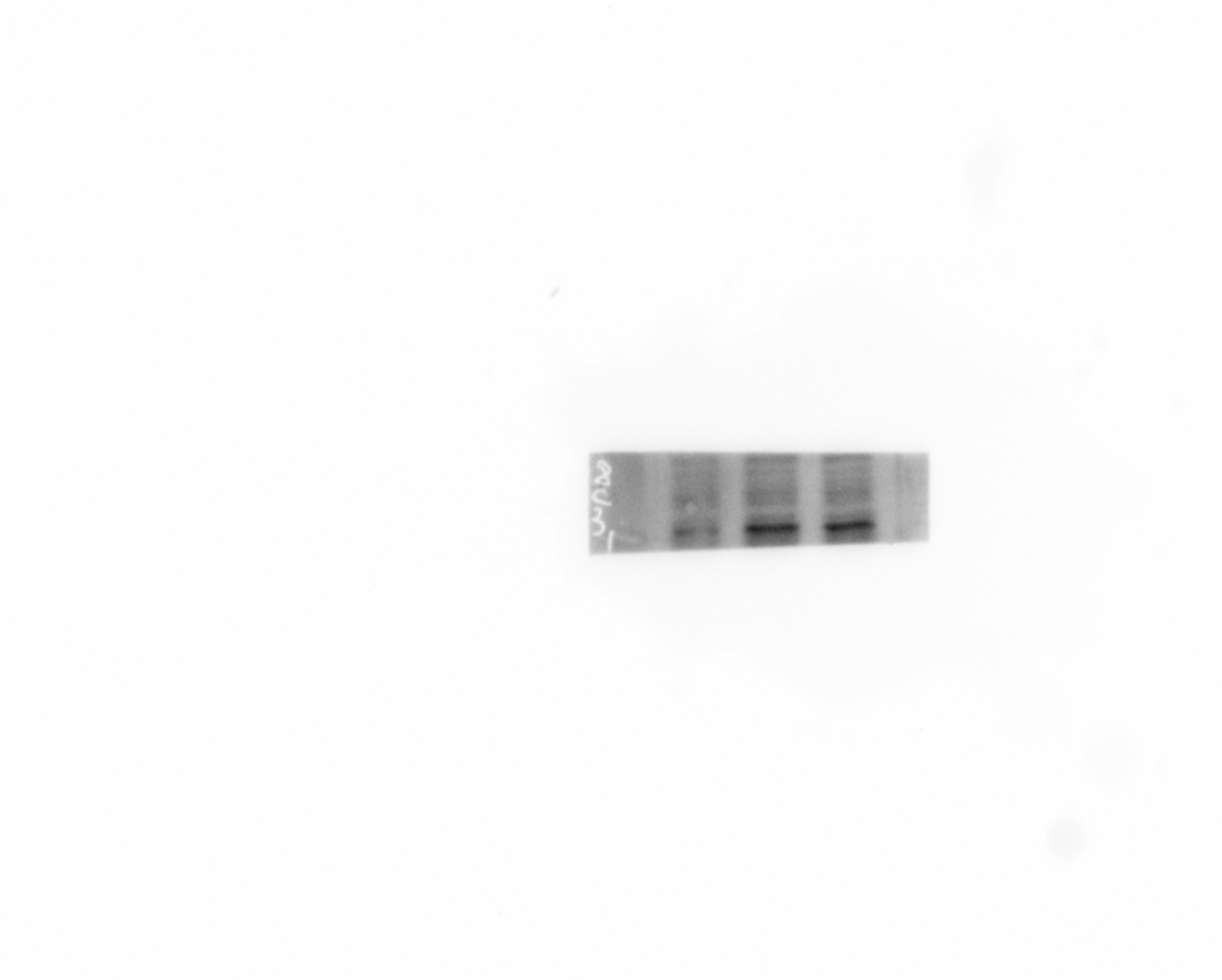

Supplement: Supplementary file 3 [file DataSheet1.ZIP › WB/8226/3CLEAVED-cASPASE 3/9.18/8 CC3.Tif]

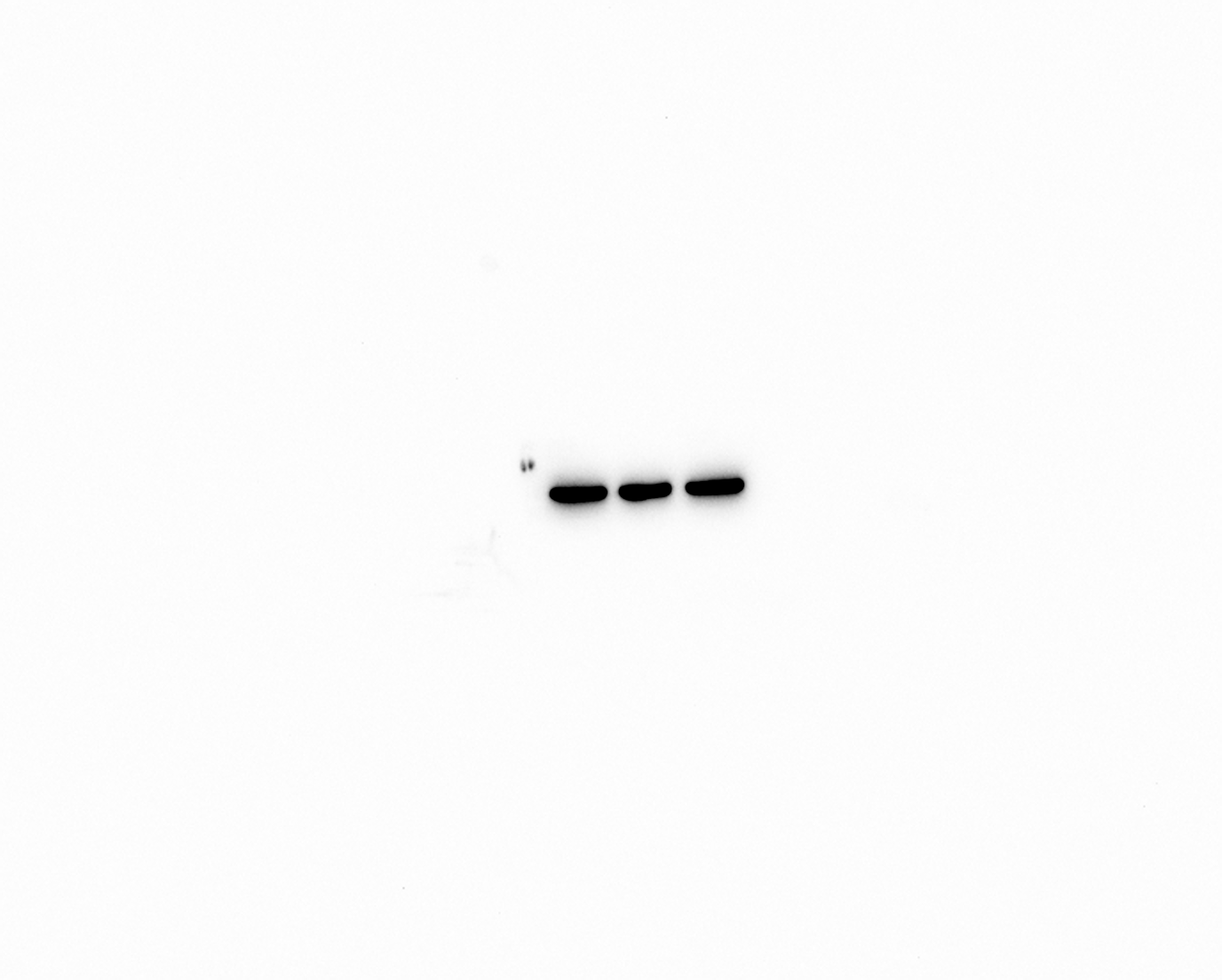

Supplement: Supplementary file 3 [file DataSheet1.ZIP › WB/8226/3CLEAVED-cASPASE 3/9.18/8 a┬-actin.Tif]

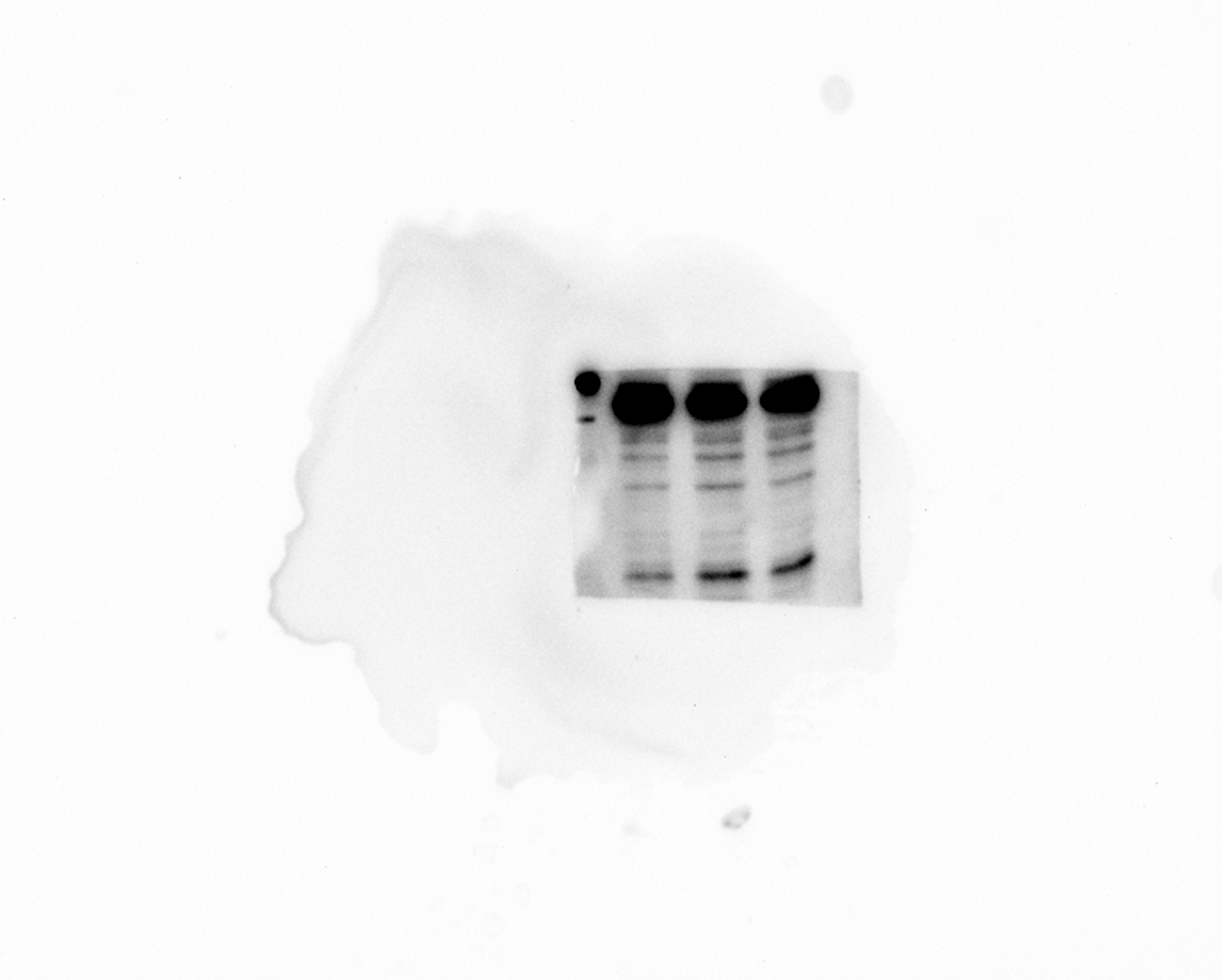

Supplement: Supplementary file 3 [file DataSheet1.ZIP › WB/8226/3CLEAVED-cASPASE 3/9.20/8226 CC3.Tif]

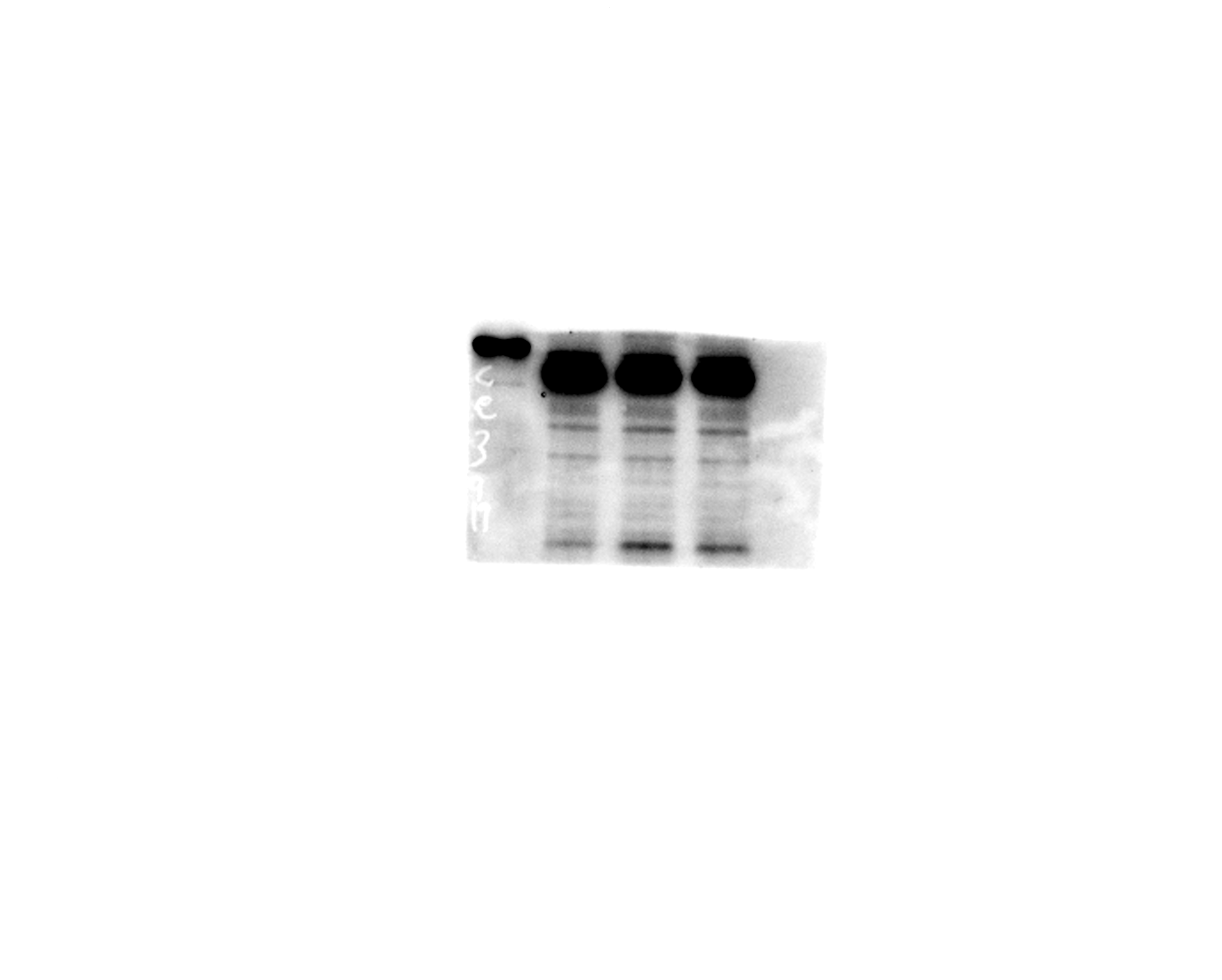

Supplement: Supplementary file 3 [file DataSheet1.ZIP › WB/8226/3CLEAVED-cASPASE 3/9.21/8226 casps 3.Tif]

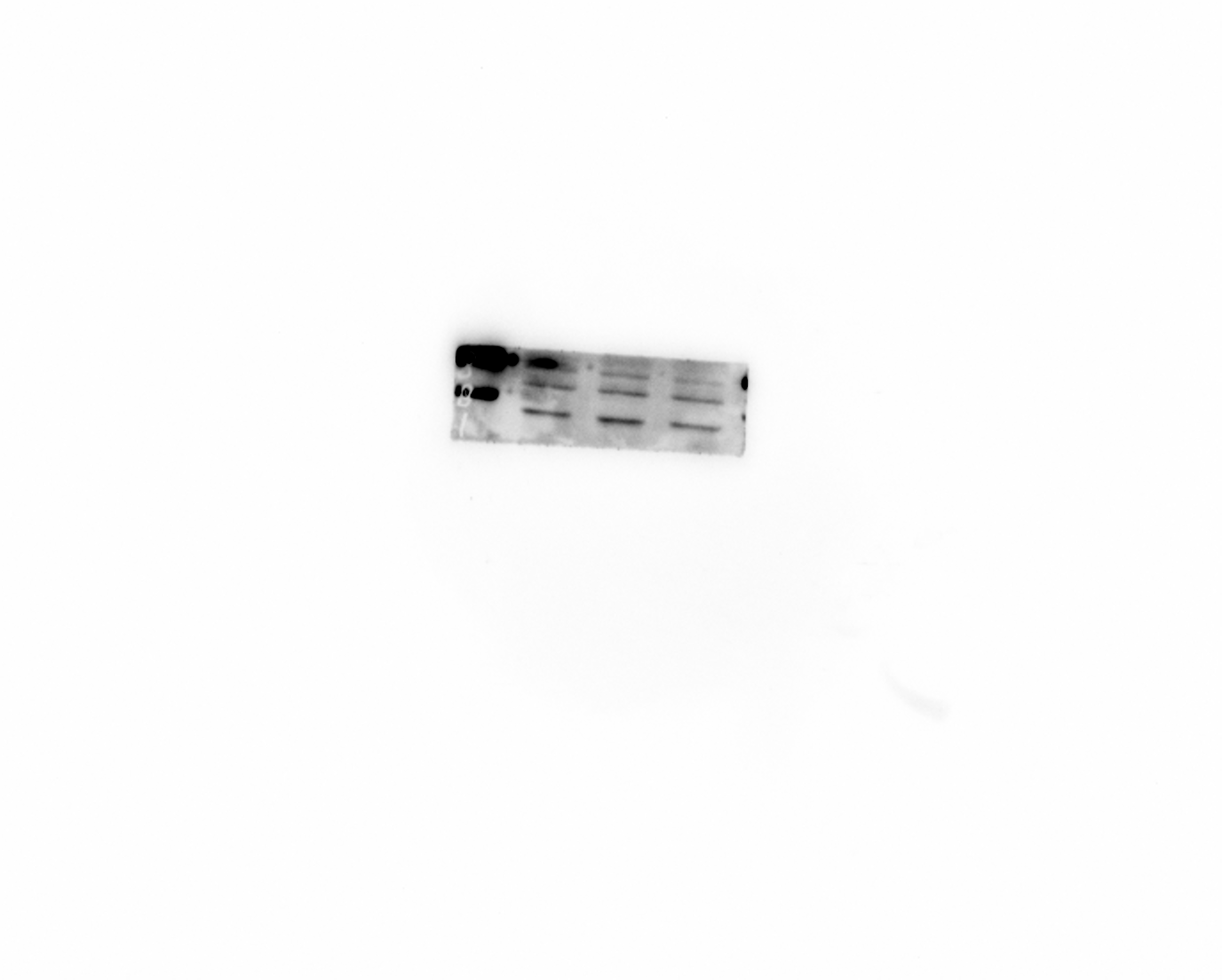

Supplement: Supplementary file 3 [file DataSheet1.ZIP › WB/8226/4CCNB1/10.3/8 CCNB1.Tif]

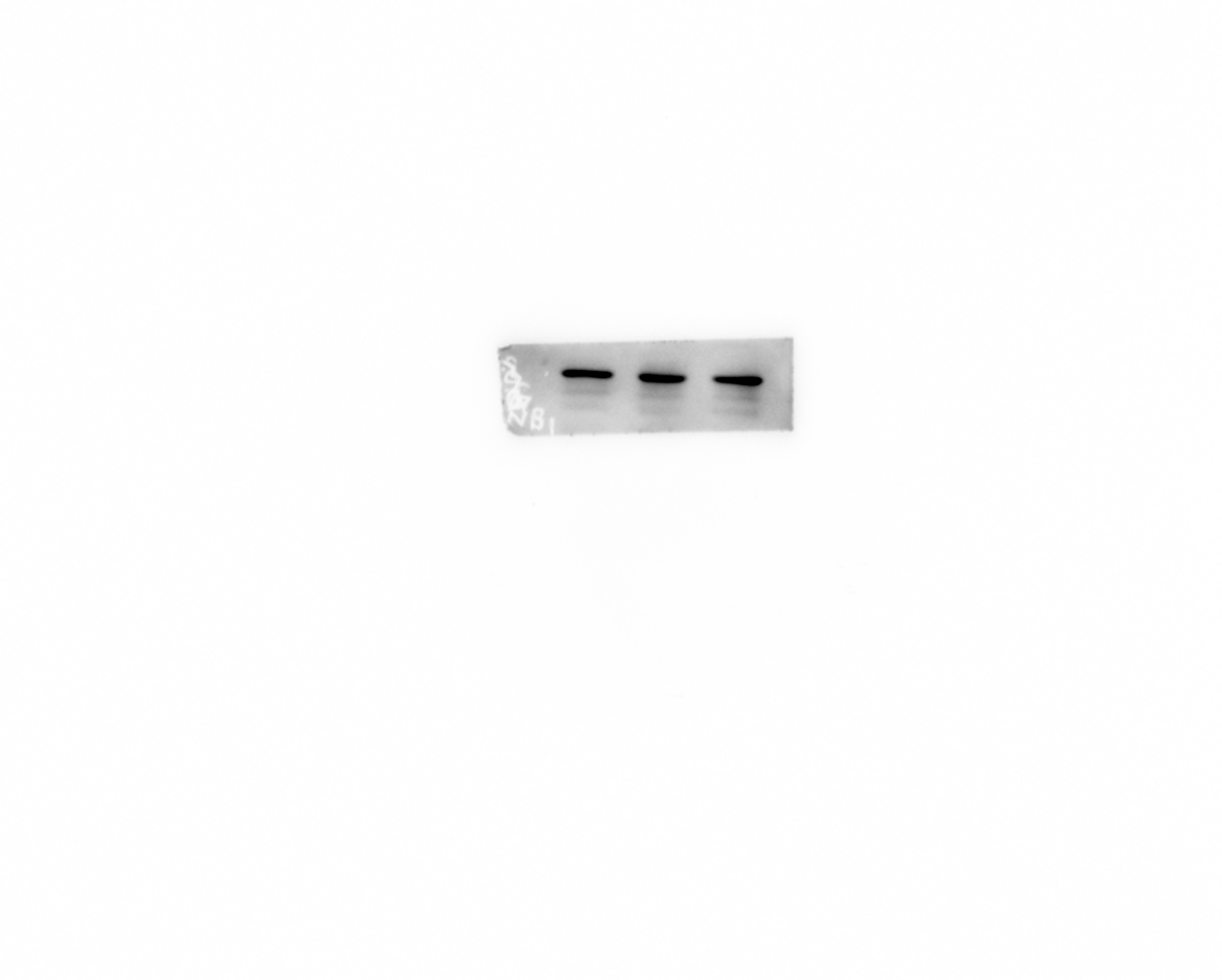

Supplement: Supplementary file 3 [file DataSheet1.ZIP › WB/8226/4CCNB1/10.3/8GAPDH.Tif]

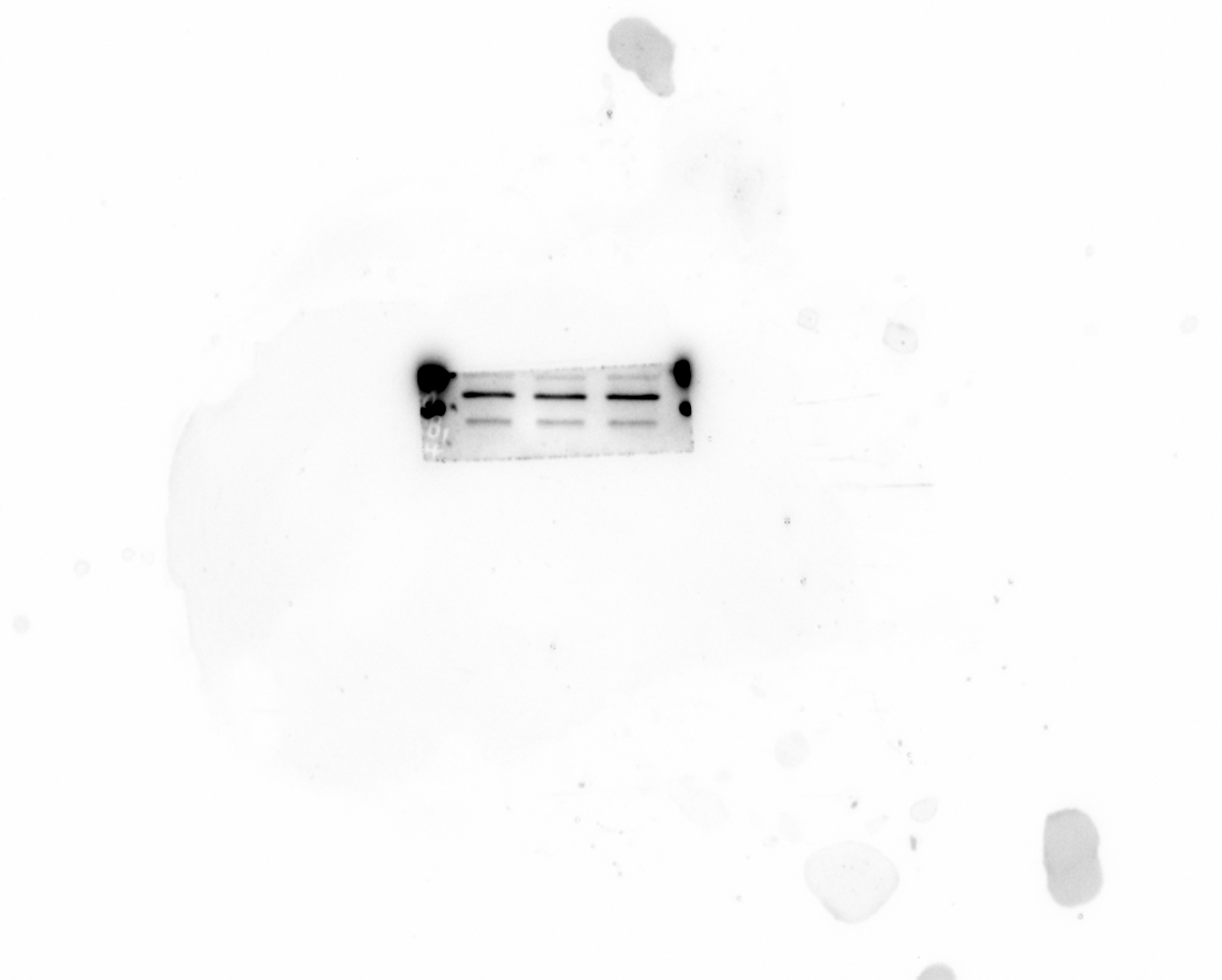

Supplement: Supplementary file 3 [file DataSheet1.ZIP › WB/8226/4CCNB1/9.28/8 CCNB1.Tif]

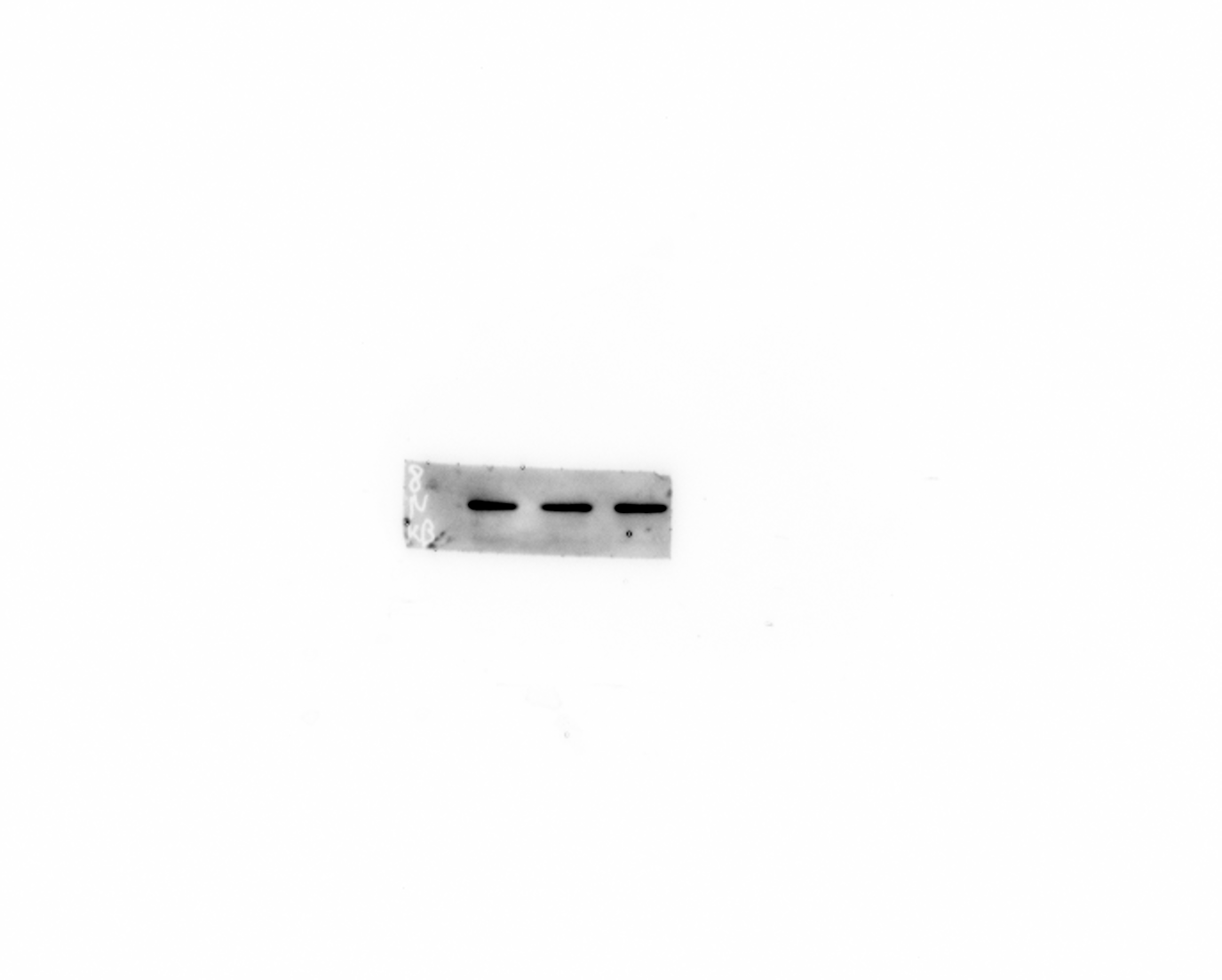

Supplement: Supplementary file 3 [file DataSheet1.ZIP › WB/8226/4CCNB1/9.28/8gapdh.Tif]

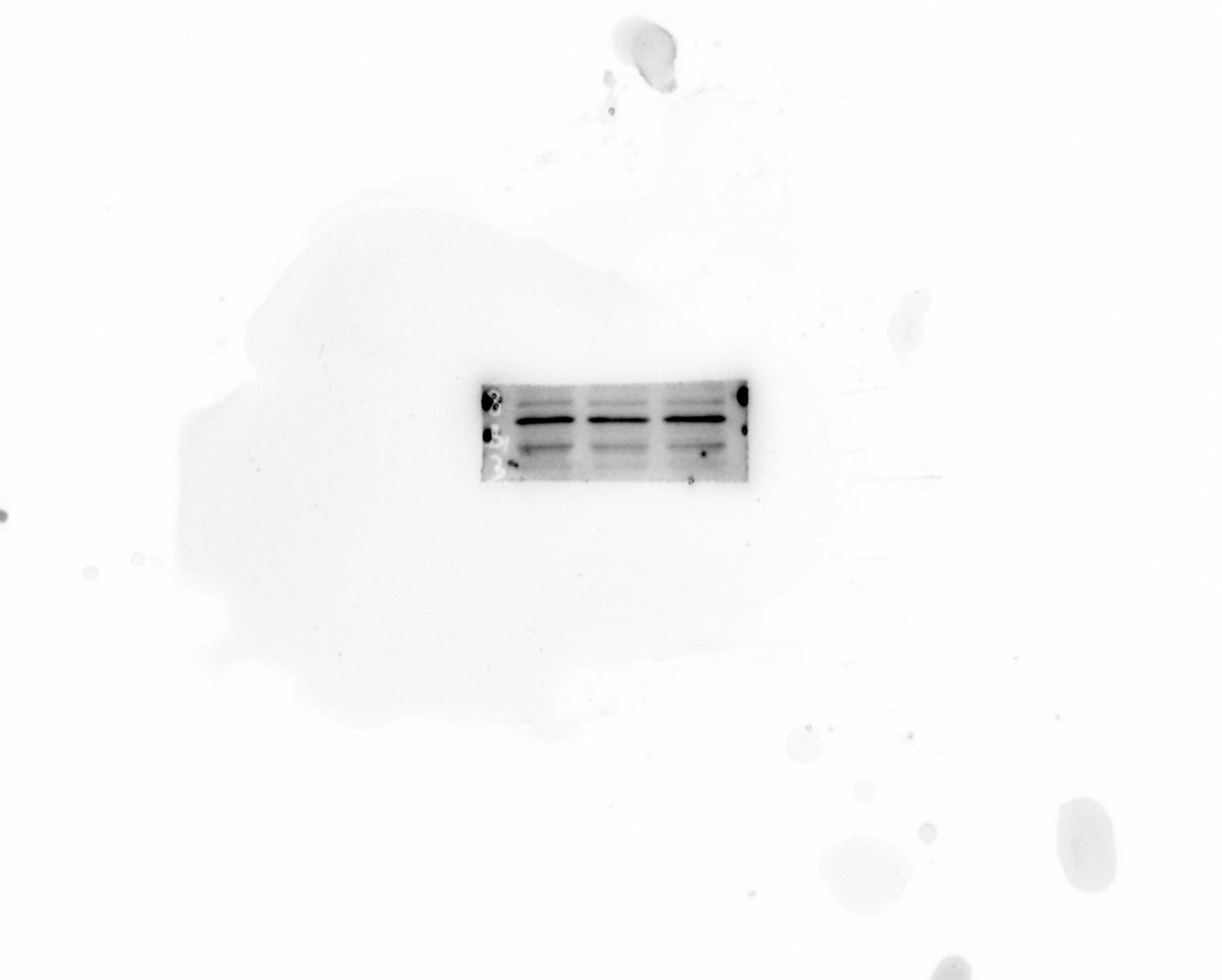

Supplement: Supplementary file 3 [file DataSheet1.ZIP › WB/8226/4CCNB1/9.29/8 CCNB1.Tif]

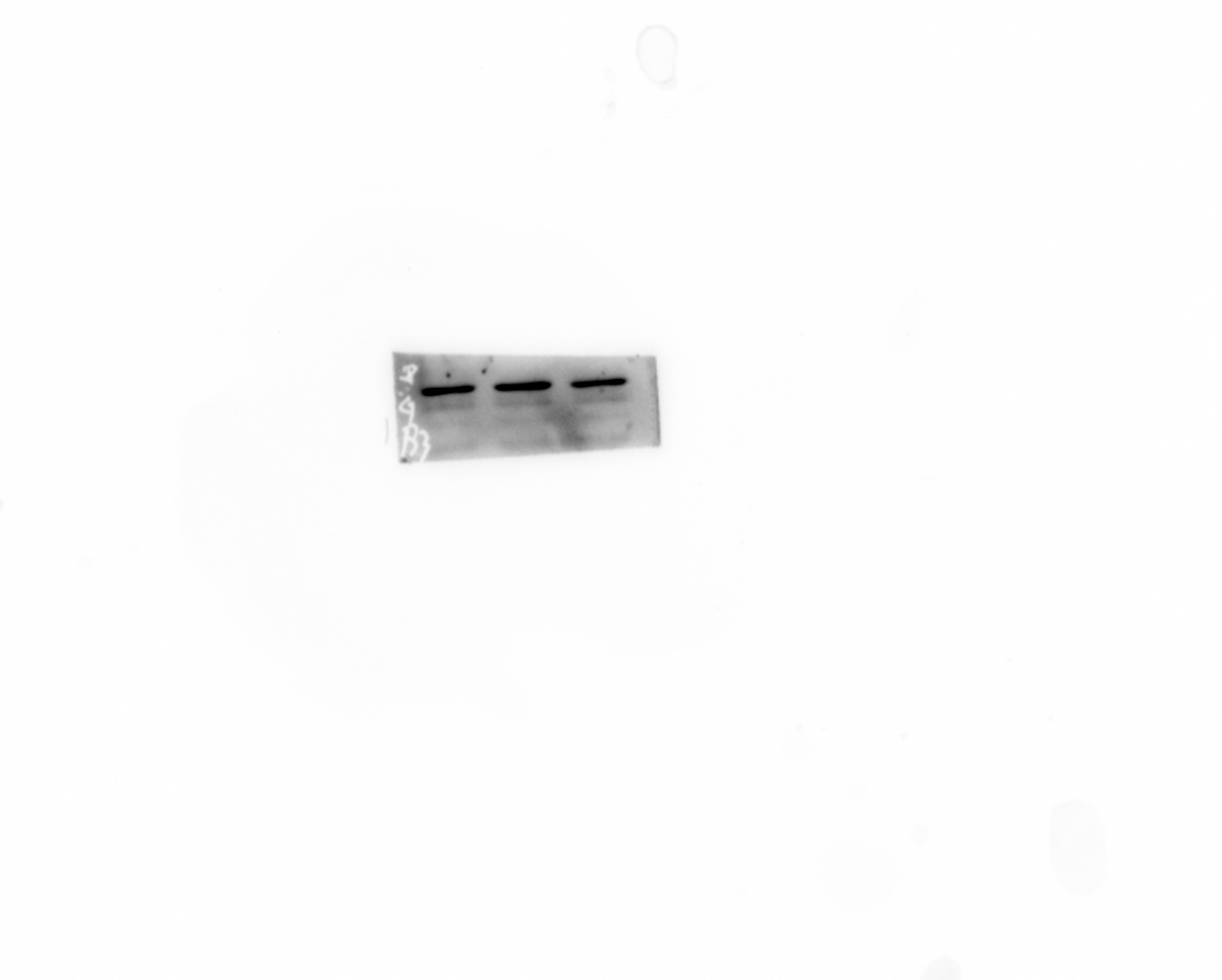

Supplement: Supplementary file 3 [file DataSheet1.ZIP › WB/8226/4CCNB1/9.29/8GAPDH.Tif]

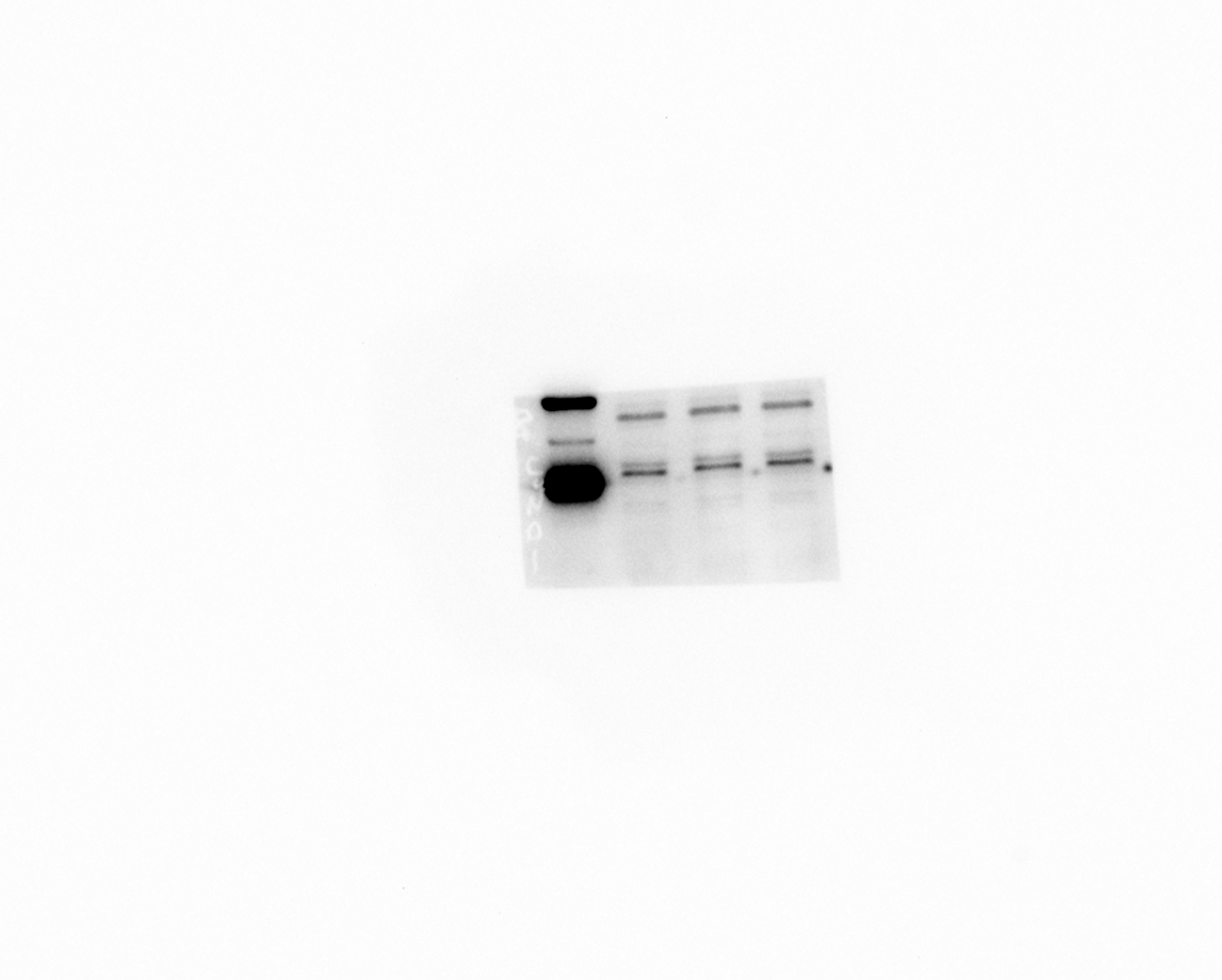

Supplement: Supplementary file 3 [file DataSheet1.ZIP › WB/8226/5CCND1/9.18/8 CD1.Tif]

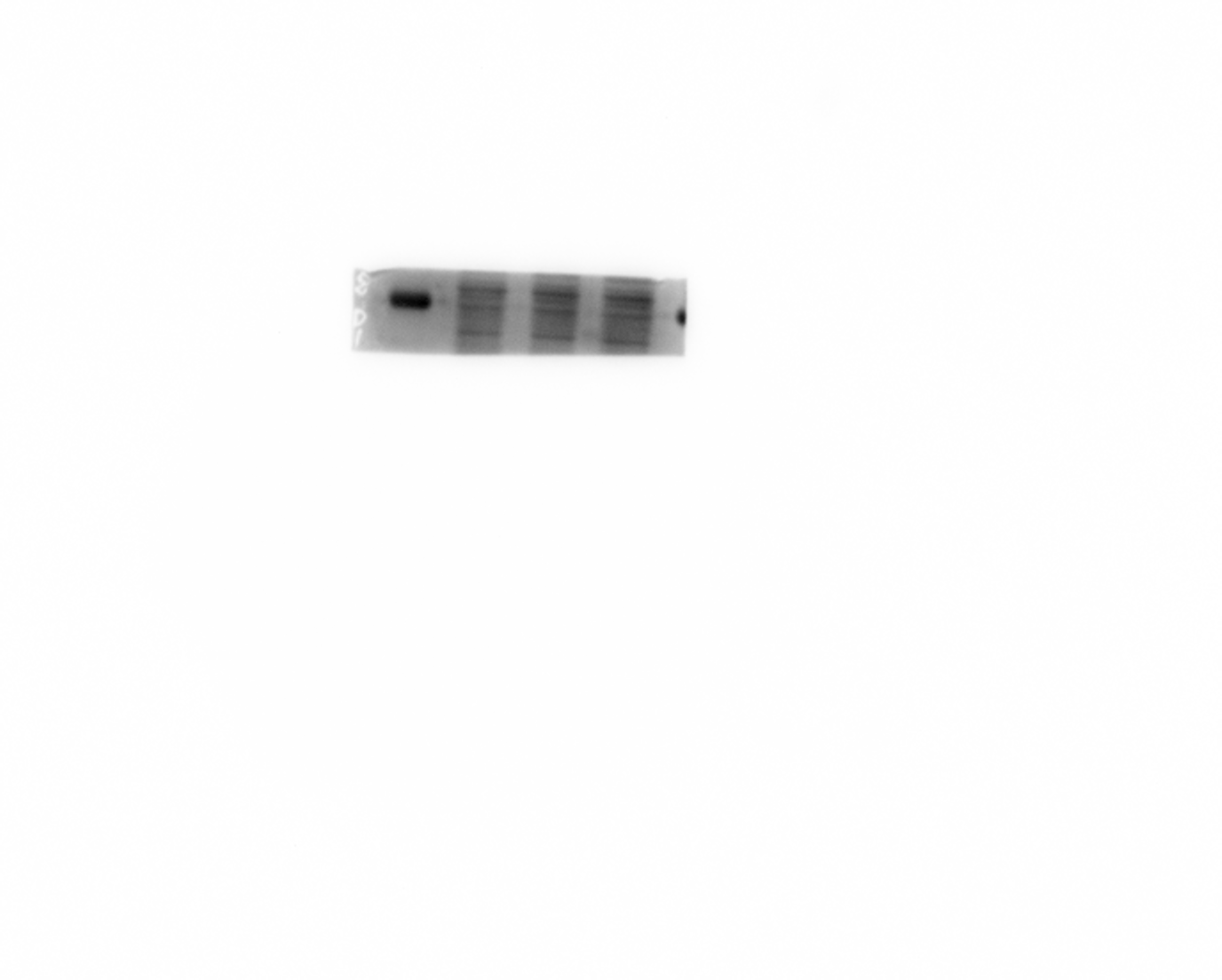

Supplement: Supplementary file 3 [file DataSheet1.ZIP › WB/8226/5CCND1/9.22/8CCND1.Tif]

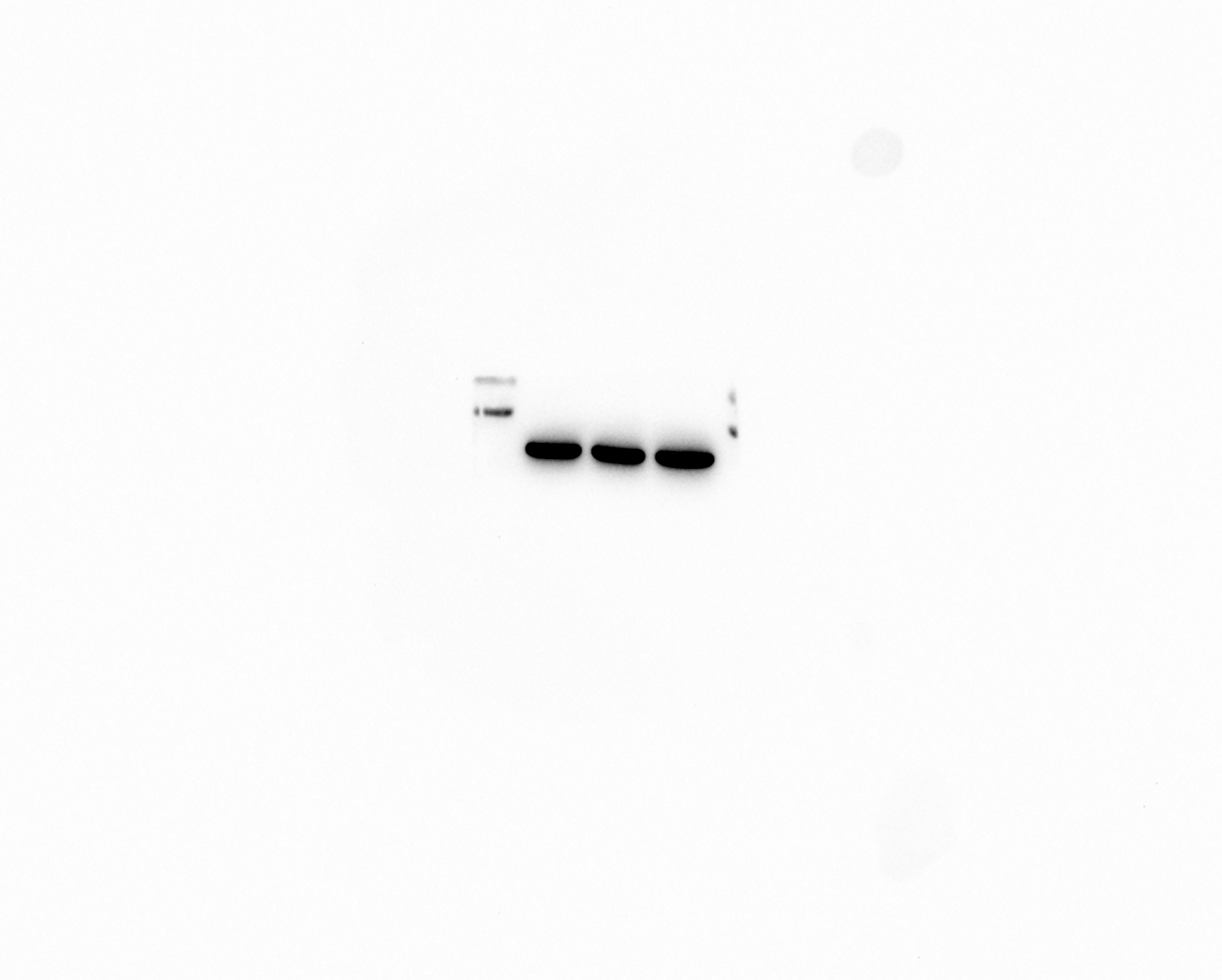

Supplement: Supplementary file 3 [file DataSheet1.ZIP › WB/8226/5CCND1/9.22/a┬-actin.Tif]

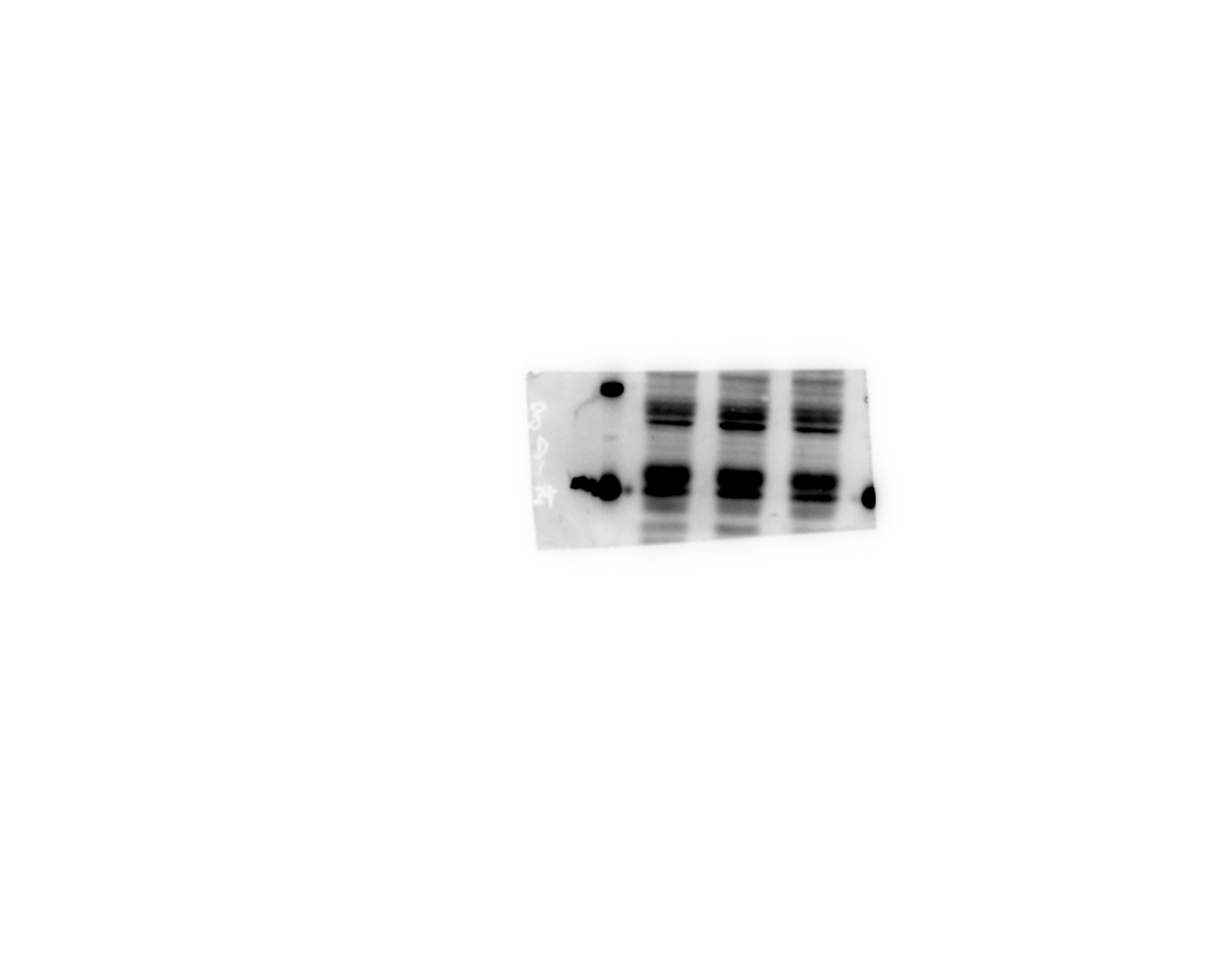

Supplement: Supplementary file 3 [file DataSheet1.ZIP › WB/8226/5CCND1/9.26/8226 CCND1.Tif]

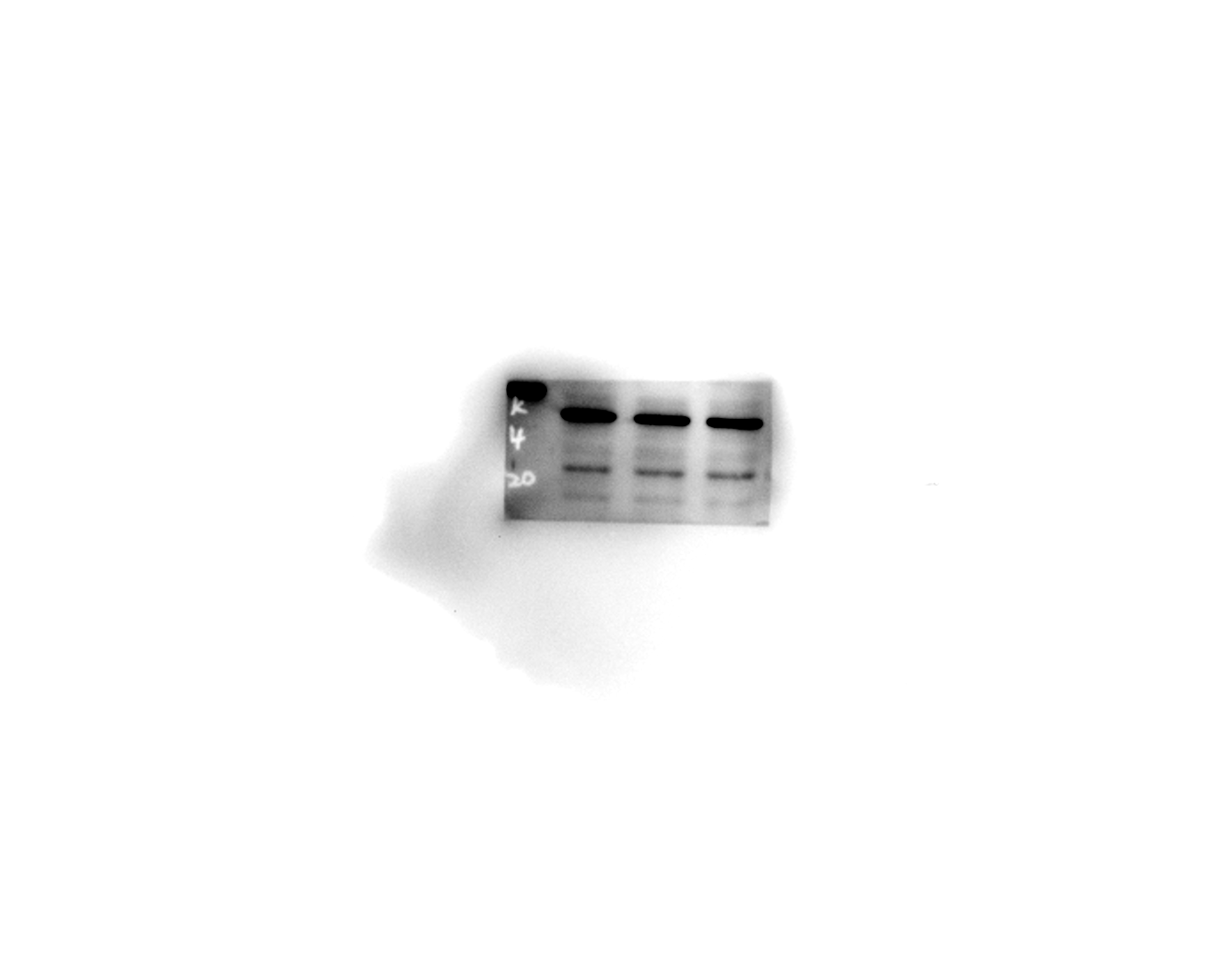

Supplement: Supplementary file 3 [file DataSheet1.ZIP › WB/8226/6cdk4/9.22/CDK4 1.Tif]

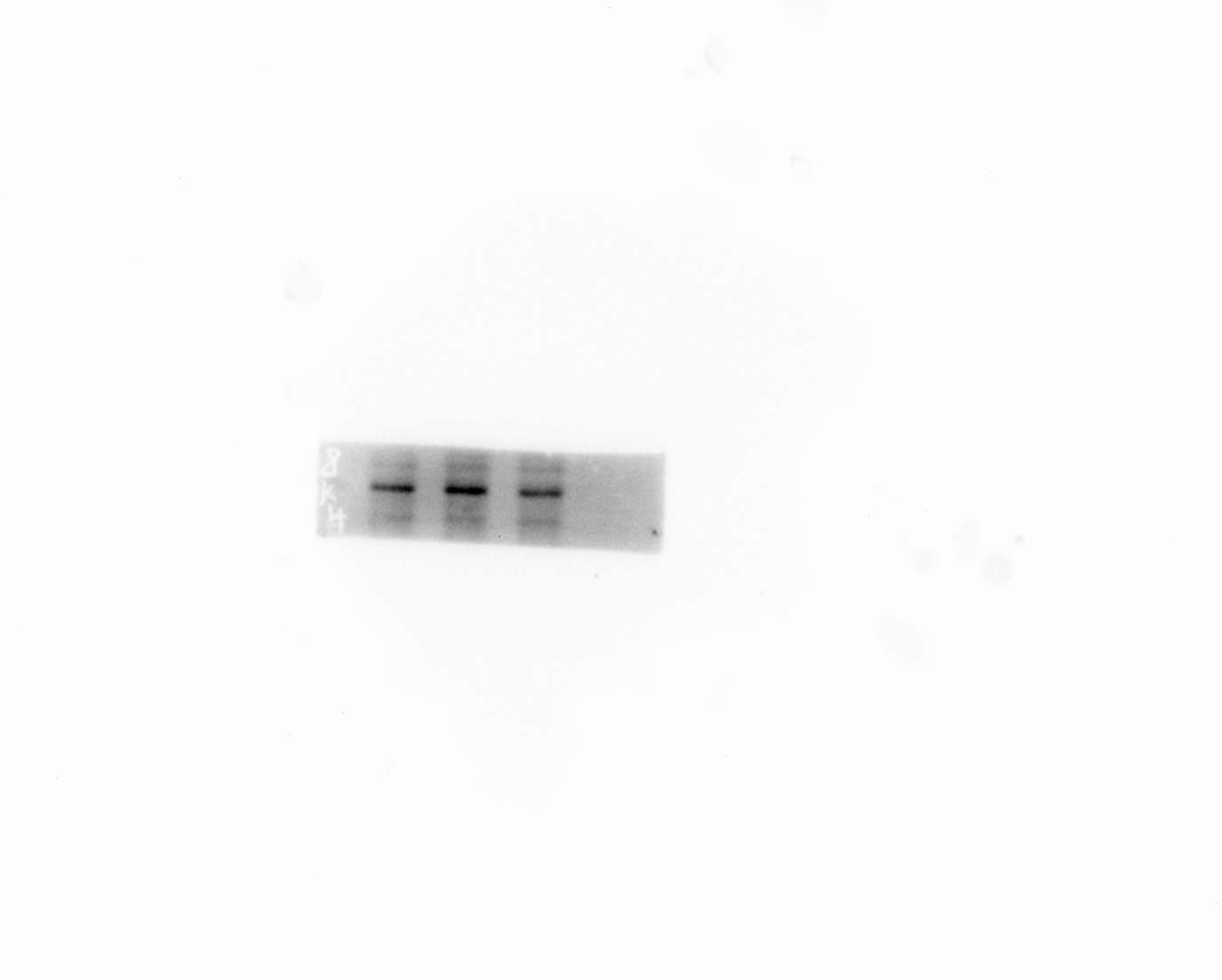

Supplement: Supplementary file 3 [file DataSheet1.ZIP › WB/8226/6cdk4/9.23/8 CDK 4.Tif]

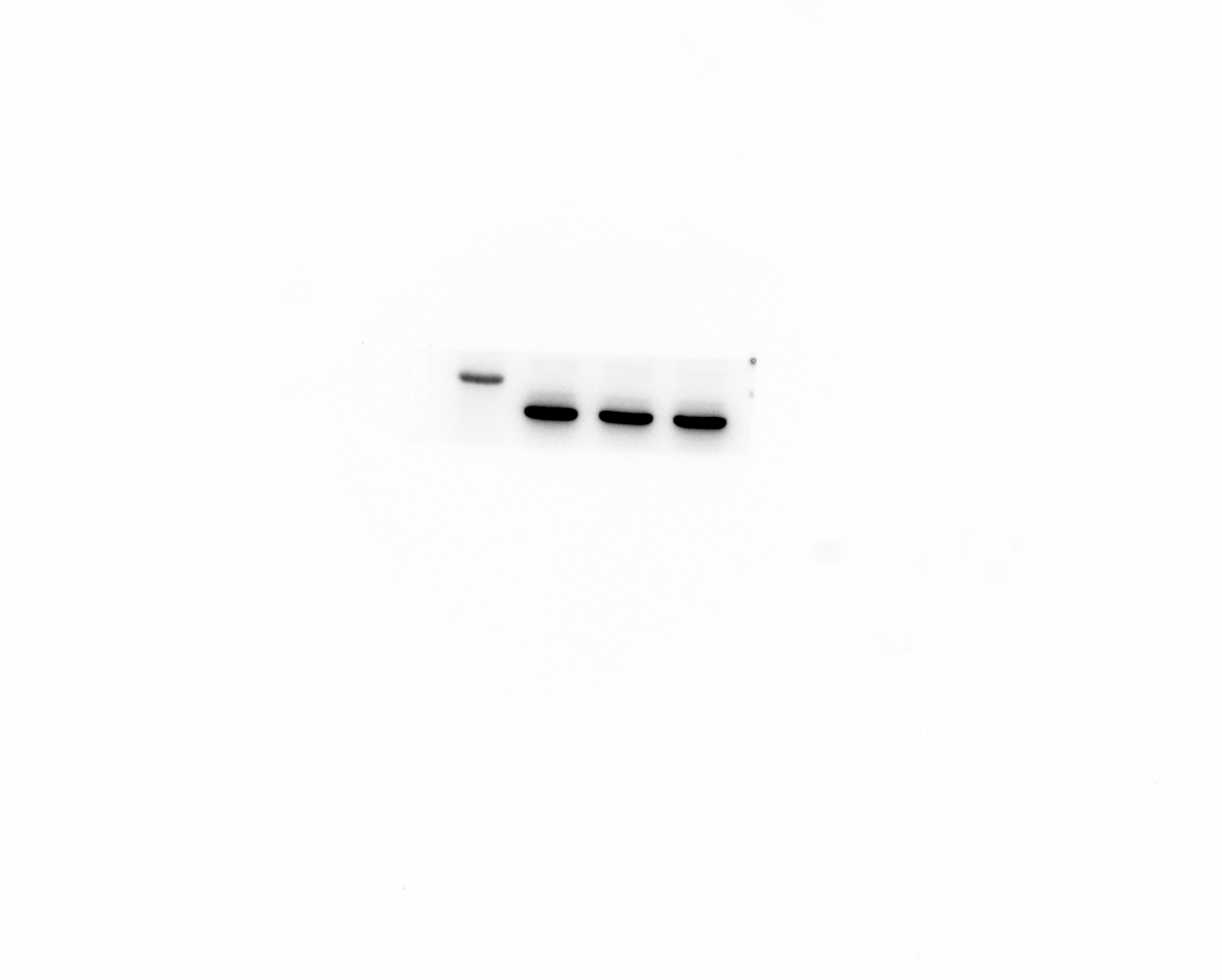

Supplement: Supplementary file 3 [file DataSheet1.ZIP › WB/8226/6cdk4/9.23/8a┬-actin.Tif]

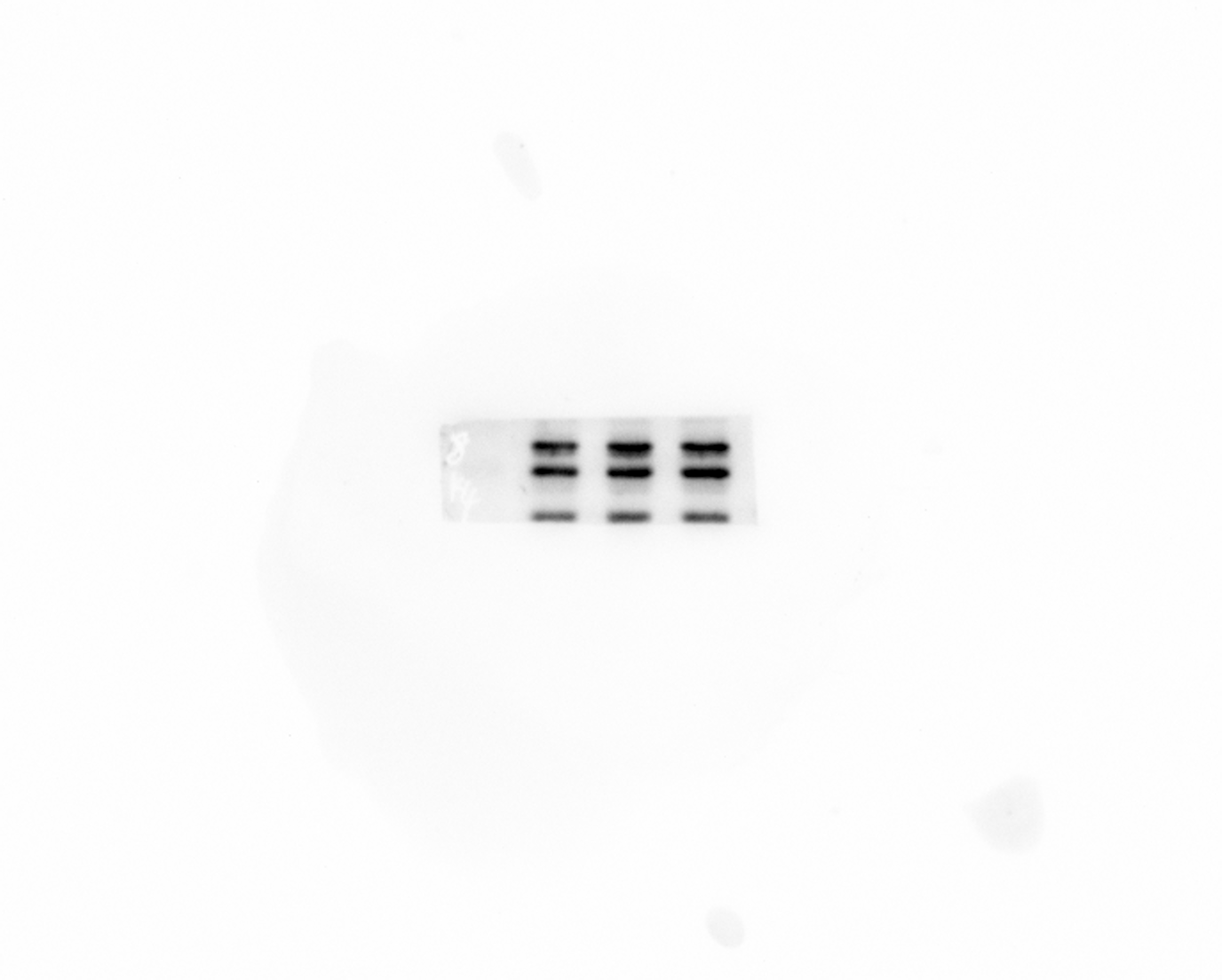

Supplement: Supplementary file 3 [file DataSheet1.ZIP › WB/8226/6cdk4/9.24/8CDK4.Tif]

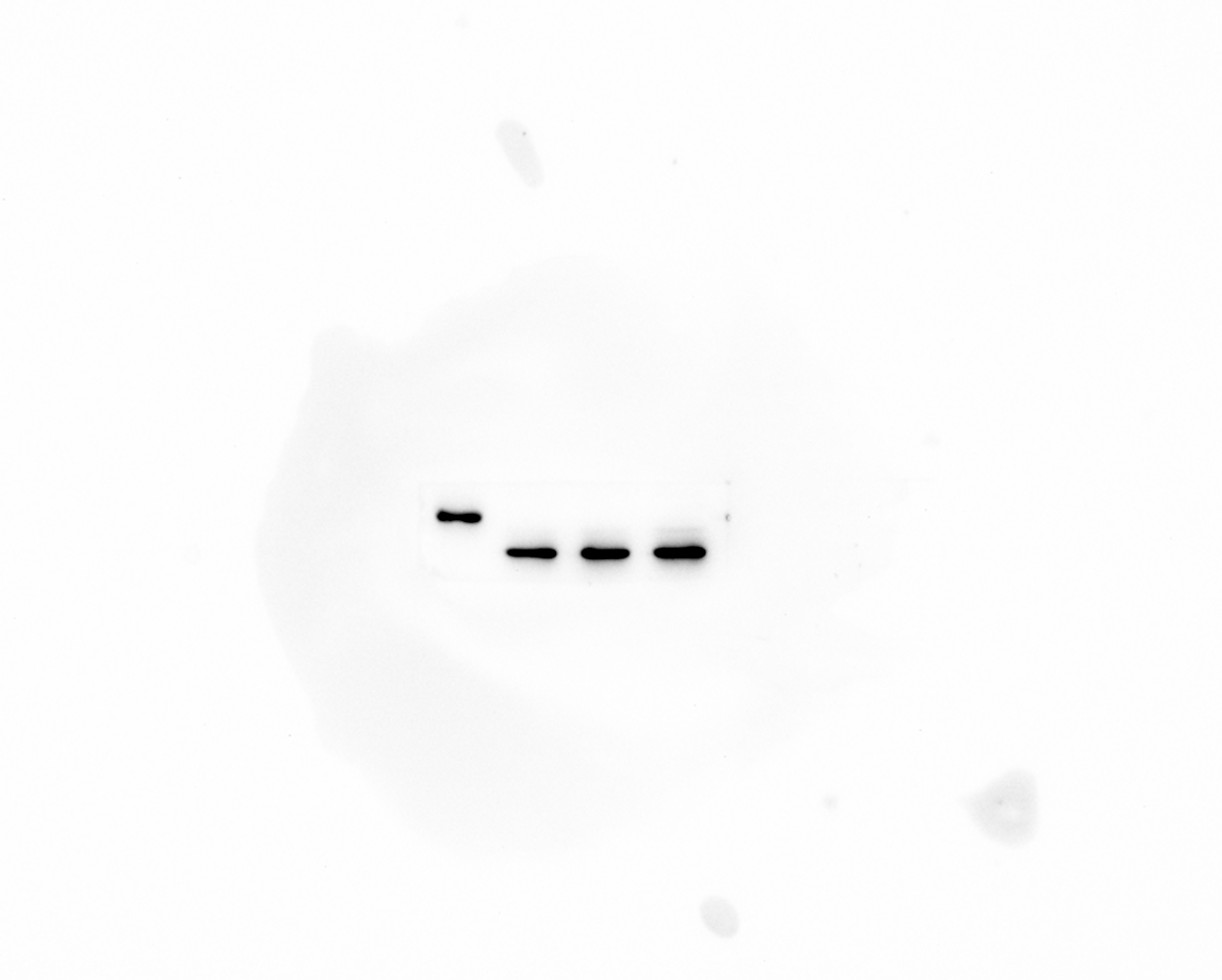

Supplement: Supplementary file 3 [file DataSheet1.ZIP › WB/8226/6cdk4/9.24/8a┬-actin.Tif]

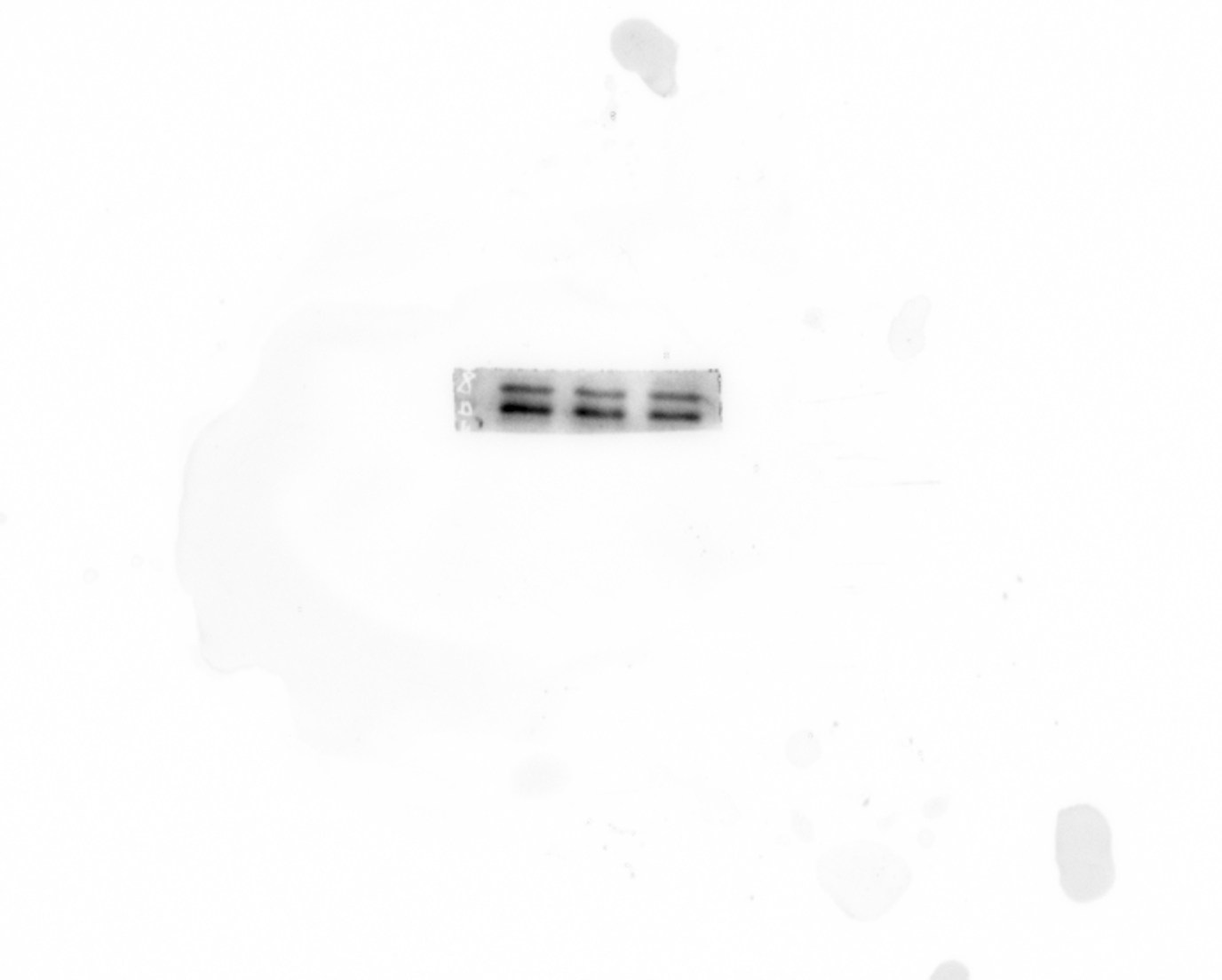

Supplement: Supplementary file 3 [file DataSheet1.ZIP › WB/8226/7P-CDK1/10.1/8 CDK1.Tif]

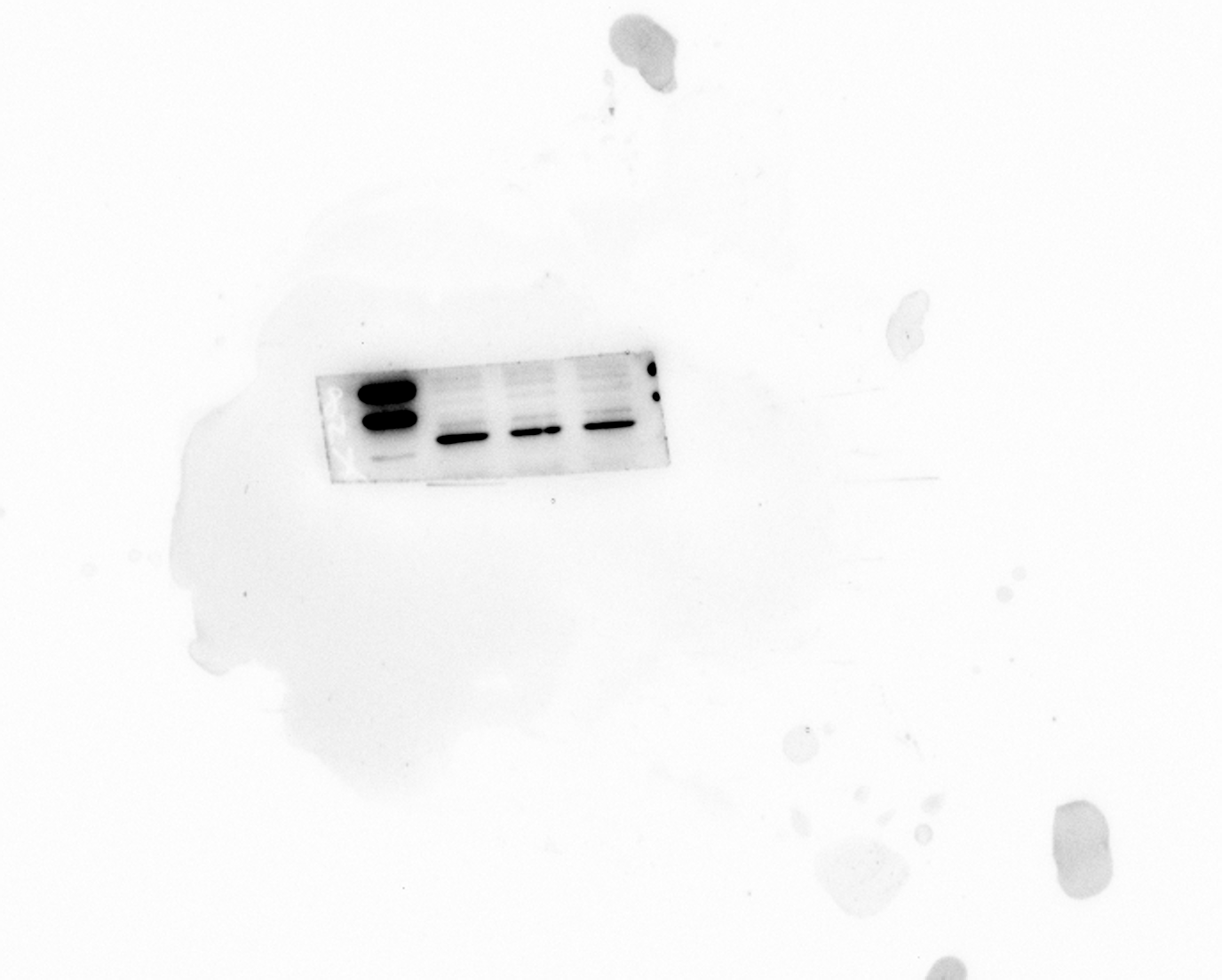

Supplement: Supplementary file 3 [file DataSheet1.ZIP › WB/8226/7P-CDK1/10.1/8 a┬-actin.Tif]

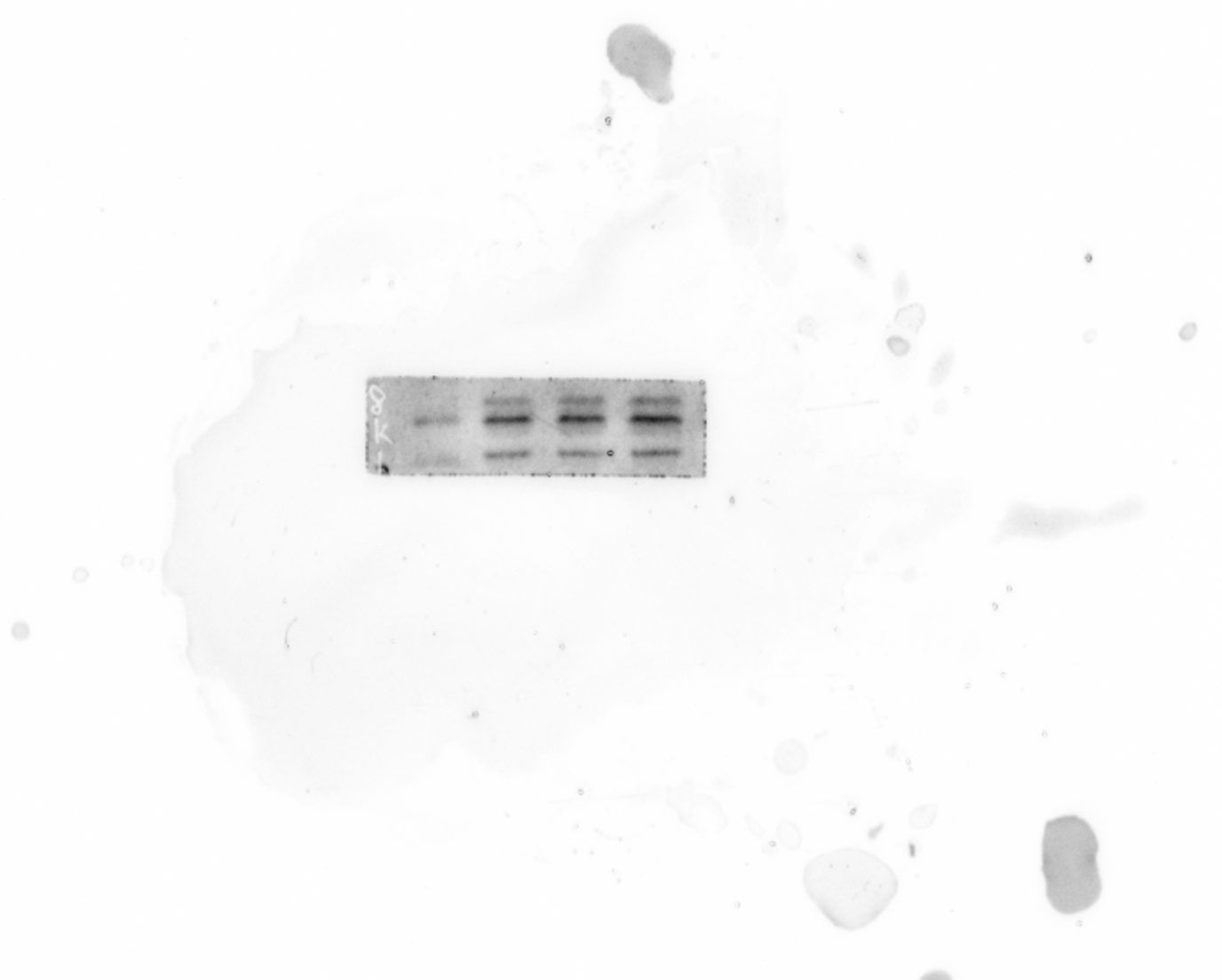

Supplement: Supplementary file 3 [file DataSheet1.ZIP › WB/8226/7P-CDK1/9.28/8CDK1 1.Tif]

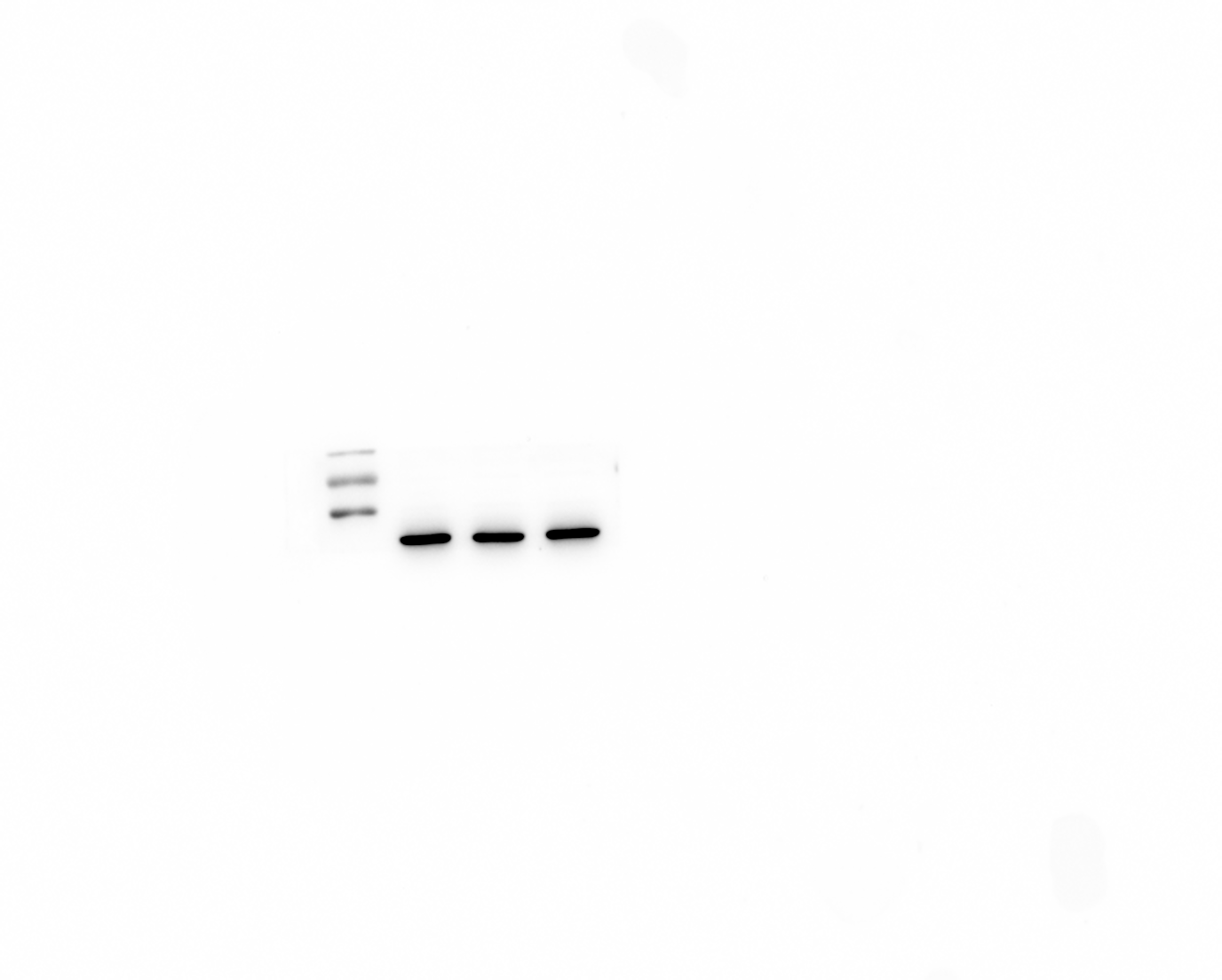

Supplement: Supplementary file 3 [file DataSheet1.ZIP › WB/8226/7P-CDK1/9.28/a┬-actin.Tif]

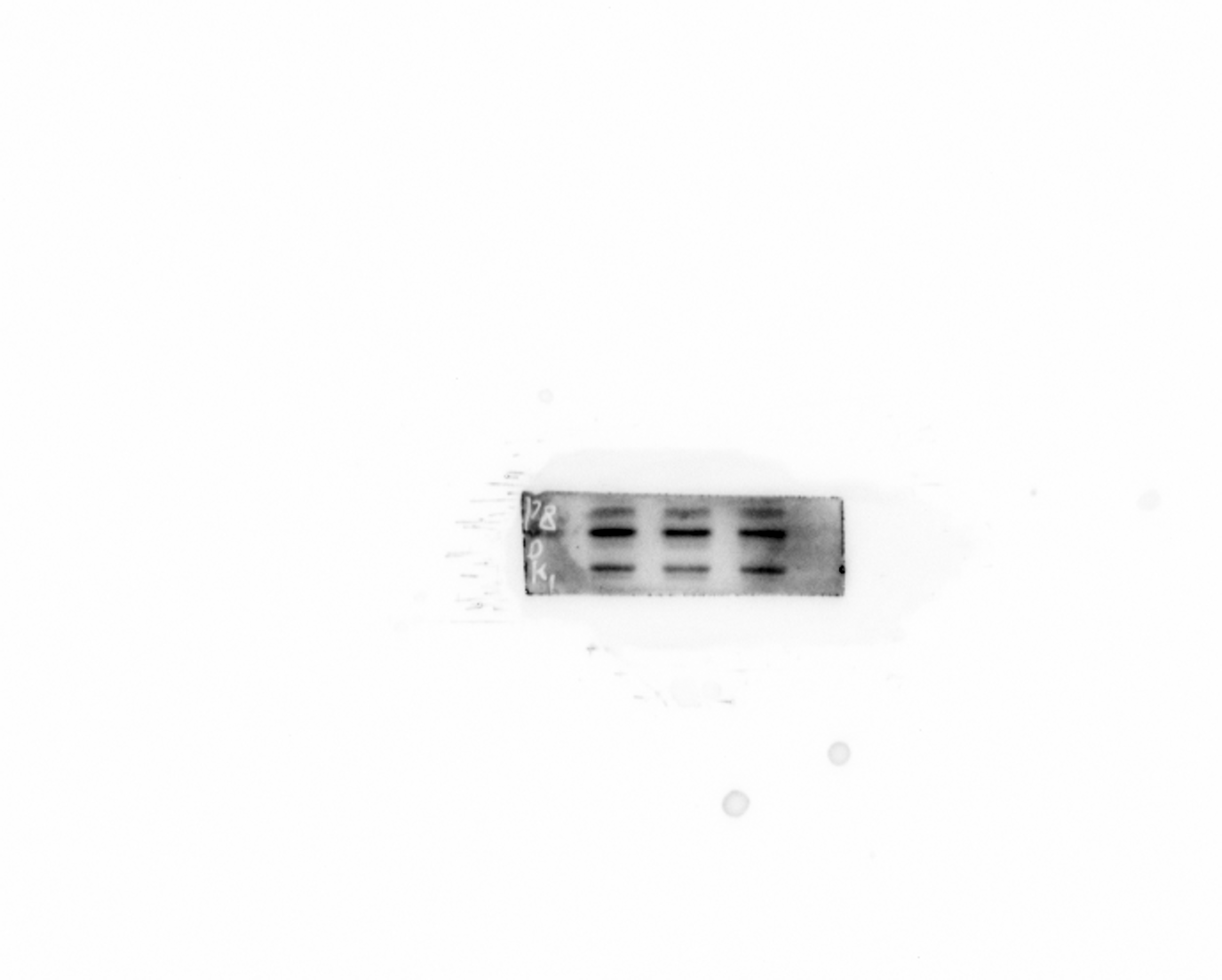

Supplement: Supplementary file 3 [file DataSheet1.ZIP › WB/8226/7P-CDK1/9.29/8 PCDK1.Tif]

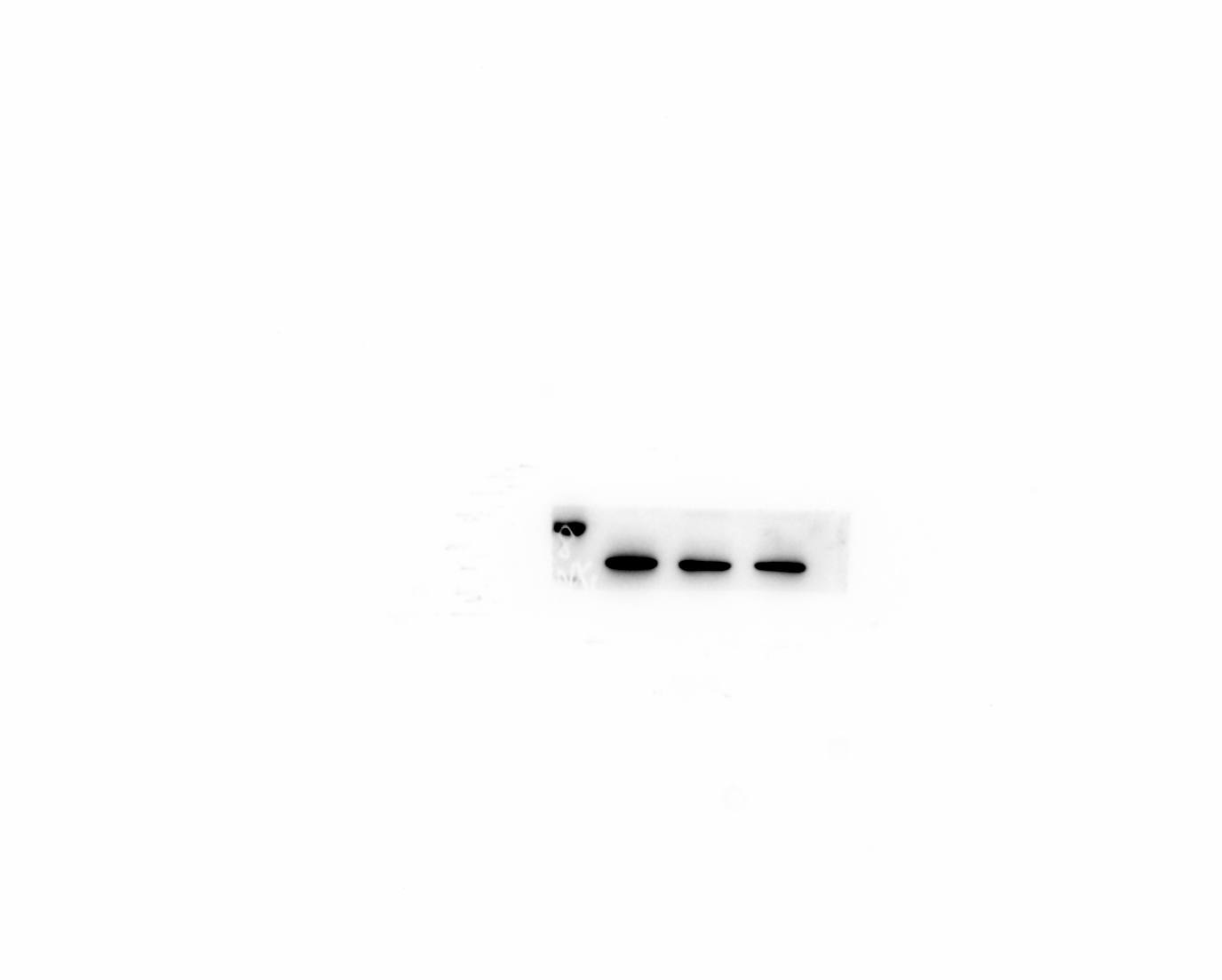

Supplement: Supplementary file 3 [file DataSheet1.ZIP › WB/8226/7P-CDK1/9.29/8a┬-actin.Tif]

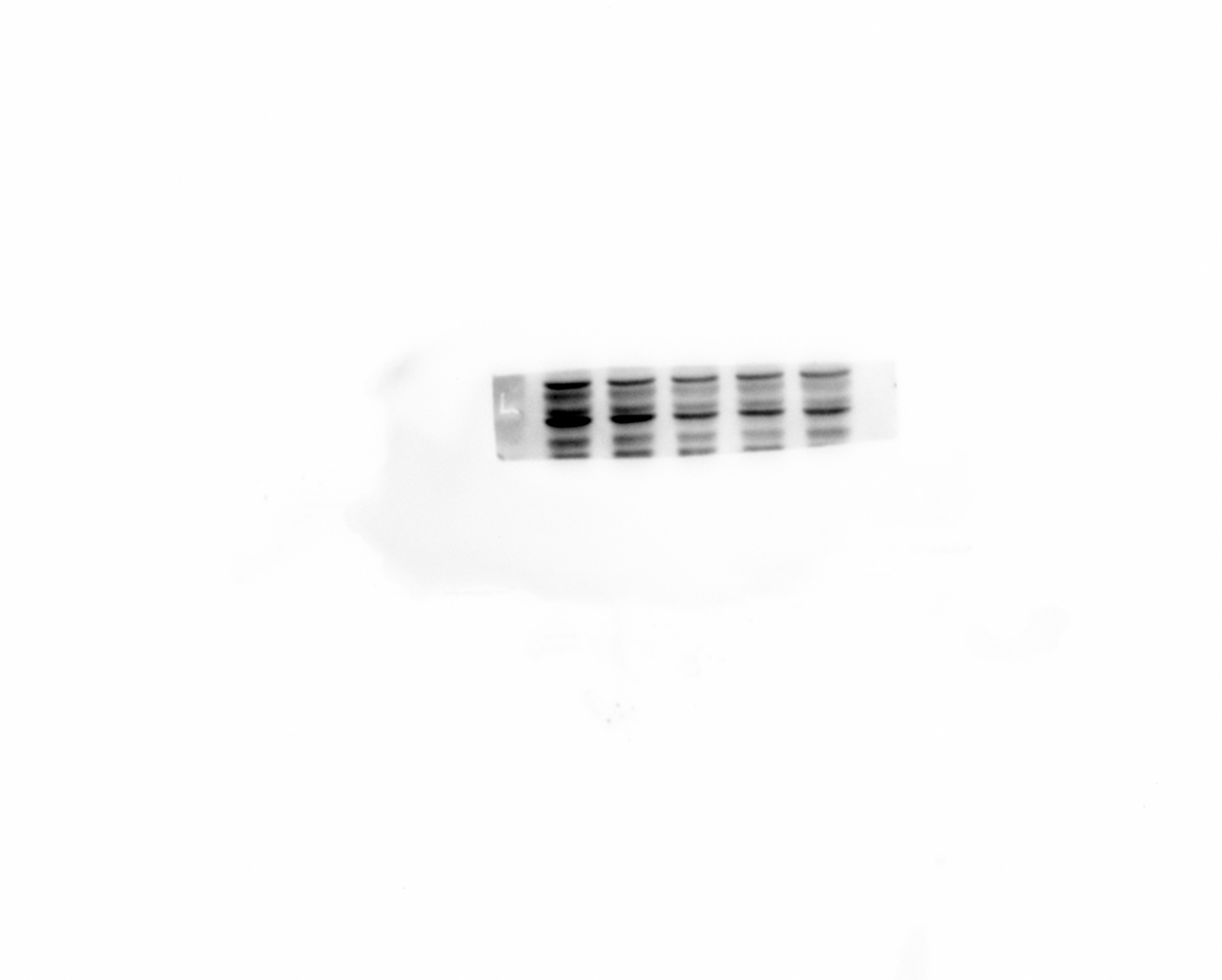

Supplement: Supplementary file 3 [file DataSheet1.ZIP › WB/8226/8LAMP5 sirna/9.12/LAMP5.Tif]

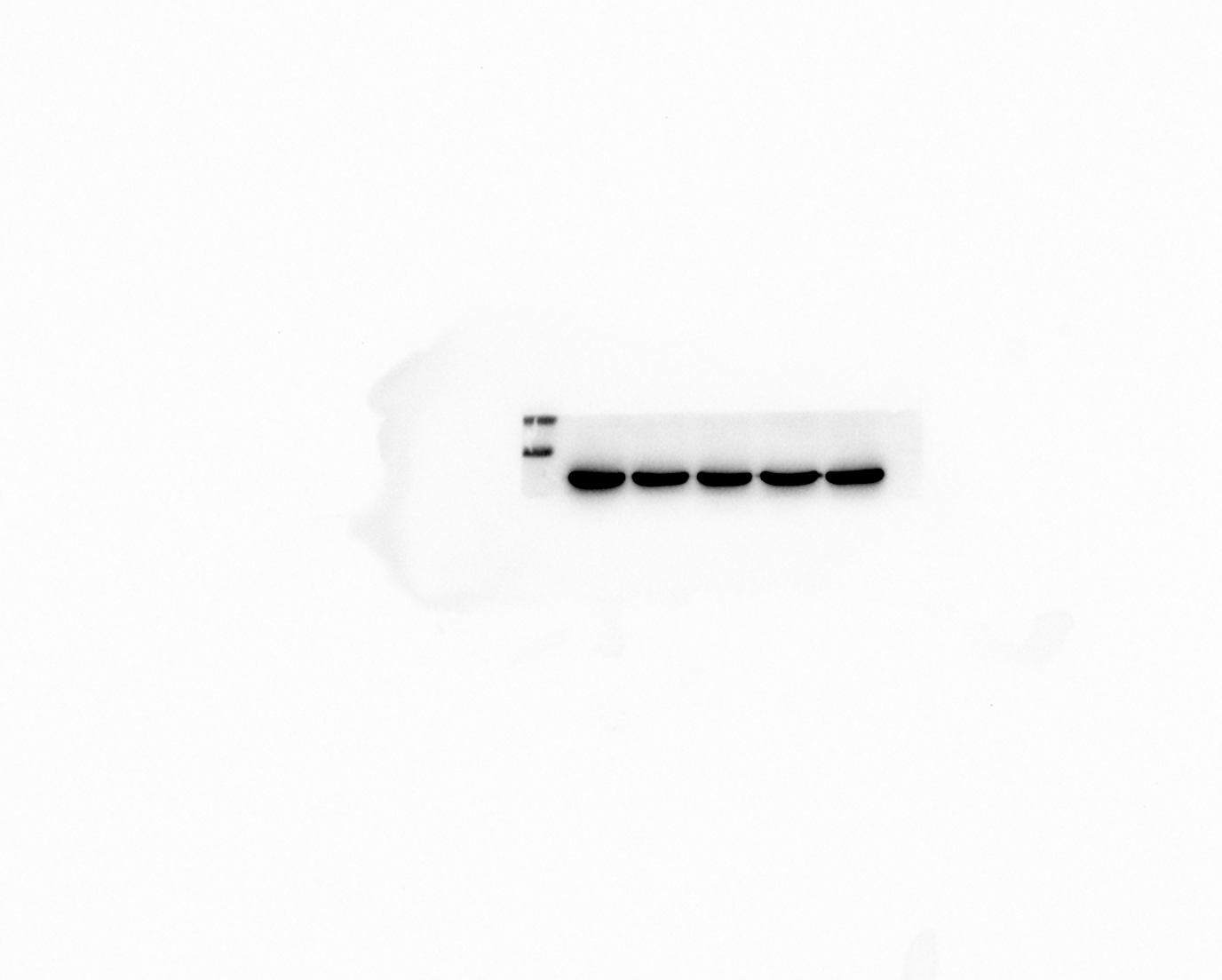

Supplement: Supplementary file 3 [file DataSheet1.ZIP › WB/8226/8LAMP5 sirna/9.12/a┬-actin.Tif]

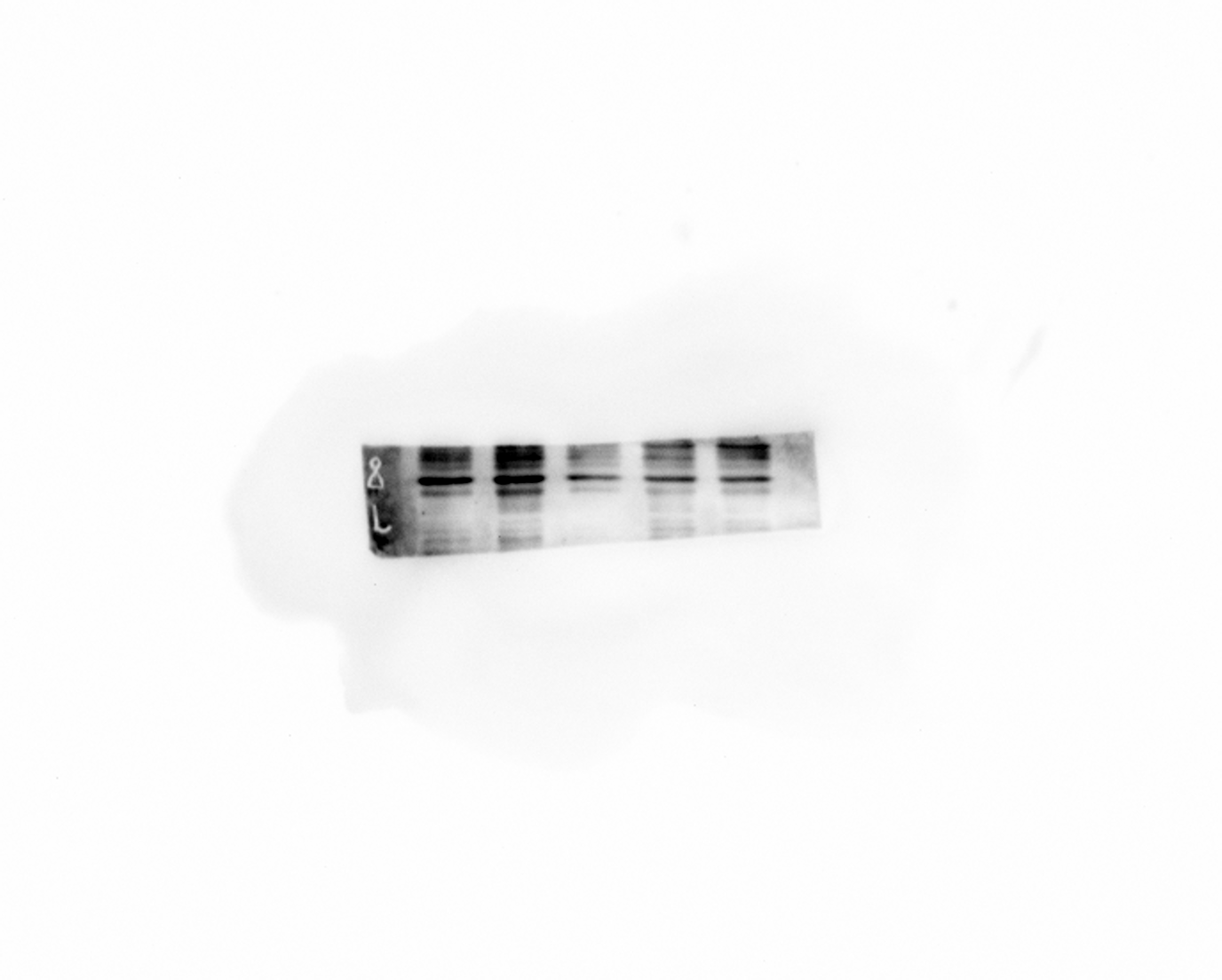

Supplement: Supplementary file 3 [file DataSheet1.ZIP › WB/8226/8LAMP5 sirna/9.17/LAMP5.Tif]

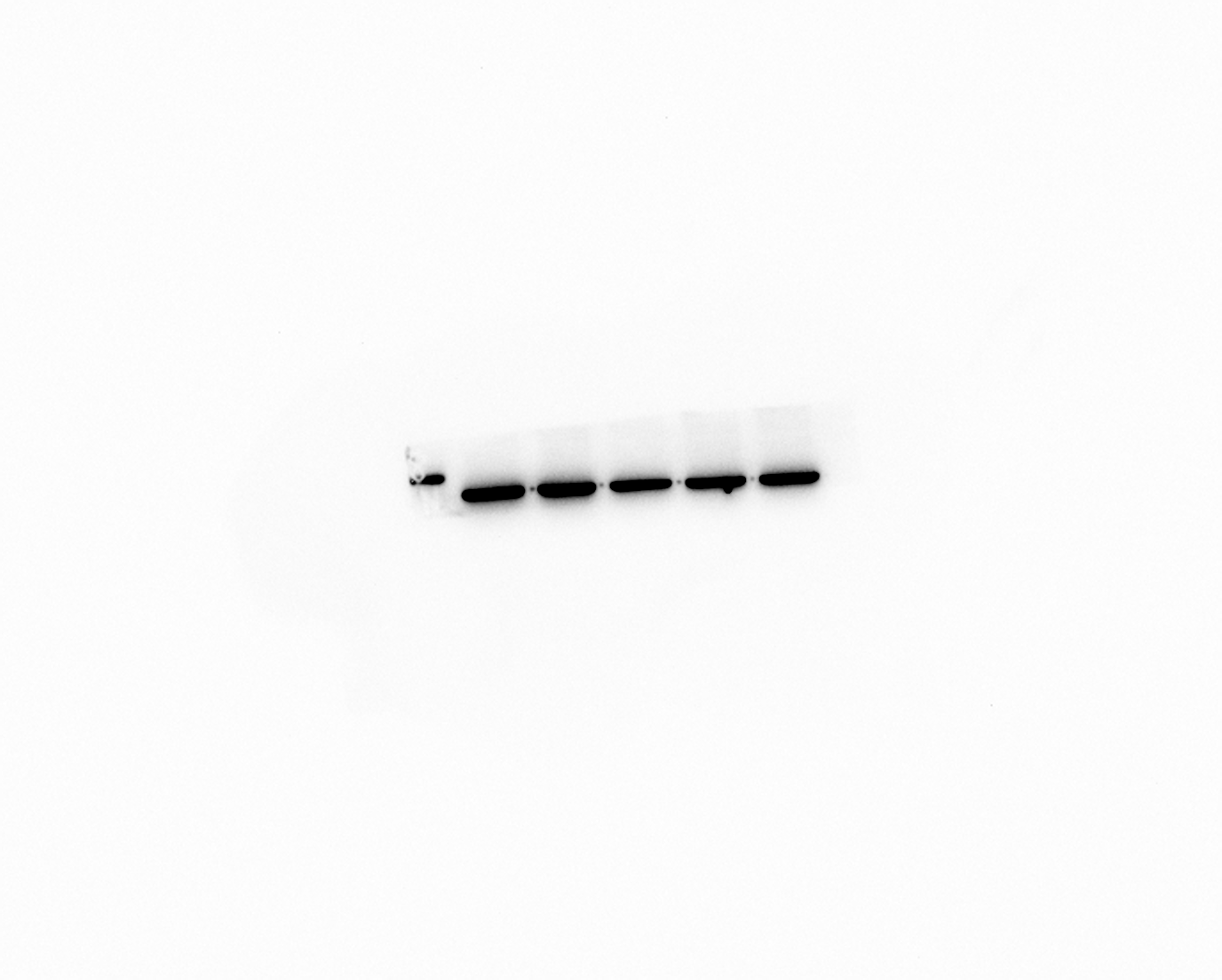

Supplement: Supplementary file 3 [file DataSheet1.ZIP › WB/8226/8LAMP5 sirna/9.17/a┬-actin.Tif]

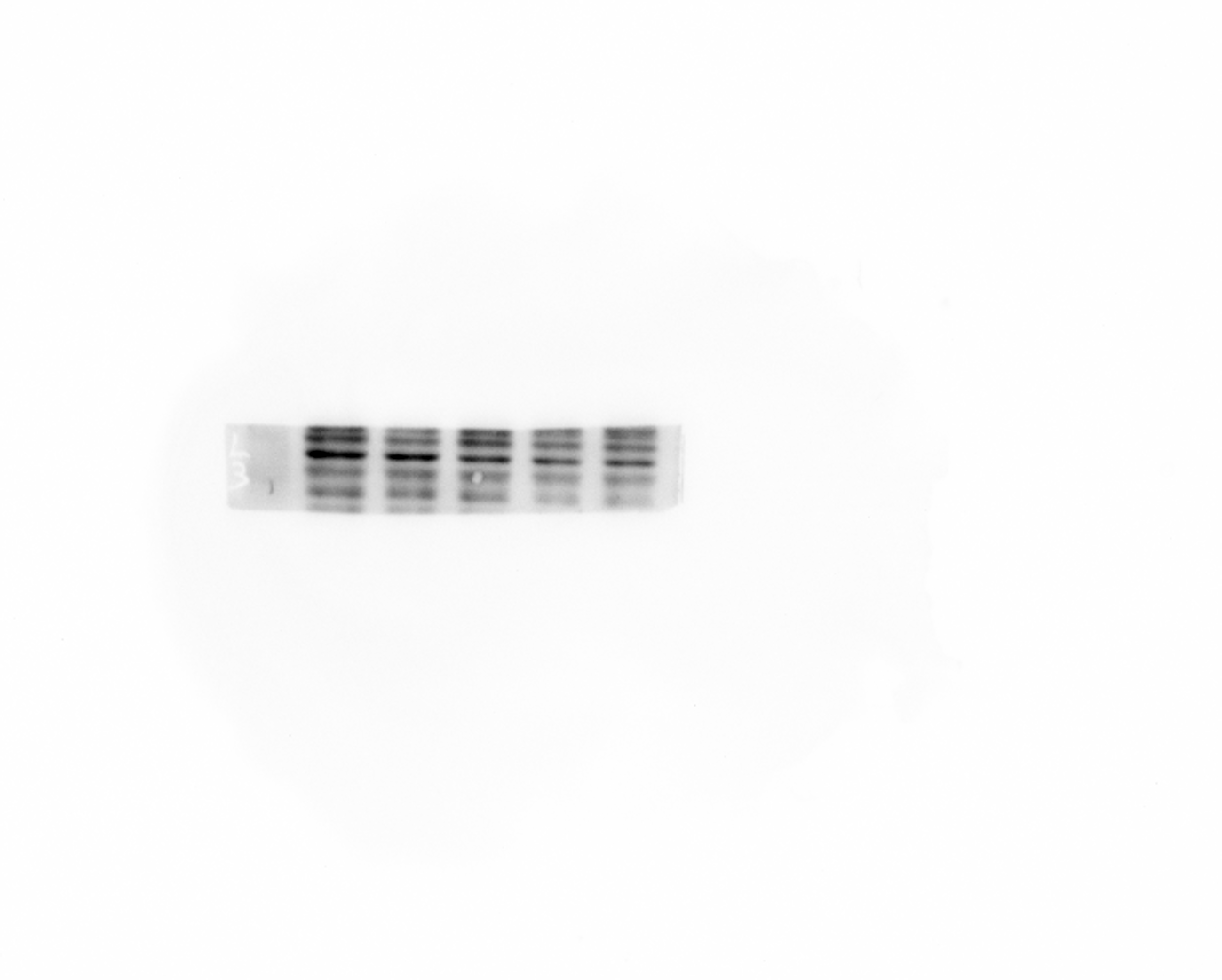

Supplement: Supplementary file 3 [file DataSheet1.ZIP › WB/8226/8LAMP5 sirna/9.21/LAMP5.Tif]

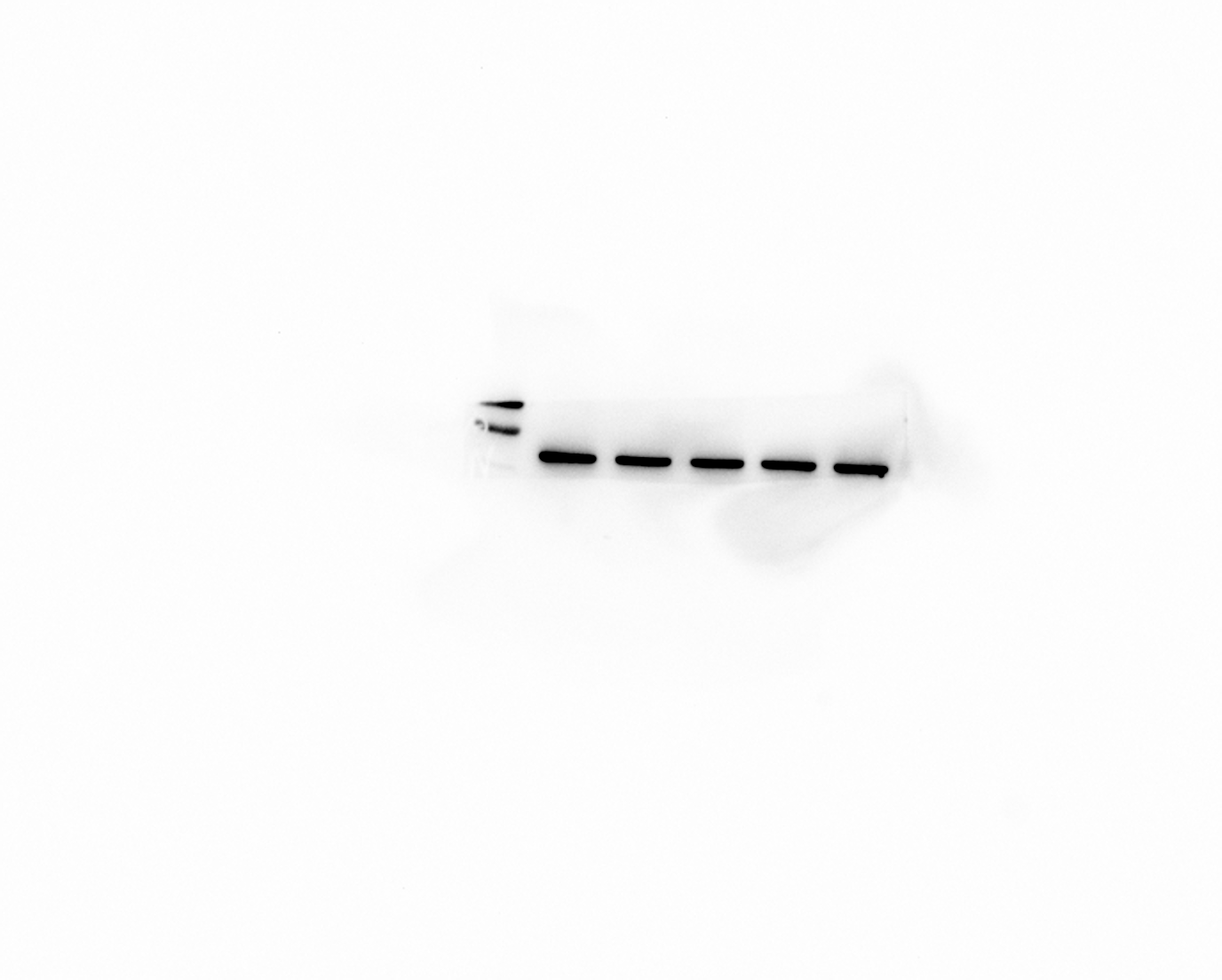

Supplement: Supplementary file 3 [file DataSheet1.ZIP › WB/8226/8LAMP5 sirna/9.21/a┬-actin.Tif]

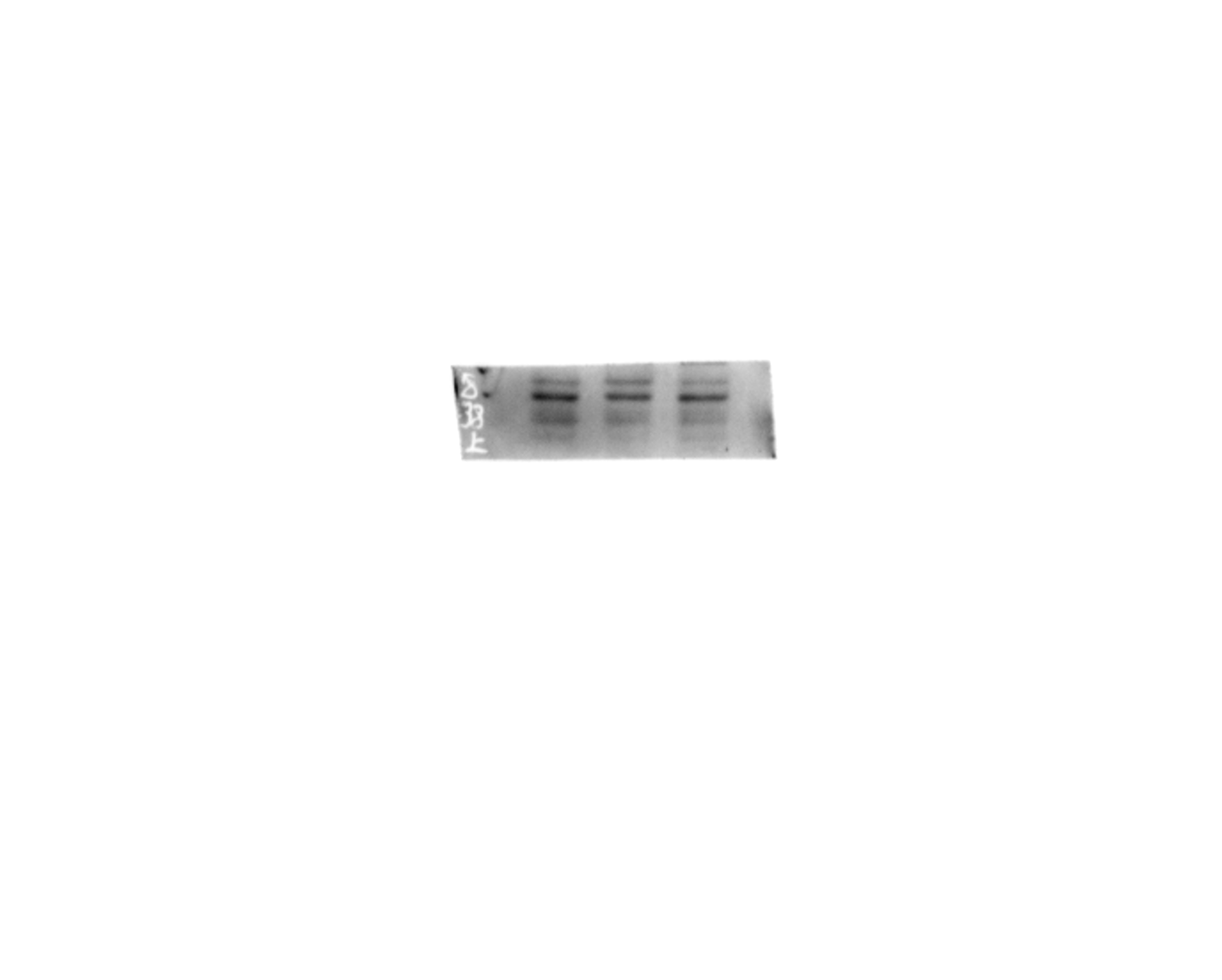

Supplement: Supplementary file 3 [file DataSheet1.ZIP › WB/8226/9 p-38/10.1/8 P38.Tif]

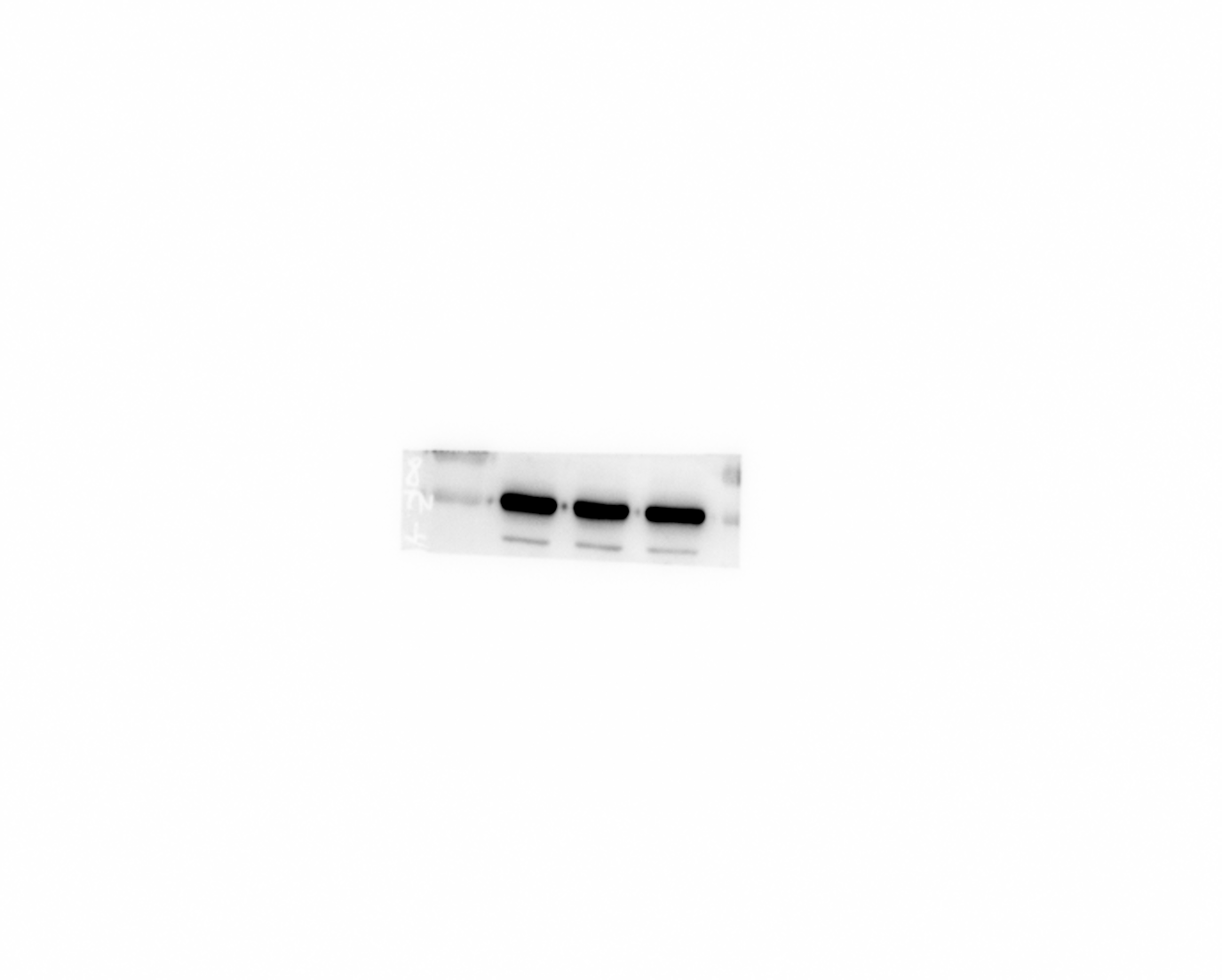

Supplement: Supplementary file 3 [file DataSheet1.ZIP › WB/8226/9 p-38/10.1/8 a┬-Tubulin.Tif]

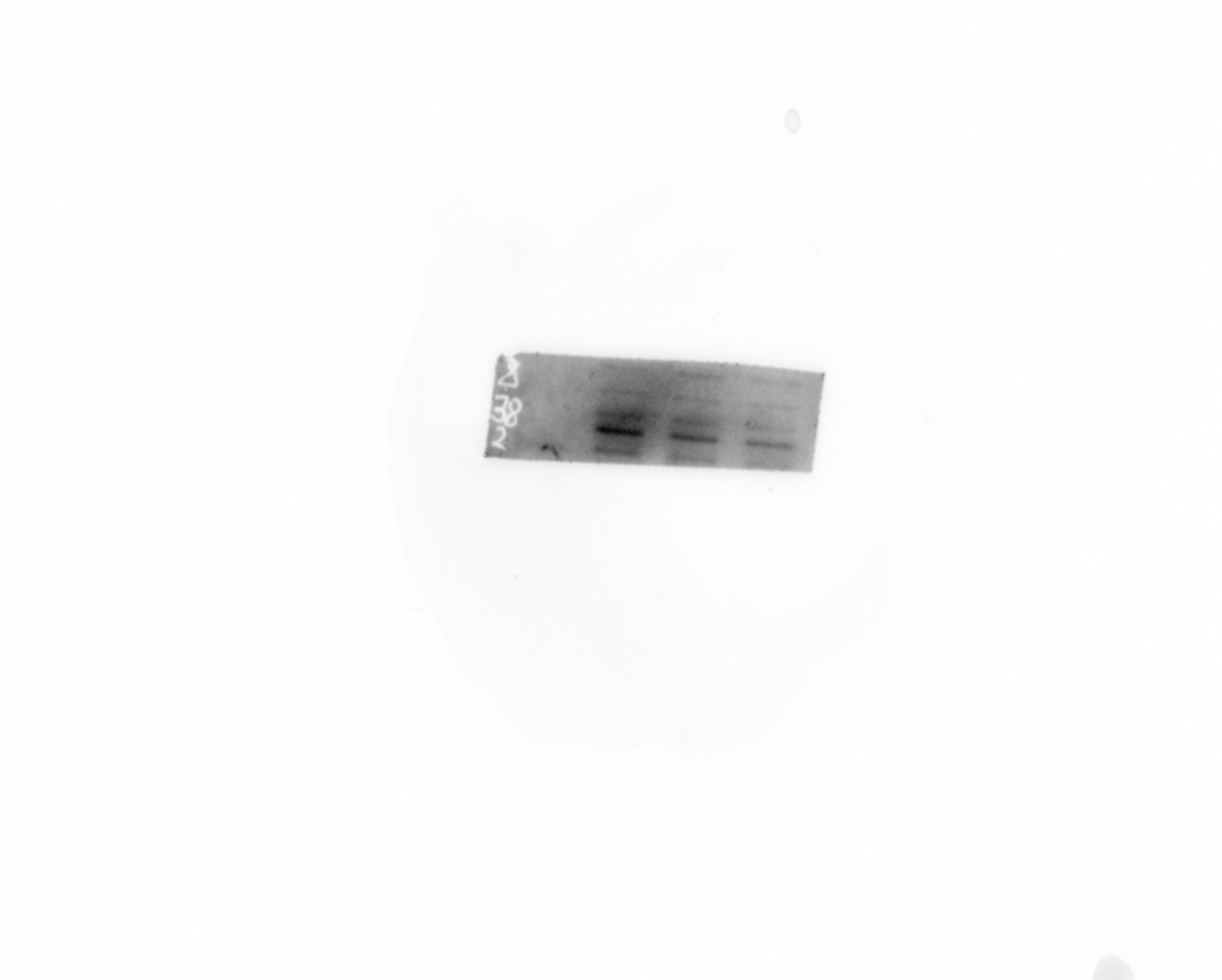

Supplement: Supplementary file 3 [file DataSheet1.ZIP › WB/8226/9 p-38/10.3/8 P 38 2.Tif]

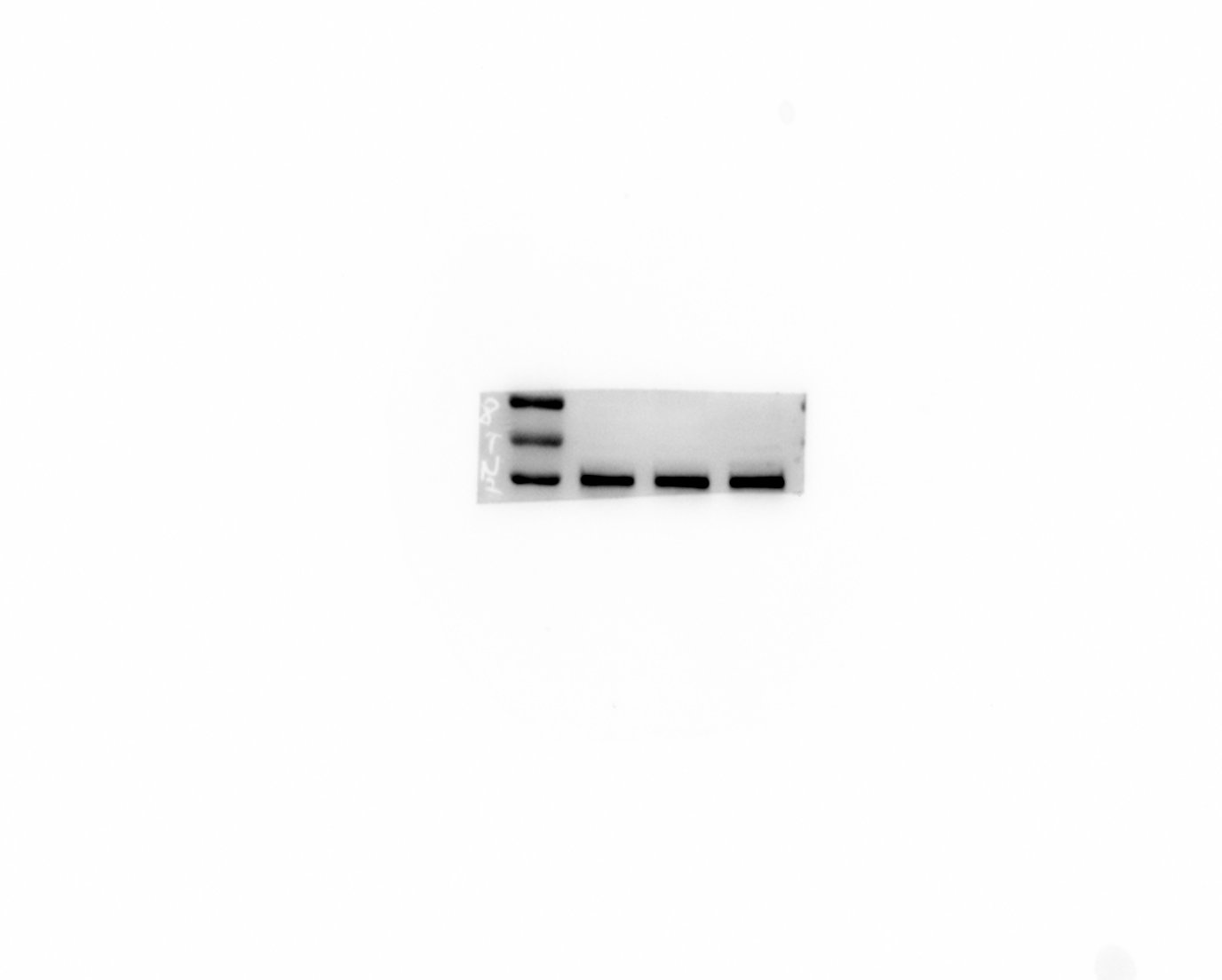

Supplement: Supplementary file 3 [file DataSheet1.ZIP › WB/8226/9 p-38/10.3/8 a┬-Tubulin.Tif]

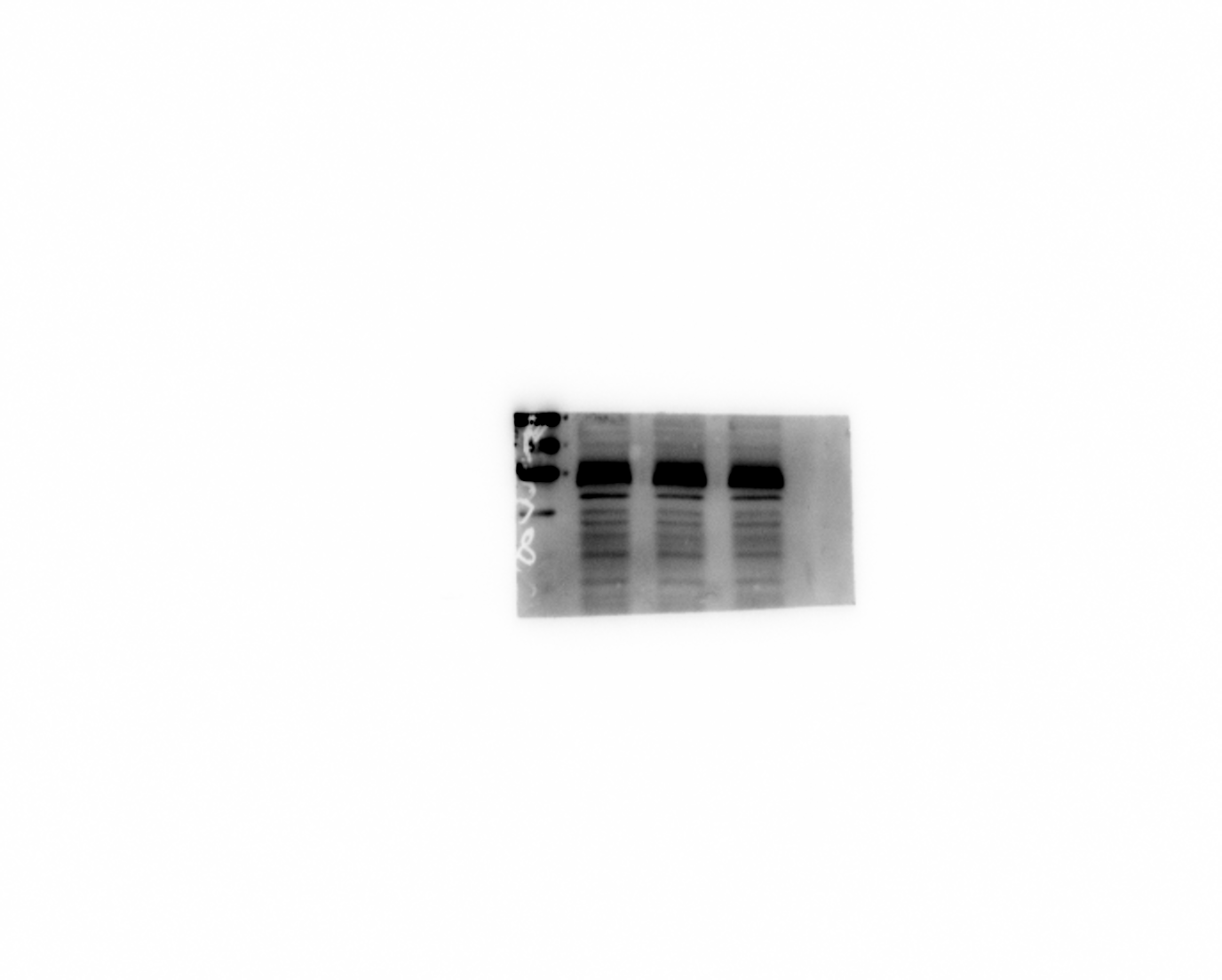

Supplement: Supplementary file 3 [file DataSheet1.ZIP › WB/8226/9 p-38/10.8/P38.Tif]

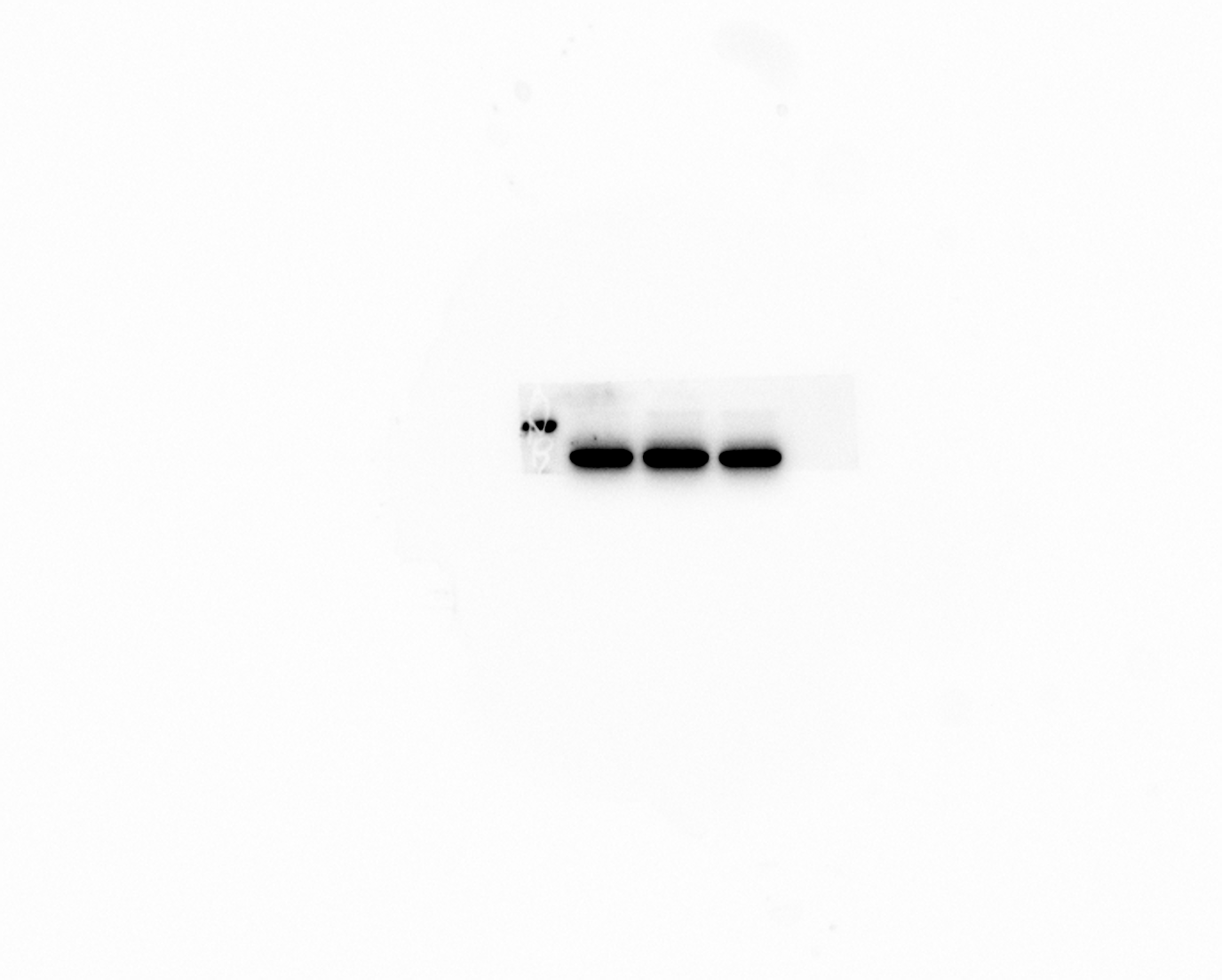

Supplement: Supplementary file 3 [file DataSheet1.ZIP › WB/AMO-1/1 bax/10.1/Aa┬-actin.Tif]

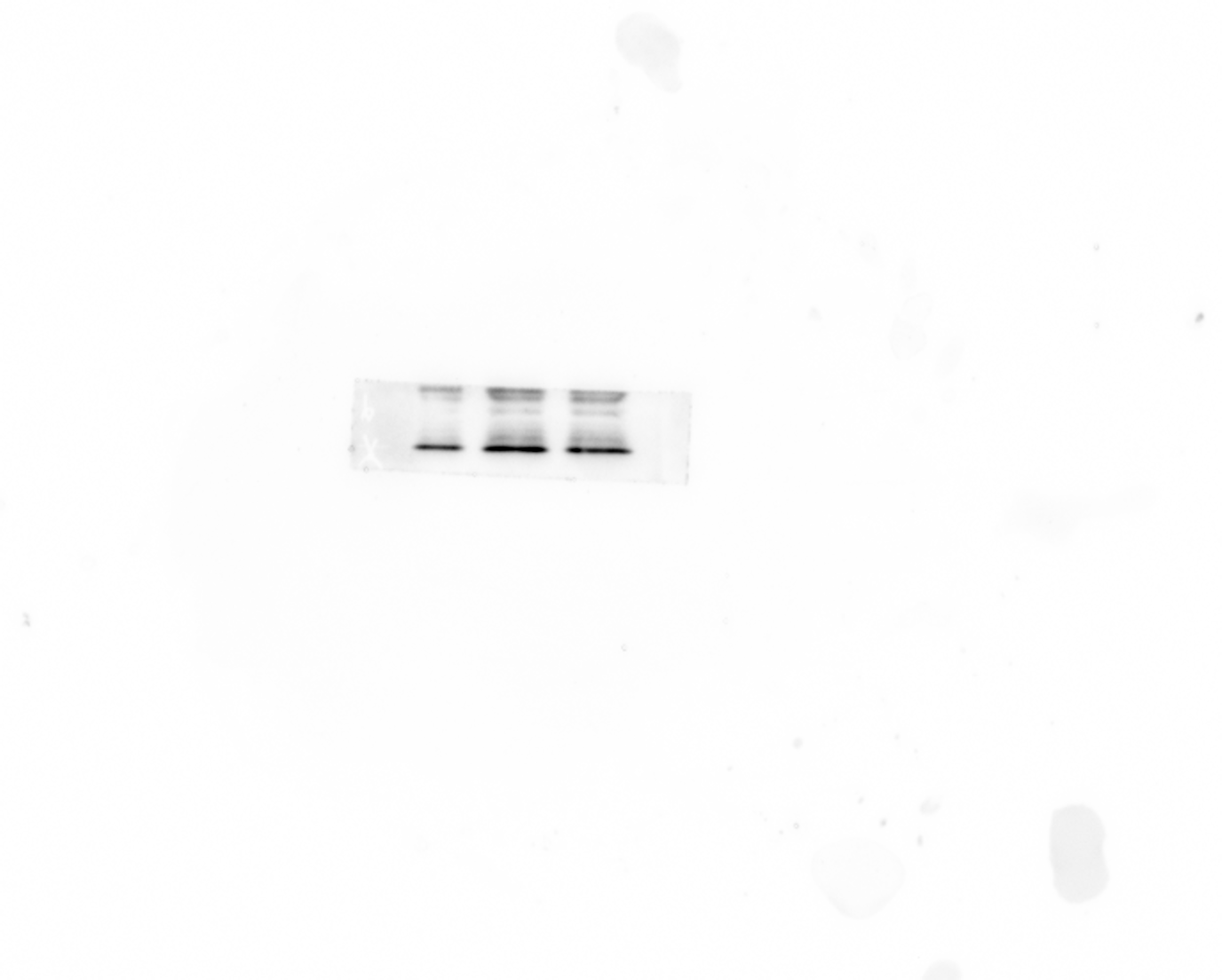

Supplement: Supplementary file 3 [file DataSheet1.ZIP › WB/AMO-1/1 bax/10.1/BAX 1.Tif]

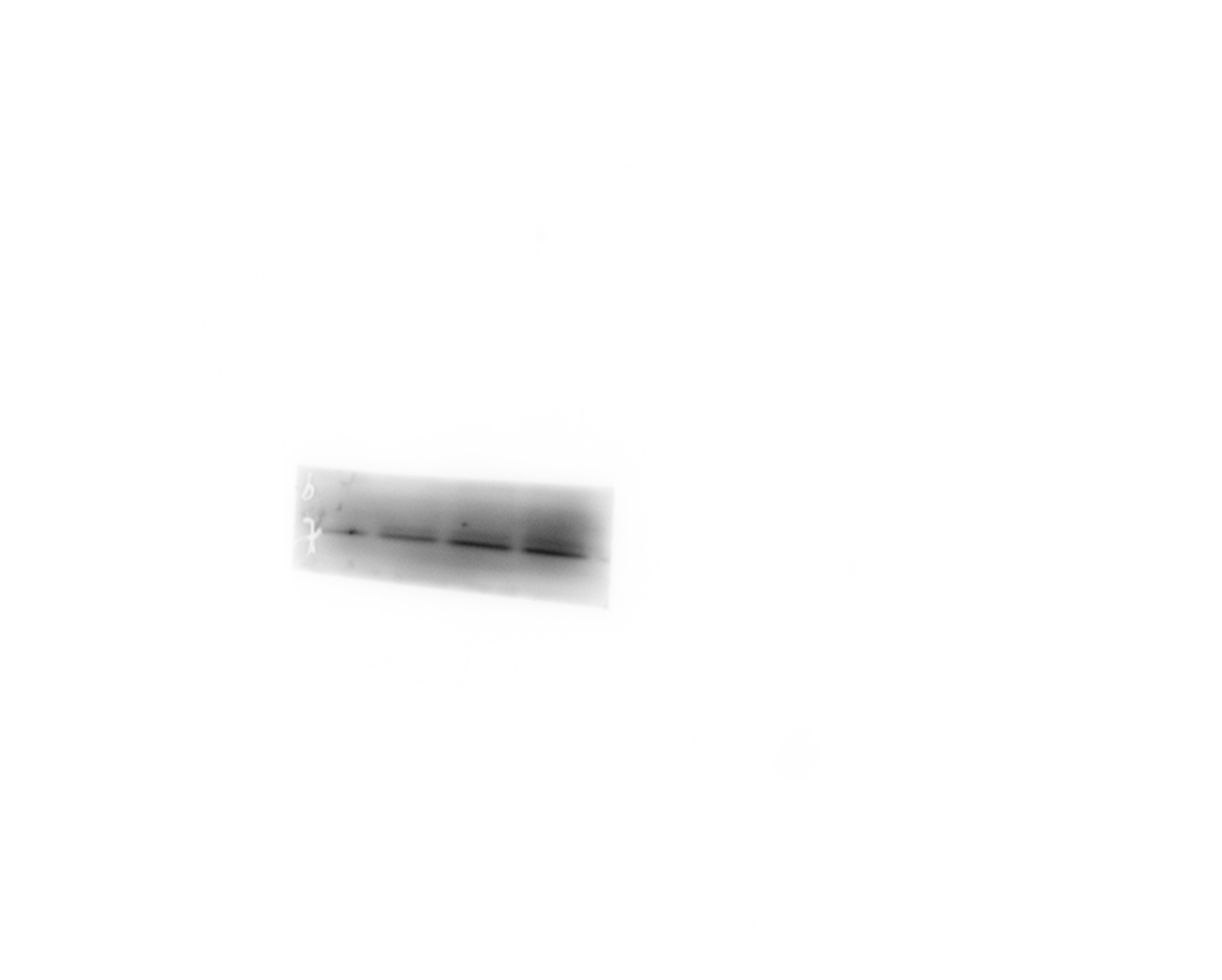

Supplement: Supplementary file 3 [file DataSheet1.ZIP › WB/AMO-1/1 bax/9.26/BAX 2.Tif]

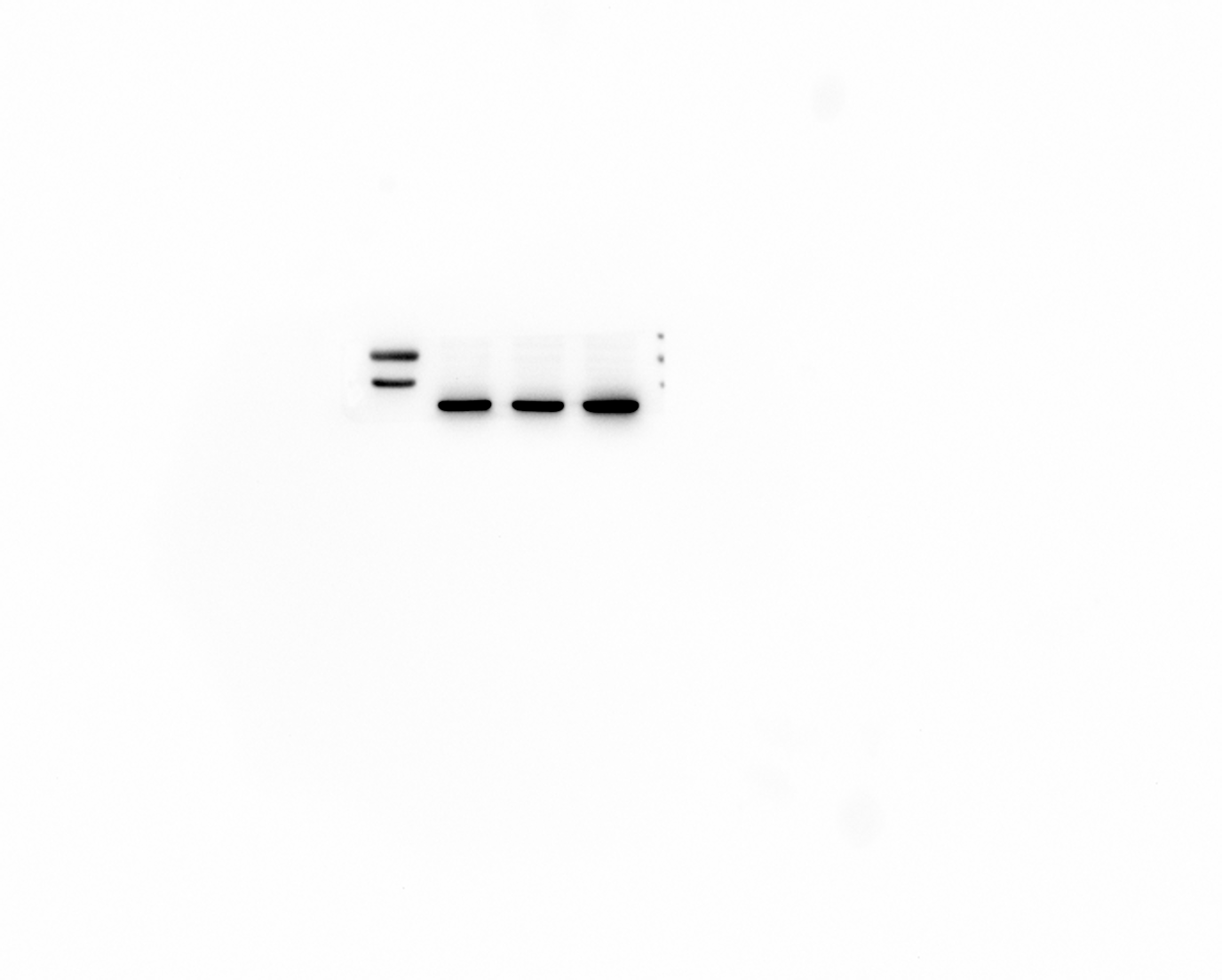

Supplement: Supplementary file 3 [file DataSheet1.ZIP › WB/AMO-1/1 bax/9.26/a┬-actin.Tif]

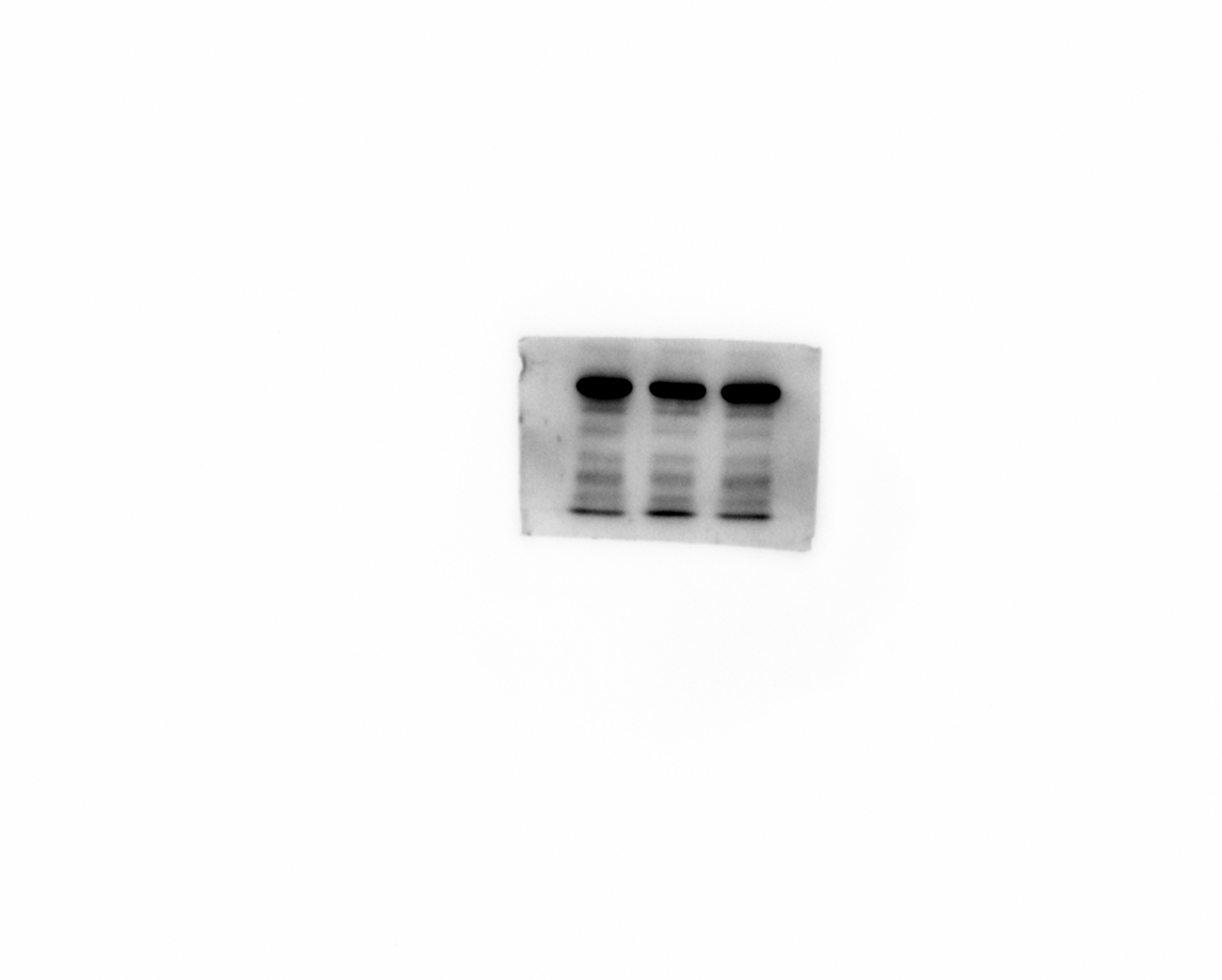

Supplement: Supplementary file 3 [file DataSheet1.ZIP › WB/AMO-1/1 bax/9.30/A BAX 1.Tif]

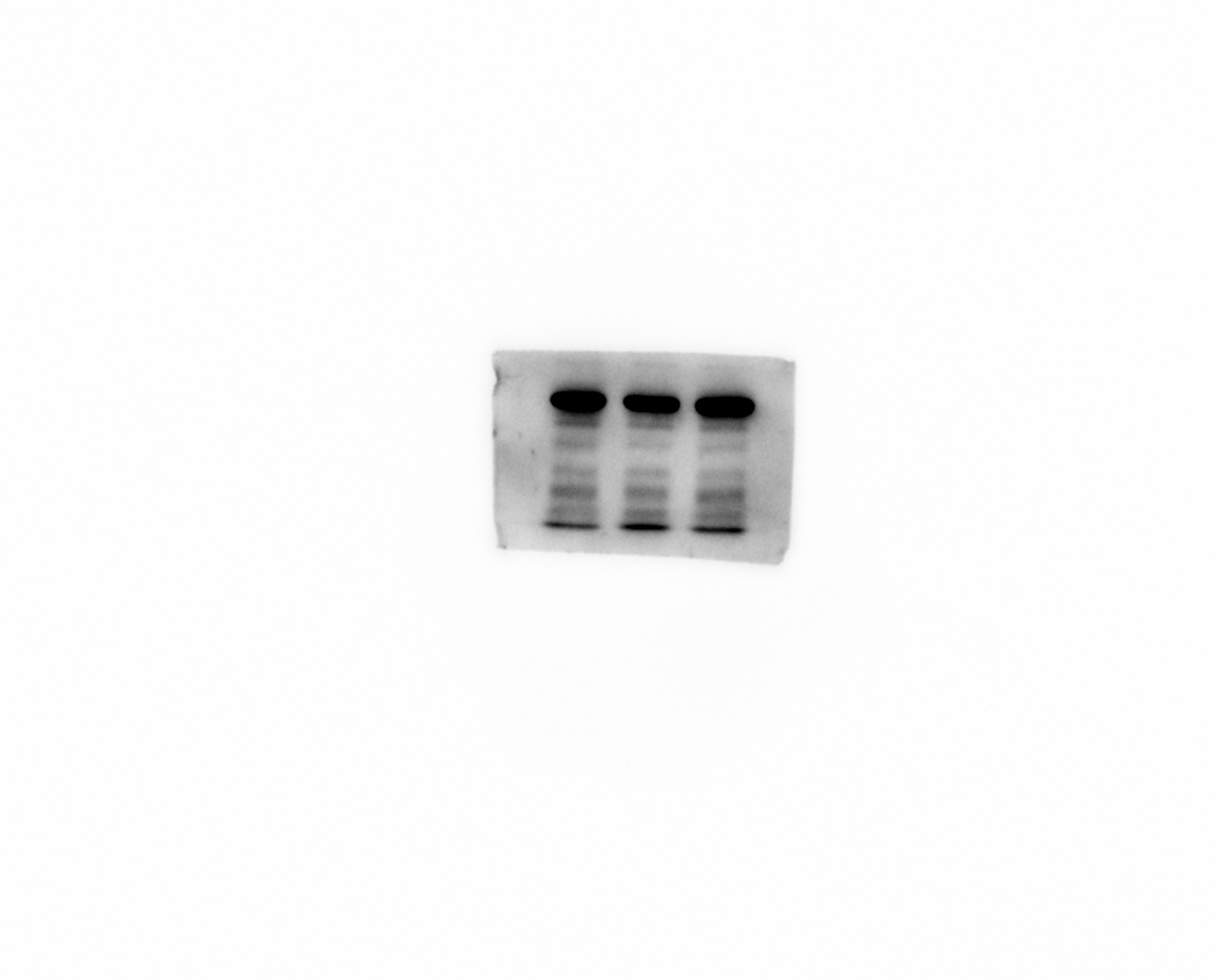

Supplement: Supplementary file 3 [file DataSheet1.ZIP › WB/AMO-1/1 bax/9.30/A BAX.Tif]

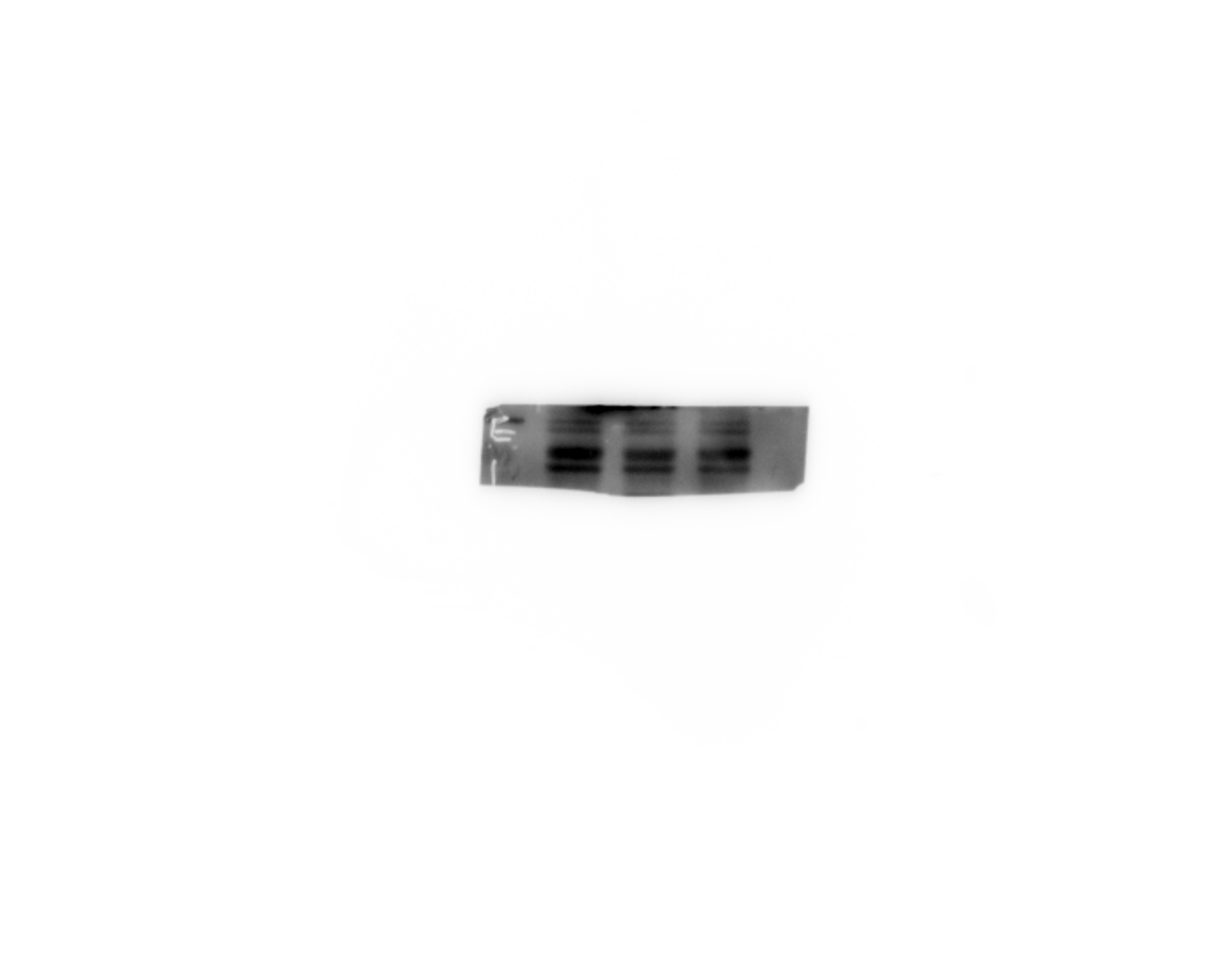

Supplement: Supplementary file 3 [file DataSheet1.ZIP › WB/AMO-1/10 p-erk12/10.10/E1.Tif]

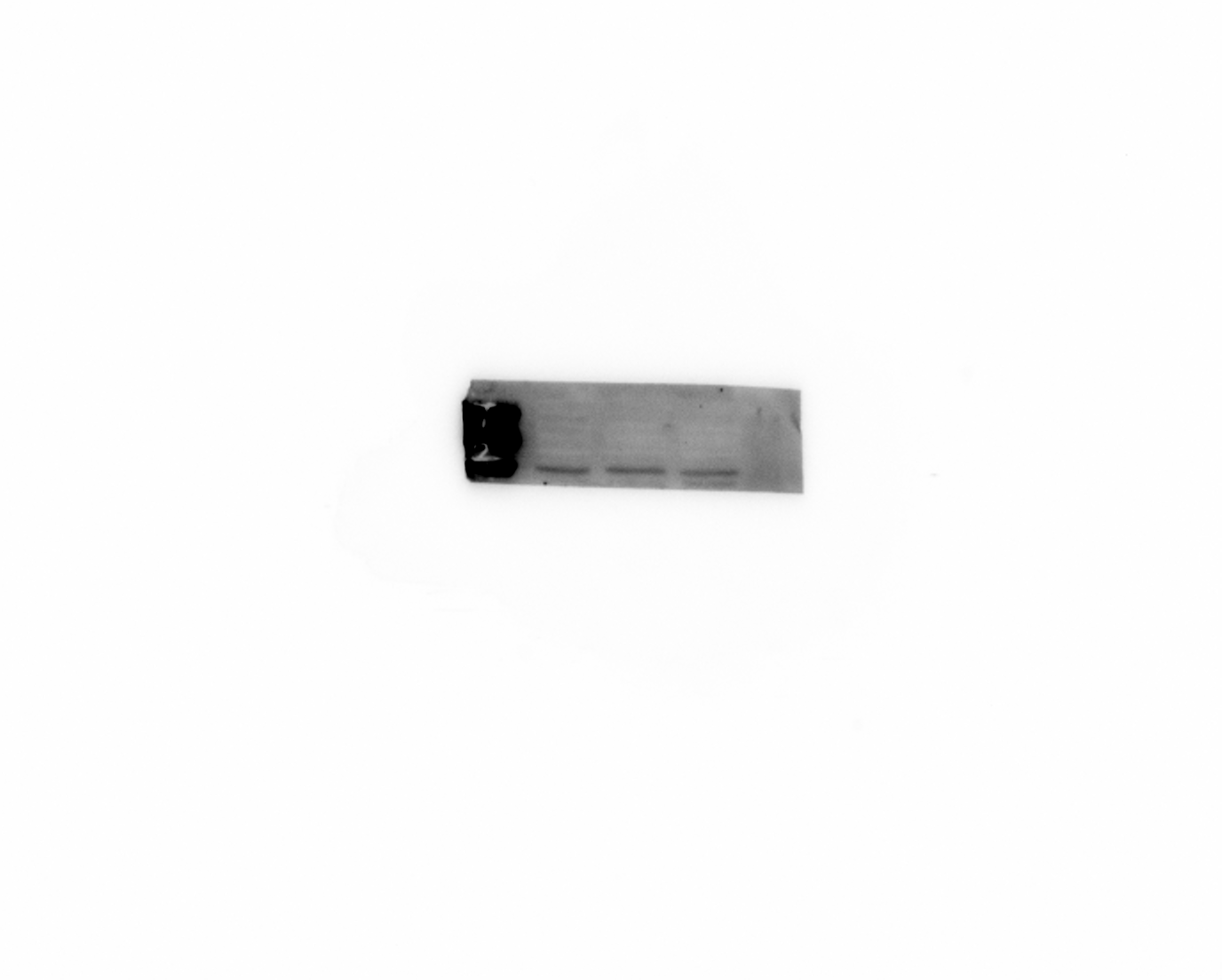

Supplement: Supplementary file 3 [file DataSheet1.ZIP › WB/AMO-1/10 p-erk12/10.10/a┬-tubulin.Tif]

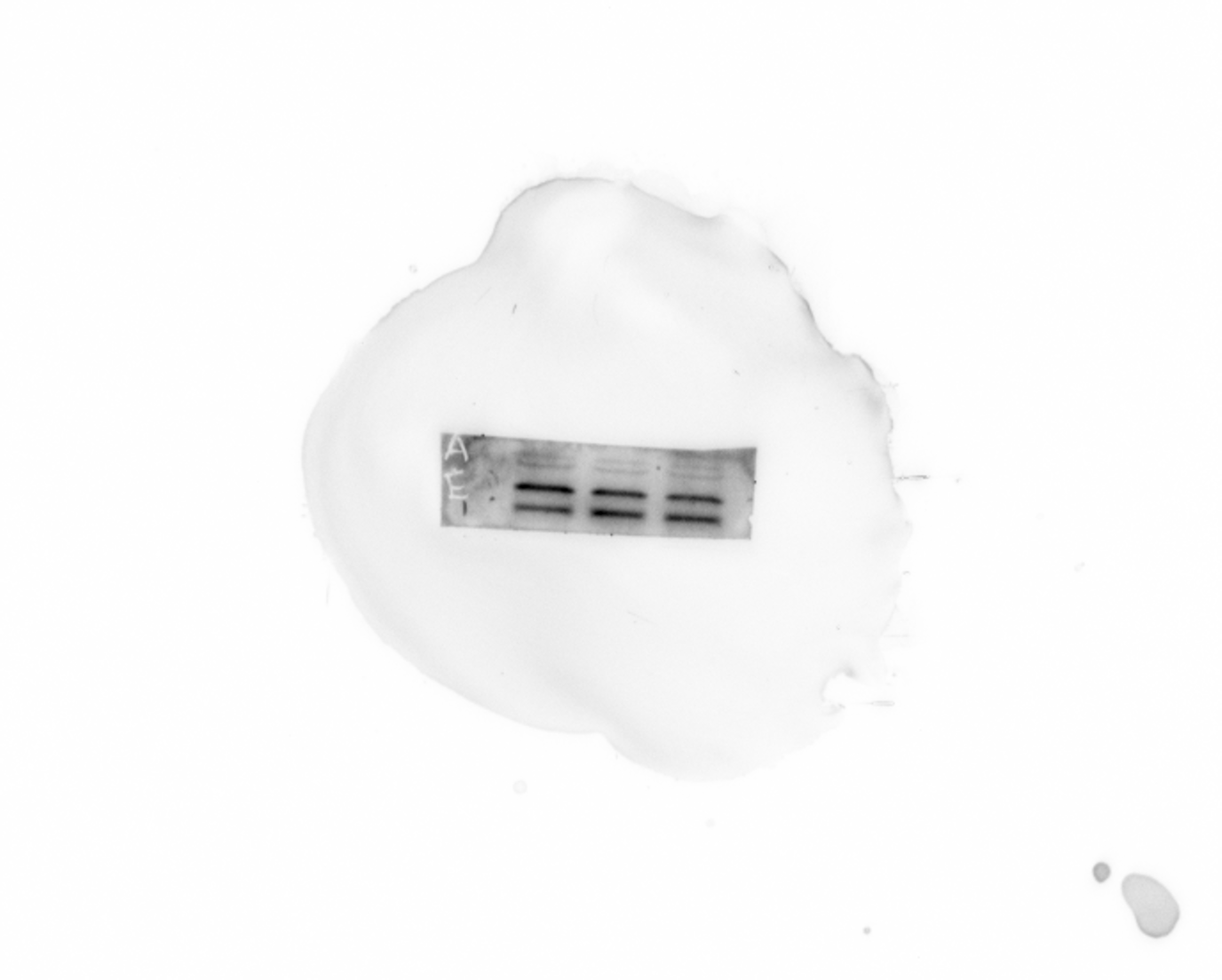

Supplement: Supplementary file 3 [file DataSheet1.ZIP › WB/AMO-1/10 p-erk12/10.7/AE.Tif]

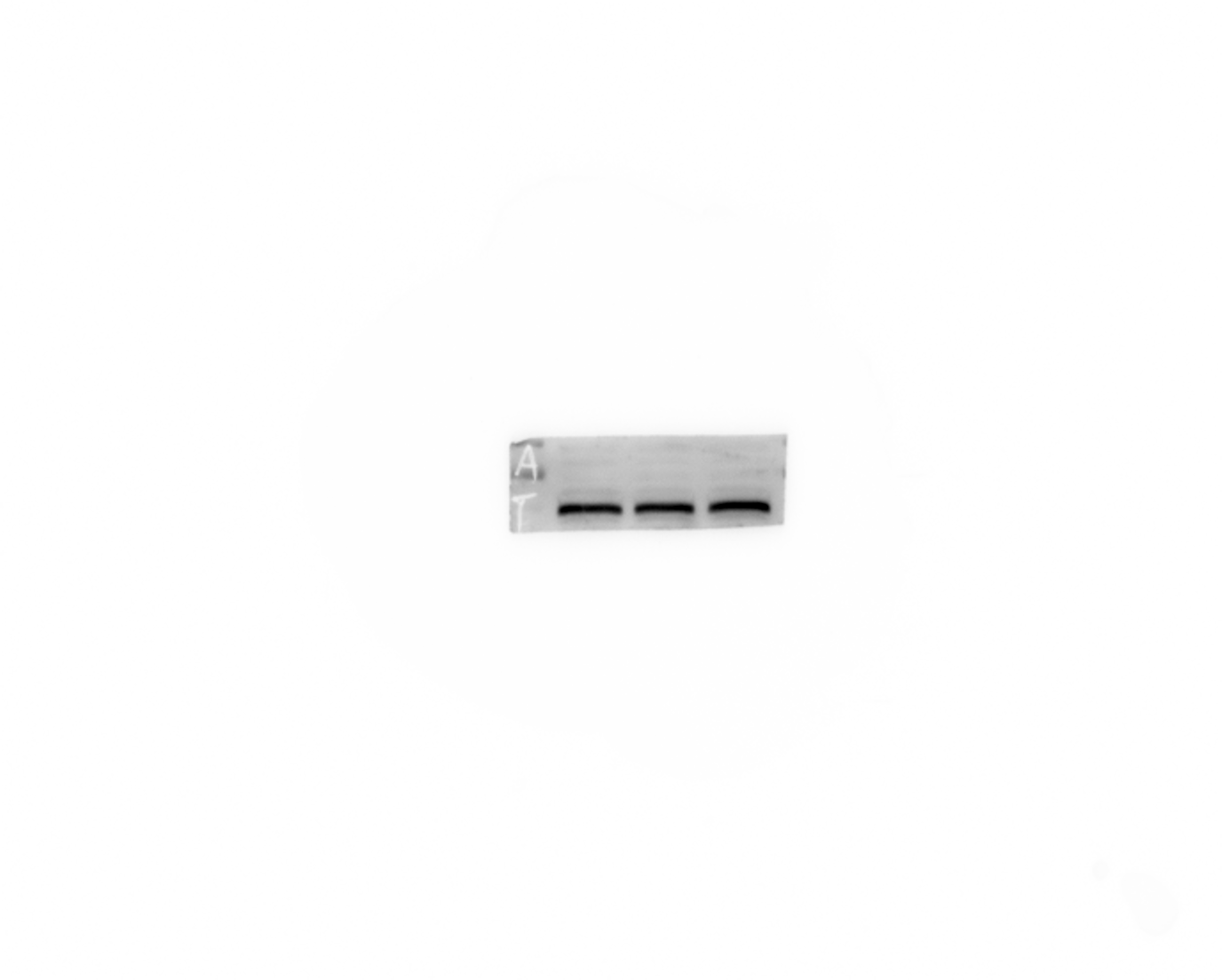

Supplement: Supplementary file 3 [file DataSheet1.ZIP › WB/AMO-1/10 p-erk12/10.7/a┬-tubulin.Tif]

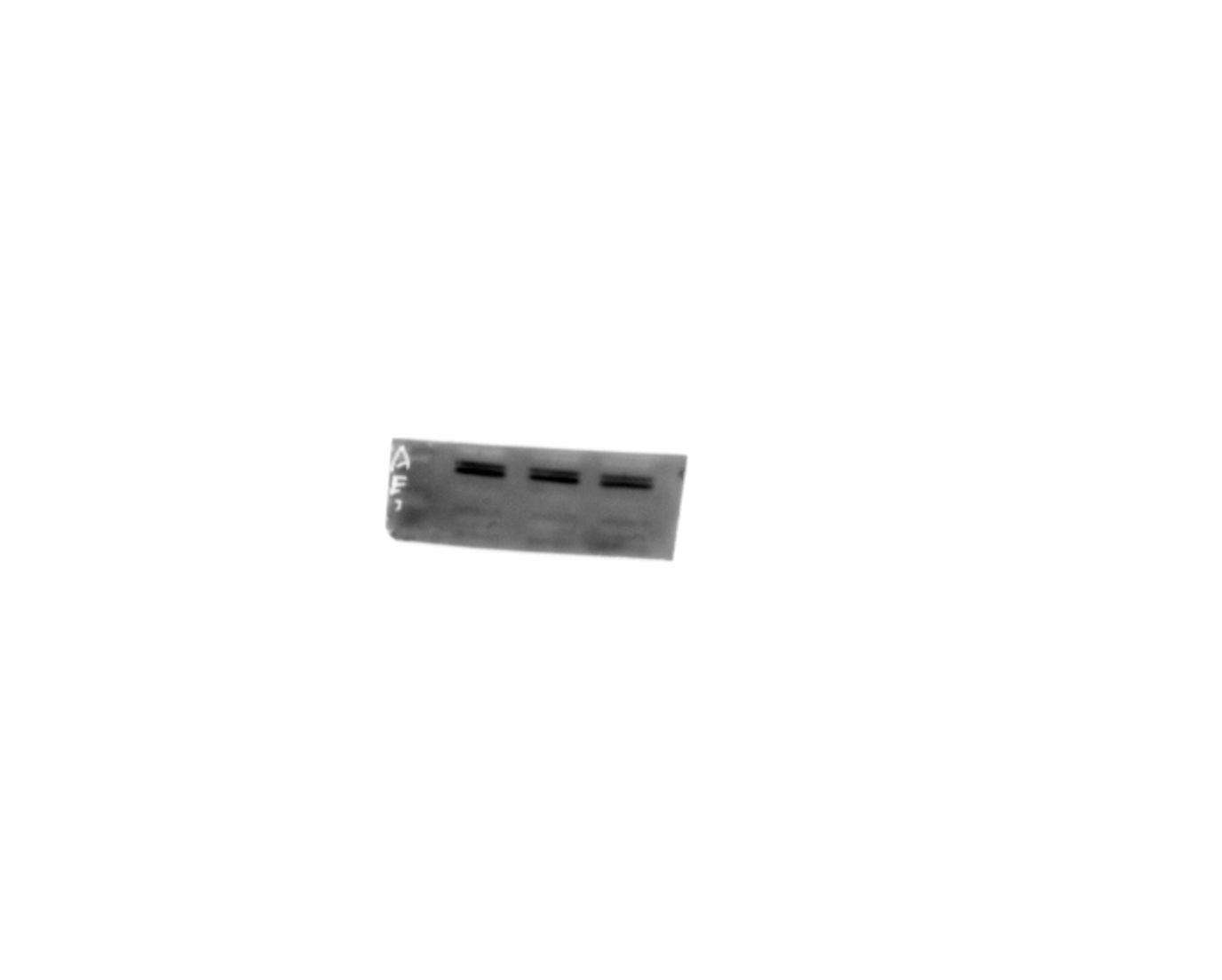

Supplement: Supplementary file 3 [file DataSheet1.ZIP › WB/AMO-1/10 p-erk12/10.9/AE2.Tif]

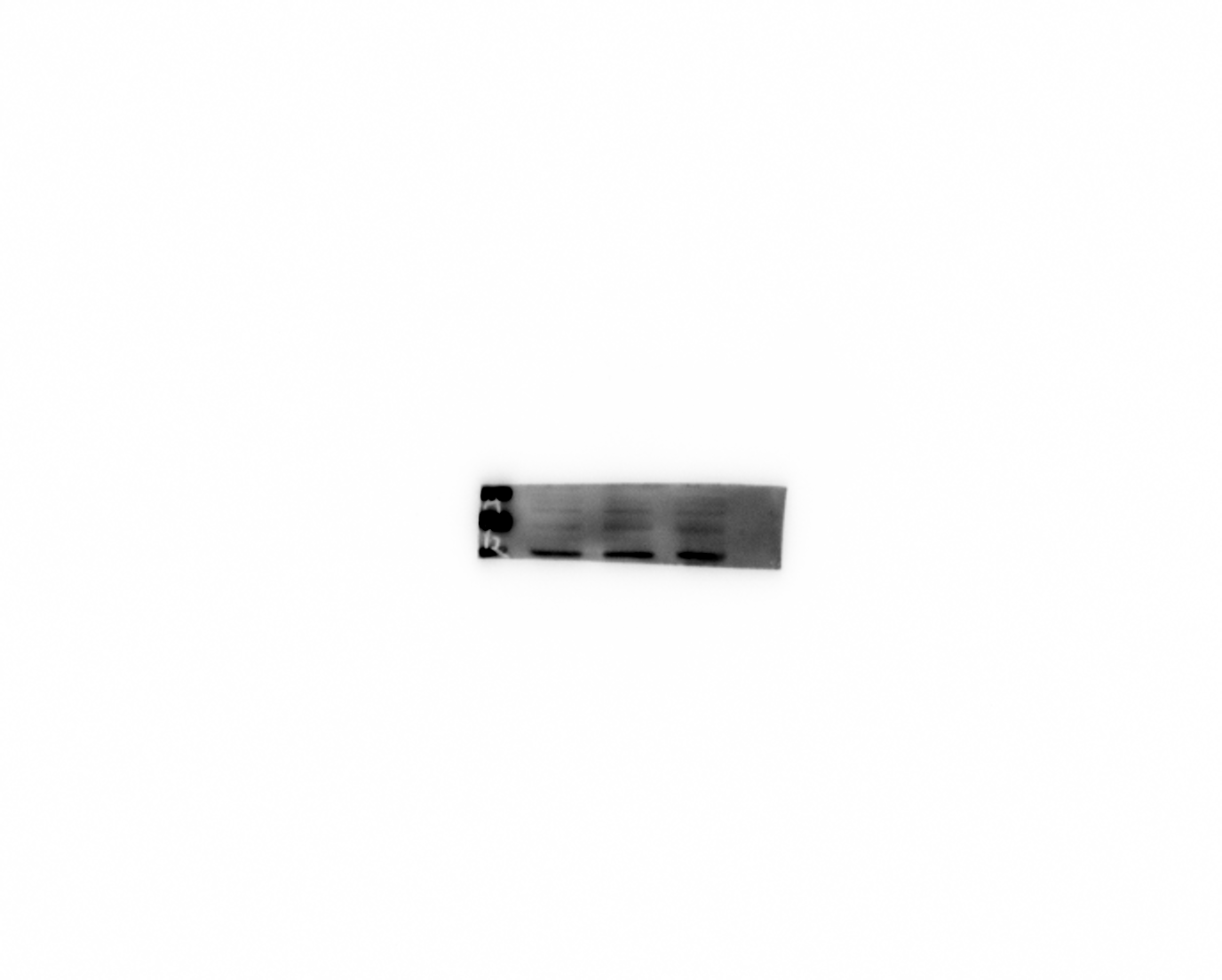

Supplement: Supplementary file 3 [file DataSheet1.ZIP › WB/AMO-1/10 p-erk12/10.9/Aa┬-tubulin.Tif]

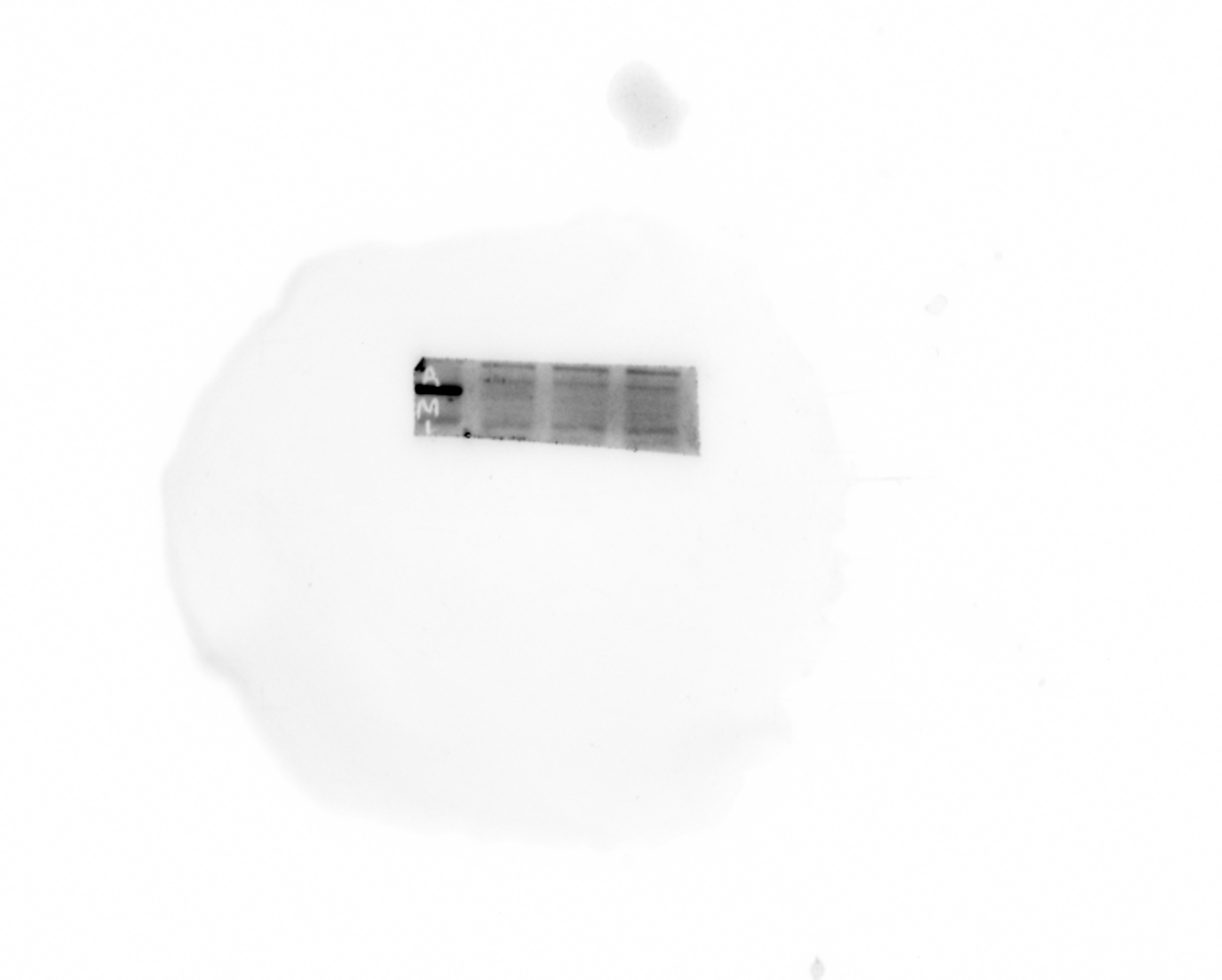

Supplement: Supplementary file 3 [file DataSheet1.ZIP › WB/AMO-1/11p-msk1/10.4/A PMSF1.Tif]

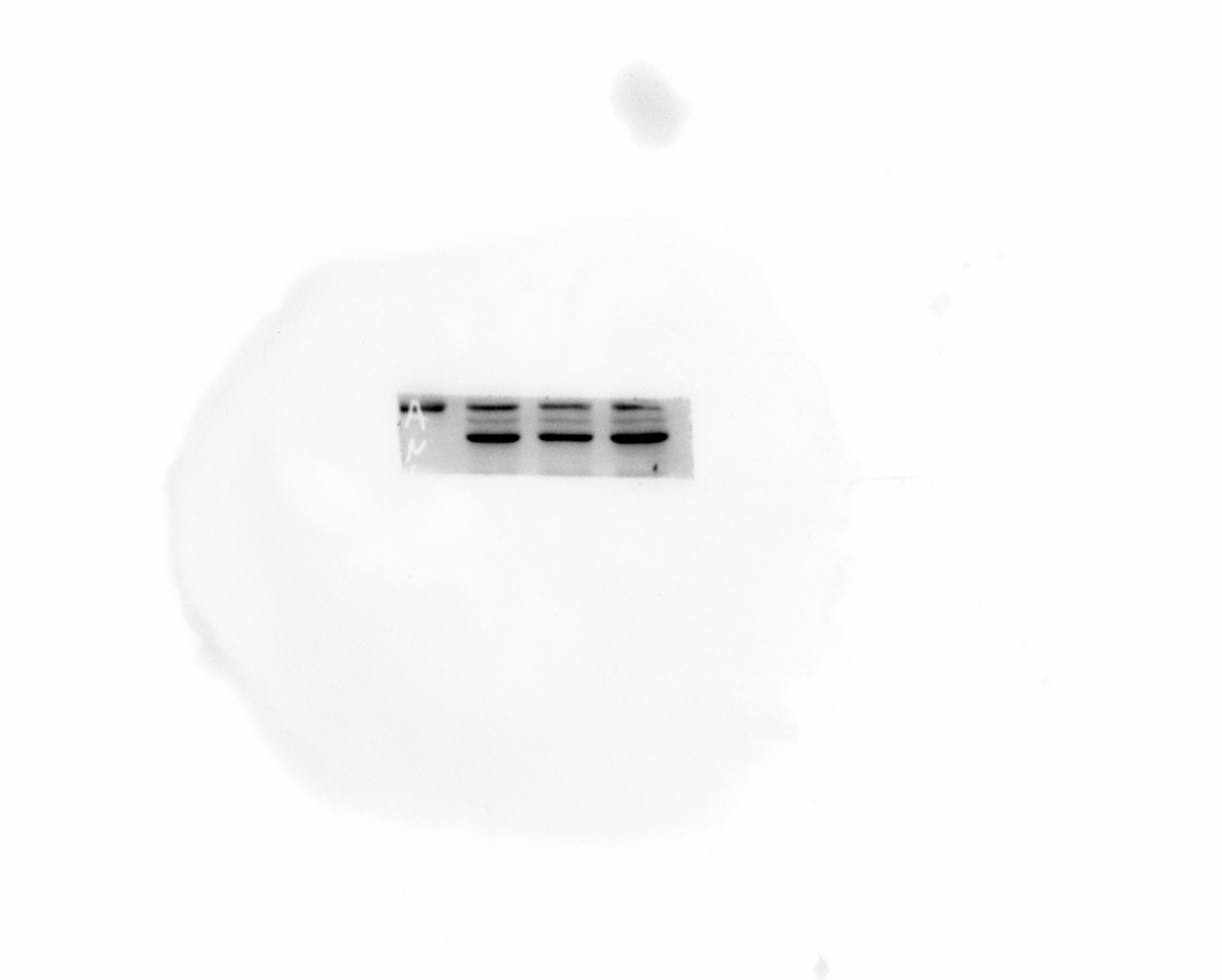

Supplement: Supplementary file 3 [file DataSheet1.ZIP › WB/AMO-1/11p-msk1/10.4/Aa┬-actin.Tif]

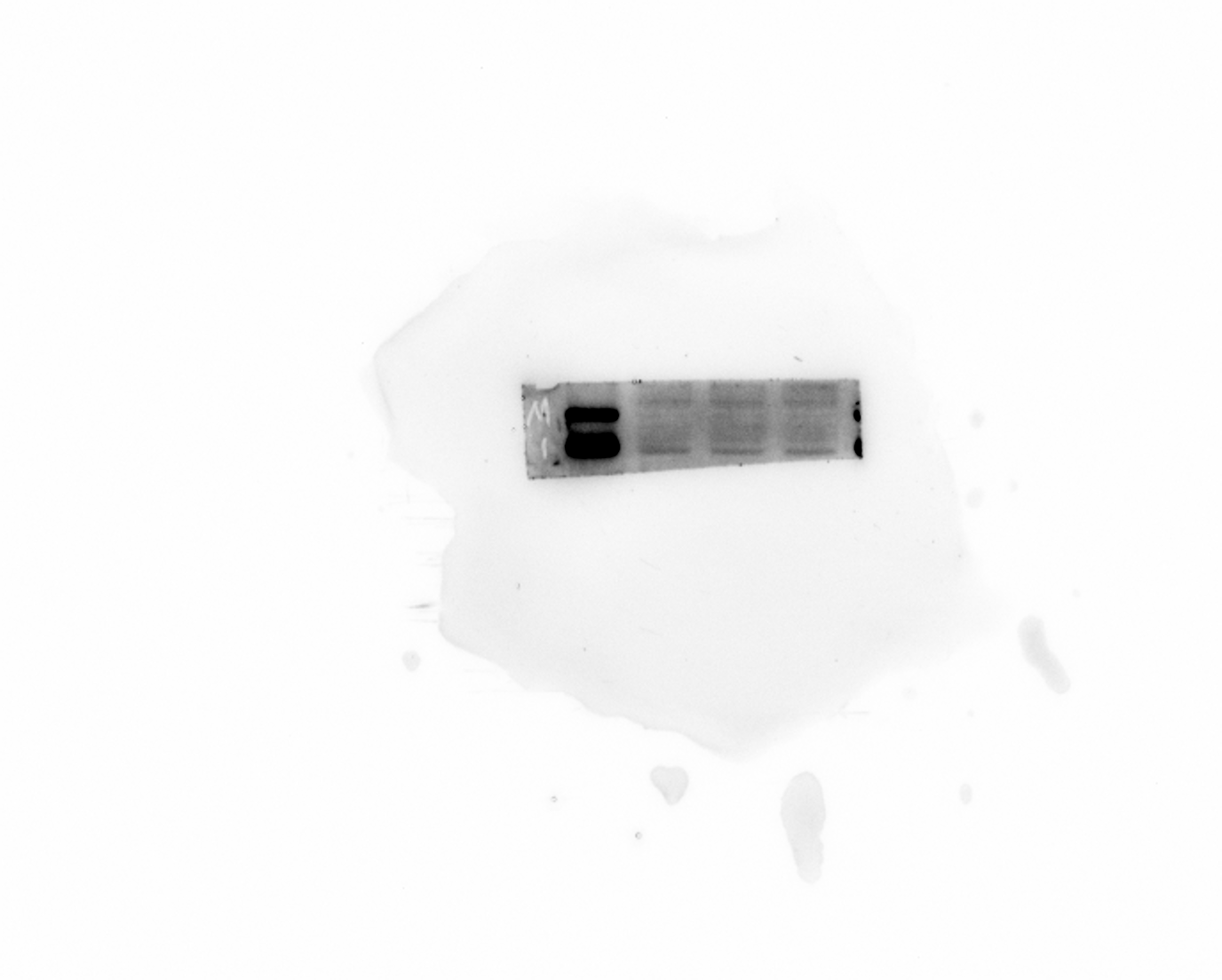

Supplement: Supplementary file 3 [file DataSheet1.ZIP › WB/AMO-1/11p-msk1/10.7/M1.Tif]

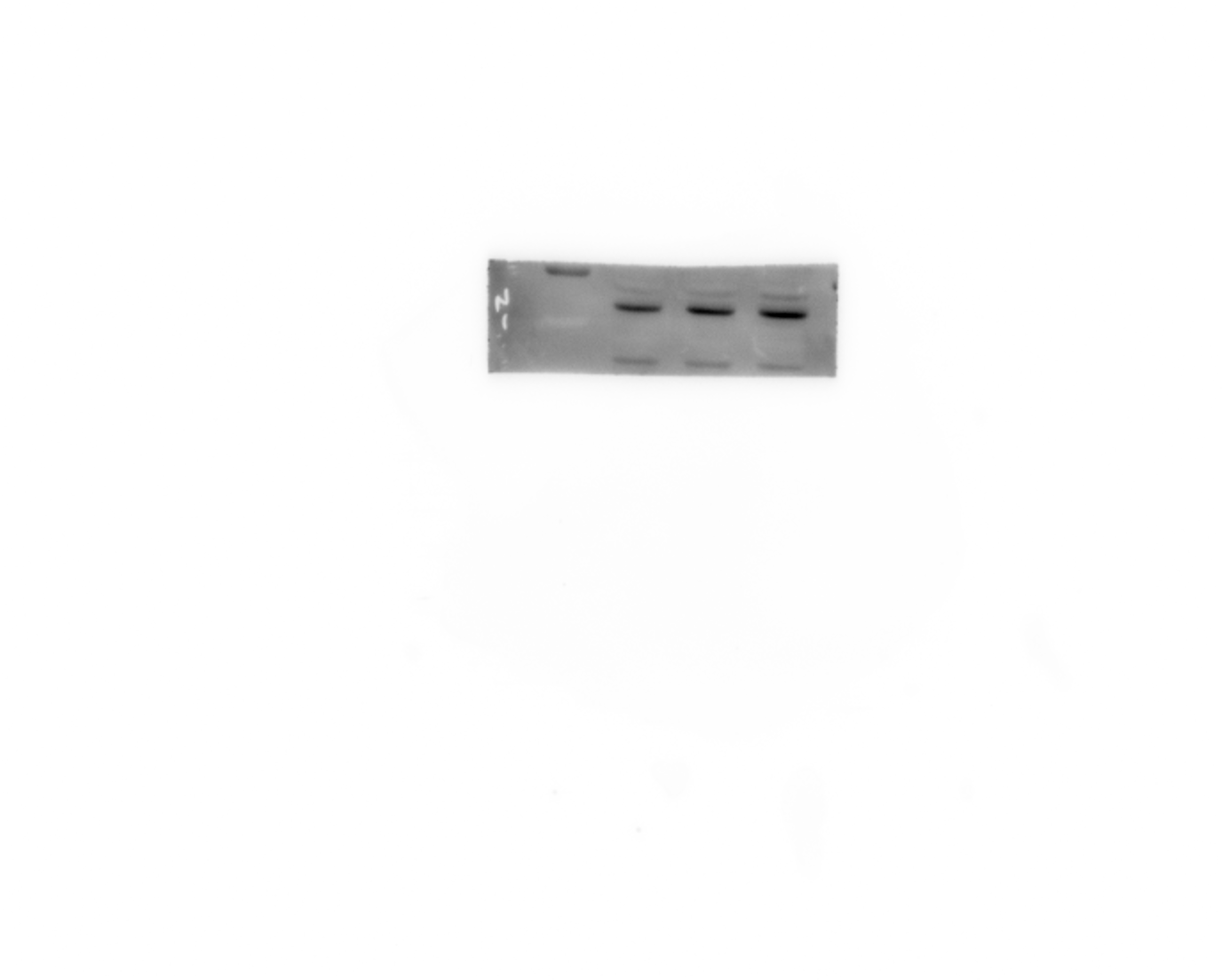

Supplement: Supplementary file 3 [file DataSheet1.ZIP › WB/AMO-1/11p-msk1/10.7/a┬-actin.Tif]

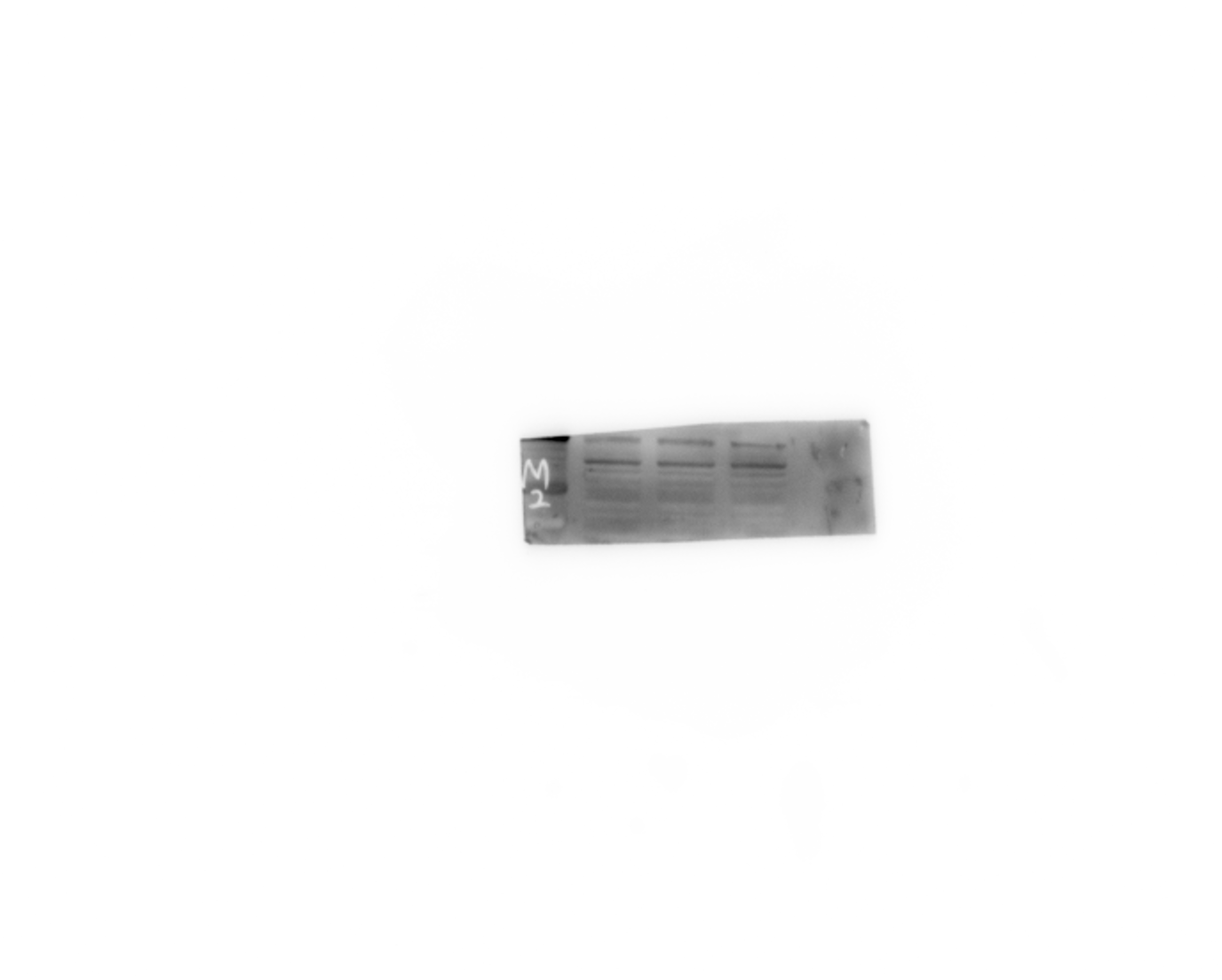

Supplement: Supplementary file 3 [file DataSheet1.ZIP › WB/AMO-1/11p-msk1/10.8/A M2.Tif]
