# Supplementary material for: LAMP5 may promote MM progression by activating p38
Source: Pathol Oncol Res. 2023 Mar 22;29:1611083. doi: 10.3389/pore.2023.1611083 (PMC10073510; doi:10.3389/pore.2023.1611083)

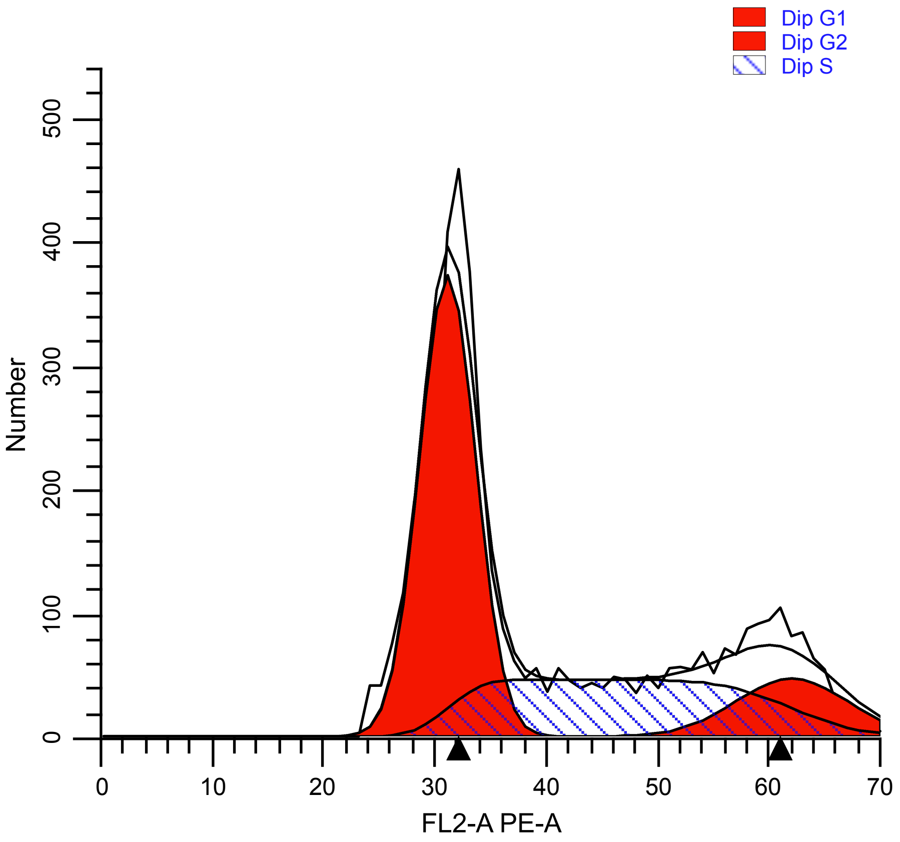


File analyzed: 8226 NC   1.fcs
Date analyzed: 10-Oct-2022
Model: 1nn0n_DSD
Analysis type: Manual analysis
Auto Linearity: No

Ploidy Mode: First cycle is diploid

Diploid: 100.00 %
  Dip G1: 54.27 % at 31.00
  Dip G2: 12.99 % at 61.99
  Dip S: 32.73 %   G2/G1: 2.00
  %CV: 8.11

Total S-Phase: 32.73 %
Total B.A.D.: 0.00 %   no debris no aggs

Debris:  %
Aggregates:  %
Modeled events: 4354
All cycle events: 4354
Cycle events per channel: 136
RCS: 3.321

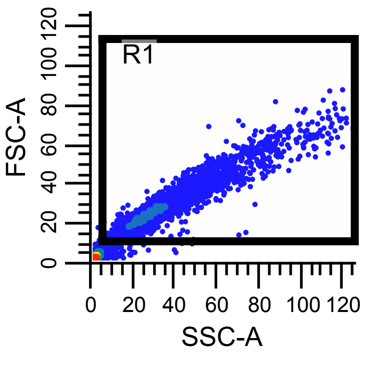


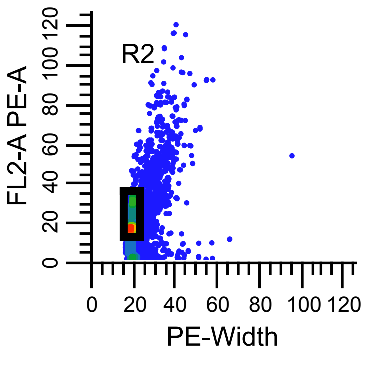

Supplement: Supplementary file 2 [file DataSheet4.ZIP › 8226 cell cycle/1/8226 nc 1 ╖╓╬÷.pdf]
