# Supplementary material for: LAMP5 may promote MM progression by activating p38
Source: Pathol Oncol Res. 2023 Mar 22;29:1611083. doi: 10.3389/pore.2023.1611083 (PMC10073510; doi:10.3389/pore.2023.1611083)

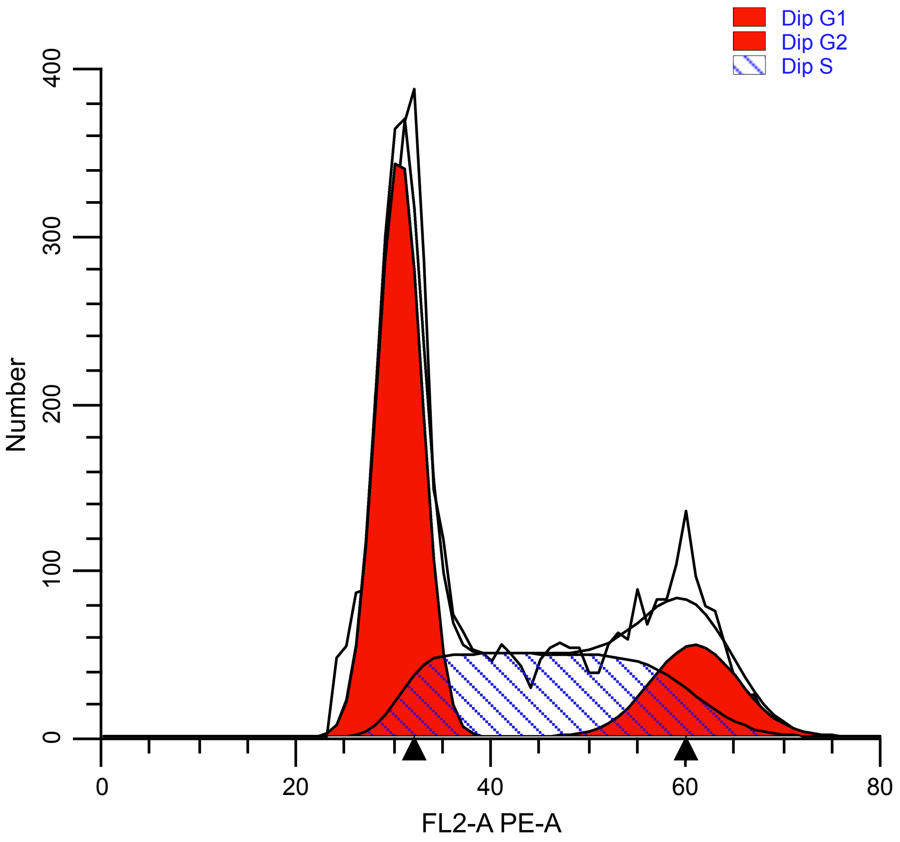

File analyzed: 8226 SI3 1.fcs
Date analyzed: 10-Oct-2022
Model: 1nn0n_DSD
Analysis type: Manual analysis
Auto Linearity: No

Ploidy Mode: First cycle is diploid

Diploid: 100.00 %
Dip G1: 48.60 % at 30.45
Dip G2: 15.09 % at 60.90
Dip S: 36.31 %   G2/G1: 2.00
%CV: 7.49

Total S-Phase: 36.31 %
Total B.A.D.: 0.00 %   no debris no aggs

Debris: %
Aggregates: %
Modeled events: 4153
All cycle events: 4153
Cycle events per channel: 132
RCS: 3.306

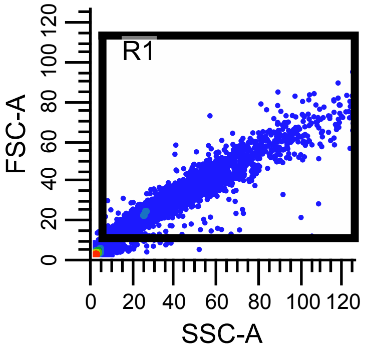

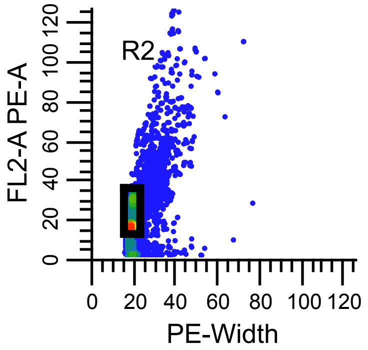

Supplement: Supplementary file 2 [file DataSheet4.ZIP › 8226 cell cycle/1/8226 si3 1 ╖╓╬÷.pdf]
