# Supplementary material for: LAMP5 may promote MM progression by activating p38
Source: Pathol Oncol Res. 2023 Mar 22;29:1611083. doi: 10.3389/pore.2023.1611083 (PMC10073510; doi:10.3389/pore.2023.1611083)
Supplement: Supplementary file 2 [file DataSheet4.ZIP › 8226 cell cycle/1/8226 si4 1 ╖╓╬÷.pdf]

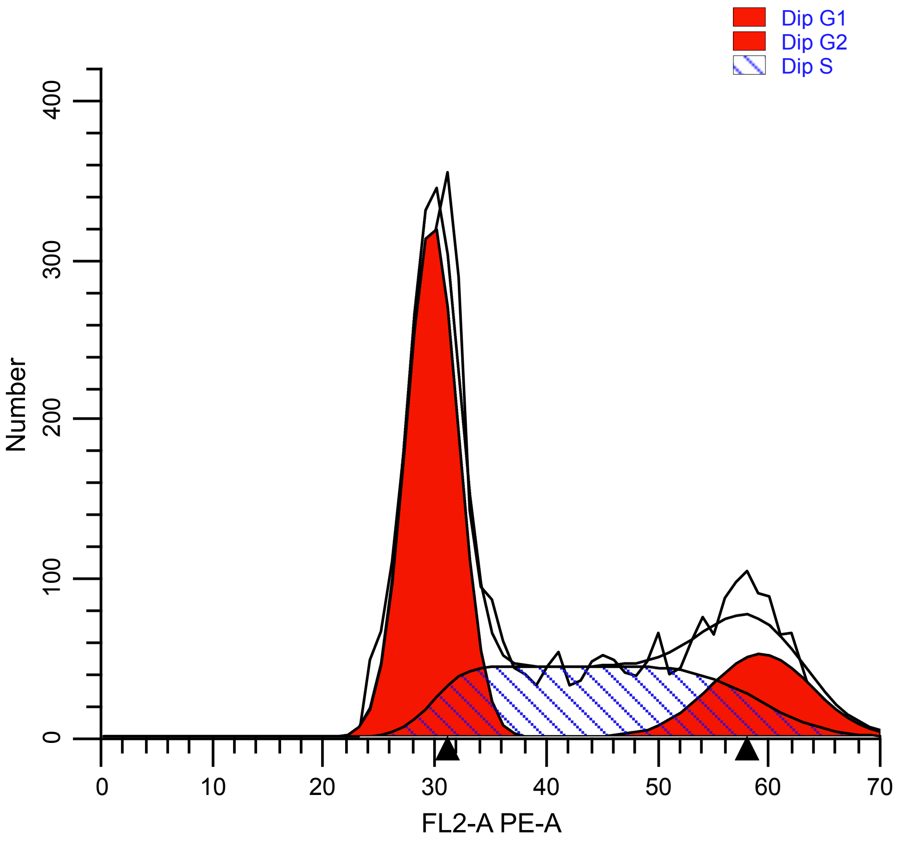

File analyzed: 8226 SI4 1.fcs
Date analyzed: 10-Oct-2022
Model: 1nn0n_DSD
Analysis type: Manual analysis
Auto Linearity: No

Ploidy Mode: First cycle is diploid

Diploid: 100.00 %
Dip G1: 49.77 % at 29.61
Dip G2: 15.65 % at 59.21
Dip S: 34.57 %   G2/G1: 2.00
%CV: 7.76

Total S-Phase: 34.57 %
Total B.A.D.: 0.00 %   no debris no aggs

Debris: %
Aggregates: %
Modeled events: 3780
All cycle events: 3780
Cycle events per channel: 124
RCS: 3.357

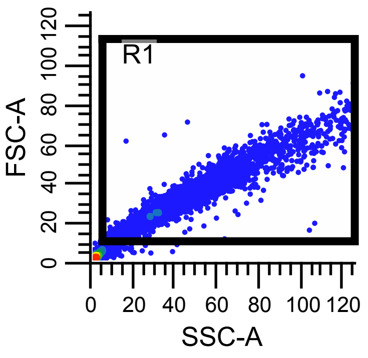

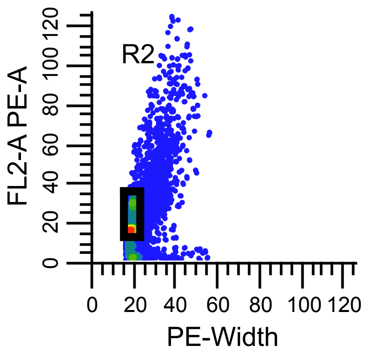

ModFit LT V4.1.7(Mac)
