# Supplementary material for: LAMP5 may promote MM progression by activating p38
Source: Pathol Oncol Res. 2023 Mar 22;29:1611083. doi: 10.3389/pore.2023.1611083 (PMC10073510; doi:10.3389/pore.2023.1611083)

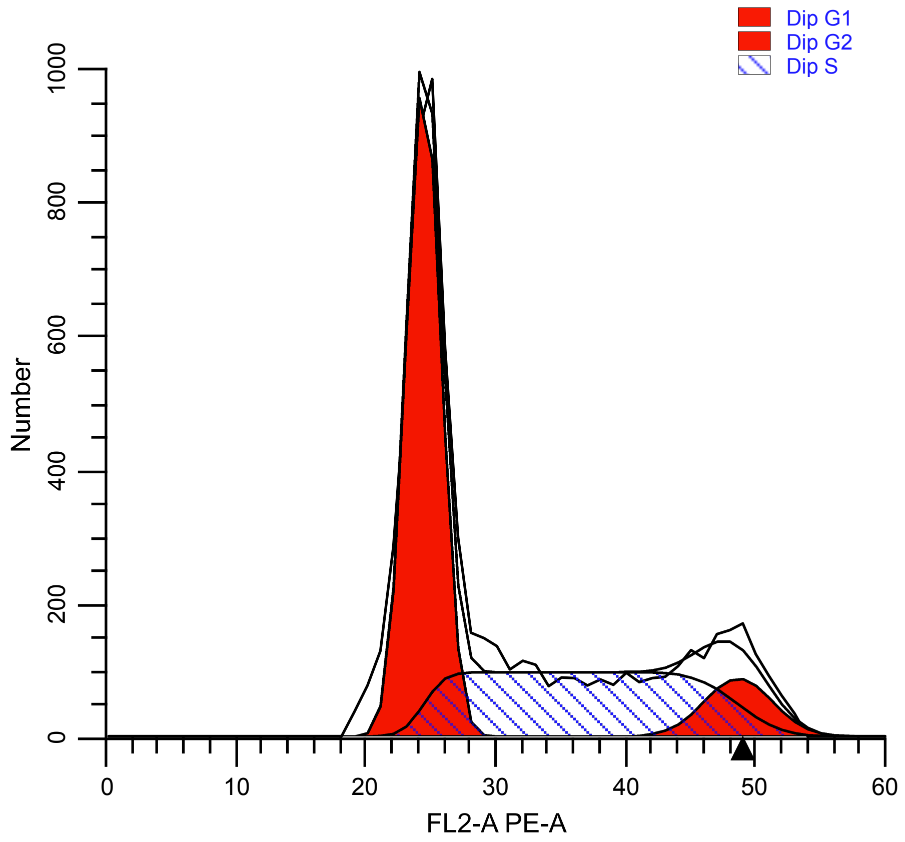

File analyzed: 8226 SI4 4.fcs
Date analyzed: 10-Oct-2022
Model: 1nn0n_DSD
Analysis type: Manual analysis
Auto Linearity: No

Ploidy Mode: First cycle is diploid

Diploid: 100.00 %
Dip G1: 53.11 % at 24.32
Dip G2: 9.22 % at 48.64
Dip S: 37.68 % G2/G1: 2.00
%CV: 5.37

Total S-Phase: 37.68 %
Total B.A.D.: 0.00 % no debris no aggs

Debris: %
Aggregates: %
Modeled events: 6213
All cycle events: 6213
Cycle events per channel: 245
RCS: 11.414

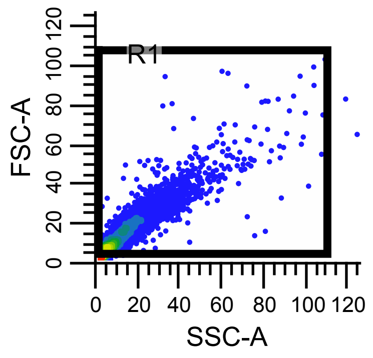

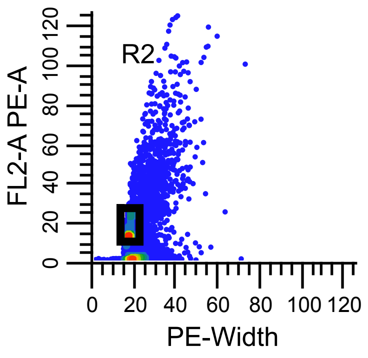

Supplement: Supplementary file 2 [file DataSheet4.ZIP › 8226 cell cycle/3/8226 si4 4 ╖╓╬÷.pdf]
