# Supplementary figures and images for: LAMP5 may promote MM progression by activating p38 (part 2 of 2)
Source: Pathol Oncol Res. 2023 Mar 22;29:1611083. doi: 10.3389/pore.2023.1611083 (PMC10073510; doi:10.3389/pore.2023.1611083)

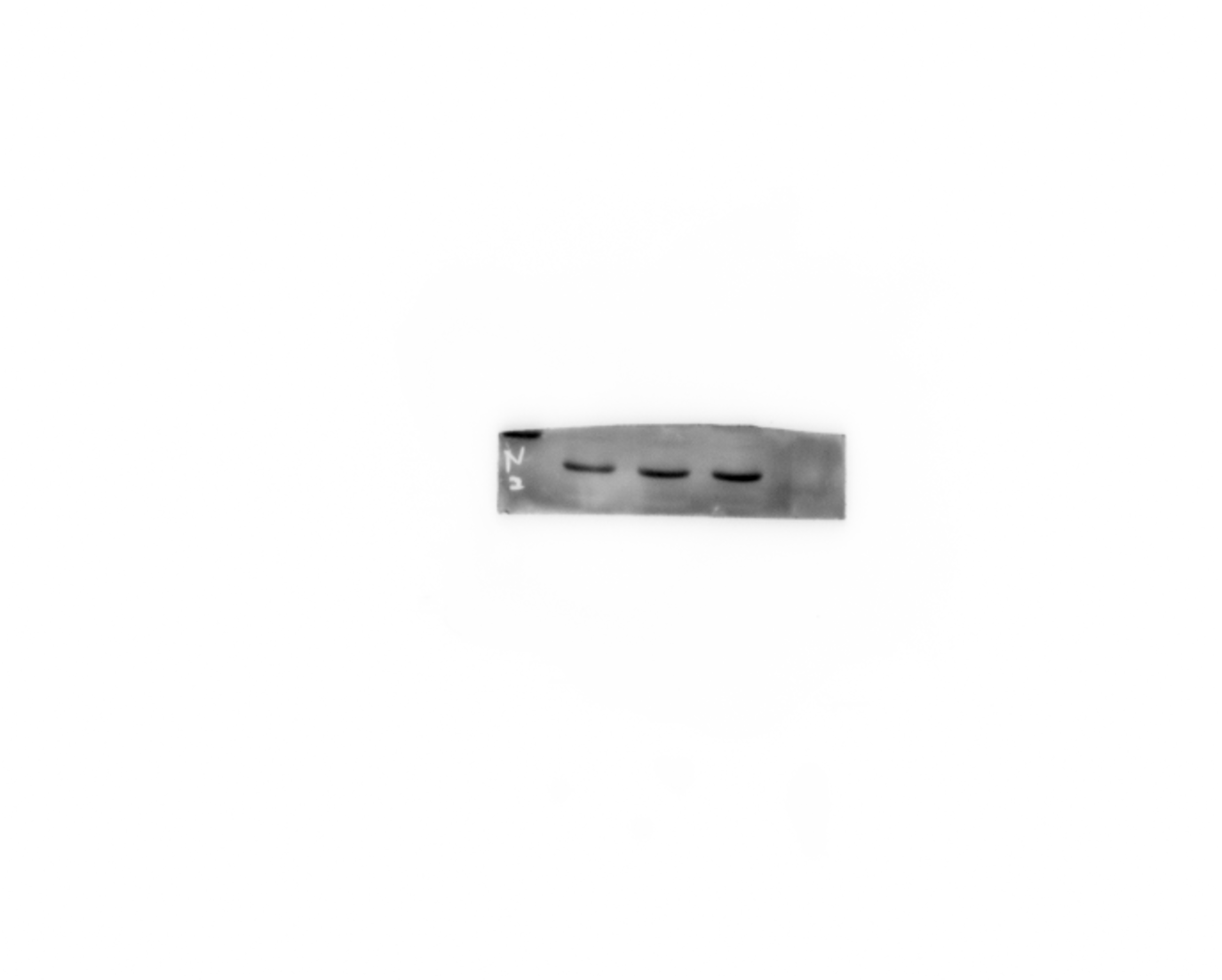

Supplement: Supplementary file 3 [file DataSheet1.ZIP › WB/AMO-1/11p-msk1/10.8/a┬-actin.Tif]

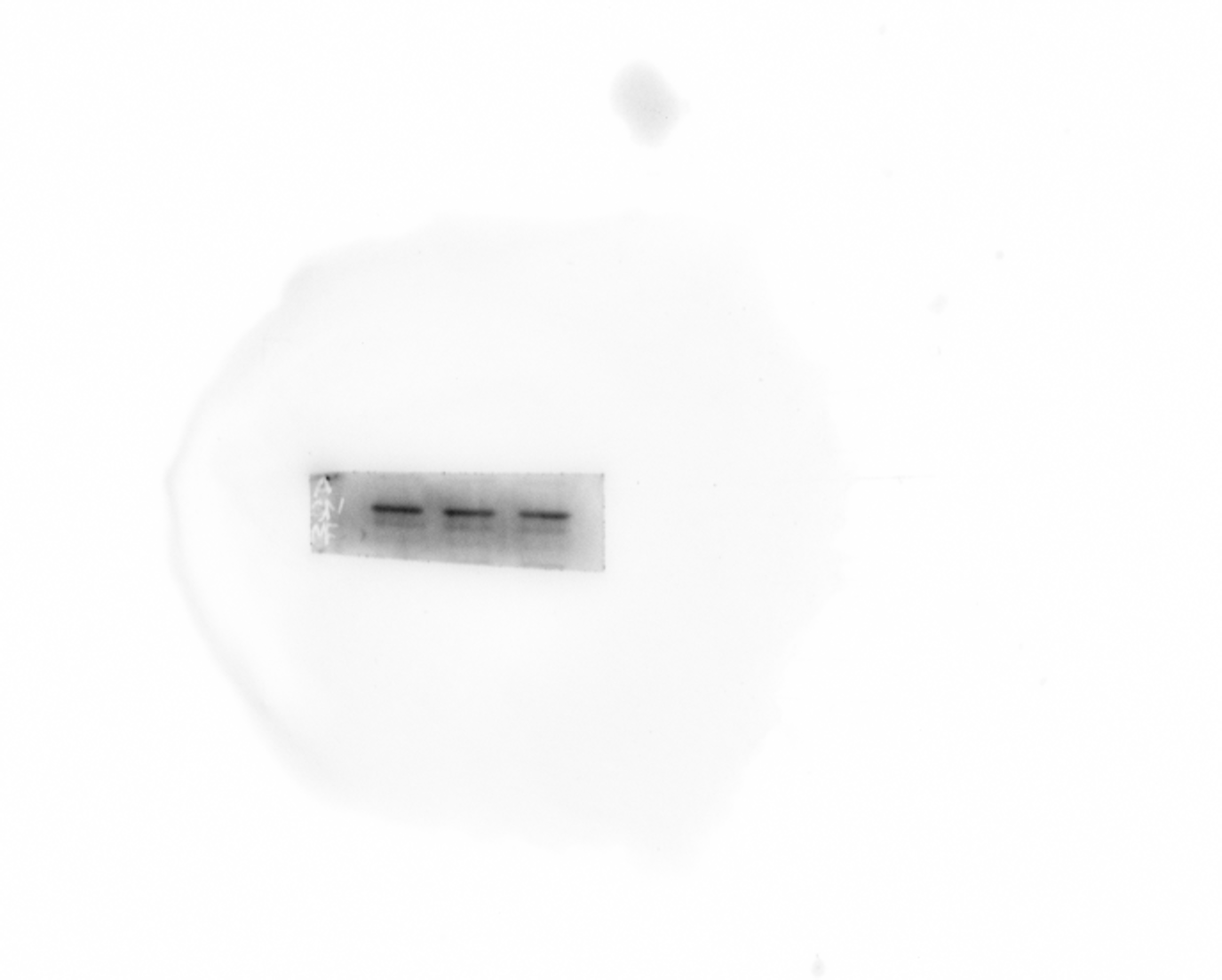

Supplement: Supplementary file 3 [file DataSheet1.ZIP › WB/AMO-1/12p-nf kb/10.4/A GAPDH.Tif]

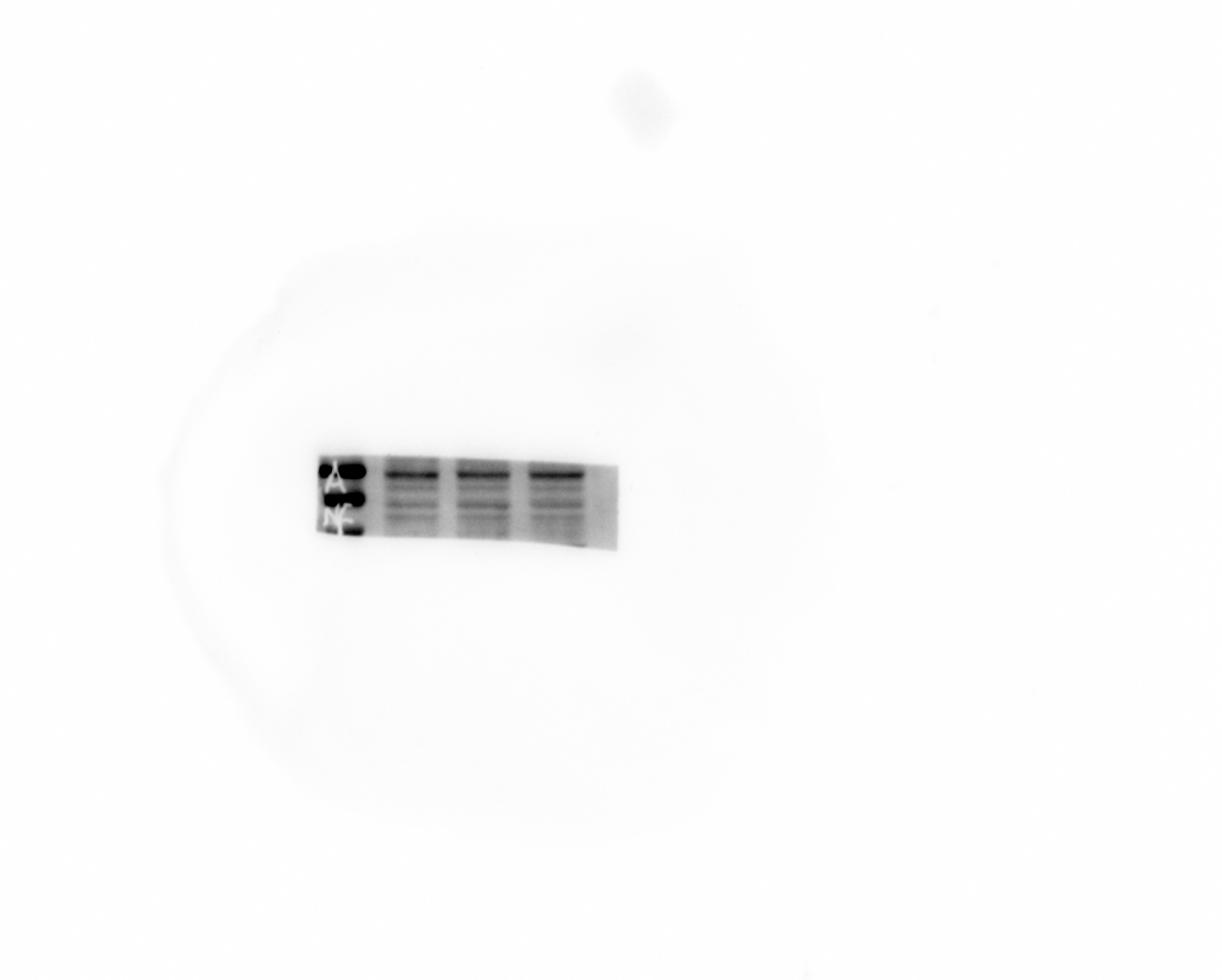

Supplement: Supplementary file 3 [file DataSheet1.ZIP › WB/AMO-1/12p-nf kb/10.4/A NF.Tif]

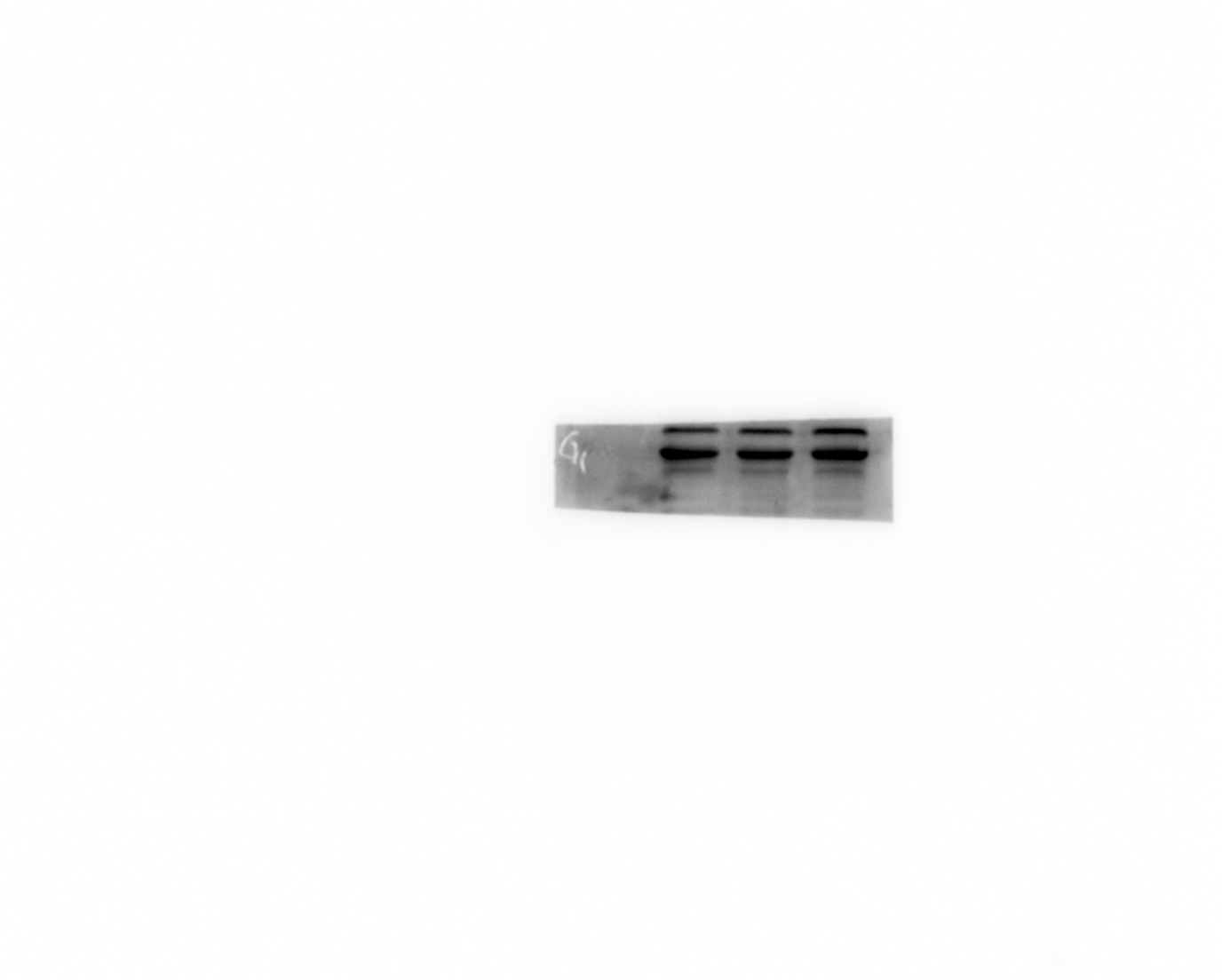

Supplement: Supplementary file 3 [file DataSheet1.ZIP › WB/AMO-1/12p-nf kb/10.6/A GAPDH.Tif]

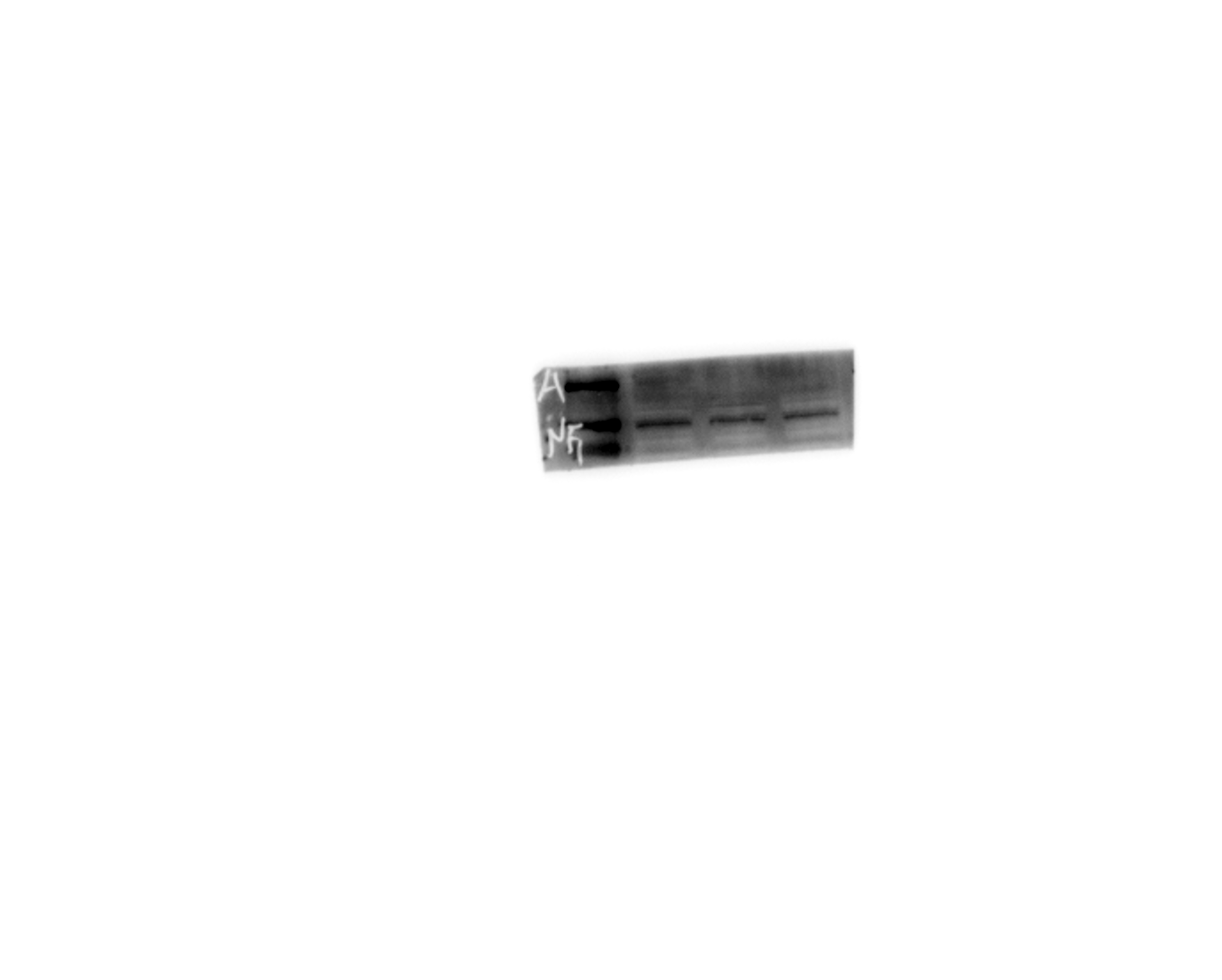

Supplement: Supplementary file 3 [file DataSheet1.ZIP › WB/AMO-1/12p-nf kb/10.6/A NFKB 1.Tif]

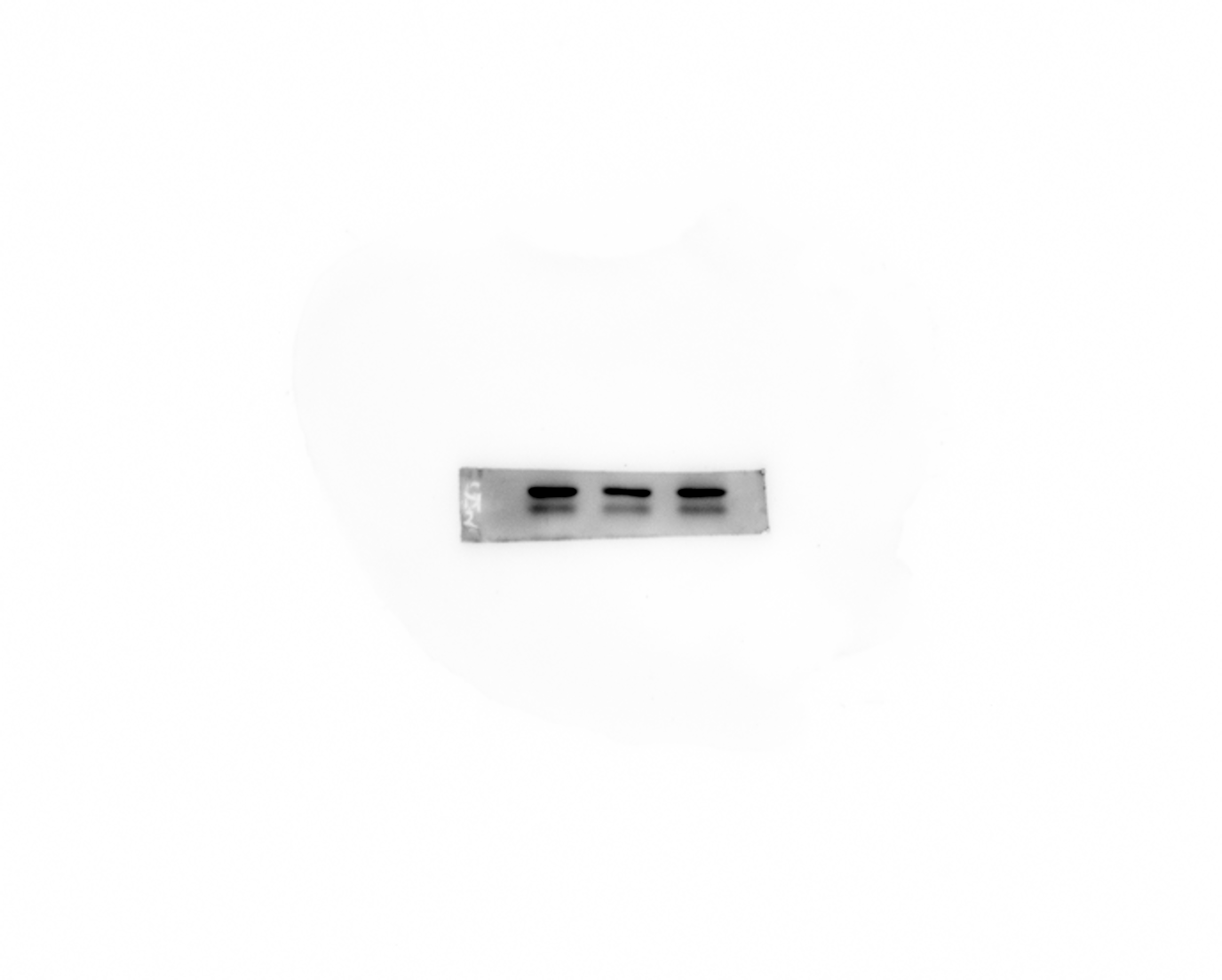

Supplement: Supplementary file 3 [file DataSheet1.ZIP › WB/AMO-1/12p-nf kb/10.9/GAPDH.Tif]

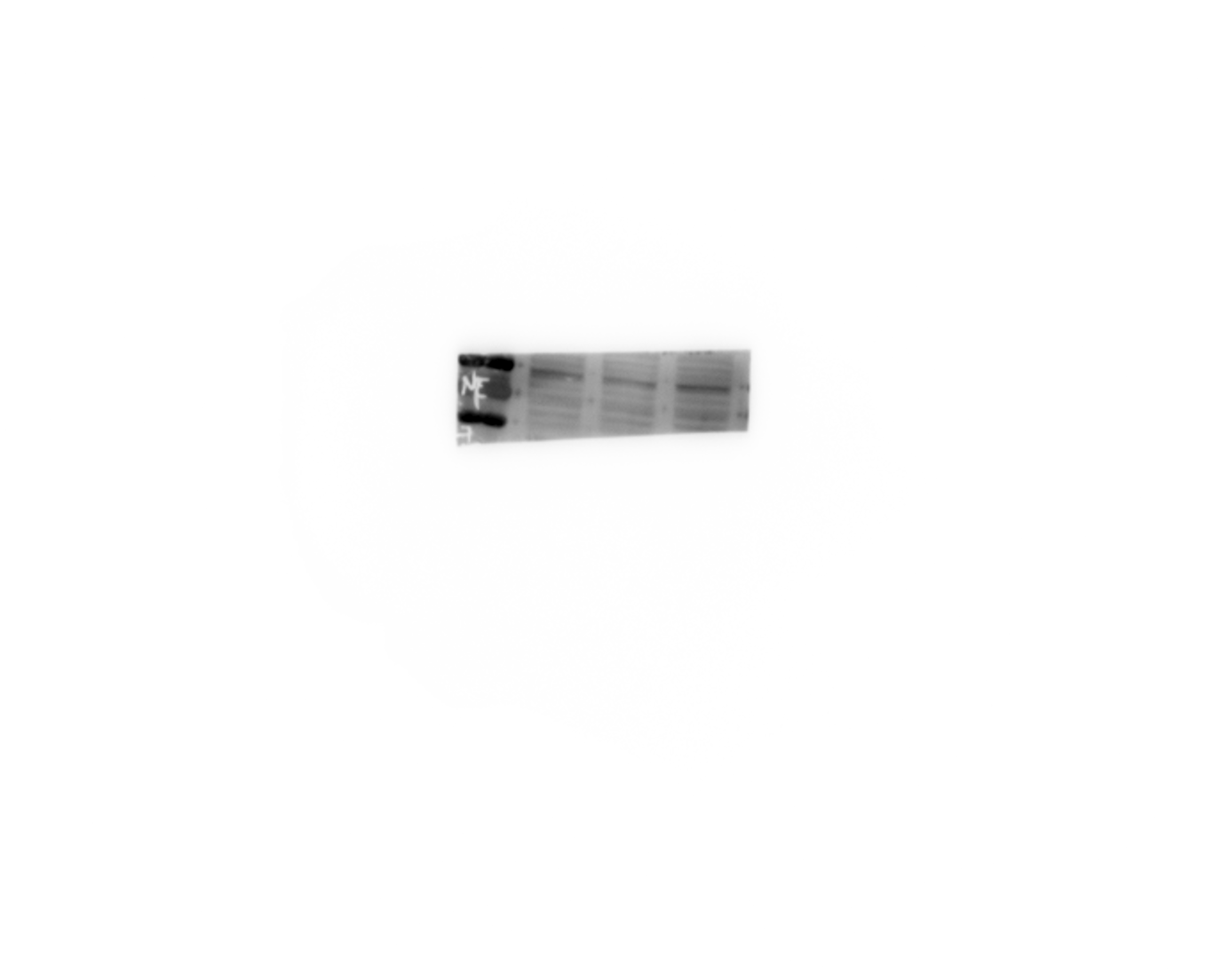

Supplement: Supplementary file 3 [file DataSheet1.ZIP › WB/AMO-1/12p-nf kb/10.9/NF2 2.Tif]

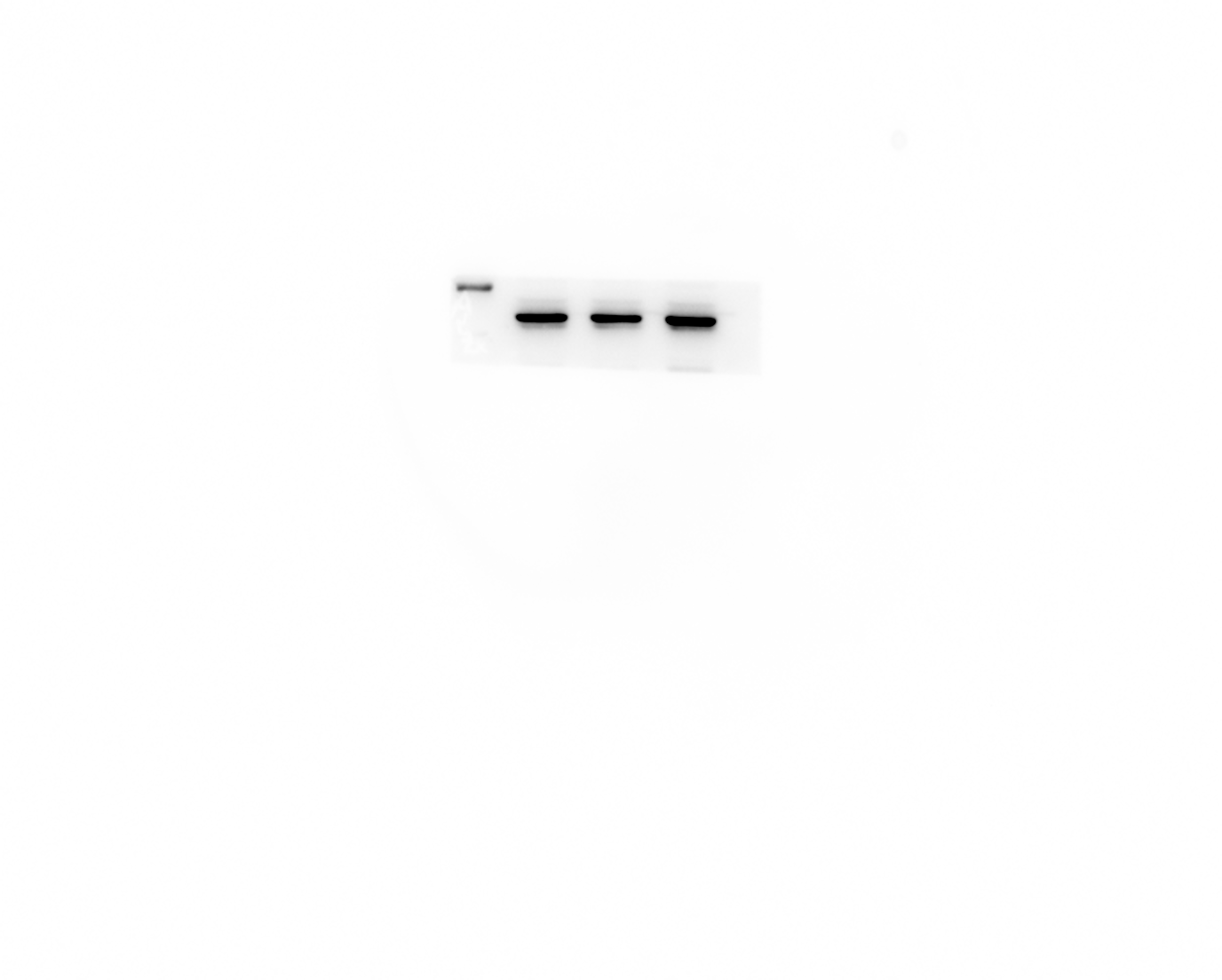

Supplement: Supplementary file 3 [file DataSheet1.ZIP › WB/AMO-1/2bcl2/9.26/Aa┬-actin.Tif]

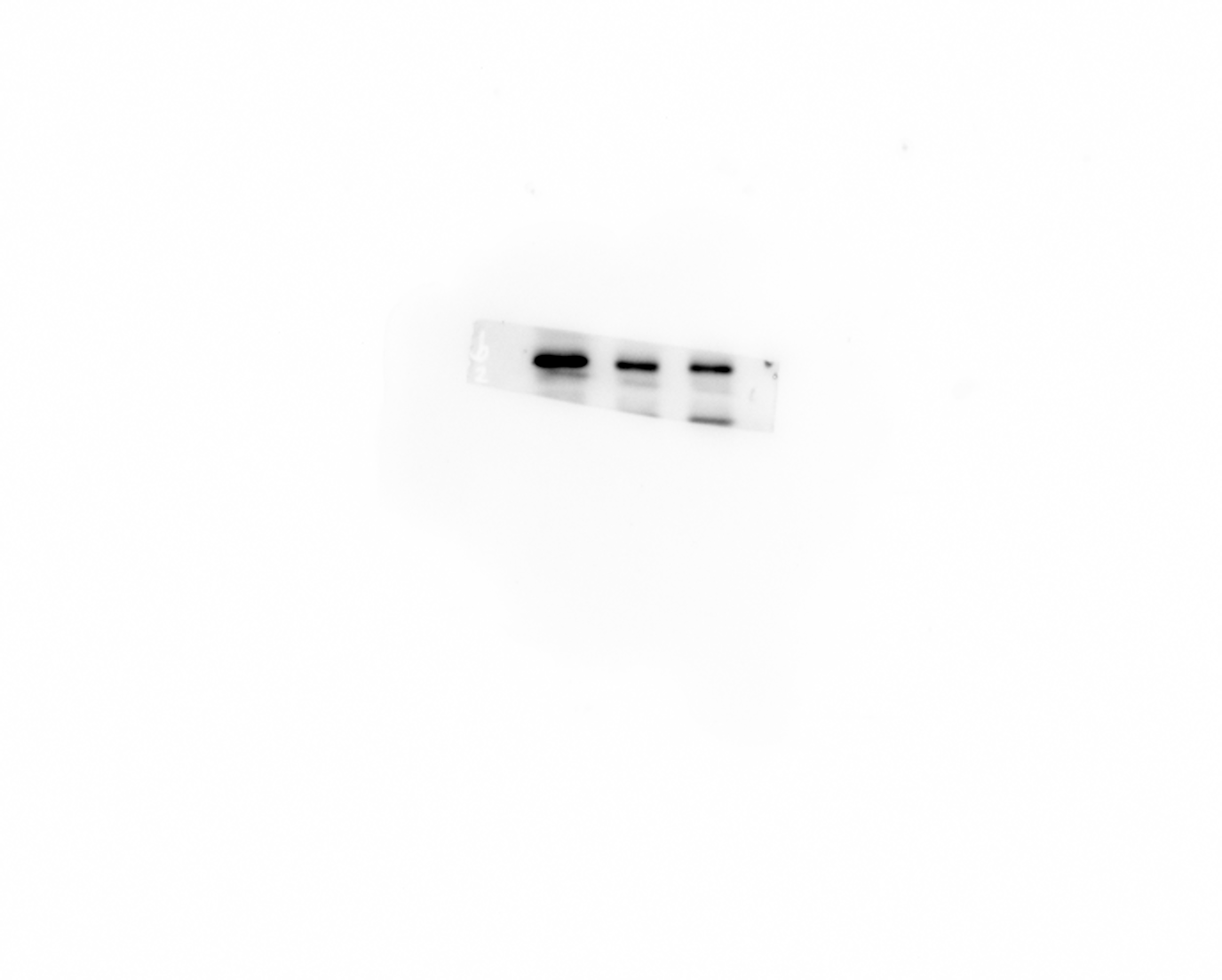

Supplement: Supplementary file 3 [file DataSheet1.ZIP › WB/AMO-1/2bcl2/9.26/BCL2.Tif]

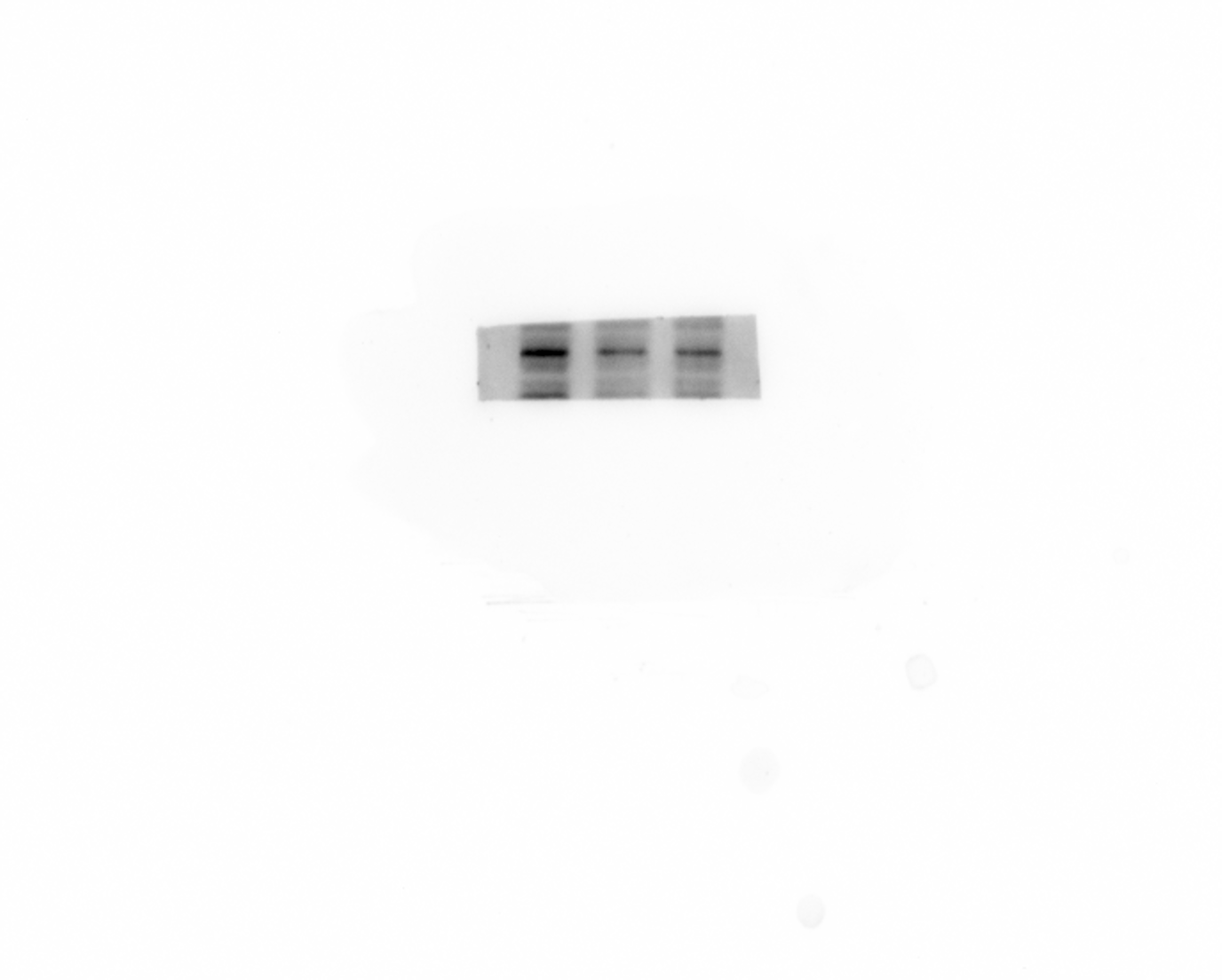

Supplement: Supplementary file 3 [file DataSheet1.ZIP › WB/AMO-1/2bcl2/9.27/A B2 1.Tif]

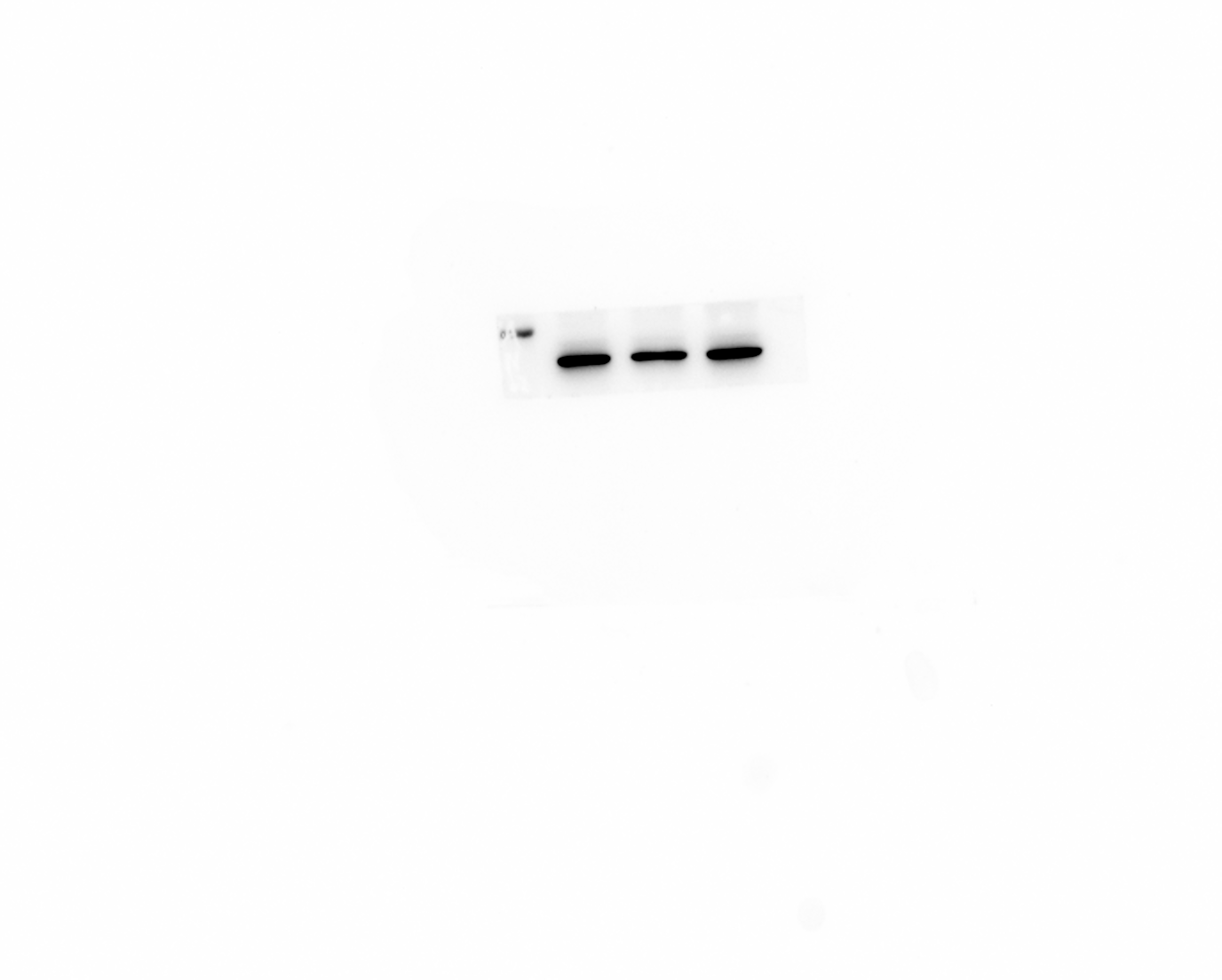

Supplement: Supplementary file 3 [file DataSheet1.ZIP › WB/AMO-1/2bcl2/9.27/Aa┬-actin.Tif]

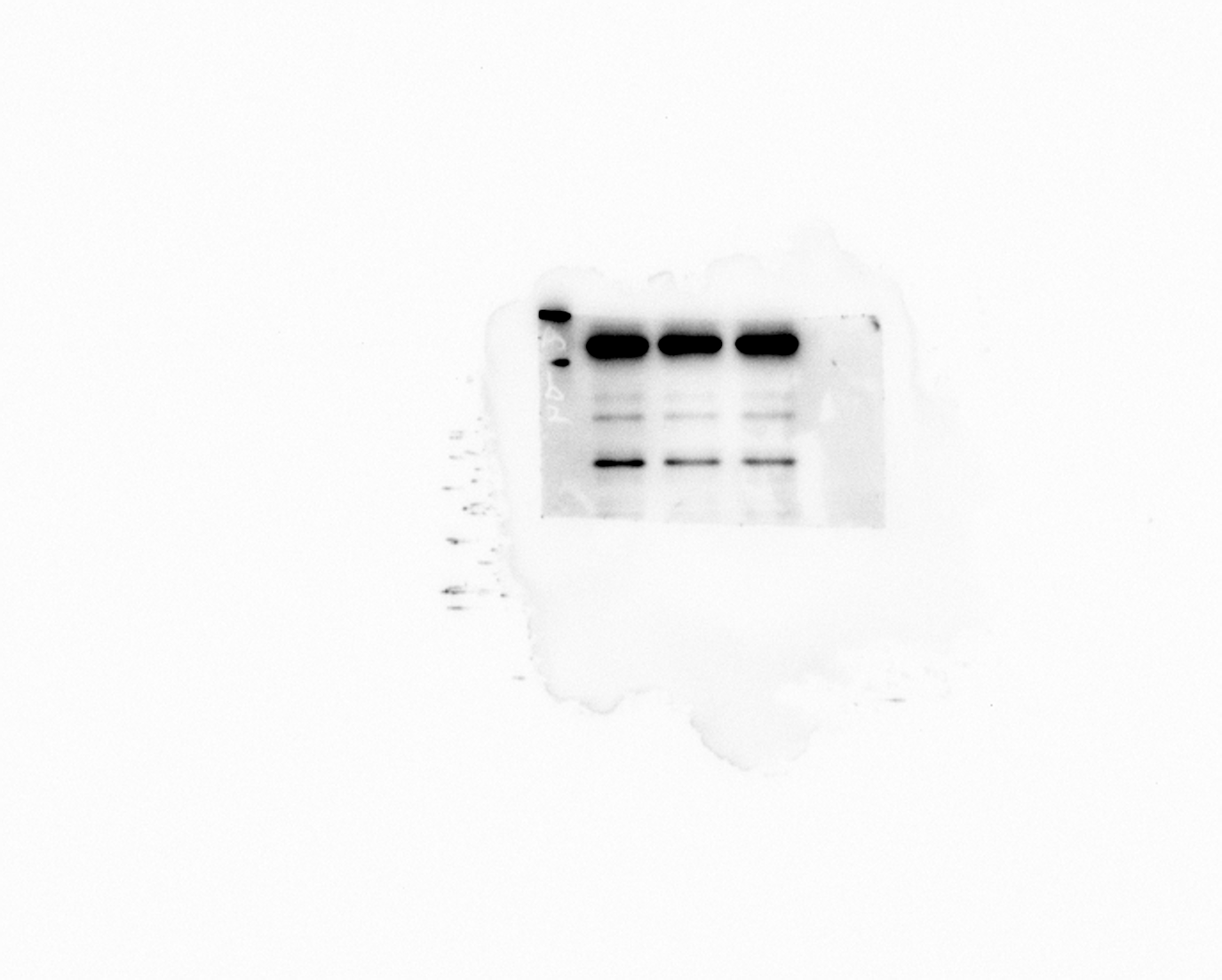

Supplement: Supplementary file 3 [file DataSheet1.ZIP › WB/AMO-1/2bcl2/9.28/AMO BCL2.Tif]

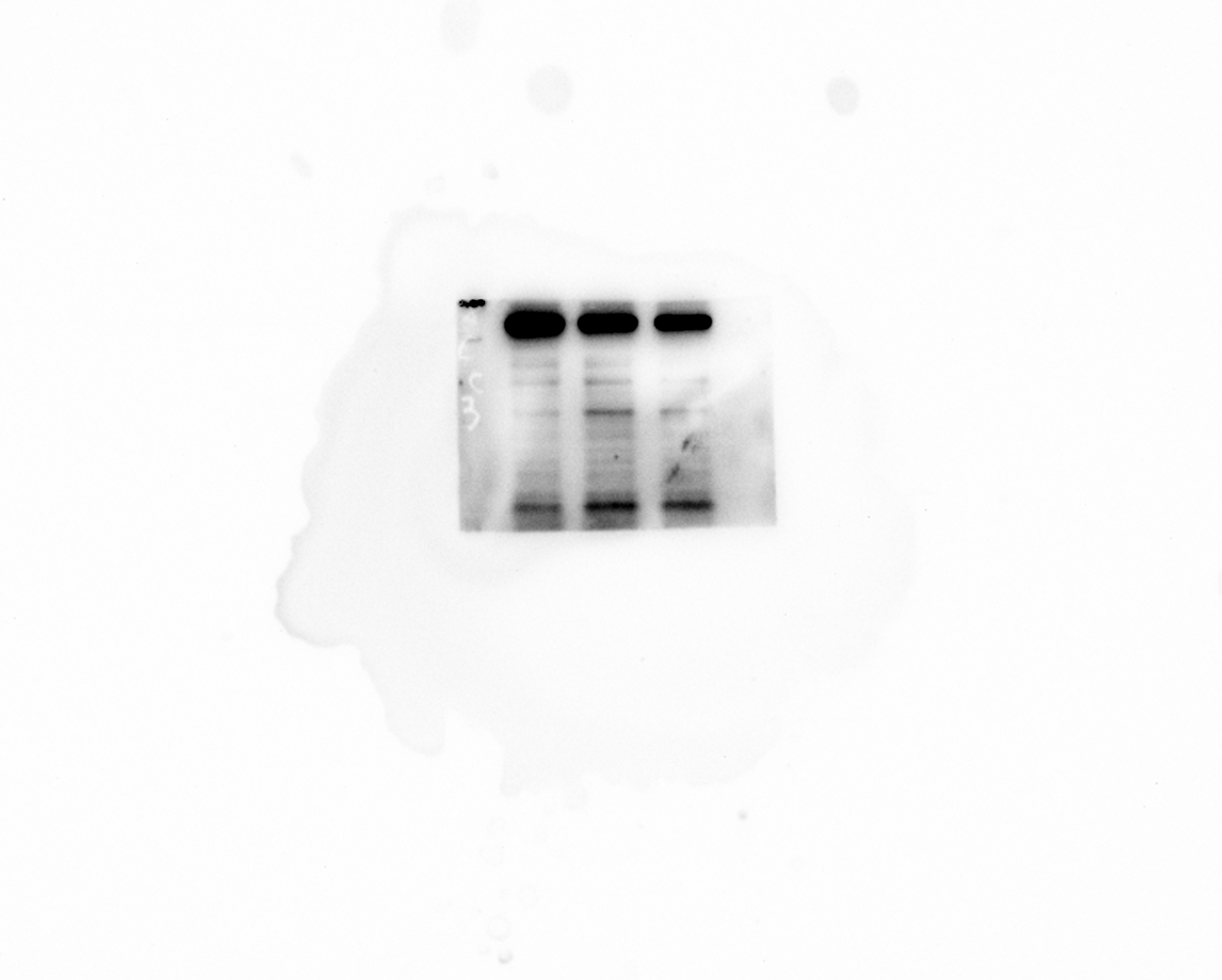

Supplement: Supplementary file 3 [file DataSheet1.ZIP › WB/AMO-1/3 cleaved-caspase3/9.20/AMO1 CC3.Tif]

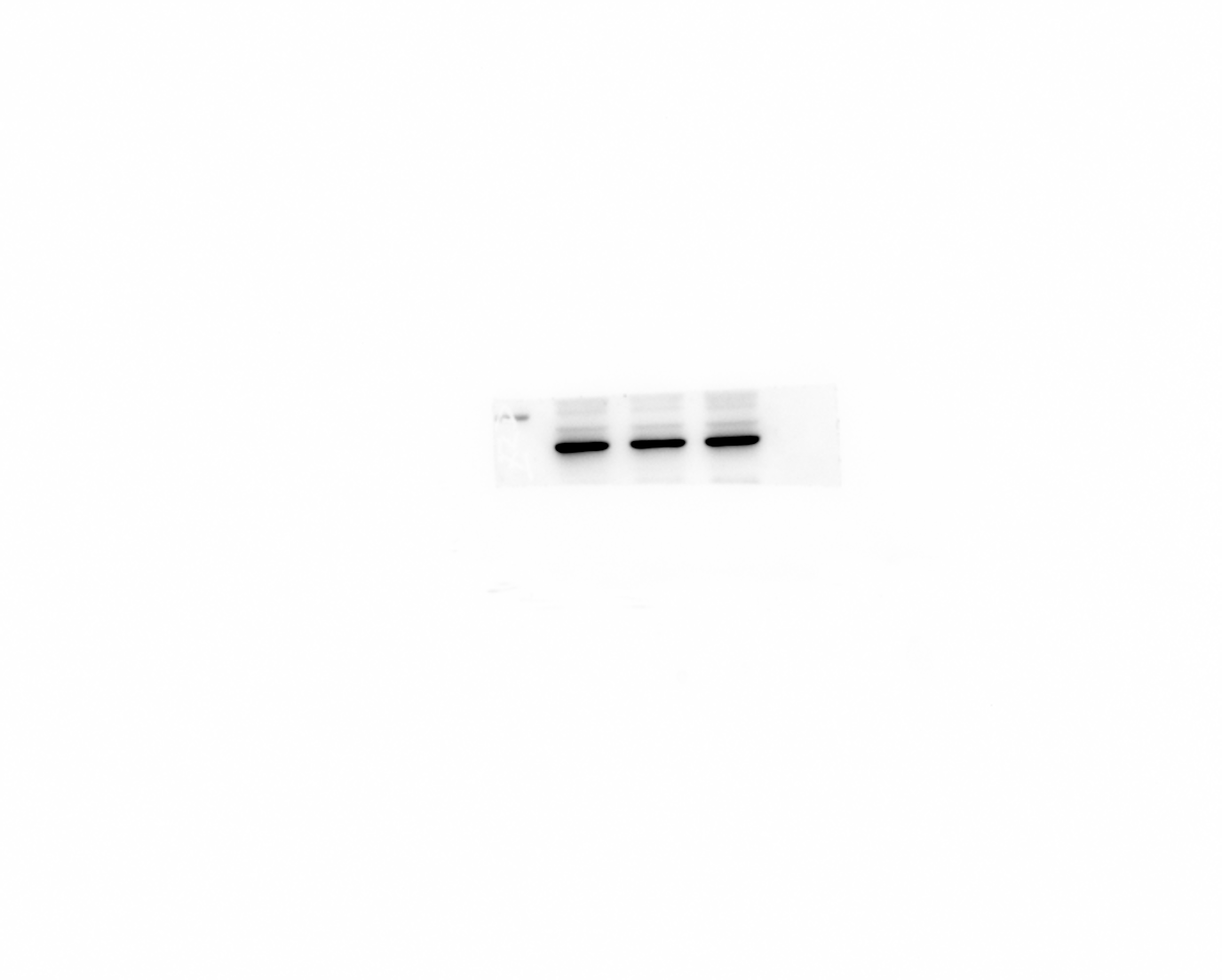

Supplement: Supplementary file 3 [file DataSheet1.ZIP › WB/AMO-1/3 cleaved-caspase3/9.28/Aa┬-actin.Tif]

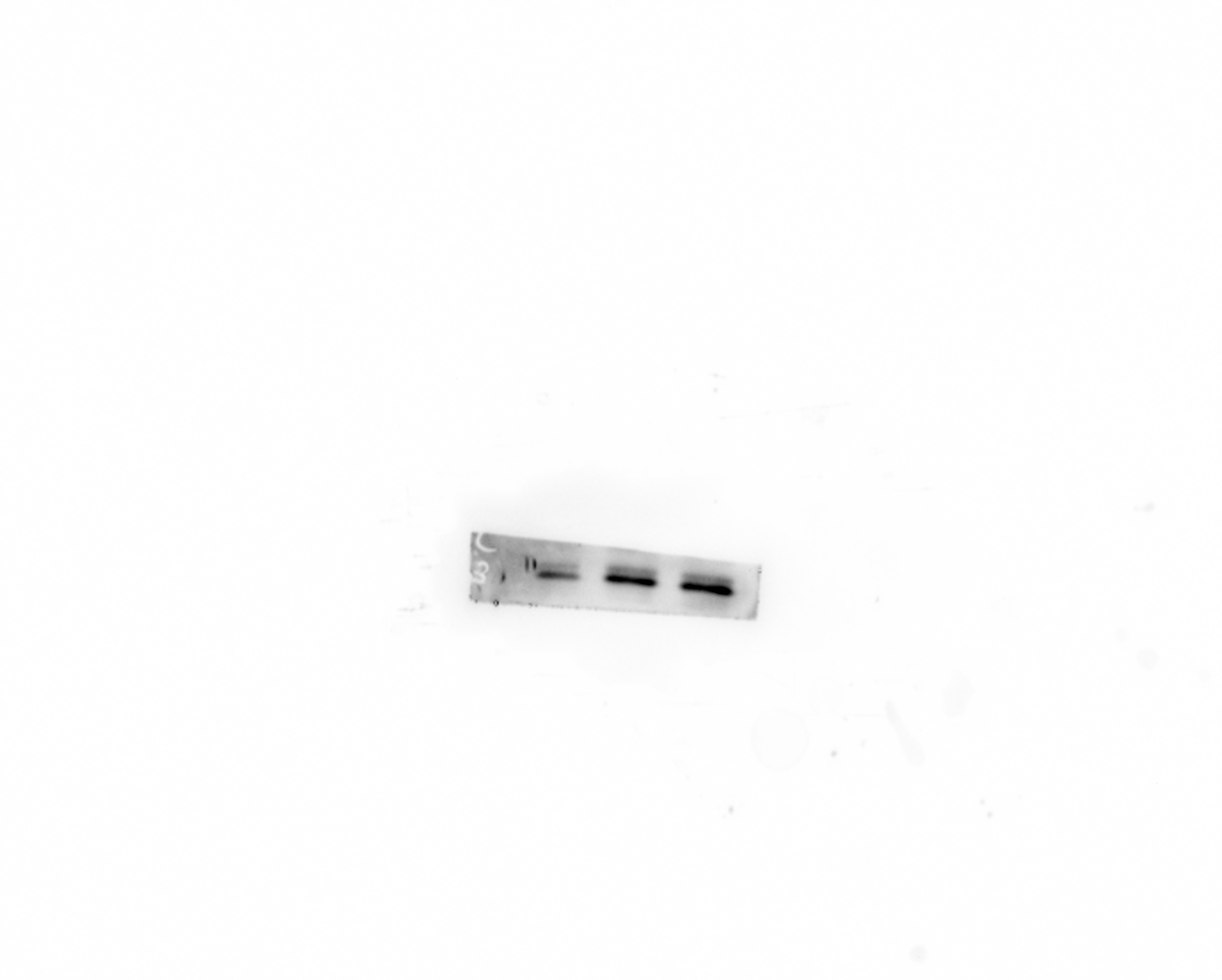

Supplement: Supplementary file 3 [file DataSheet1.ZIP › WB/AMO-1/3 cleaved-caspase3/9.28/CC3 1.Tif]

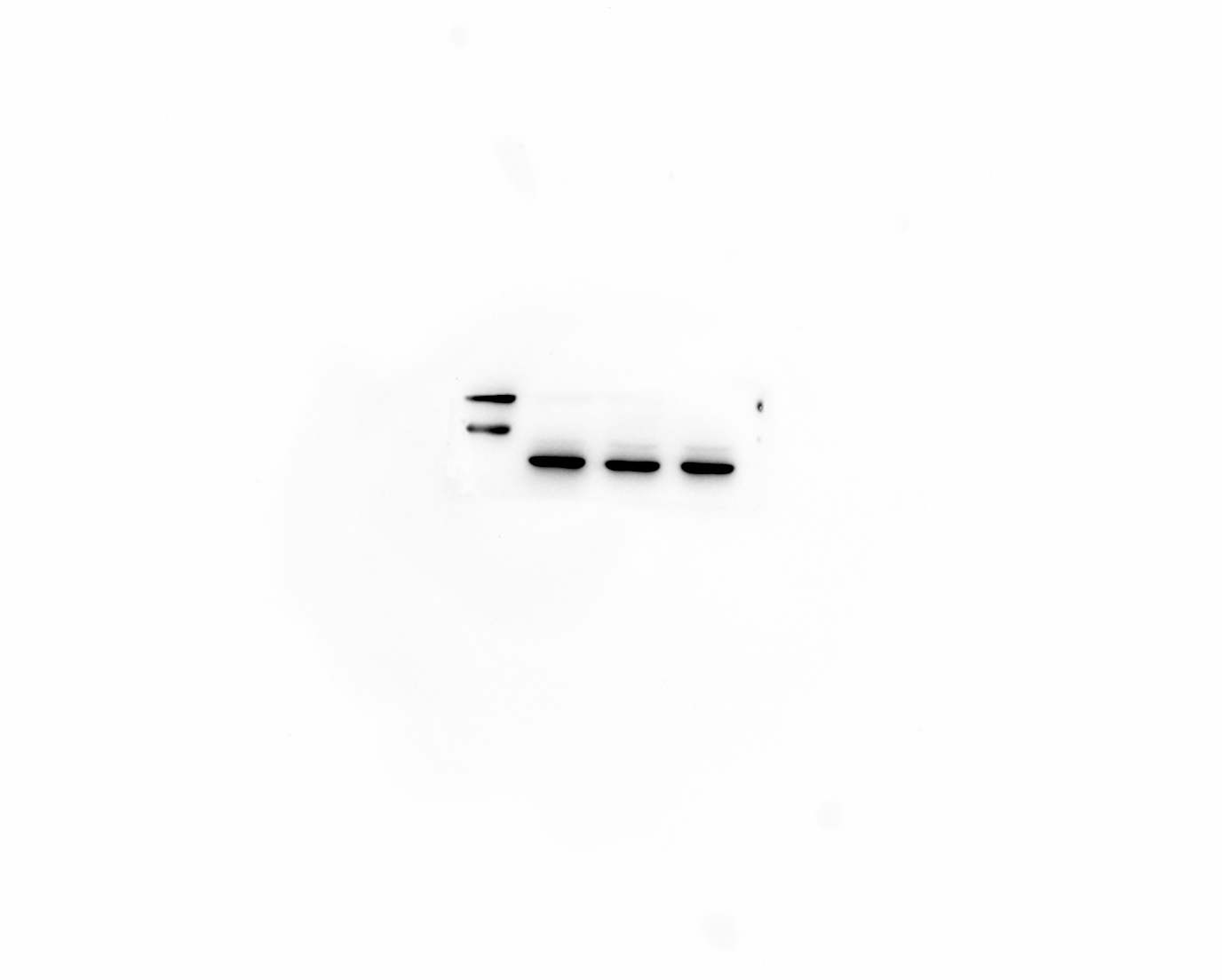

Supplement: Supplementary file 3 [file DataSheet1.ZIP › WB/AMO-1/3 cleaved-caspase3/9.29/8a┬-actin.Tif]

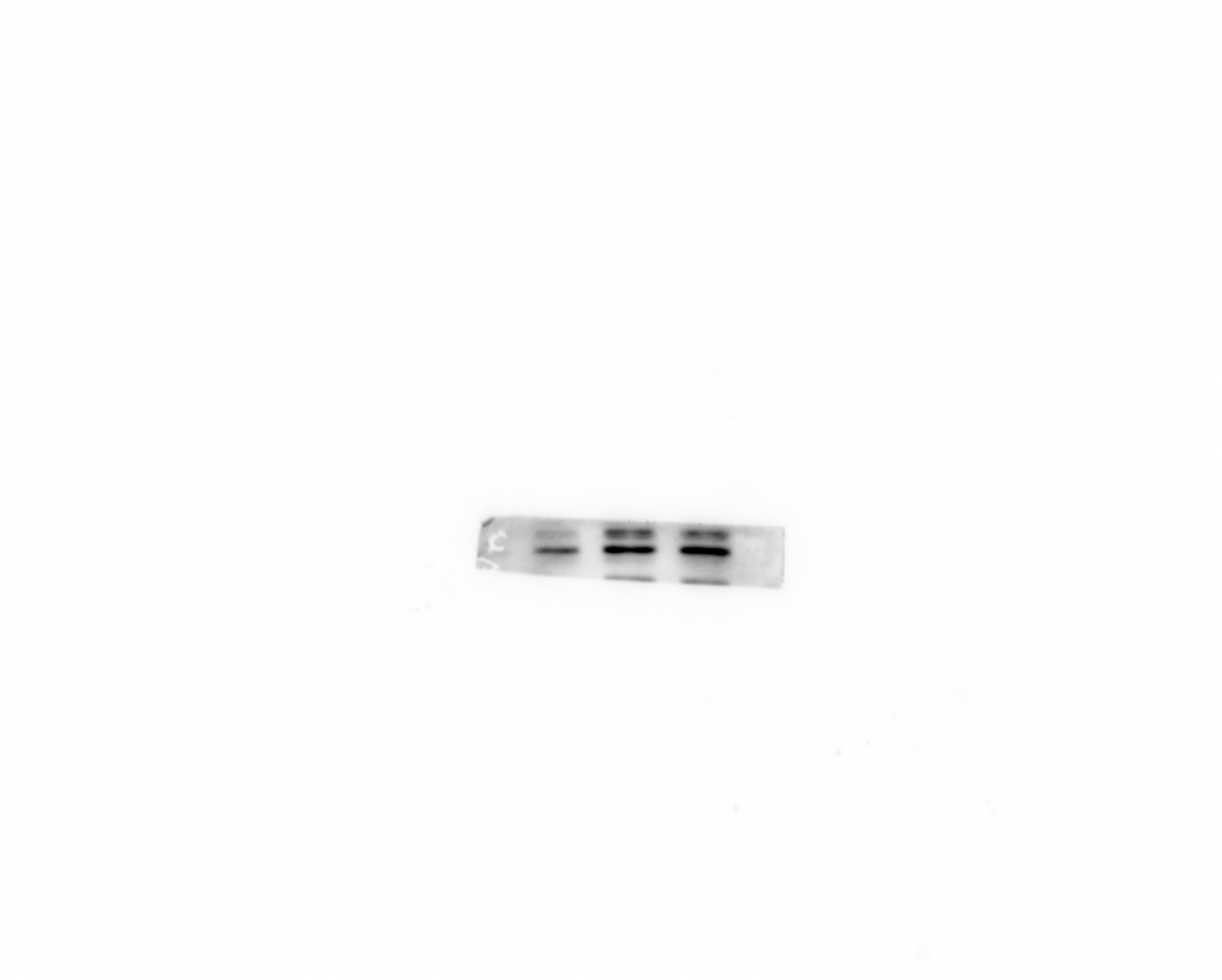

Supplement: Supplementary file 3 [file DataSheet1.ZIP › WB/AMO-1/3 cleaved-caspase3/9.29/CC3.Tif]

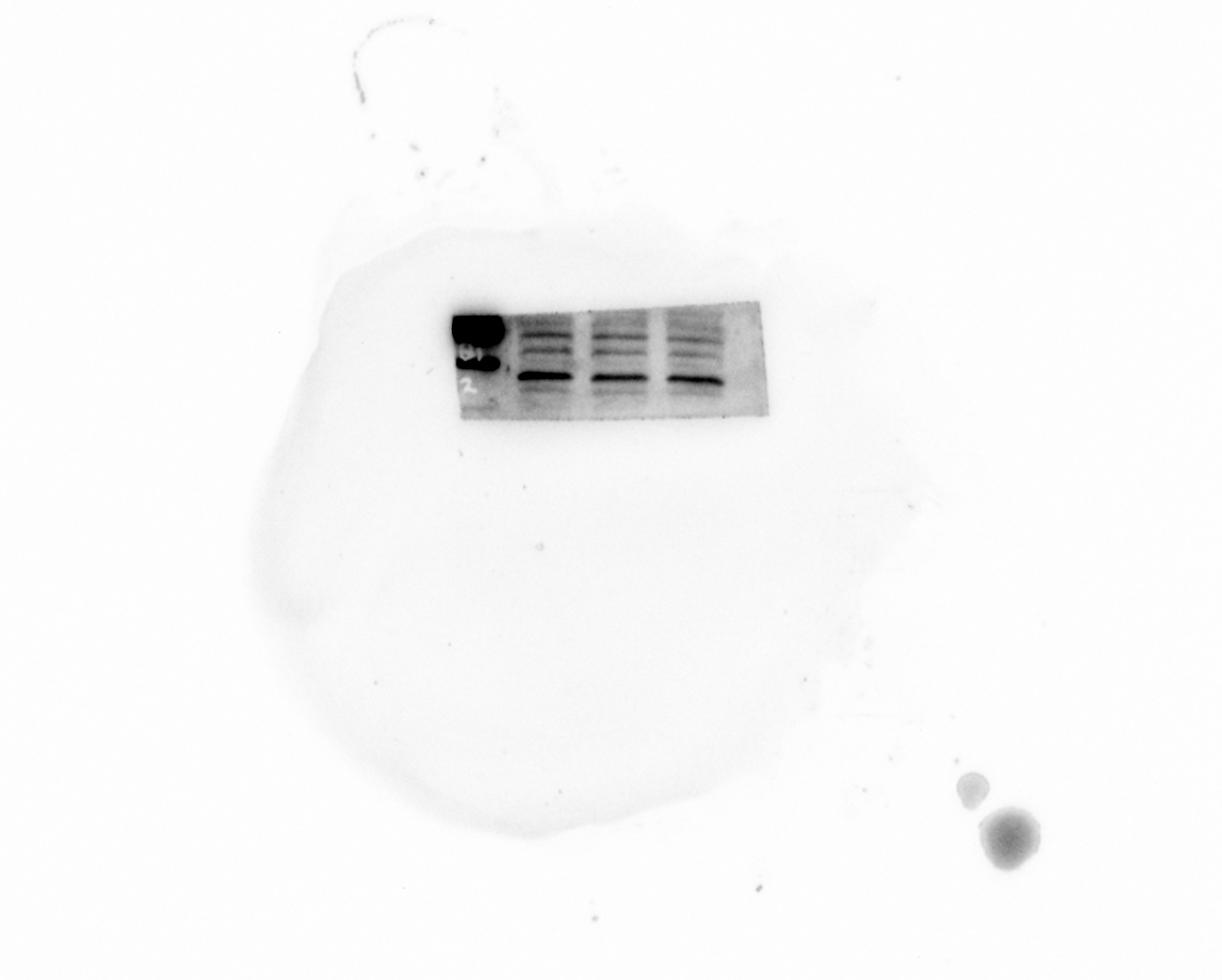

Supplement: Supplementary file 3 [file DataSheet1.ZIP › WB/AMO-1/4 CCNB1/10.5/B1 2.Tif]

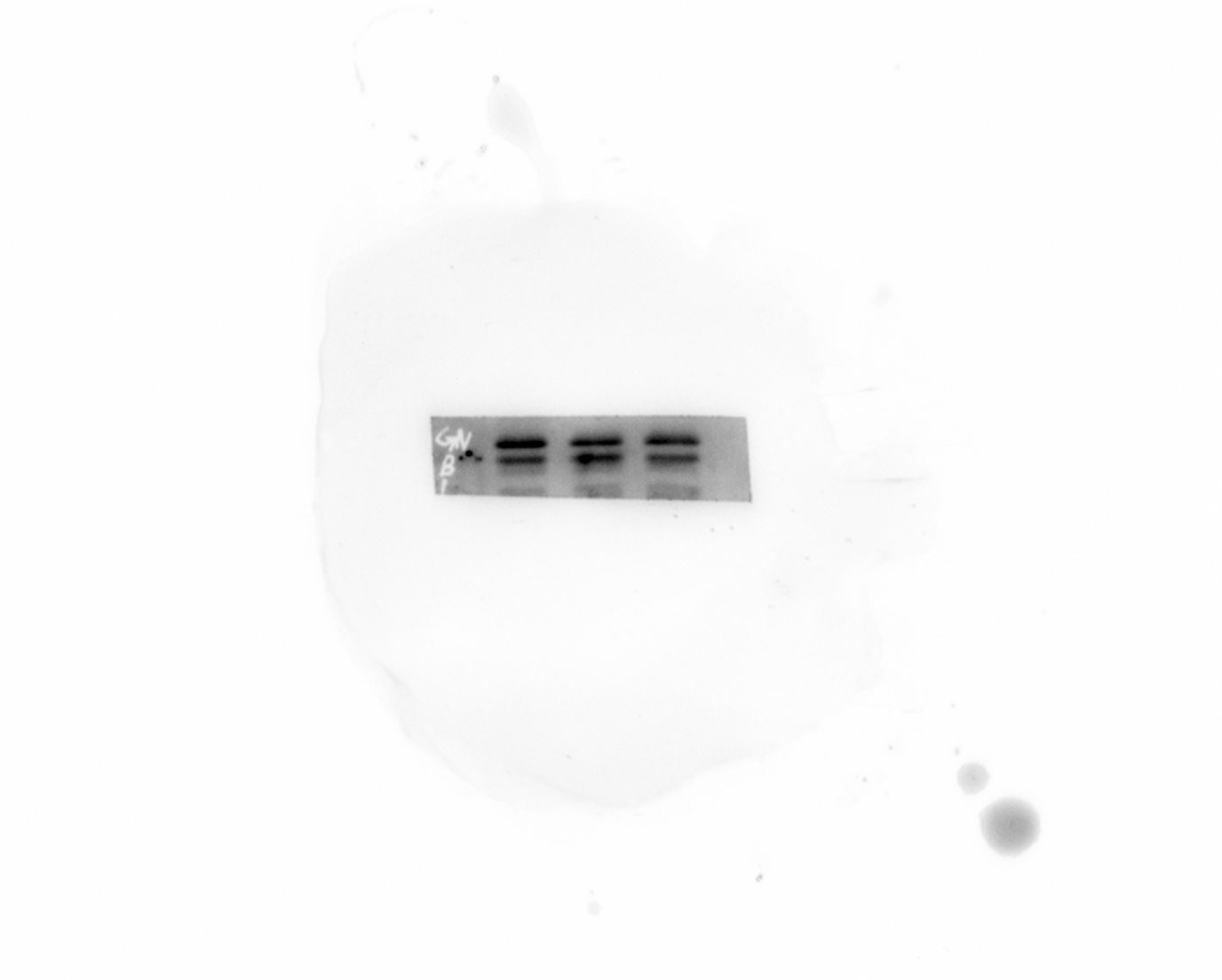

Supplement: Supplementary file 3 [file DataSheet1.ZIP › WB/AMO-1/4 CCNB1/10.5/gapdh.Tif]

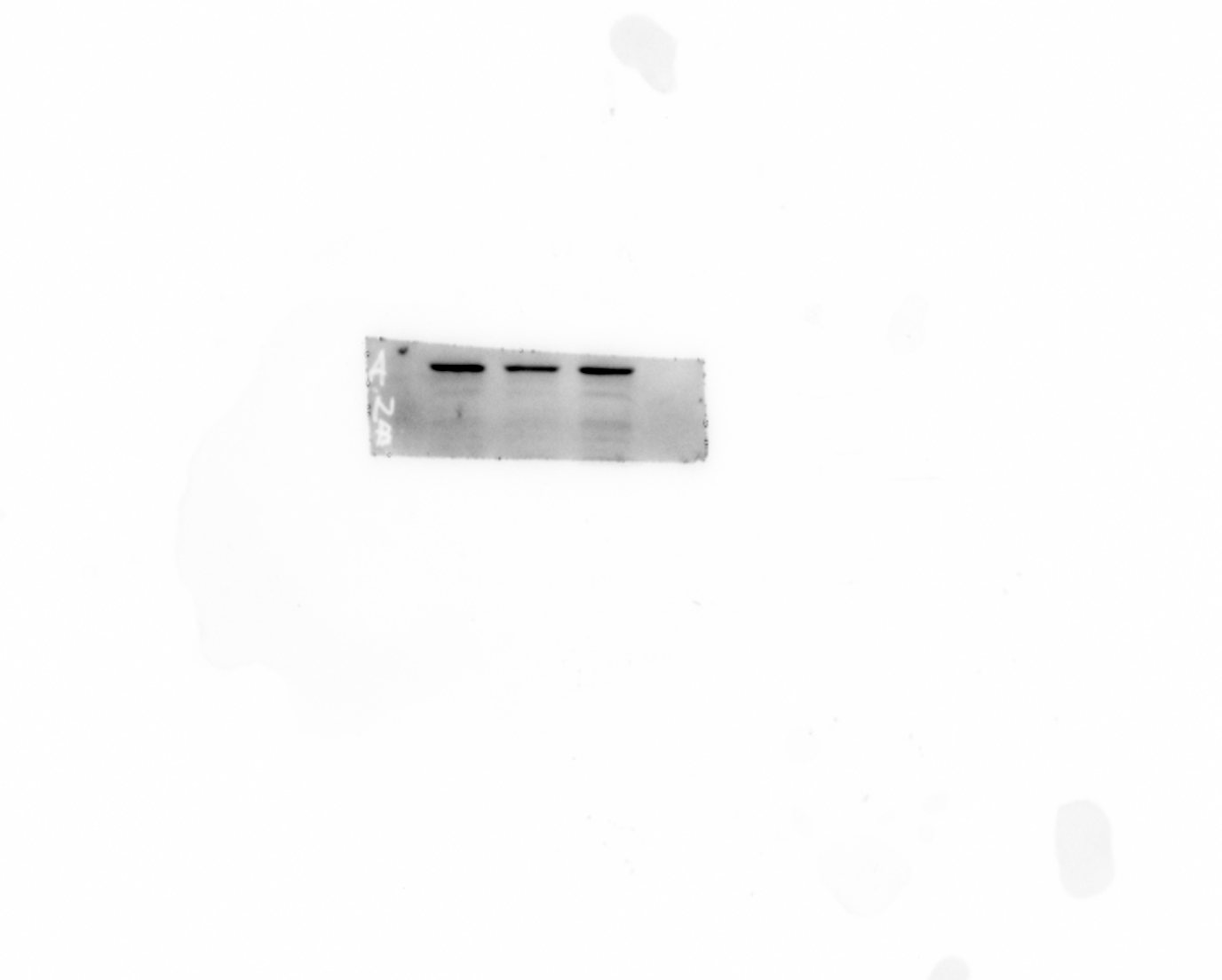

Supplement: Supplementary file 3 [file DataSheet1.ZIP › WB/AMO-1/4 CCNB1/9.28/A GAPDH B1.Tif]

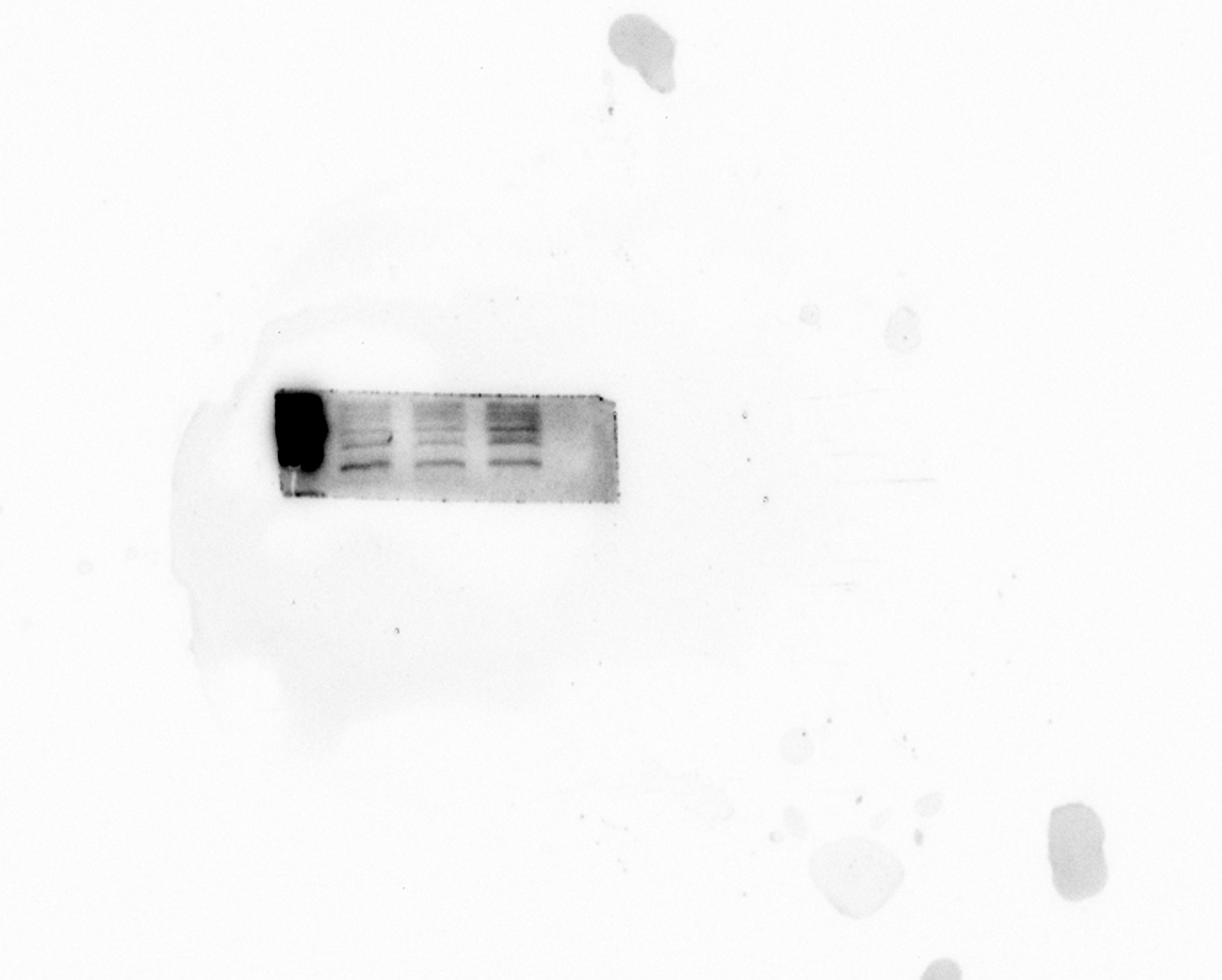

Supplement: Supplementary file 3 [file DataSheet1.ZIP › WB/AMO-1/4 CCNB1/9.28/ACCNB1.Tif]

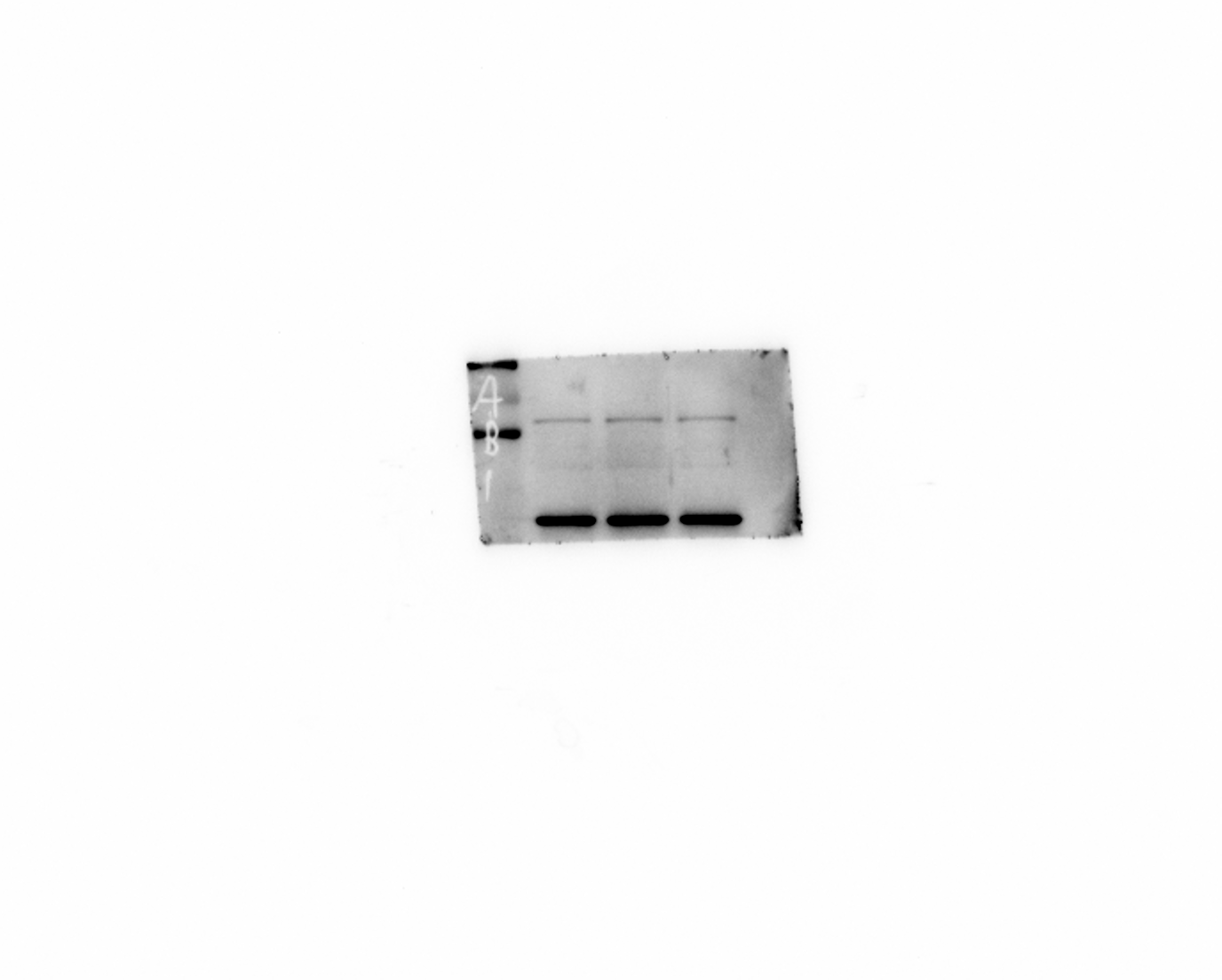

Supplement: Supplementary file 3 [file DataSheet1.ZIP › WB/AMO-1/4 CCNB1/9.30/ACCNB1 DAMO 1.Tif]

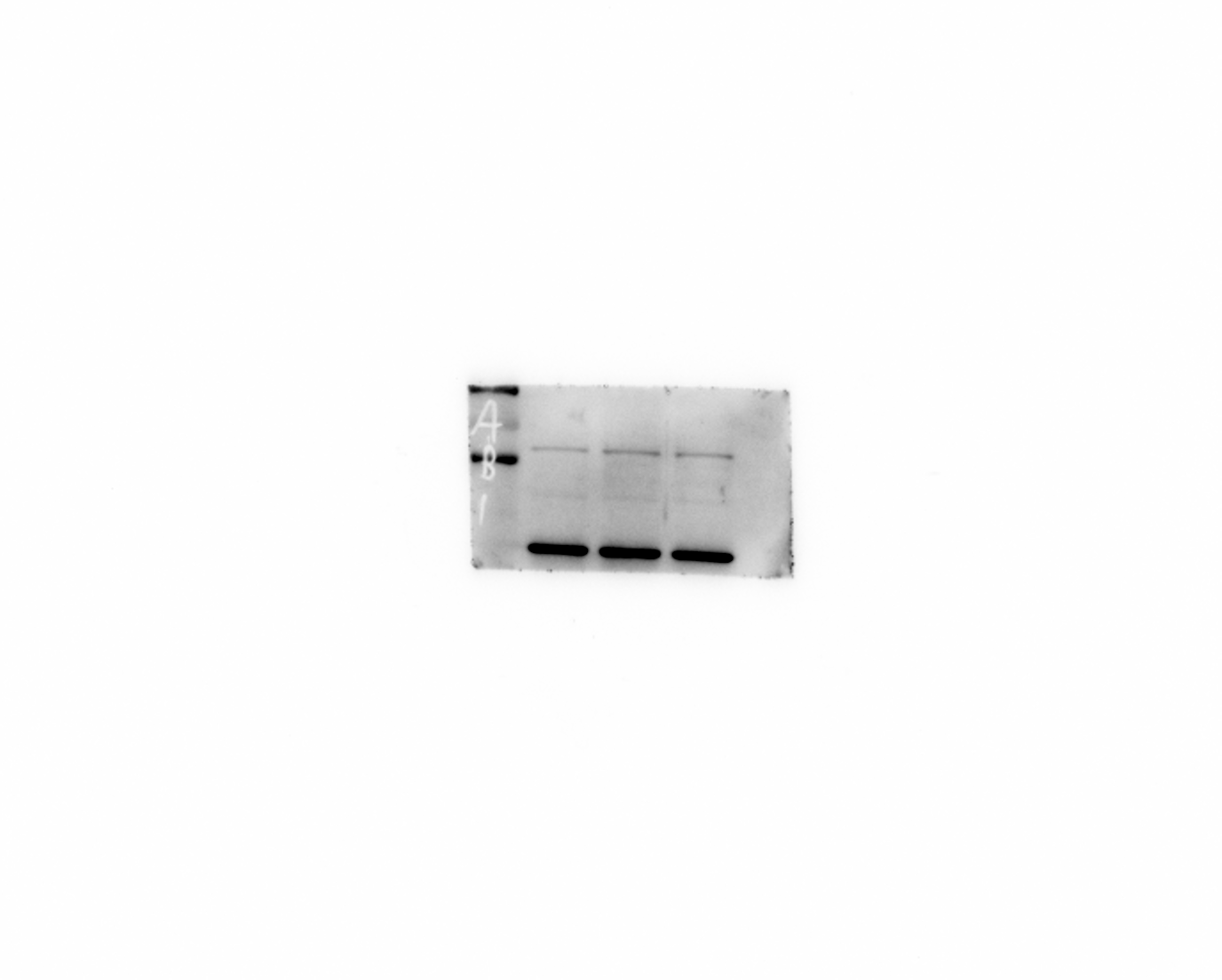

Supplement: Supplementary file 3 [file DataSheet1.ZIP › WB/AMO-1/4 CCNB1/9.30/ACCNB1 DAMO.Tif]

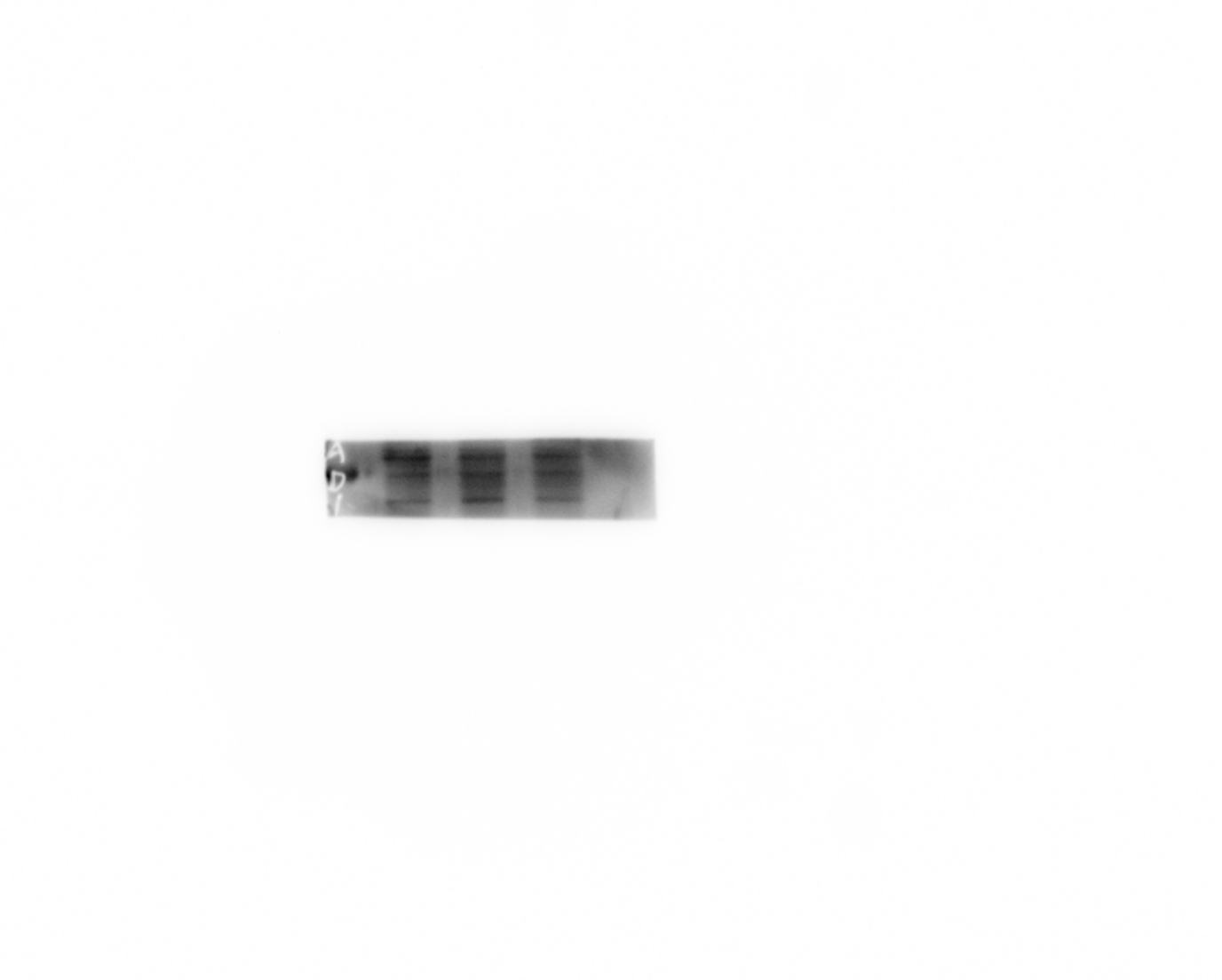

Supplement: Supplementary file 3 [file DataSheet1.ZIP › WB/AMO-1/5 CCND1/9.22/AD1 1.Tif]

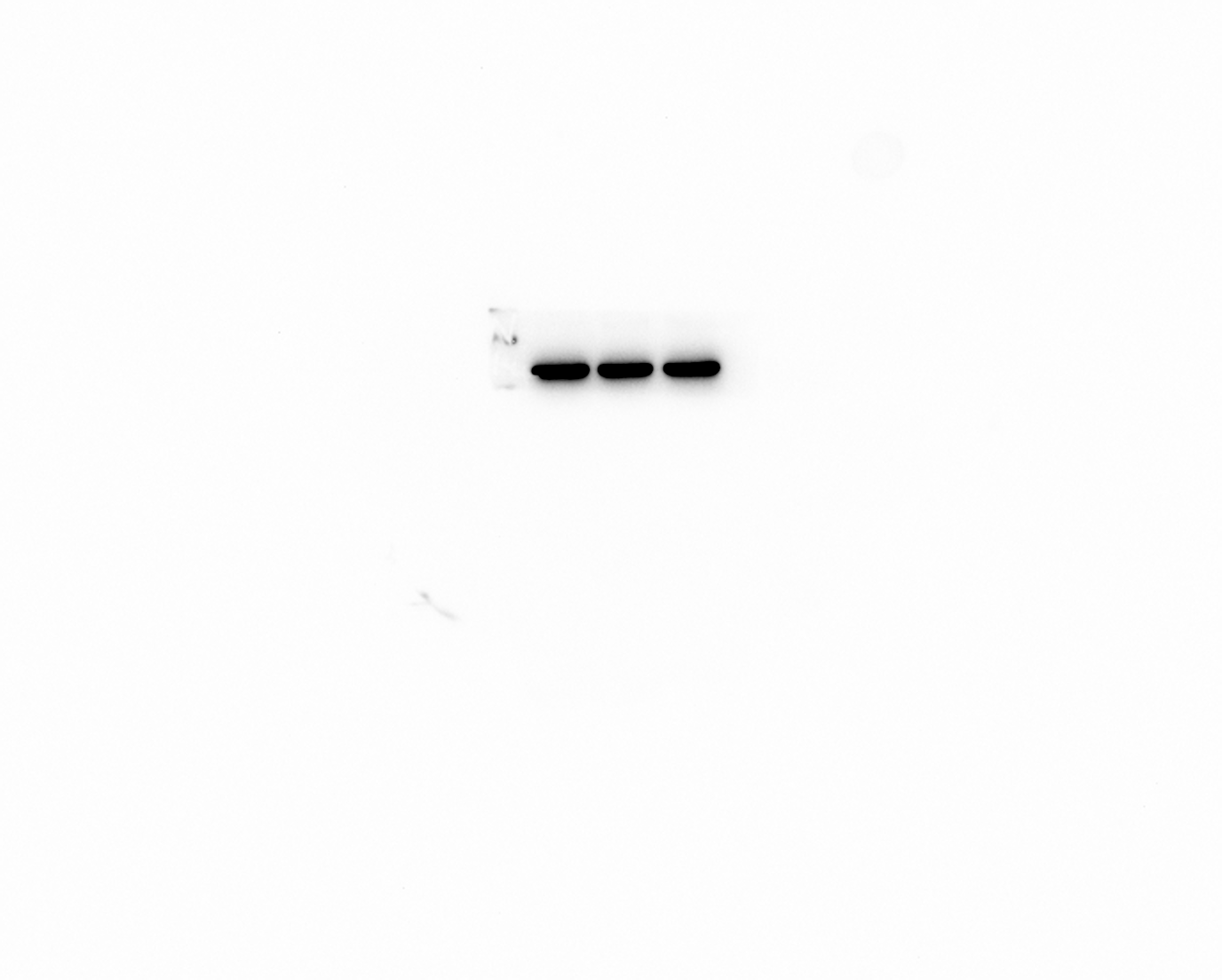

Supplement: Supplementary file 3 [file DataSheet1.ZIP › WB/AMO-1/5 CCND1/9.22/gapdh.Tif]

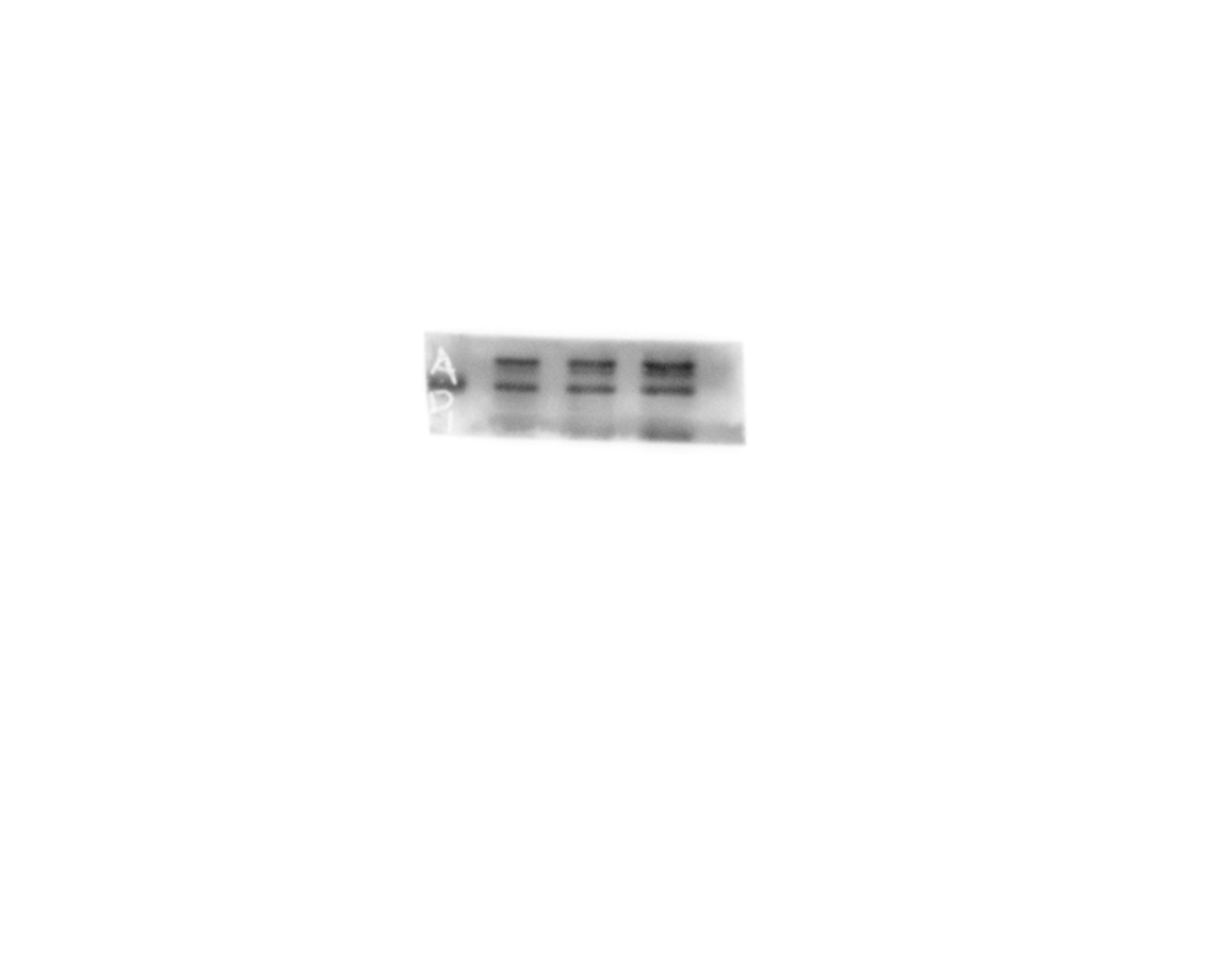

Supplement: Supplementary file 3 [file DataSheet1.ZIP › WB/AMO-1/5 CCND1/9.24/AD1.Tif]

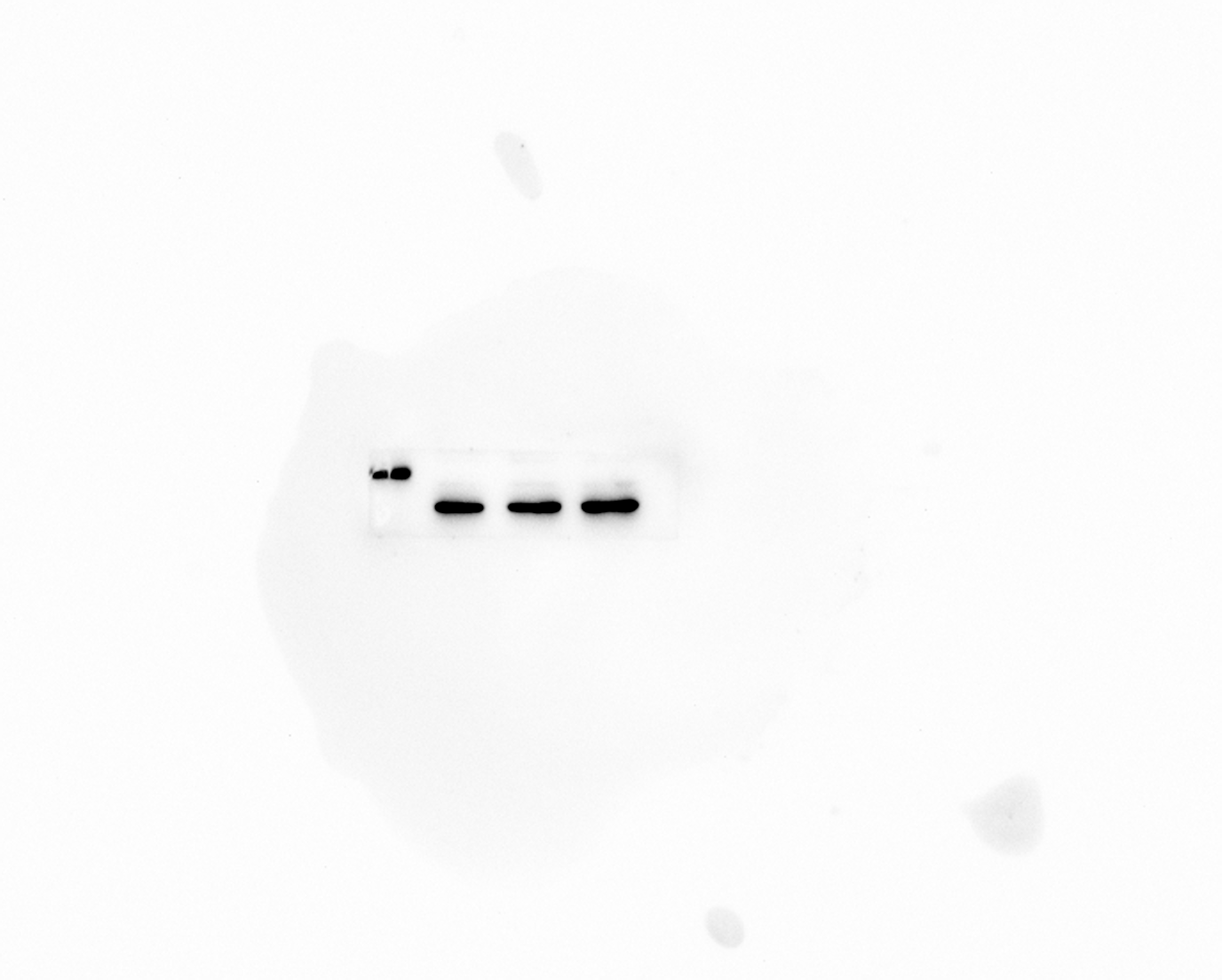

Supplement: Supplementary file 3 [file DataSheet1.ZIP › WB/AMO-1/5 CCND1/9.24/gapdh.Tif]

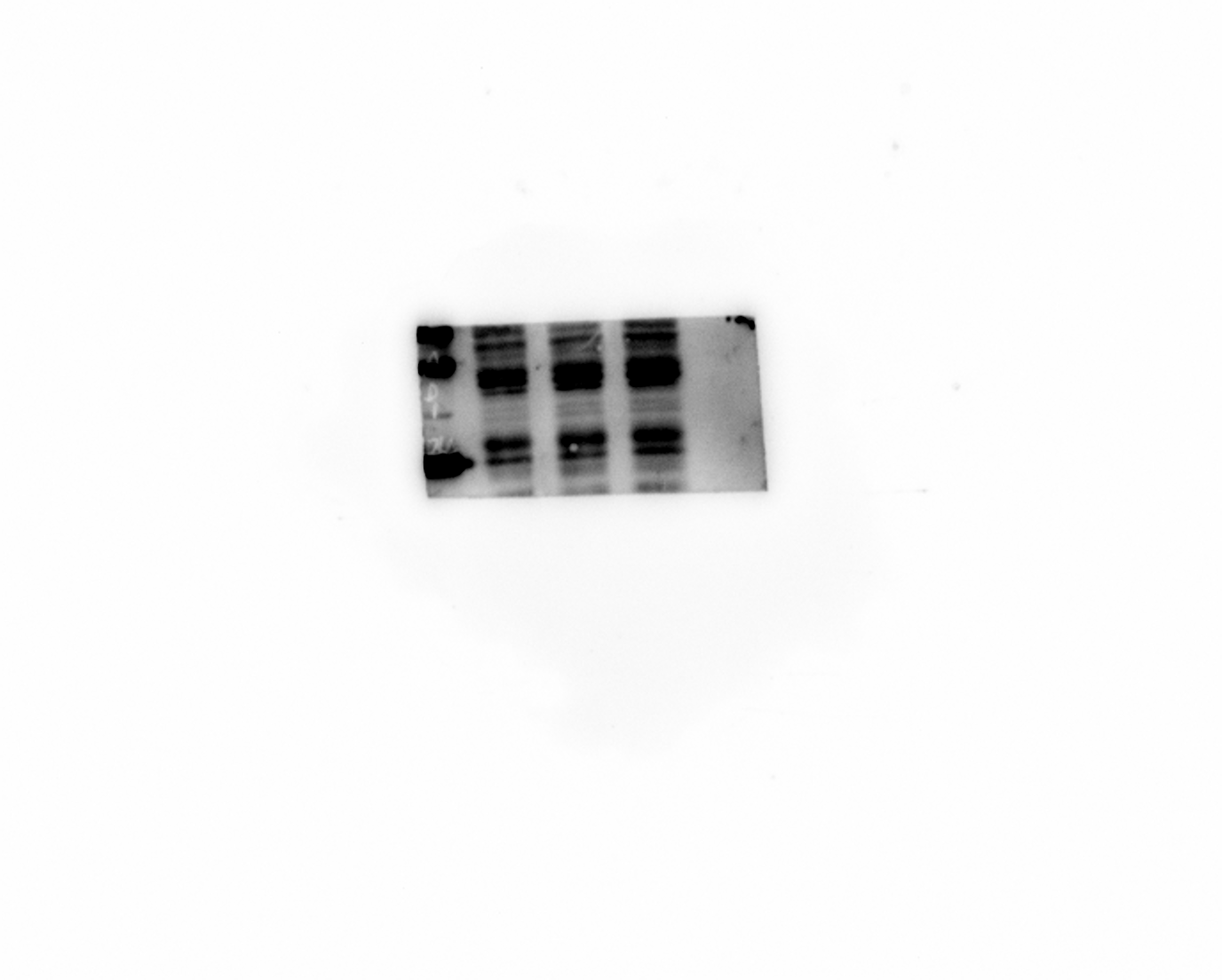

Supplement: Supplementary file 3 [file DataSheet1.ZIP › WB/AMO-1/5 CCND1/9.26/A CCND1.Tif]

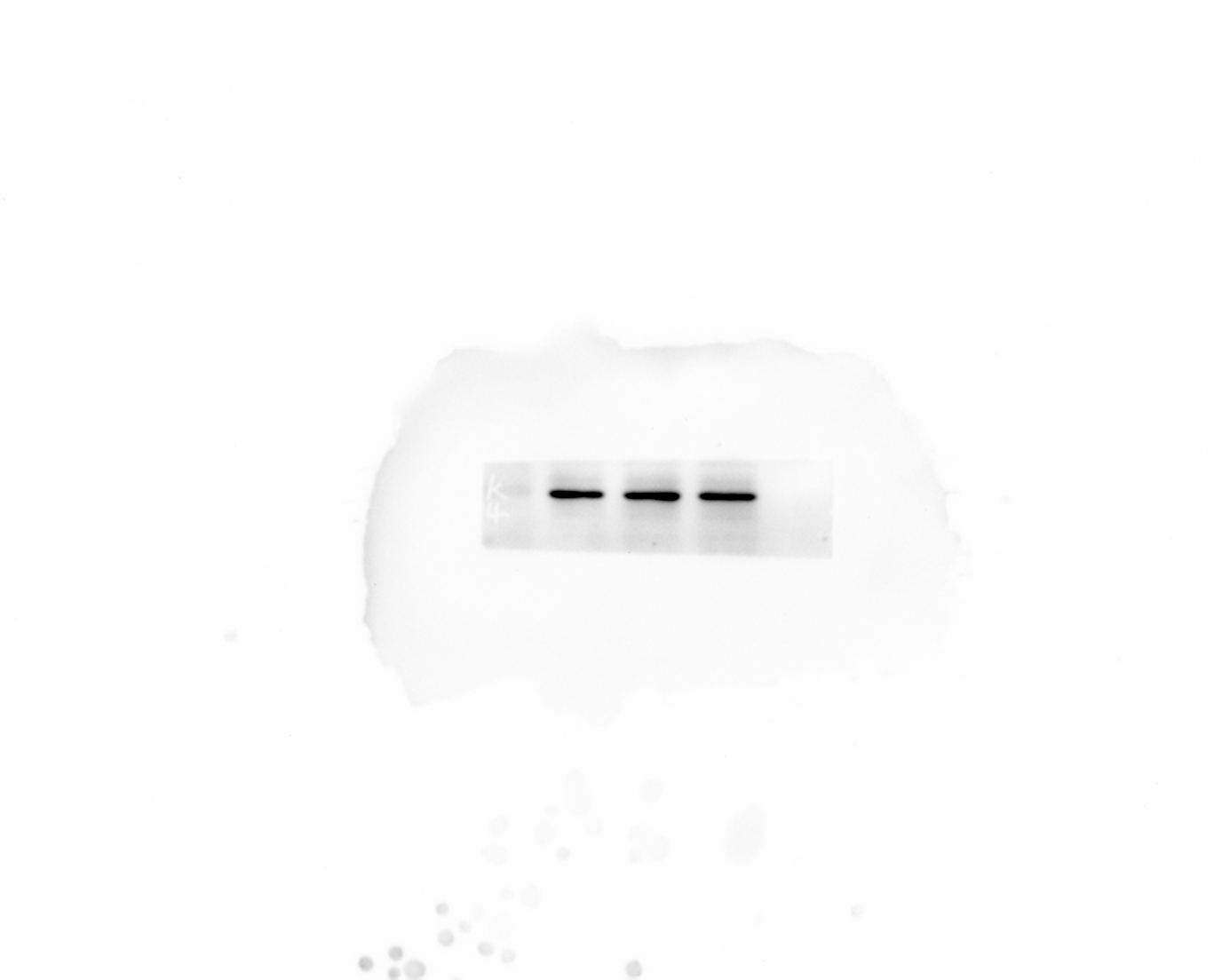

Supplement: Supplementary file 3 [file DataSheet1.ZIP › WB/AMO-1/6 CDK4/9.20/CDK4.Tif]

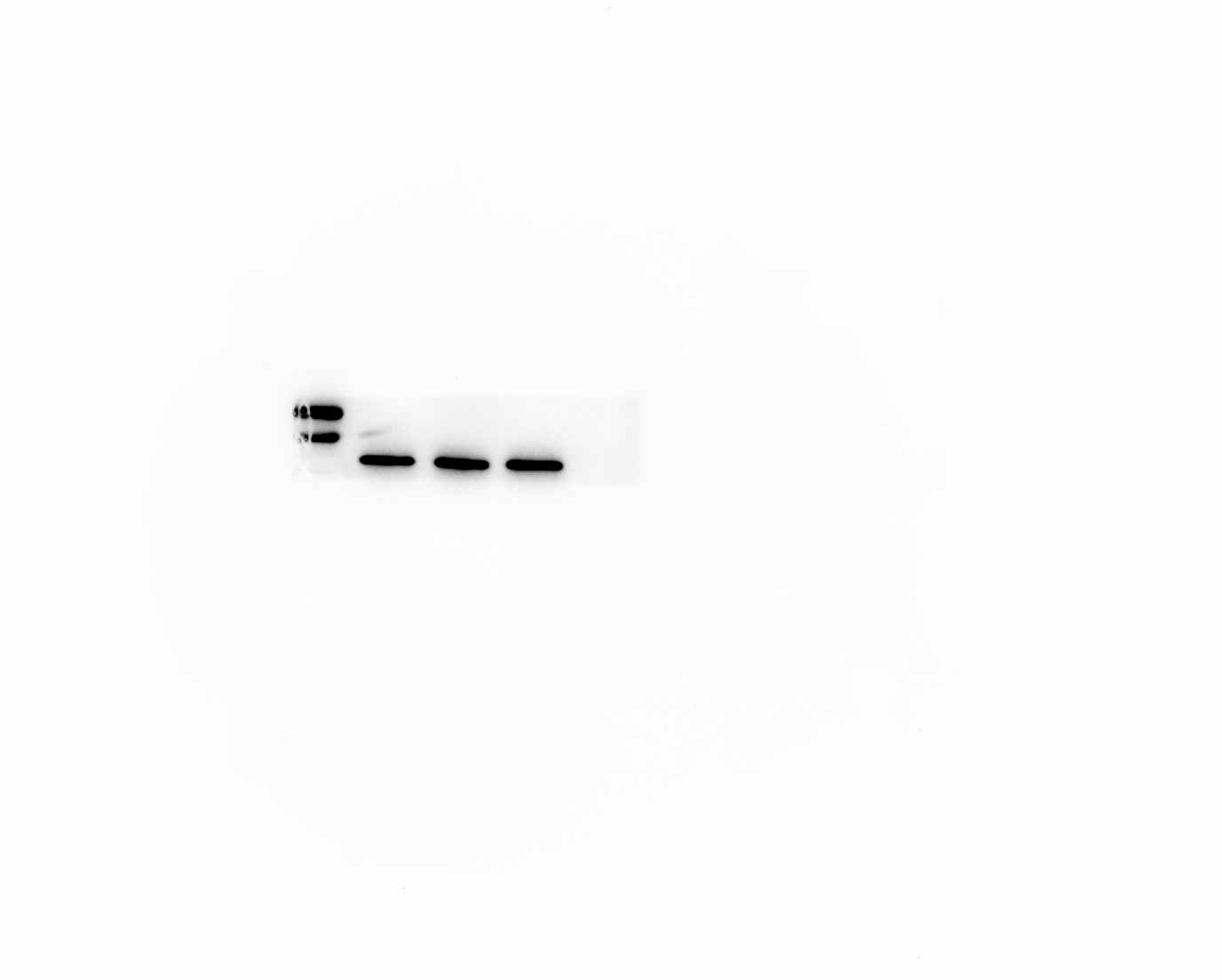

Supplement: Supplementary file 3 [file DataSheet1.ZIP › WB/AMO-1/6 CDK4/9.20/gapdh.Tif]

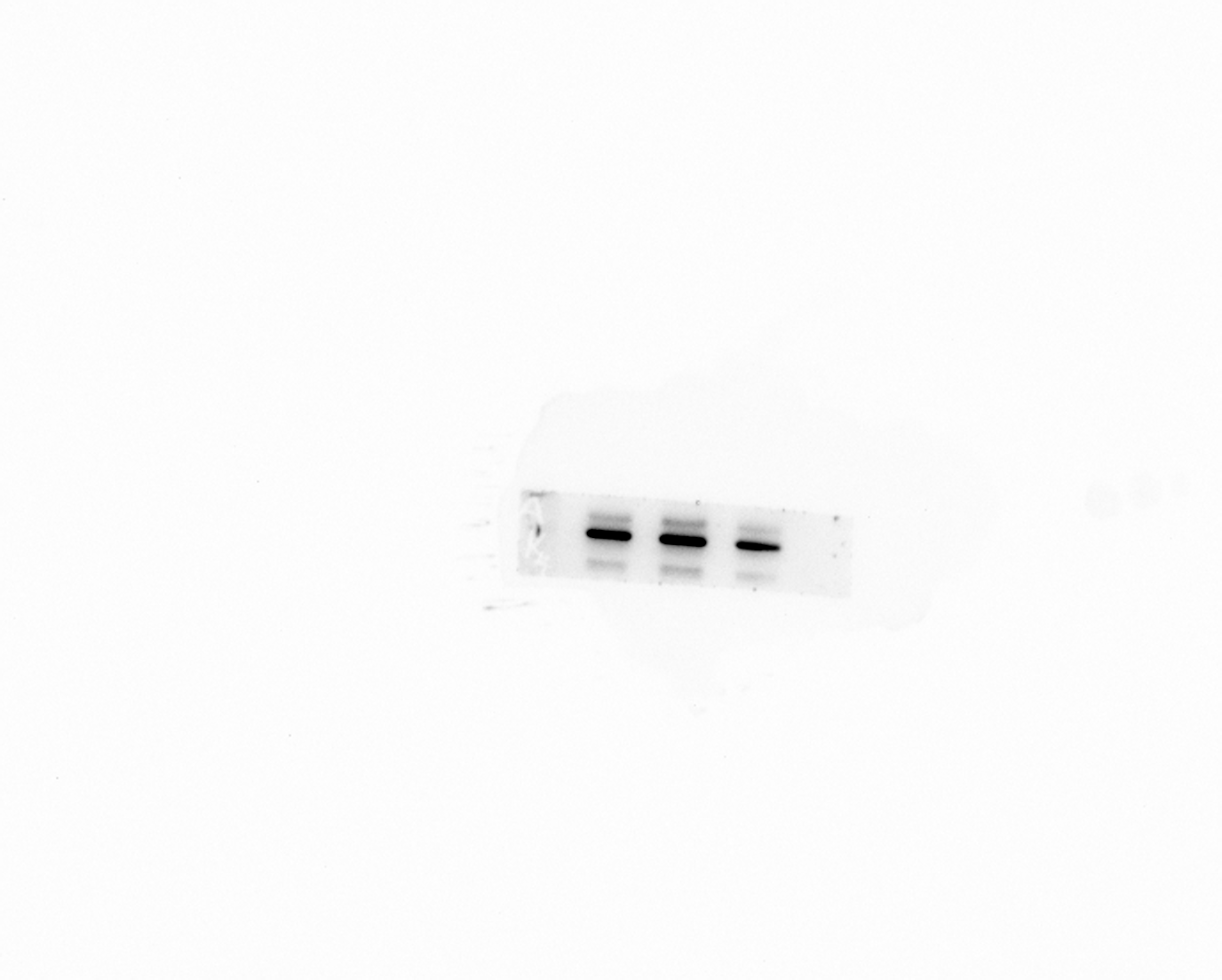

Supplement: Supplementary file 3 [file DataSheet1.ZIP › WB/AMO-1/6 CDK4/9.21/AK4.Tif]

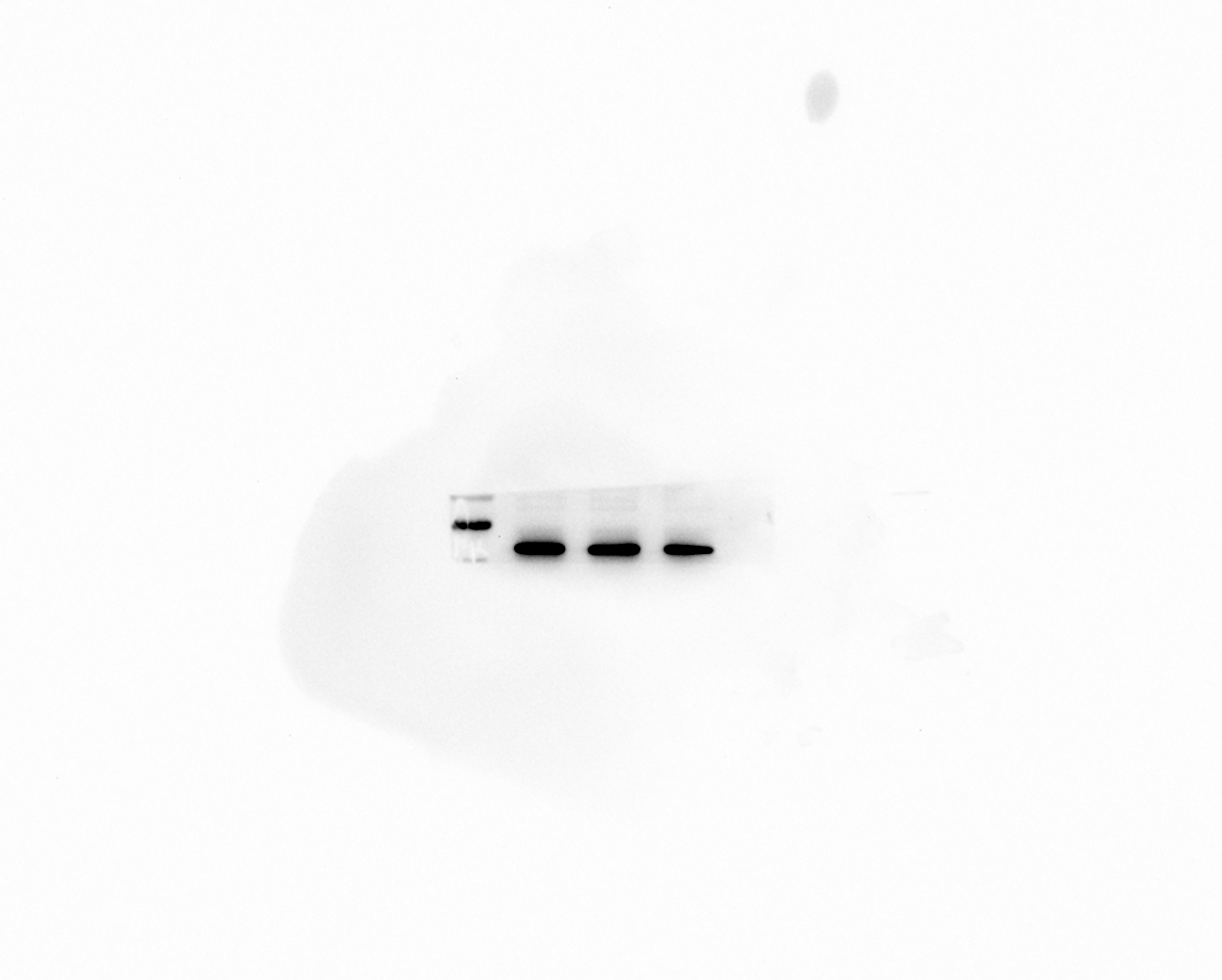

Supplement: Supplementary file 3 [file DataSheet1.ZIP › WB/AMO-1/6 CDK4/9.21/Agapdh.Tif]

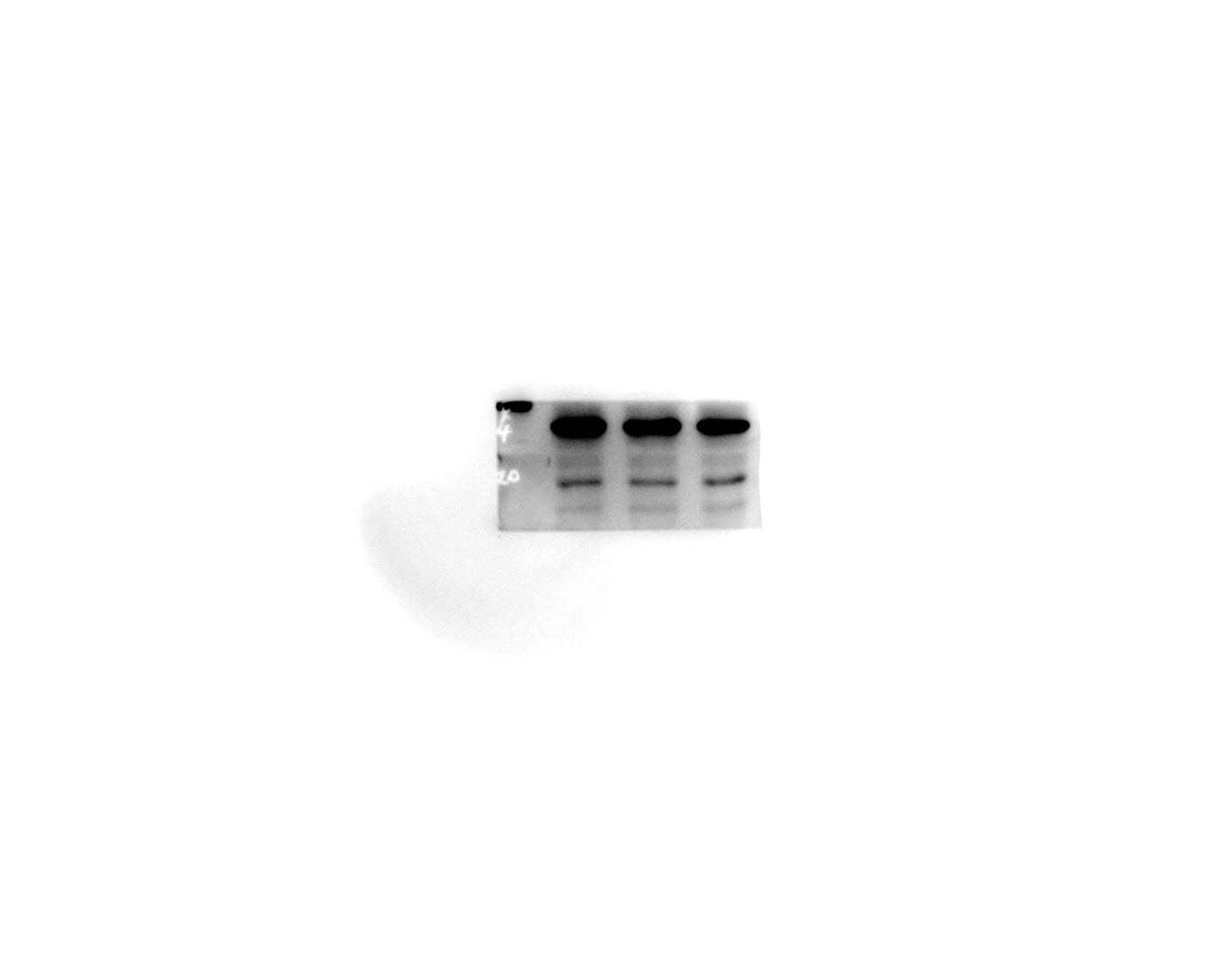

Supplement: Supplementary file 3 [file DataSheet1.ZIP › WB/AMO-1/6 CDK4/9.22/CDK4 2.Tif]

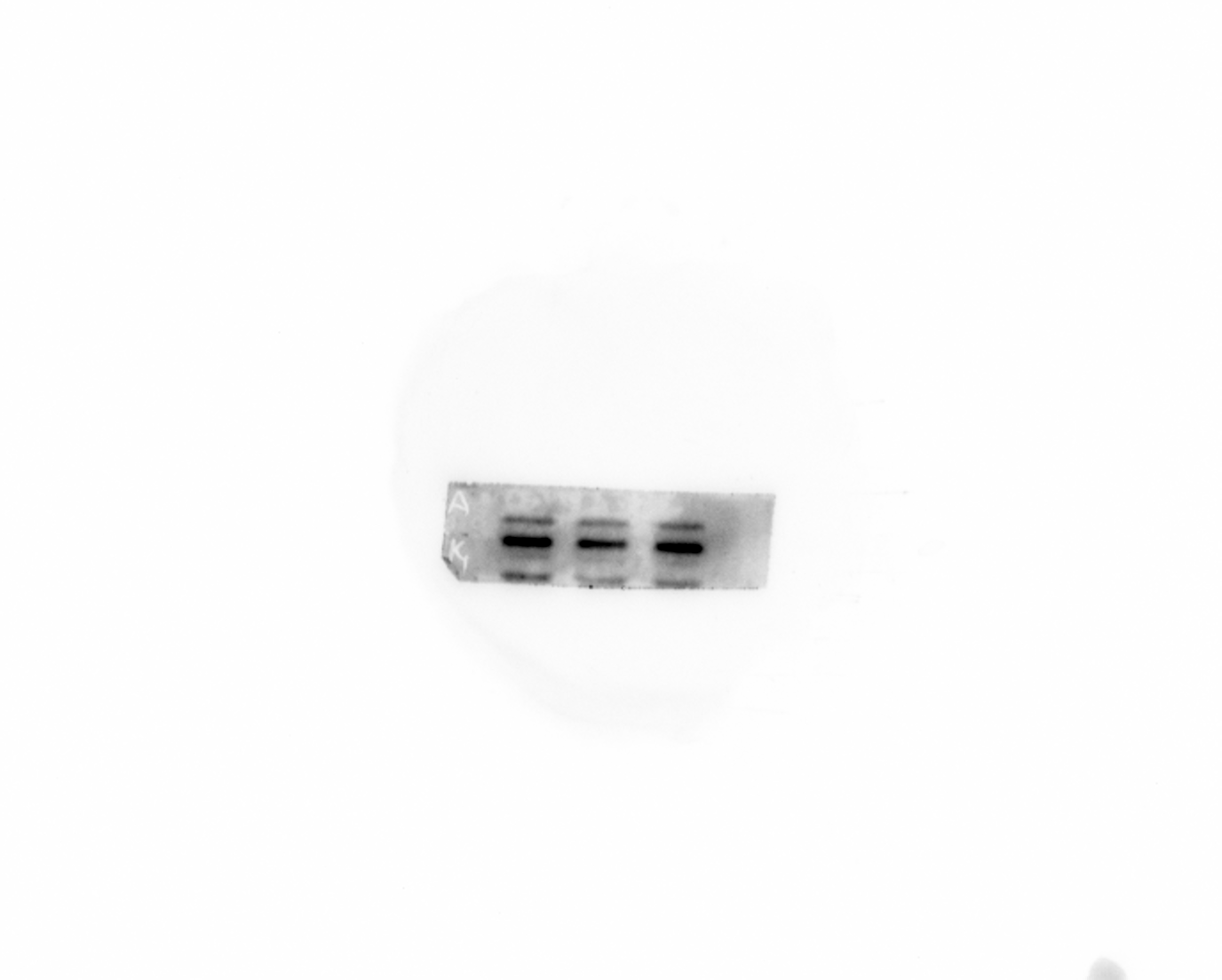

Supplement: Supplementary file 3 [file DataSheet1.ZIP › WB/AMO-1/7 p-cdk1/10.3/A K1.Tif]

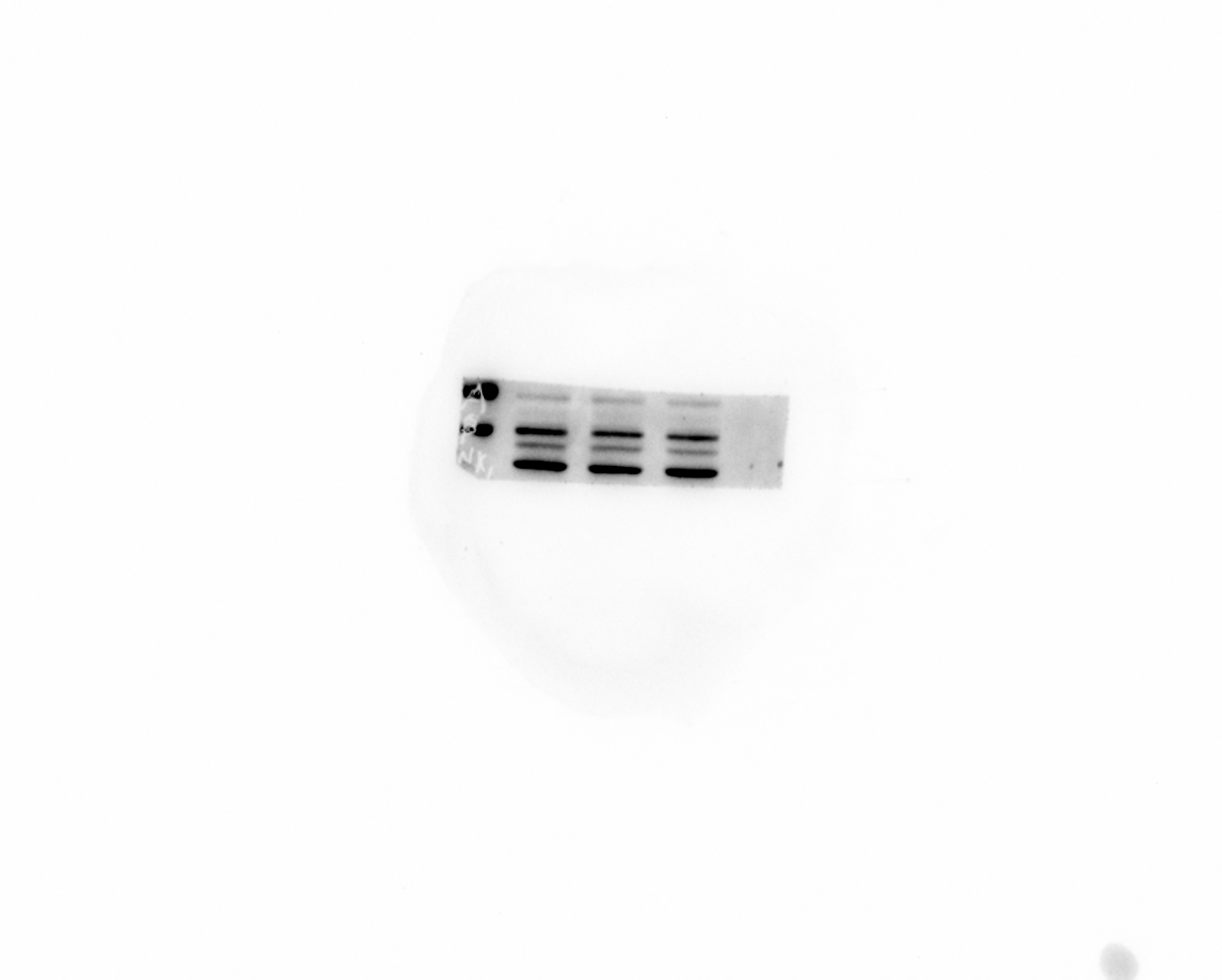

Supplement: Supplementary file 3 [file DataSheet1.ZIP › WB/AMO-1/7 p-cdk1/10.3/Agapdh.Tif]

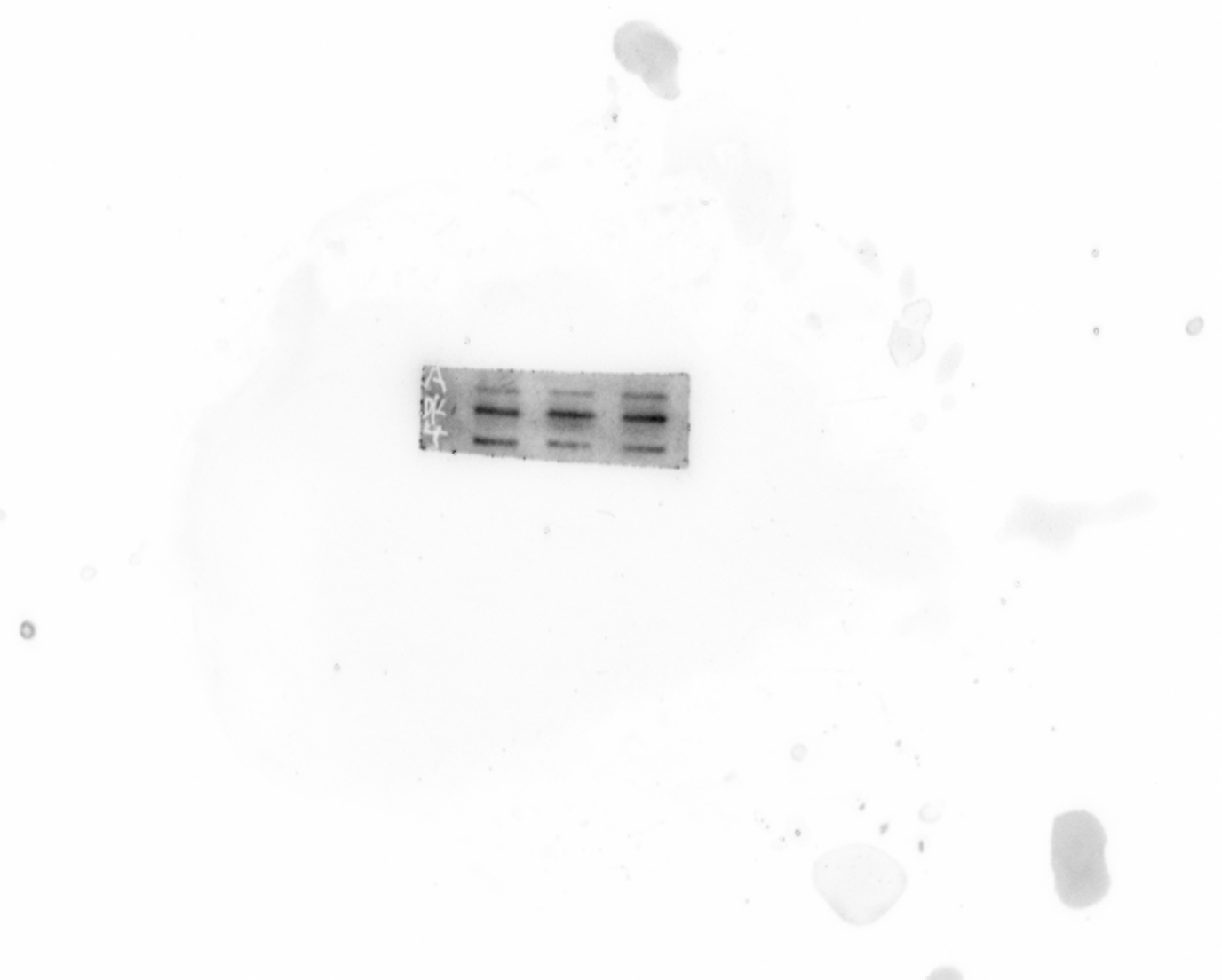

Supplement: Supplementary file 3 [file DataSheet1.ZIP › WB/AMO-1/7 p-cdk1/9.28/ACDK1 4.Tif]

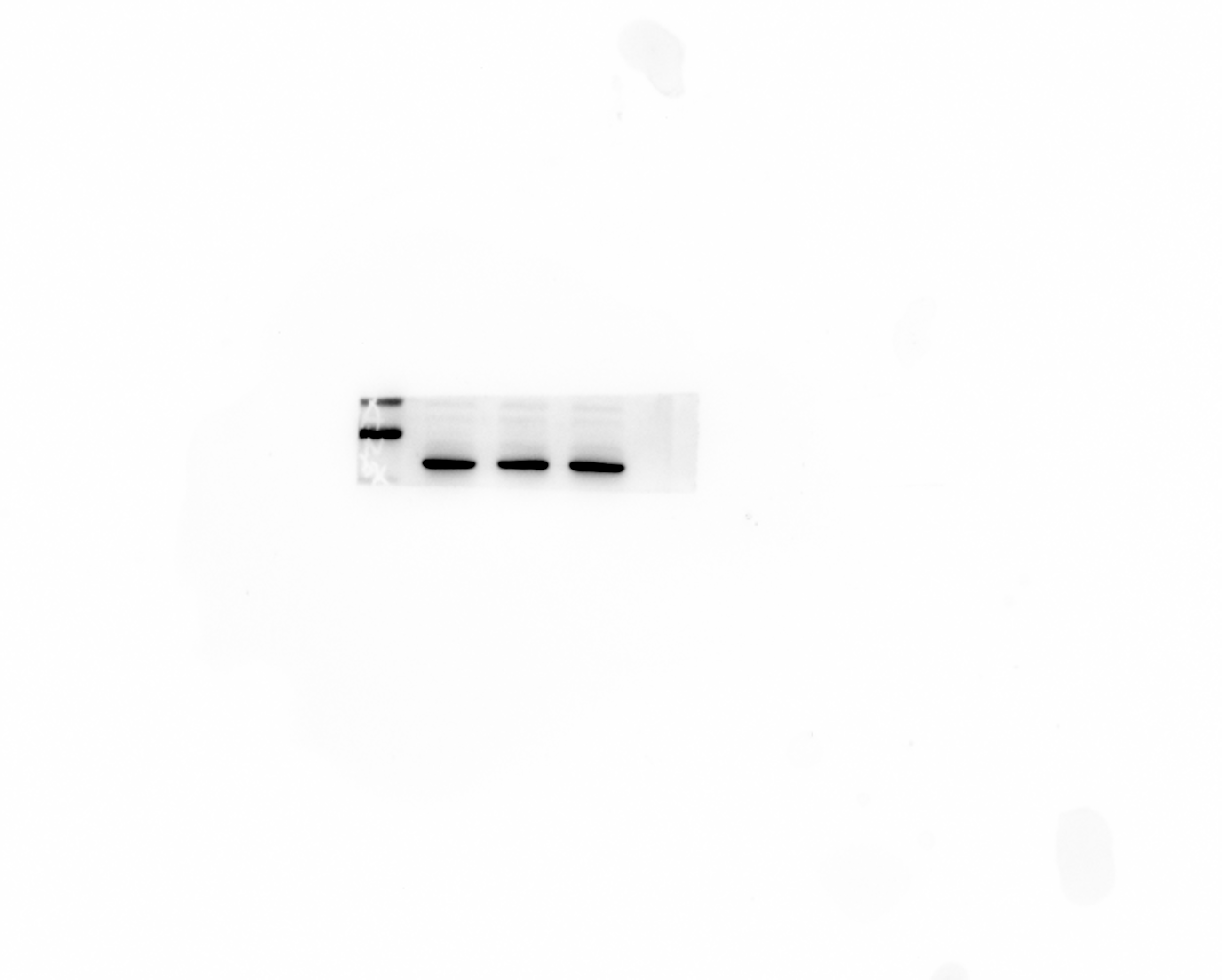

Supplement: Supplementary file 3 [file DataSheet1.ZIP › WB/AMO-1/7 p-cdk1/9.28/Agapdh.Tif]

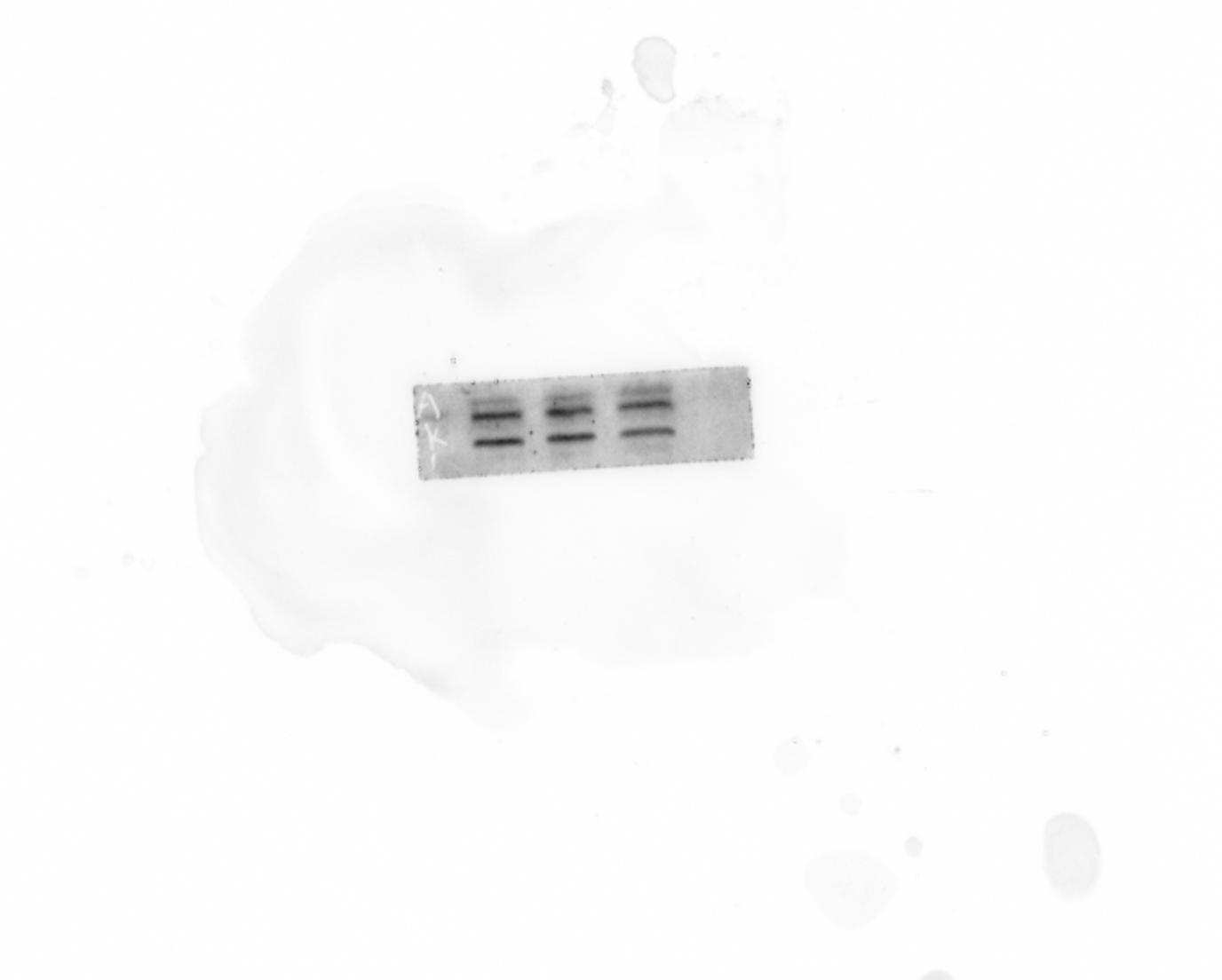

Supplement: Supplementary file 3 [file DataSheet1.ZIP › WB/AMO-1/7 p-cdk1/9.29/ACDK1 1.Tif]

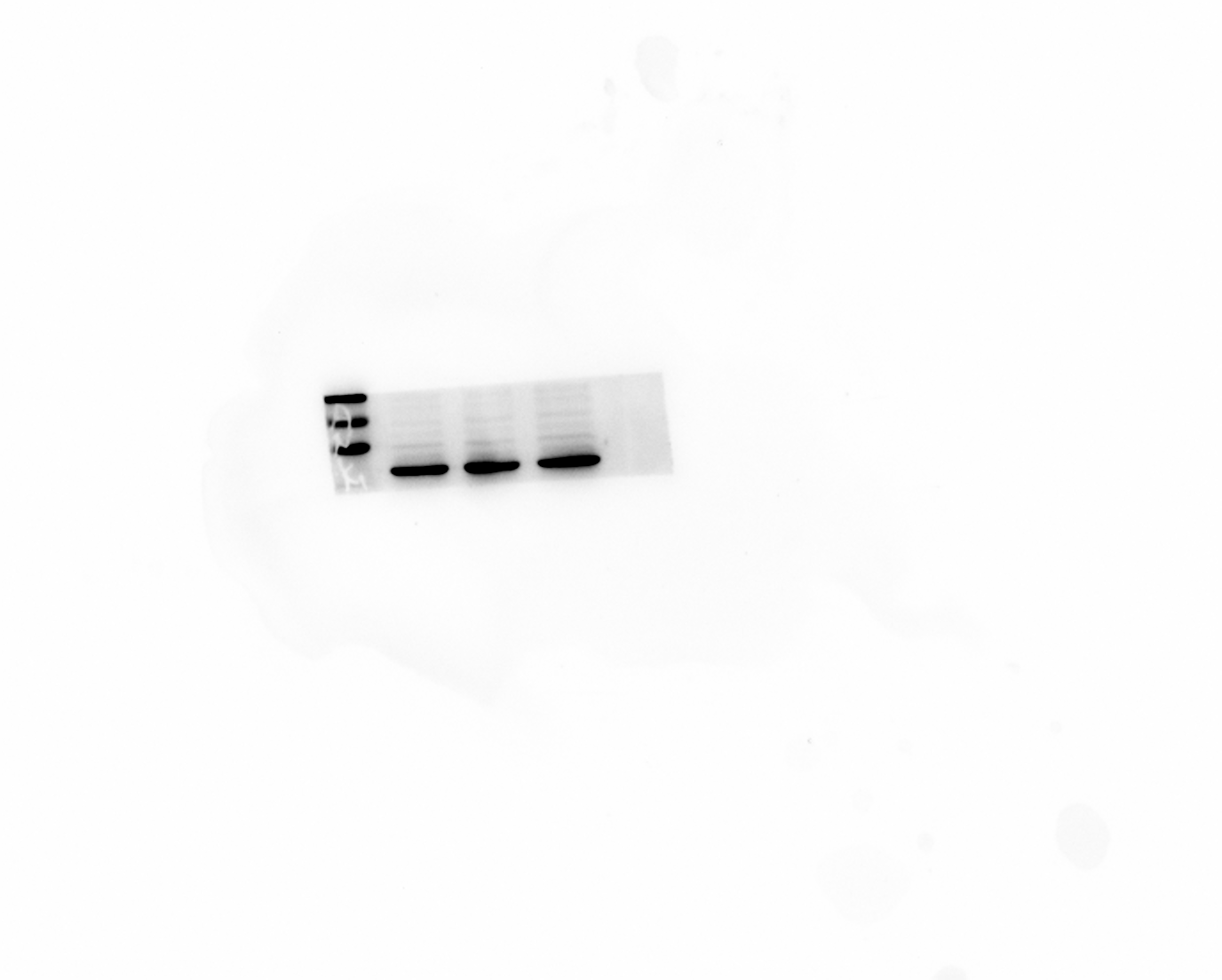

Supplement: Supplementary file 3 [file DataSheet1.ZIP › WB/AMO-1/7 p-cdk1/9.29/Agapdh.Tif]

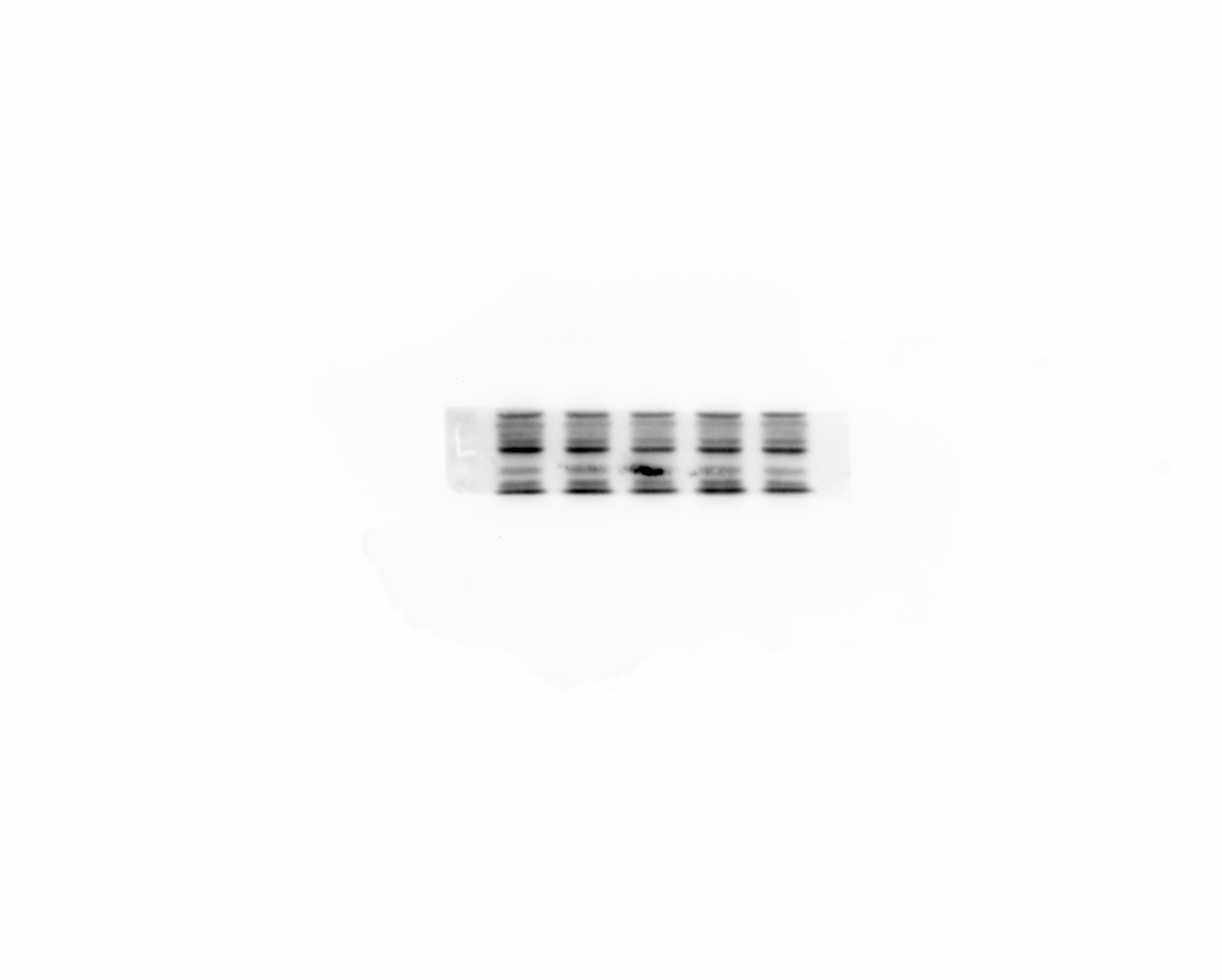

Supplement: Supplementary file 3 [file DataSheet1.ZIP › WB/AMO-1/8 LAMP5 sirna/9.11/LAMP5.Tif]

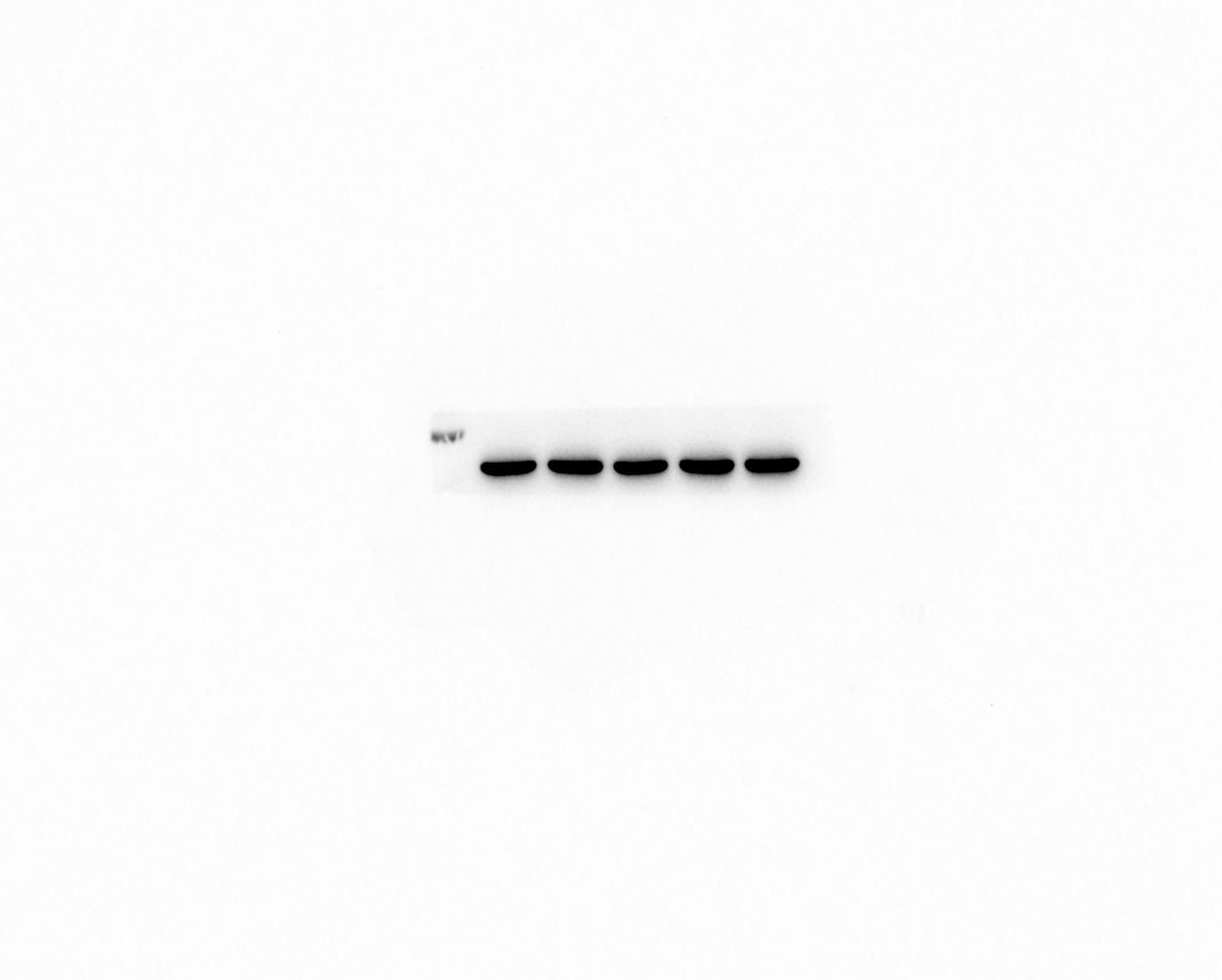

Supplement: Supplementary file 3 [file DataSheet1.ZIP › WB/AMO-1/8 LAMP5 sirna/9.11/a┬-actin.Tif]

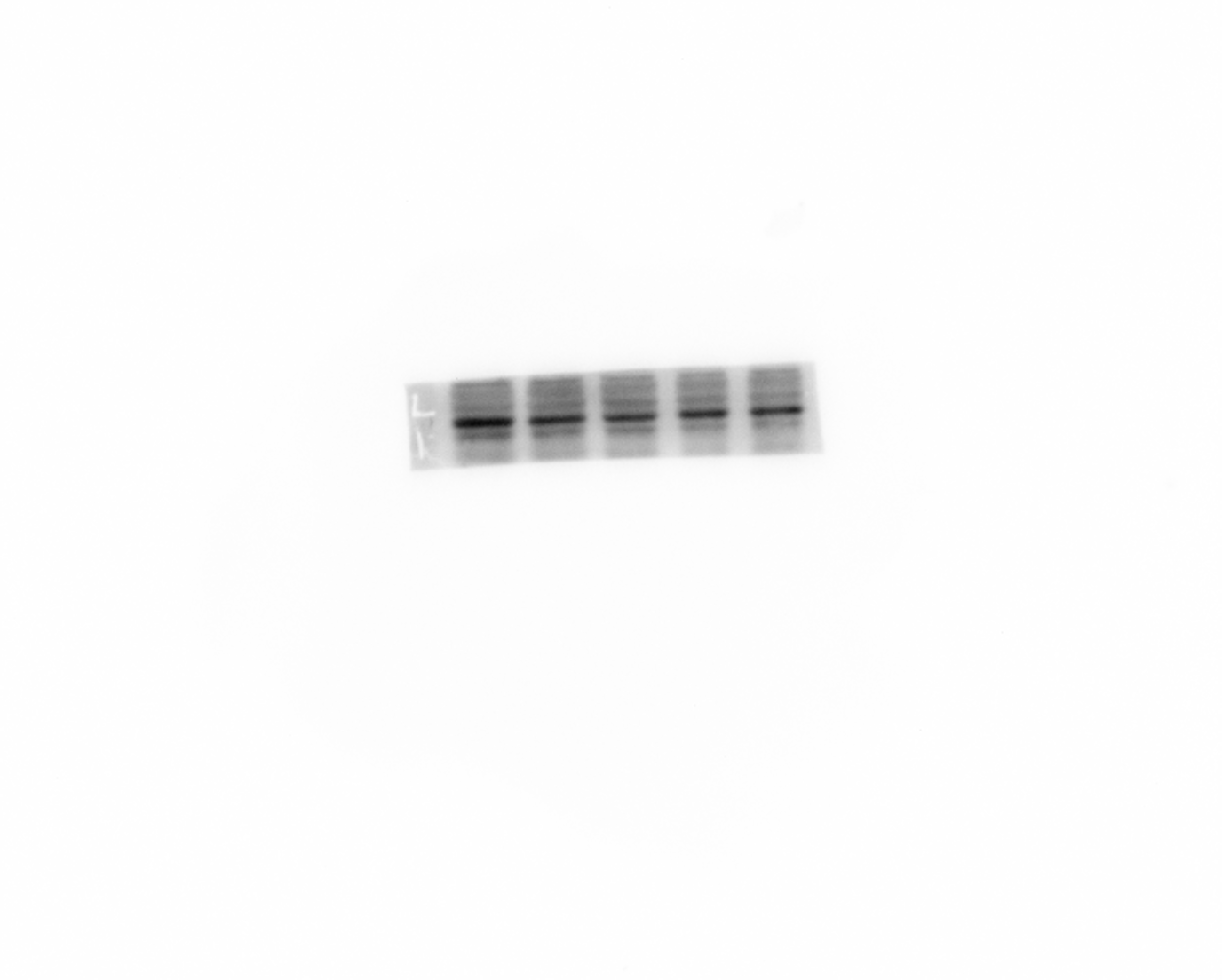

Supplement: Supplementary file 3 [file DataSheet1.ZIP › WB/AMO-1/8 LAMP5 sirna/9.21/L1.Tif]

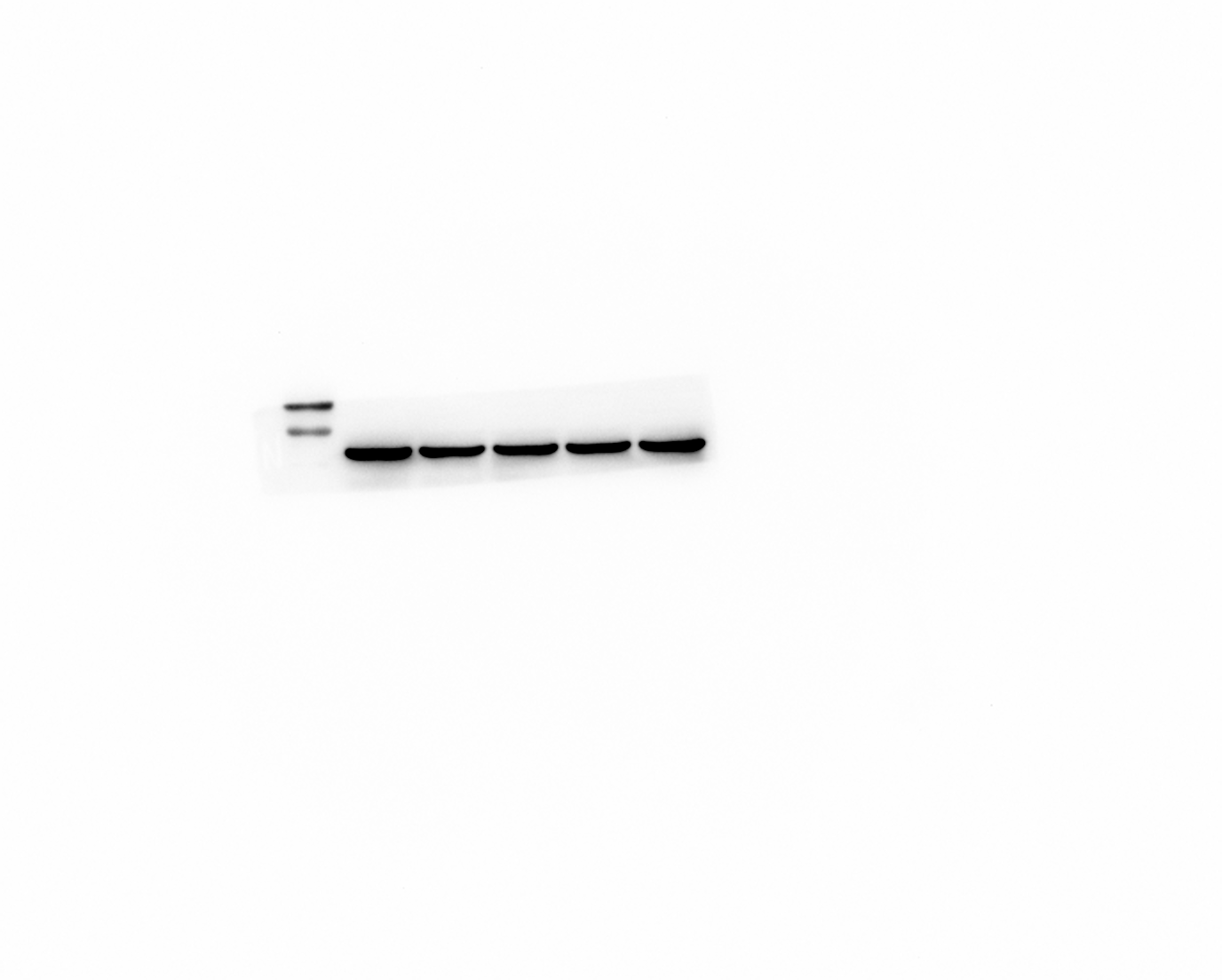

Supplement: Supplementary file 3 [file DataSheet1.ZIP › WB/AMO-1/8 LAMP5 sirna/9.21/a┬-actin.Tif]

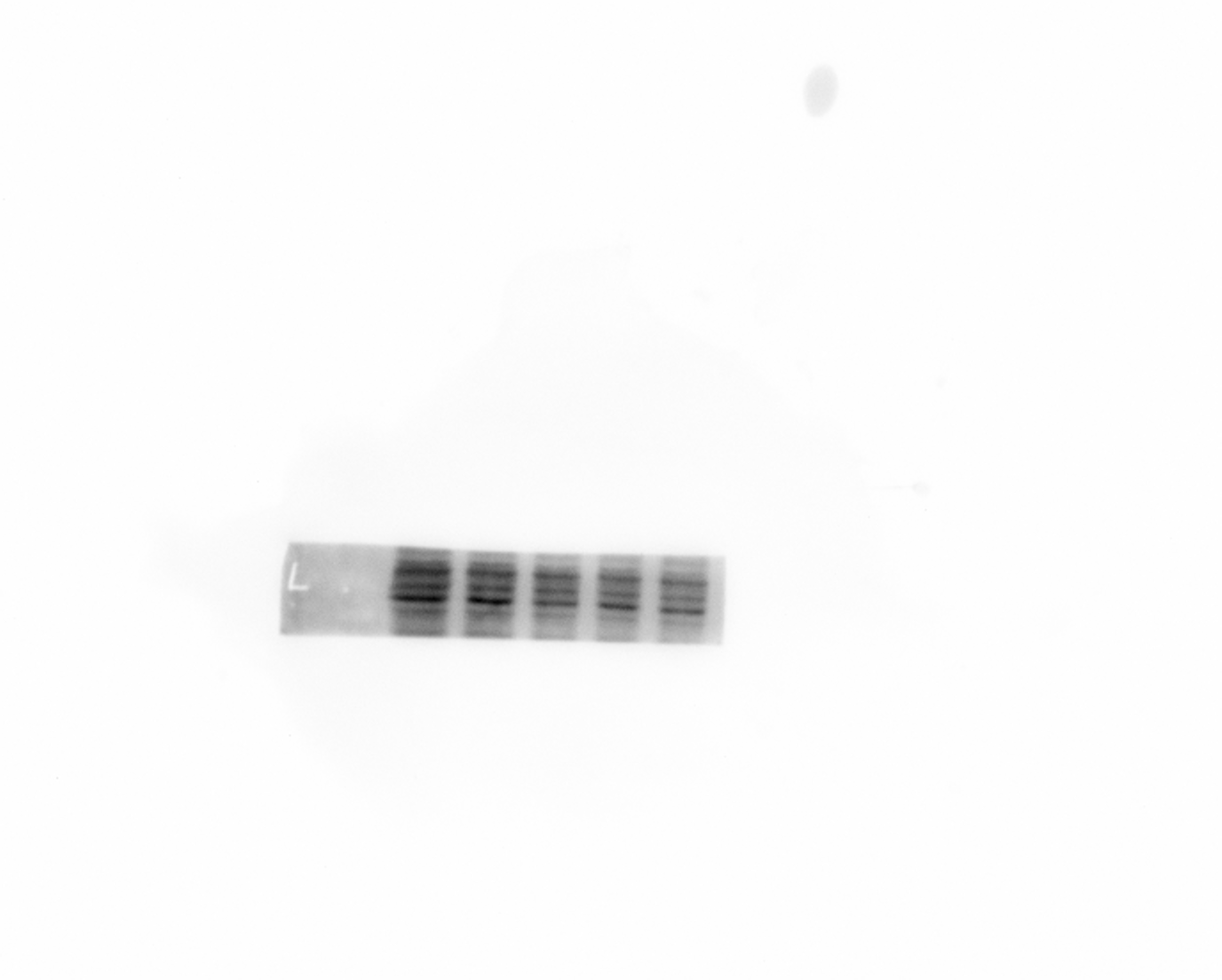

Supplement: Supplementary file 3 [file DataSheet1.ZIP › WB/AMO-1/8 LAMP5 sirna/9.22/L.Tif]

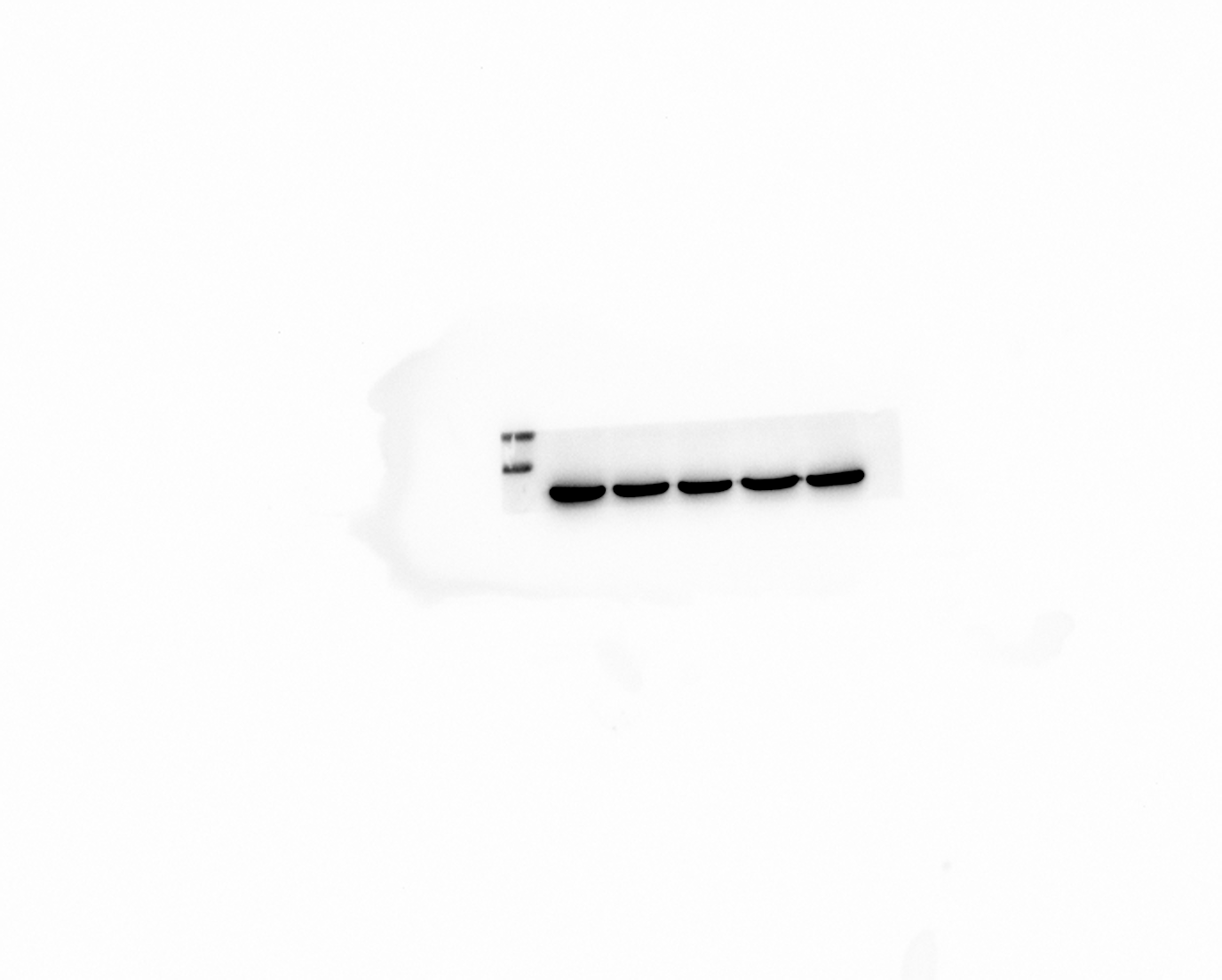

Supplement: Supplementary file 3 [file DataSheet1.ZIP › WB/AMO-1/8 LAMP5 sirna/9.22/a┬-actin.Tif]

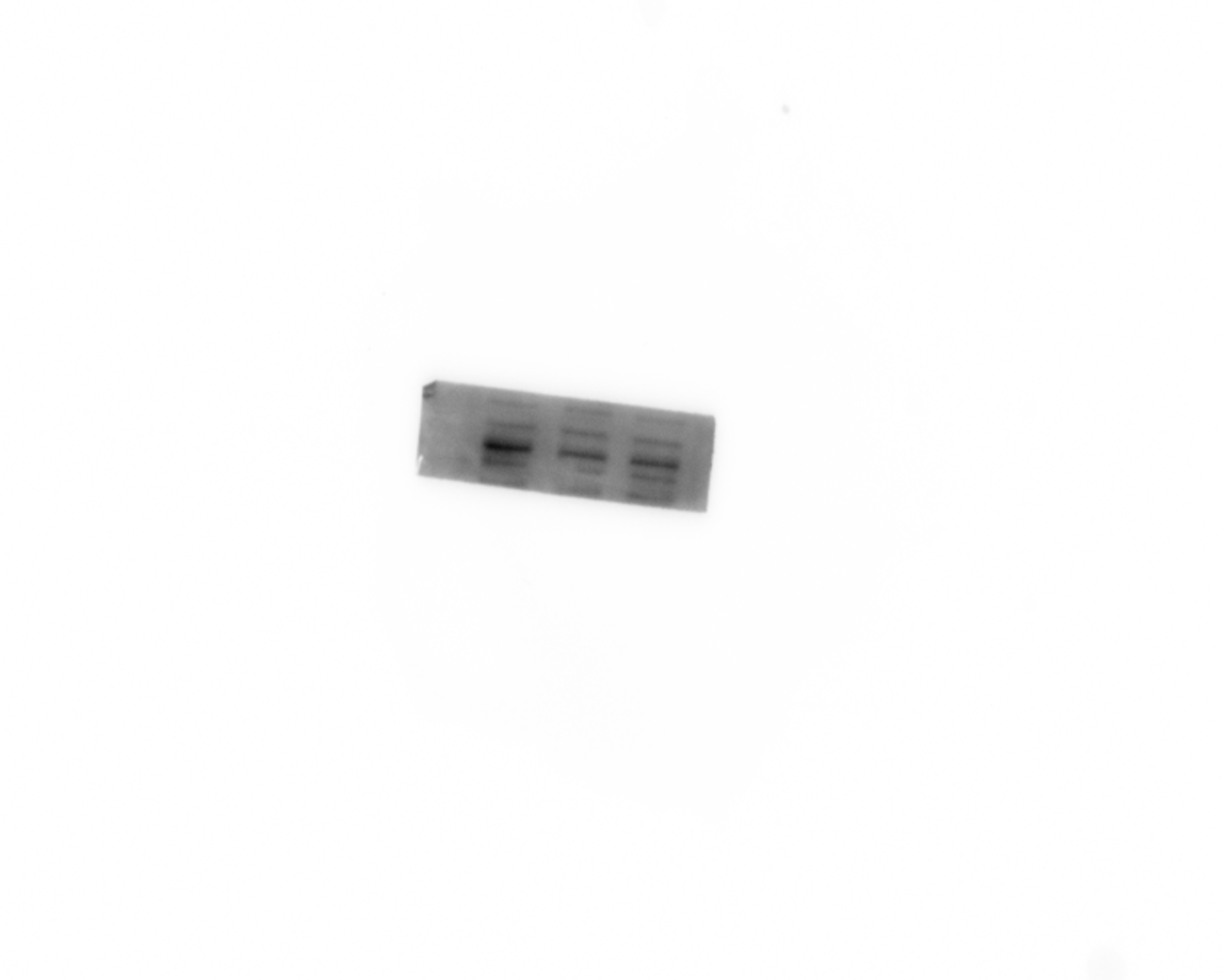

Supplement: Supplementary file 3 [file DataSheet1.ZIP › WB/AMO-1/9 p 38/10.3/P38 1.Tif]

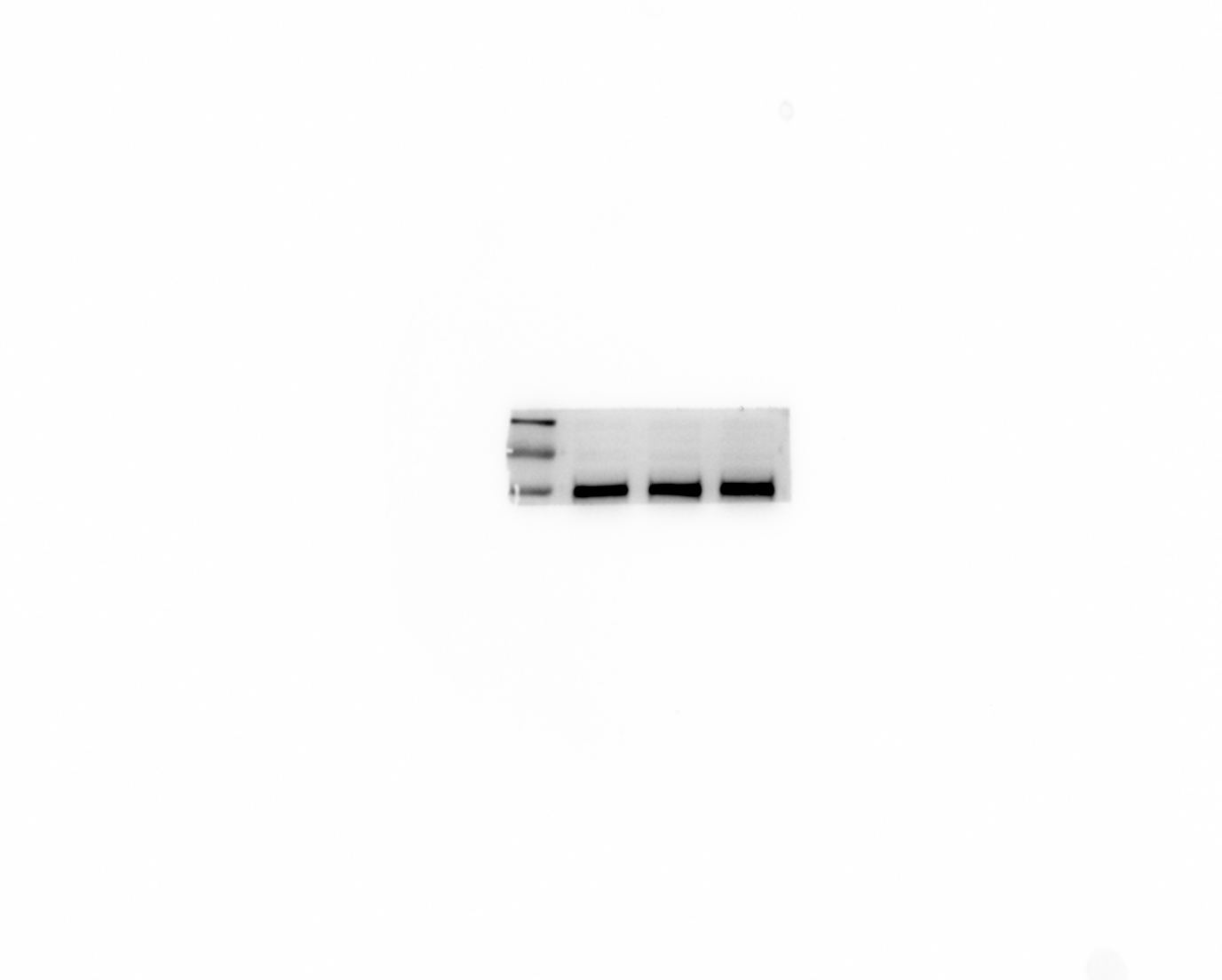

Supplement: Supplementary file 3 [file DataSheet1.ZIP › WB/AMO-1/9 p 38/10.3/a┬-tubulin.Tif]

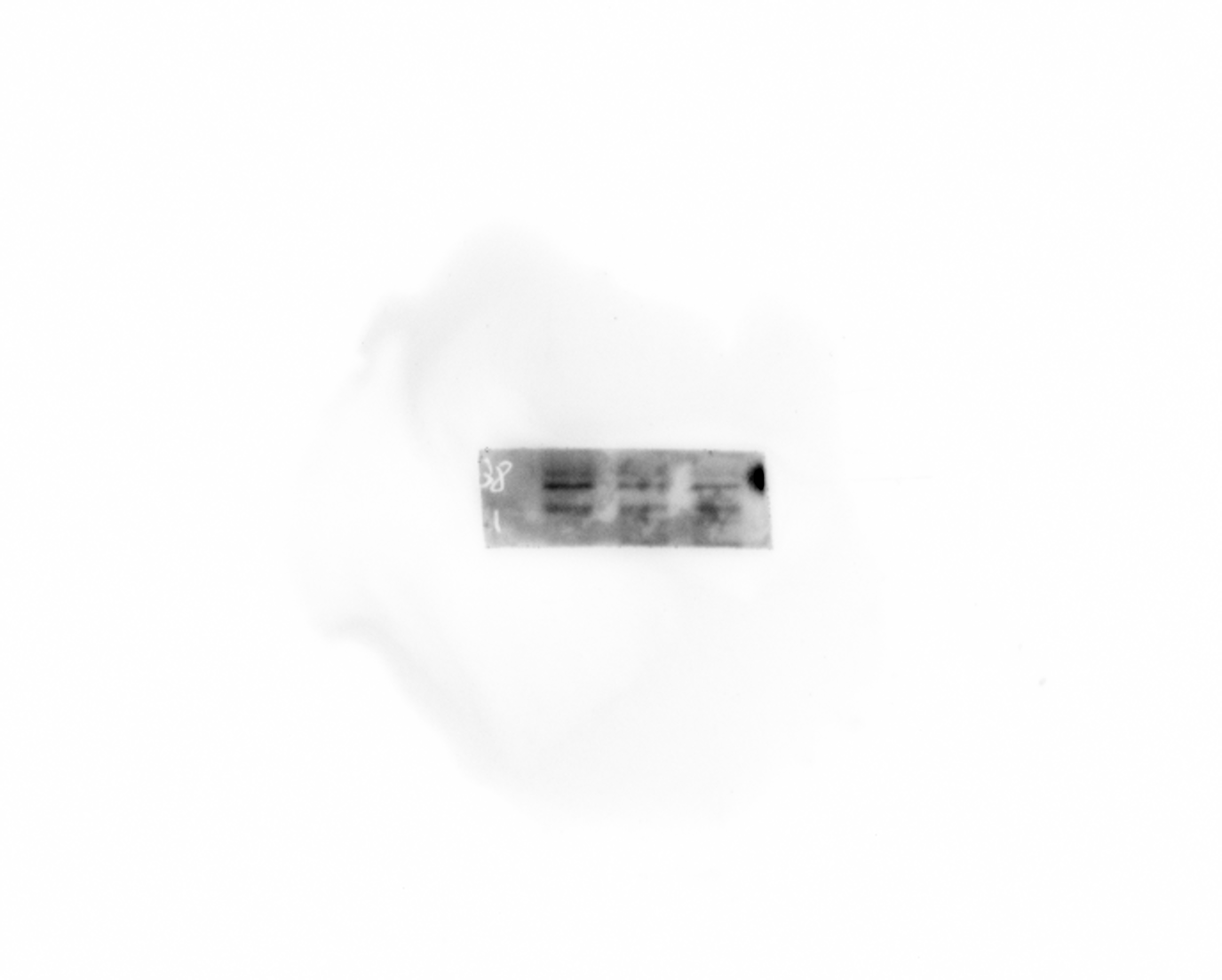

Supplement: Supplementary file 3 [file DataSheet1.ZIP › WB/AMO-1/9 p 38/10.4/P 38 1.Tif]

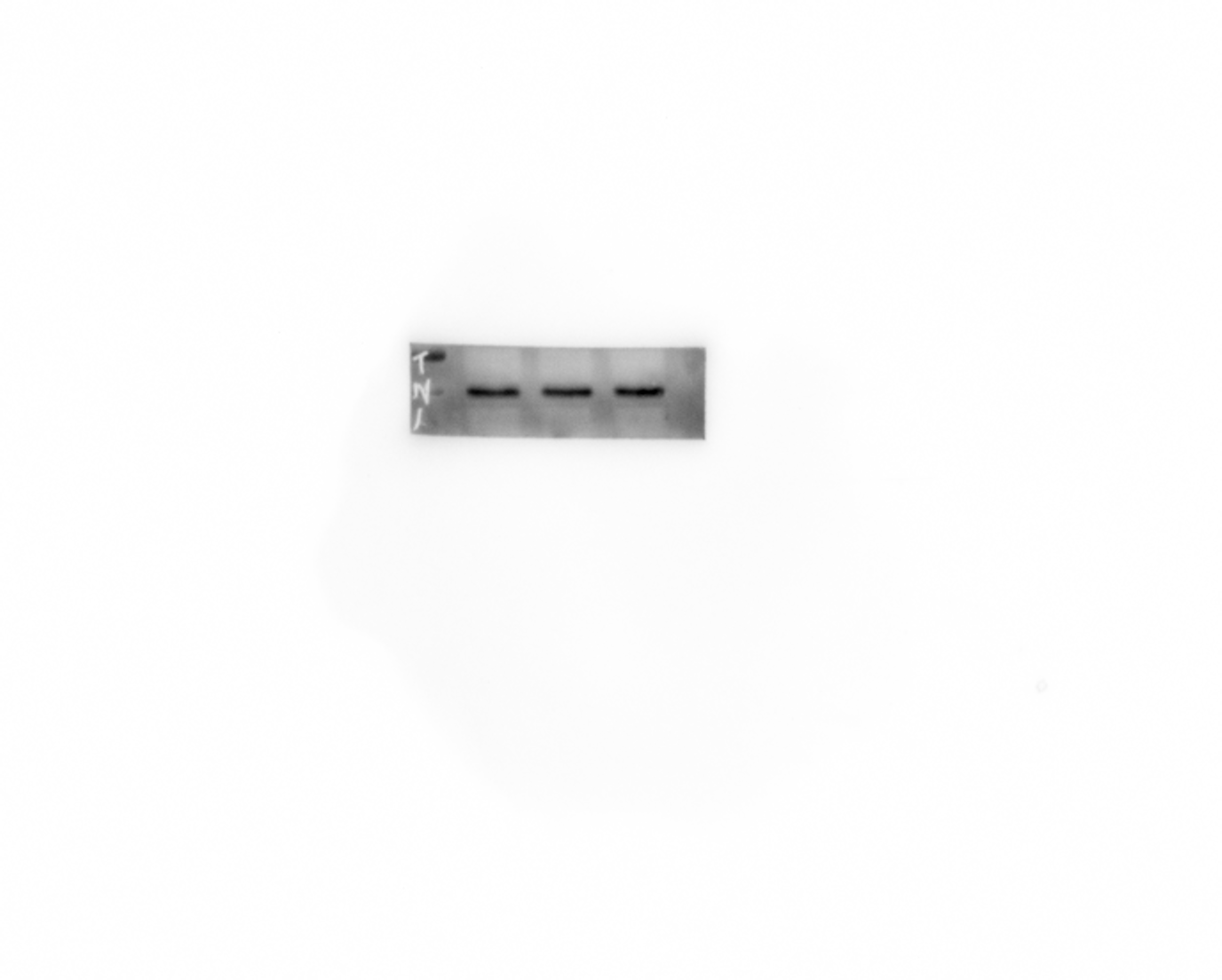

Supplement: Supplementary file 3 [file DataSheet1.ZIP › WB/AMO-1/9 p 38/10.4/a┬-tubulin.Tif]

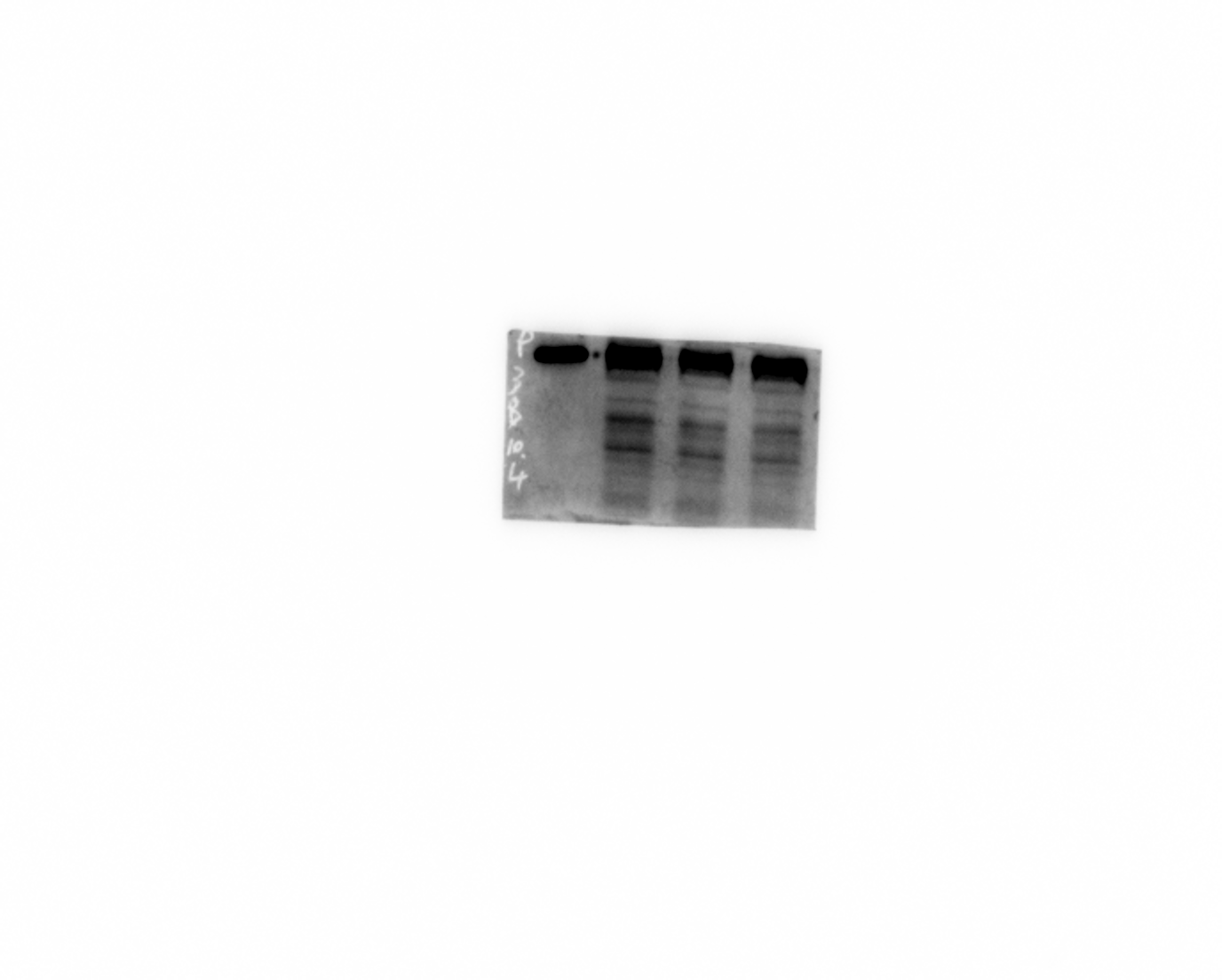

Supplement: Supplementary file 3 [file DataSheet1.ZIP › WB/AMO-1/9 p 38/10.6/A P38 1.Tif]

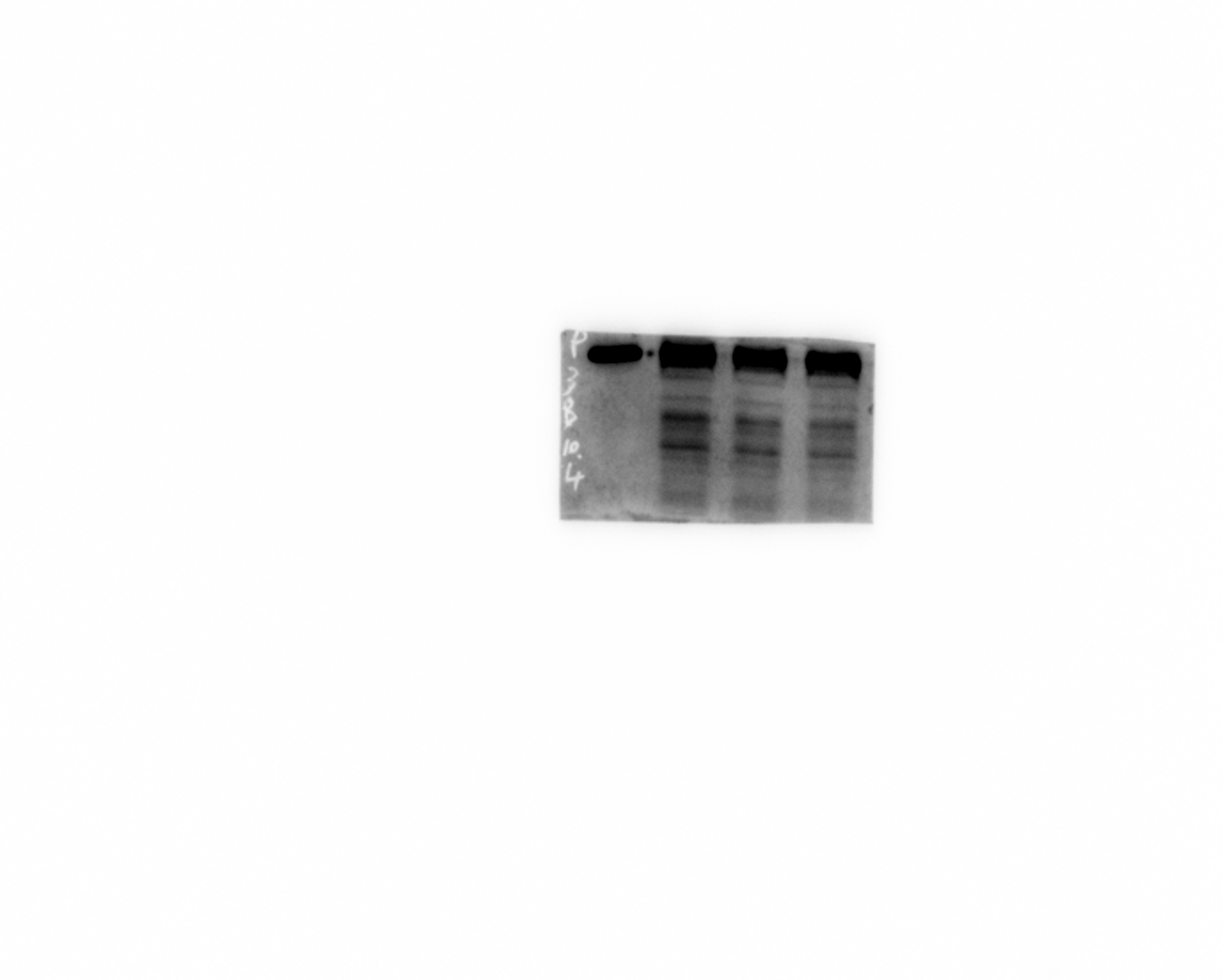

Supplement: Supplementary file 3 [file DataSheet1.ZIP › WB/AMO-1/9 p 38/10.6/P38 2.Tif]

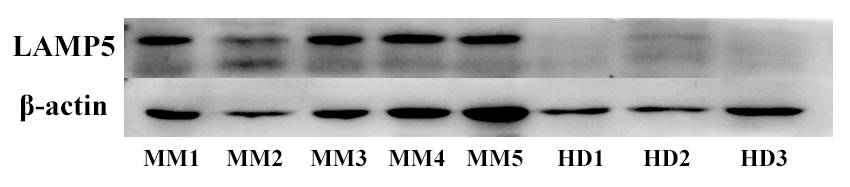

Supplement: Supplementary file 3 [file DataSheet1.ZIP › WB/Expression of LAMP5 in MM patients/LAMP5 +a┬-actin.tif]

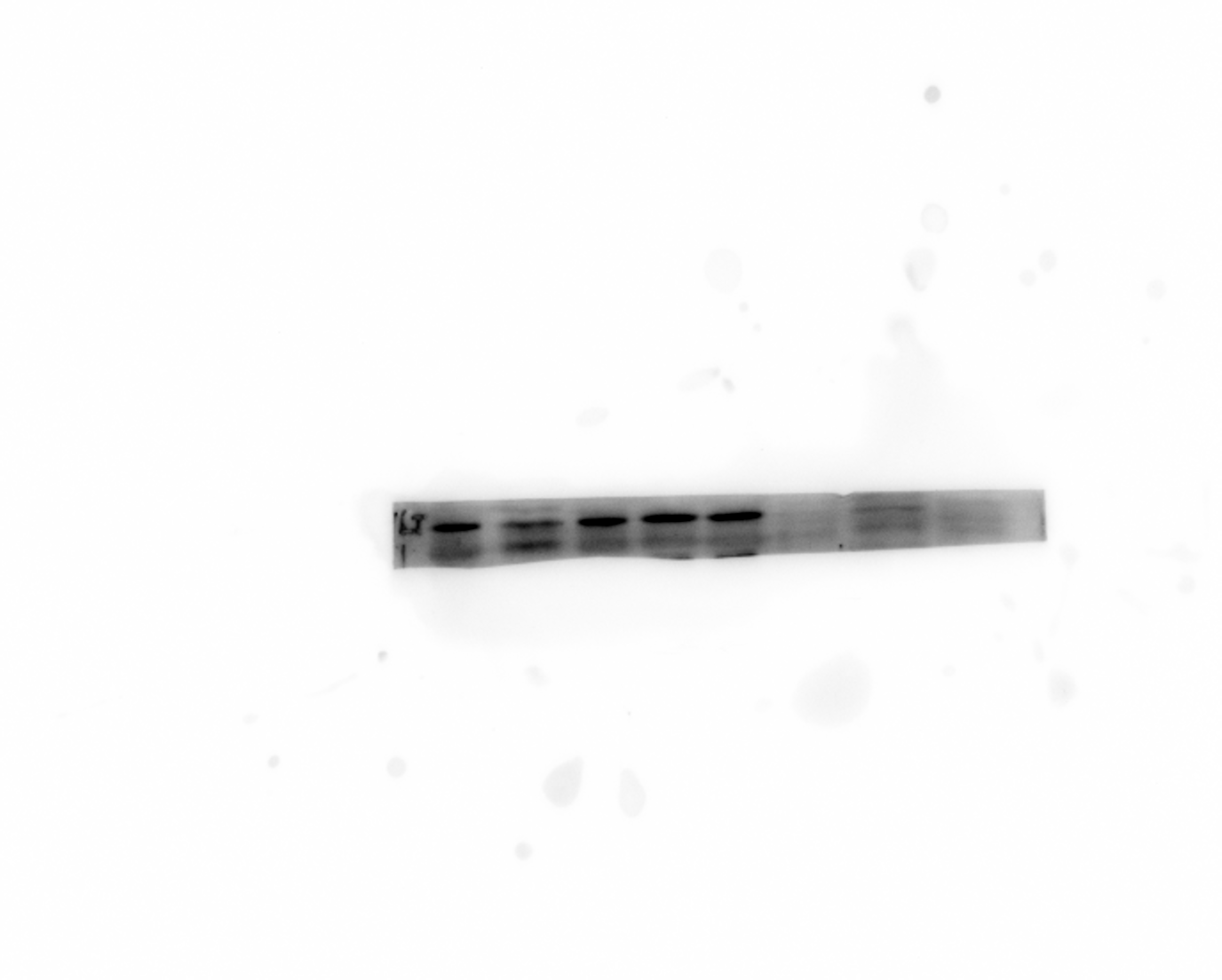

Supplement: Supplementary file 3 [file DataSheet1.ZIP › WB/Expression of LAMP5 in MM patients/LAMP5.Tif]

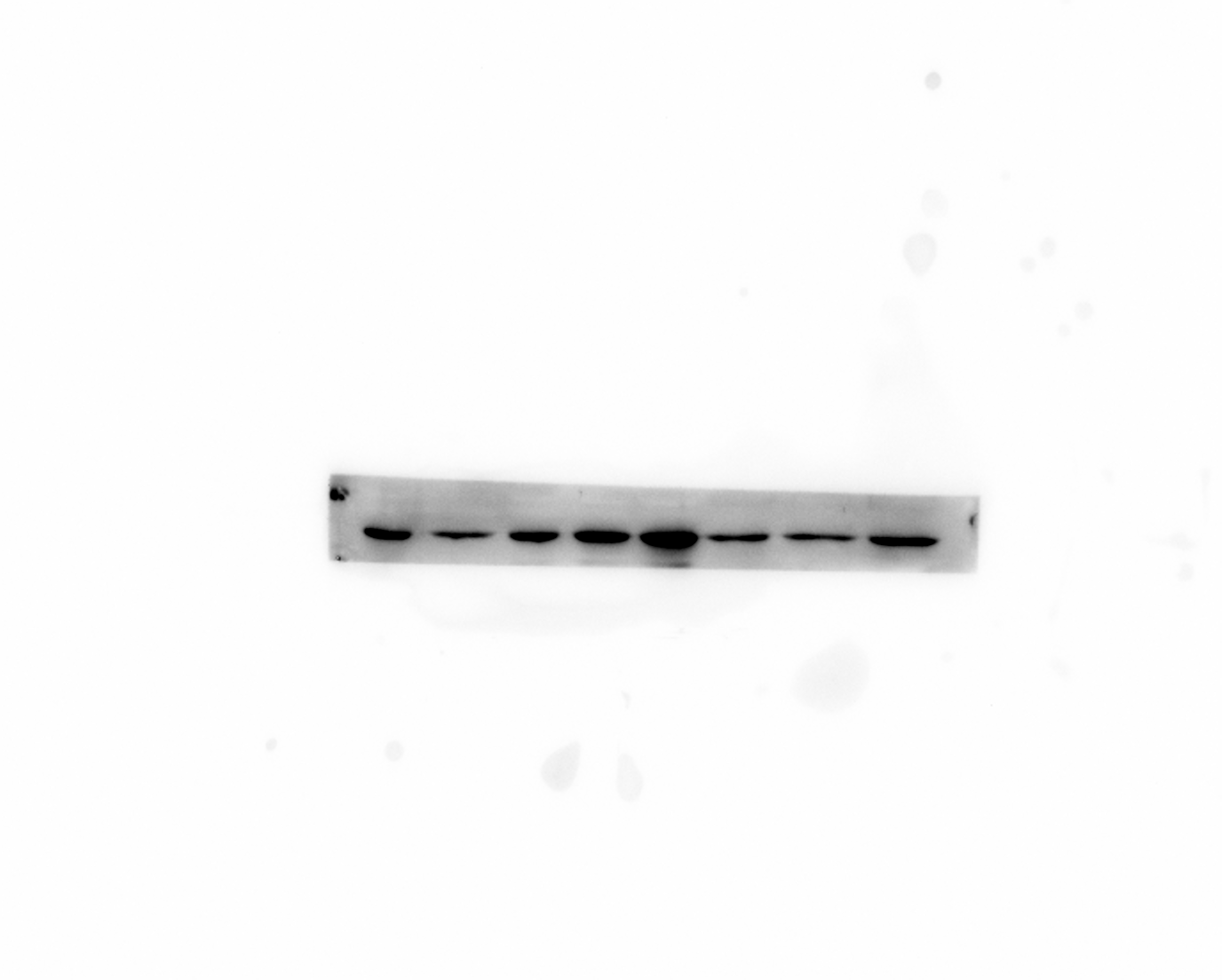

Supplement: Supplementary file 3 [file DataSheet1.ZIP › WB/Expression of LAMP5 in MM patients/a┬-actin.Tif]

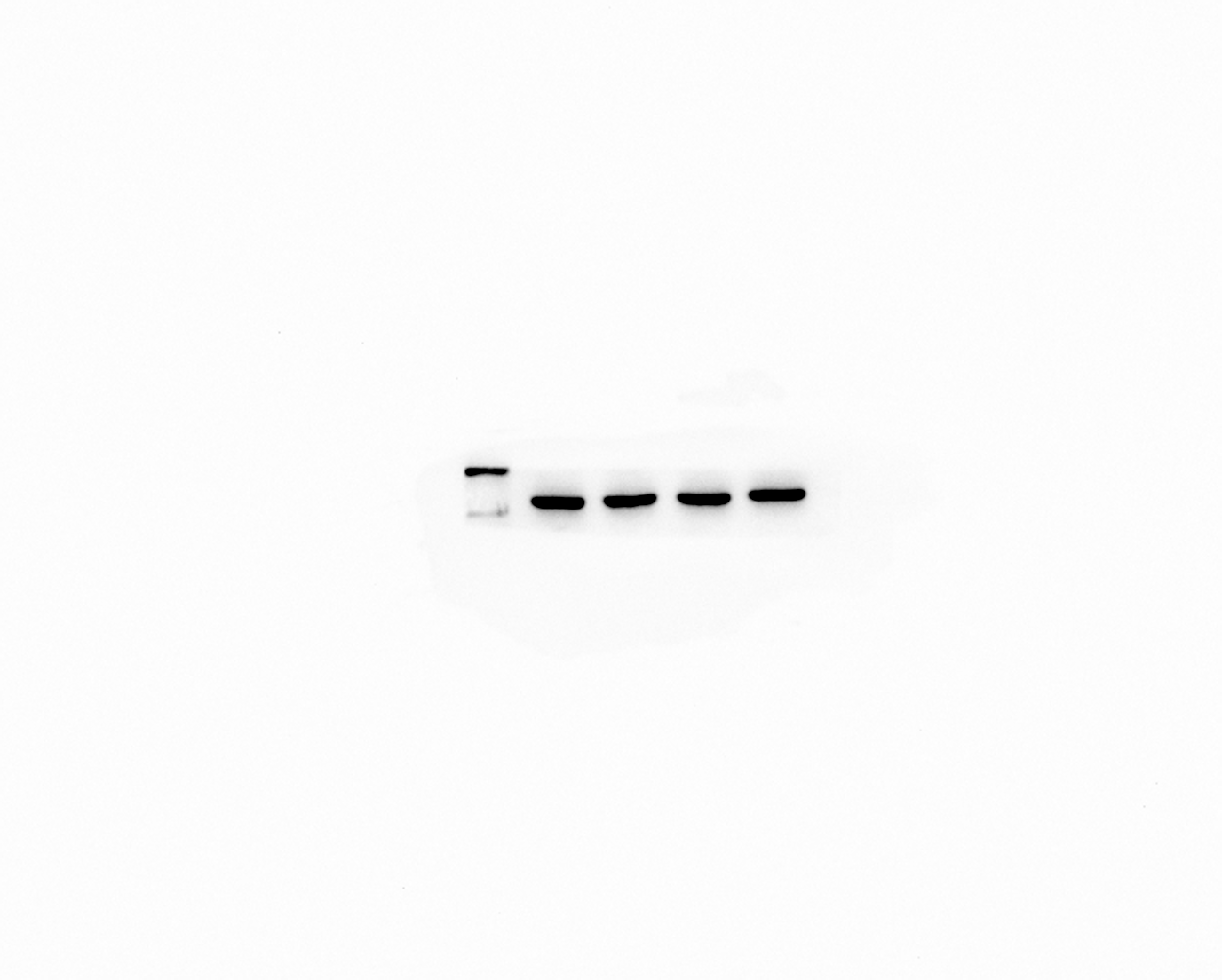

Supplement: Supplementary file 3 [file DataSheet1.ZIP › WB/Expression of LAMP5 in 4 MM cell lines/B ACTIN.Tif]

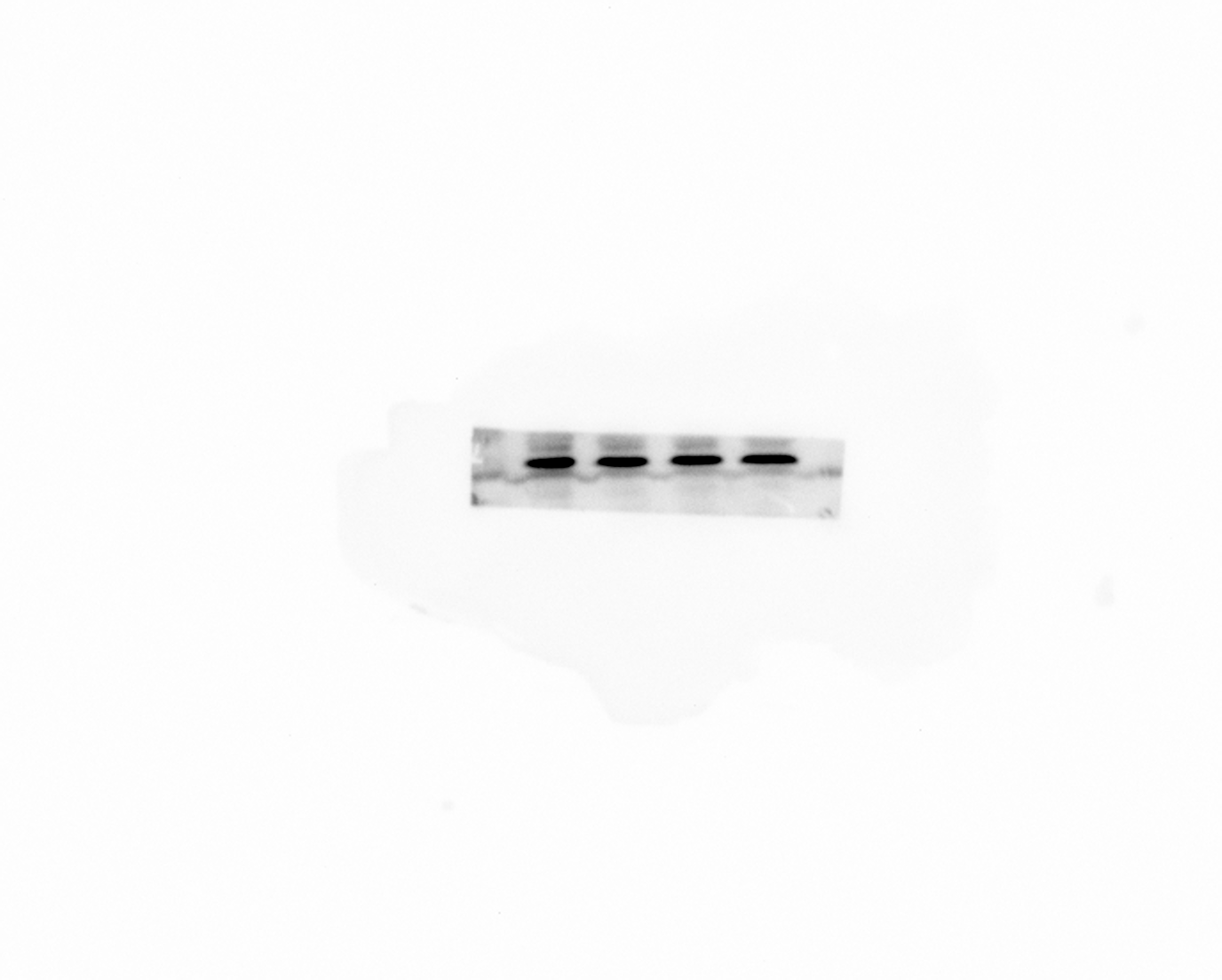

Supplement: Supplementary file 3 [file DataSheet1.ZIP › WB/Expression of LAMP5 in 4 MM cell lines/LAMP5 8226;AMO;929;U266.Tif]

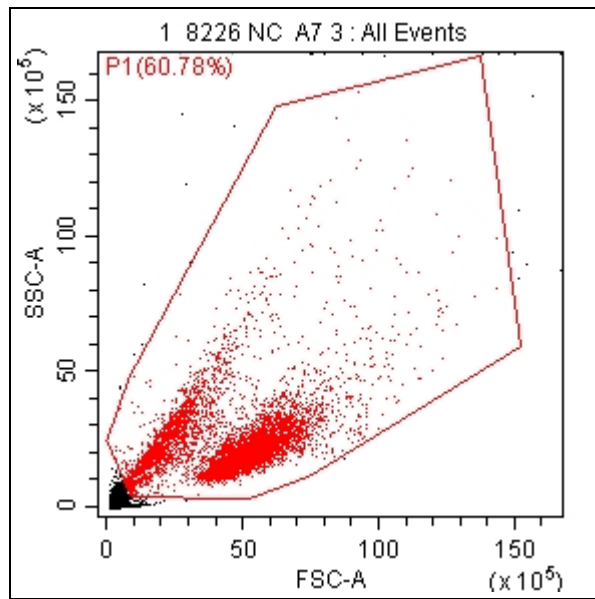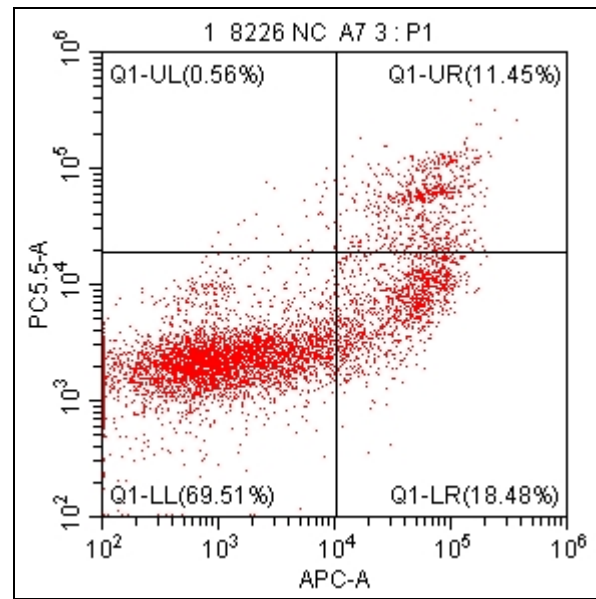

Supplement: Supplementary file 4 [file DataSheet2.ZIP › cell apoptosis/8226/1 8226 NC A7 3.pdf]

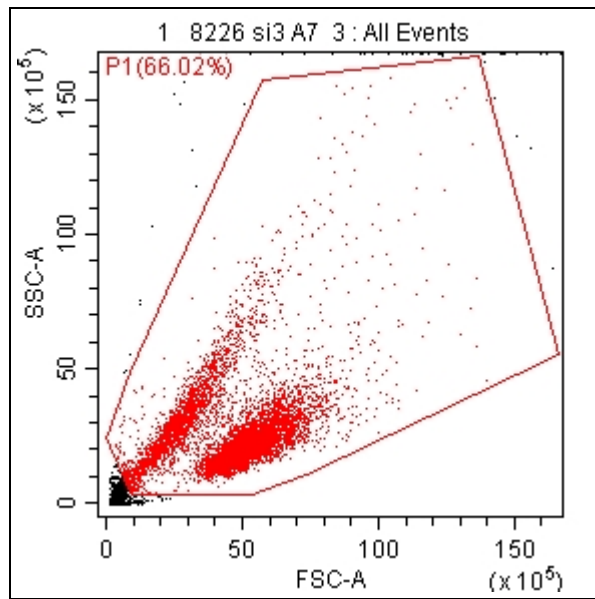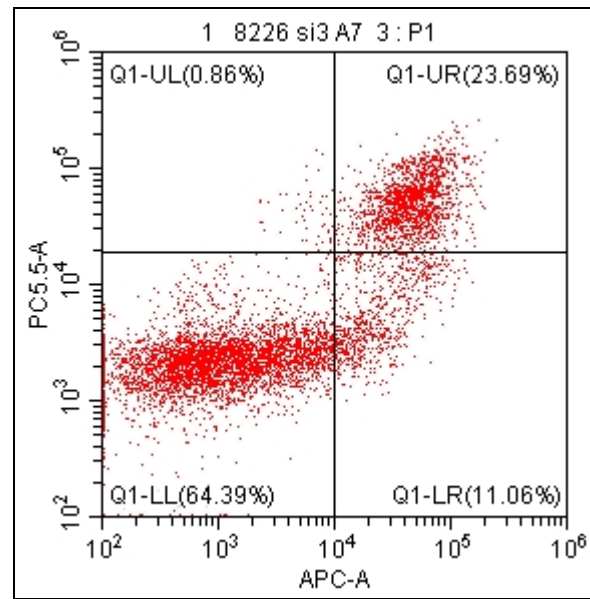

Supplement: Supplementary file 4 [file DataSheet2.ZIP › cell apoptosis/8226/1 8226 si3 A7 3.pdf]

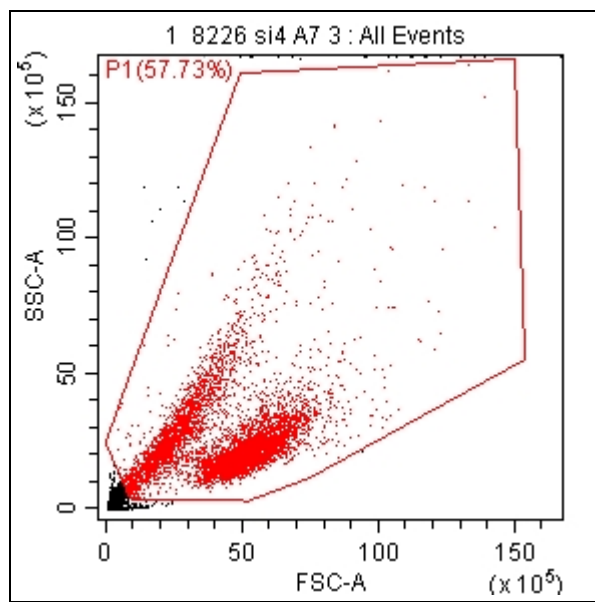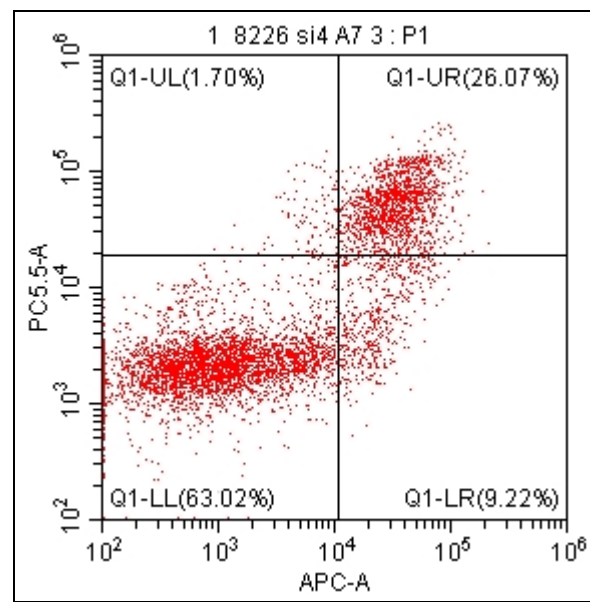

Supplement: Supplementary file 4 [file DataSheet2.ZIP › cell apoptosis/8226/1 8226 si4 A7 3.pdf]

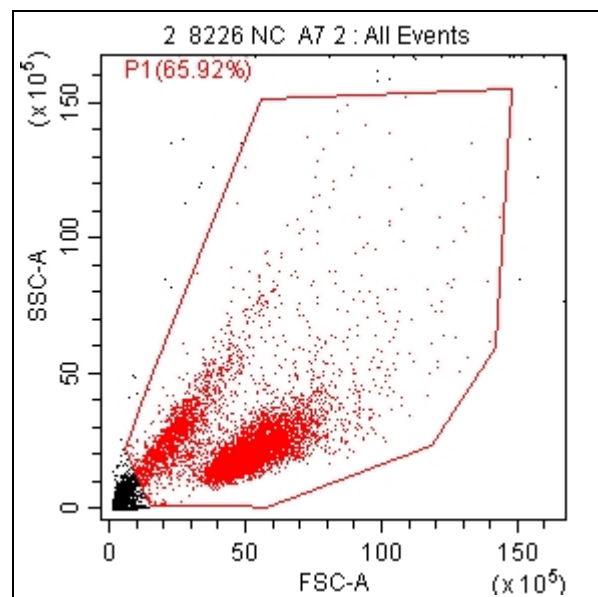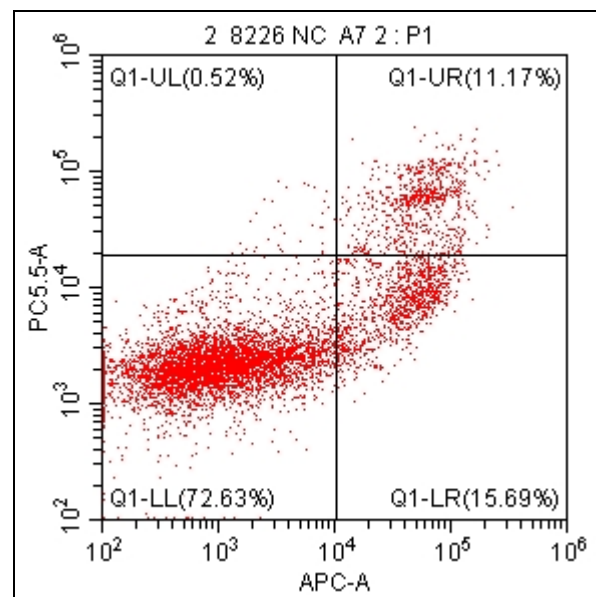

Supplement: Supplementary file 4 [file DataSheet2.ZIP › cell apoptosis/8226/2 8226 NC A7.pdf]

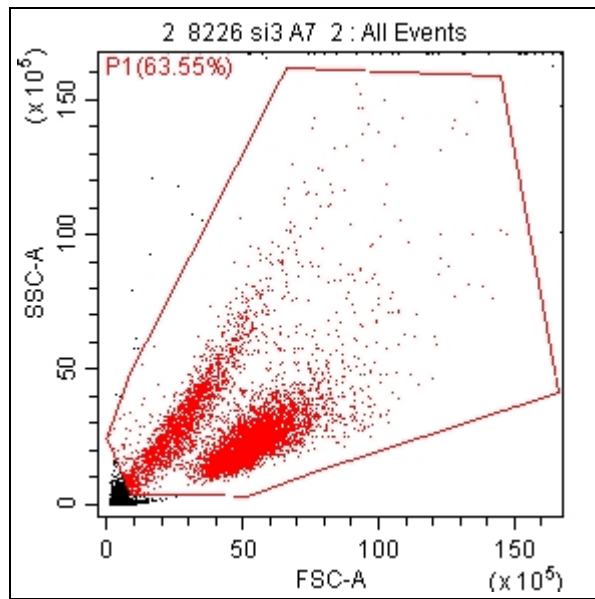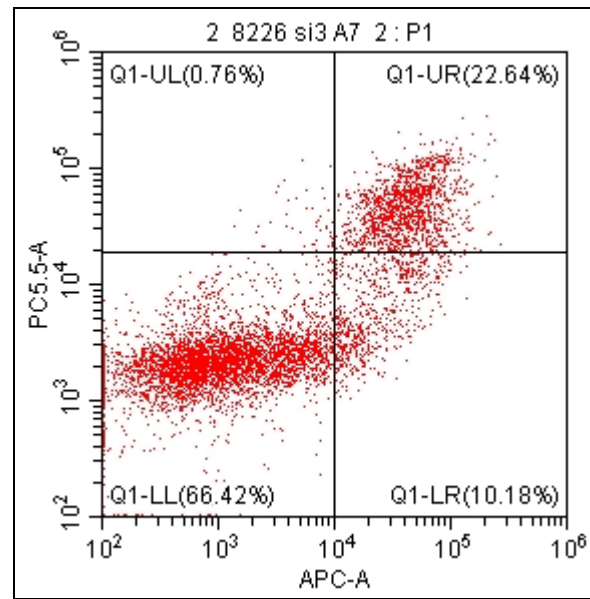

Supplement: Supplementary file 4 [file DataSheet2.ZIP › cell apoptosis/8226/2 8226 si3 A7.pdf]

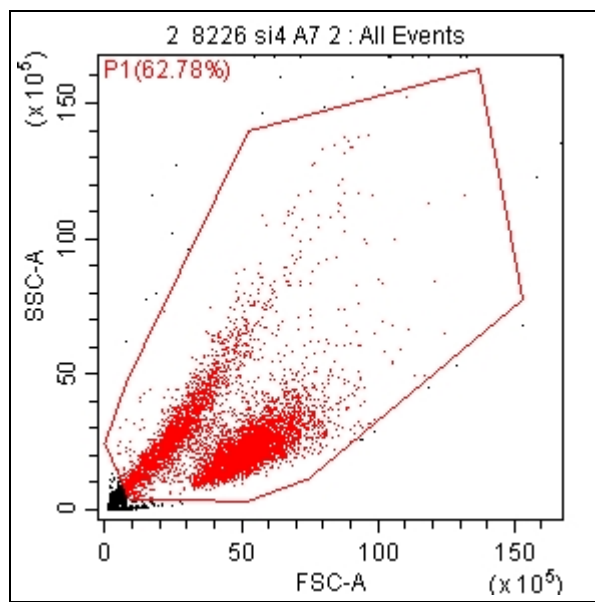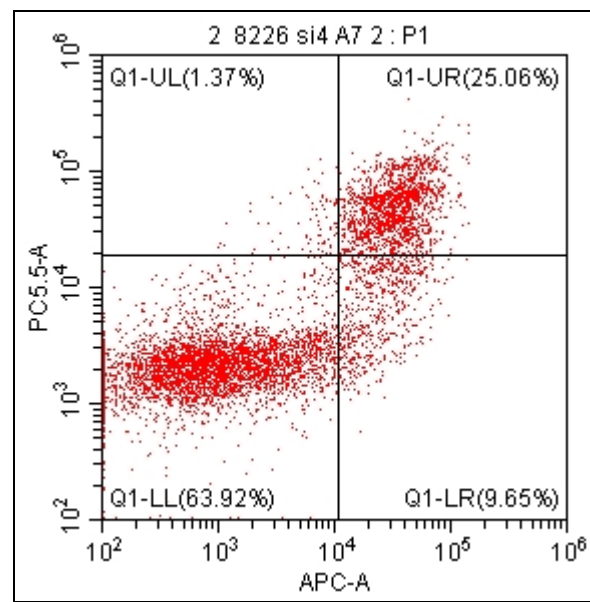

Supplement: Supplementary file 4 [file DataSheet2.ZIP › cell apoptosis/8226/2 8226 si4 A7.pdf]

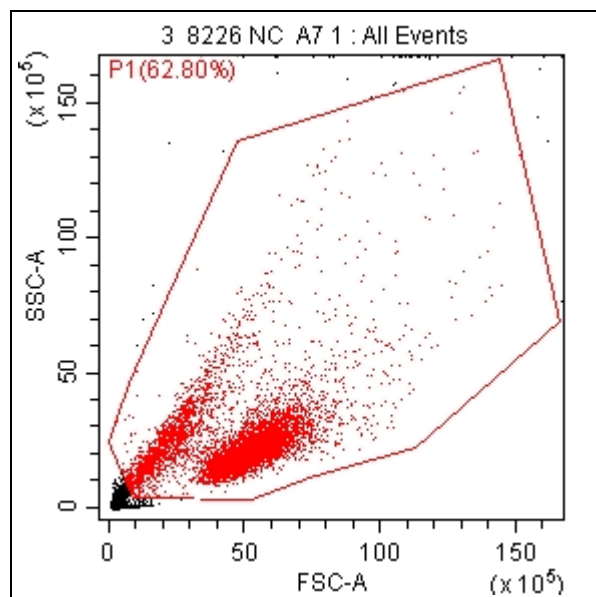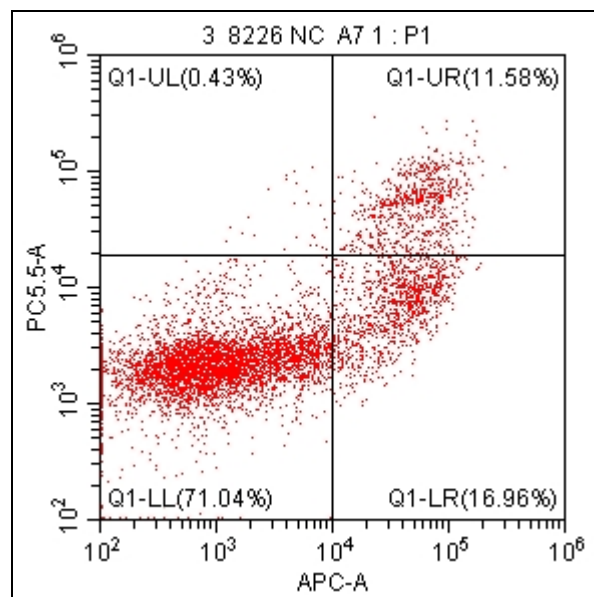

Supplement: Supplementary file 4 [file DataSheet2.ZIP › cell apoptosis/8226/3 8226 NC A7 1.pdf]

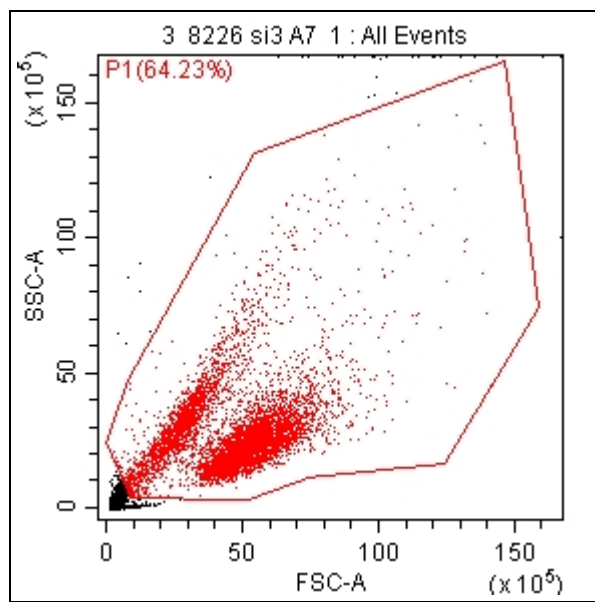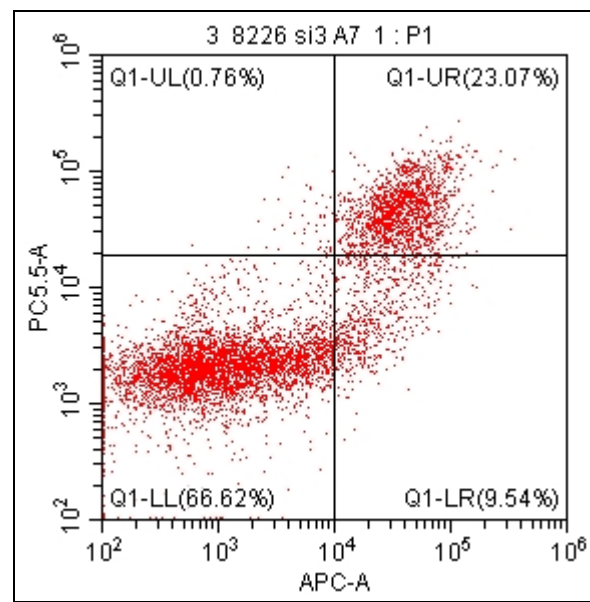

Supplement: Supplementary file 4 [file DataSheet2.ZIP › cell apoptosis/8226/3 8226 si3 A7 1.pdf]

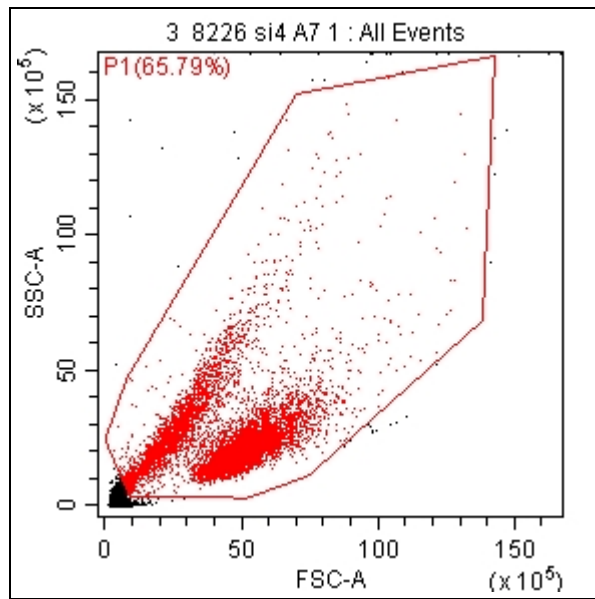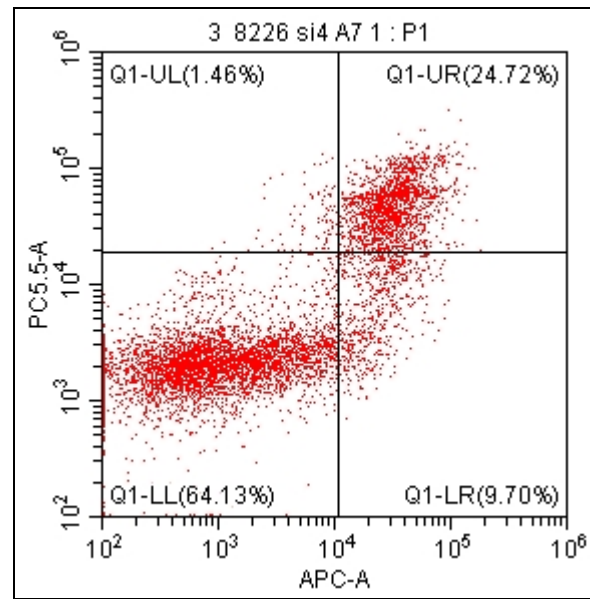

Supplement: Supplementary file 4 [file DataSheet2.ZIP › cell apoptosis/8226/3 8226 si4 A7 1.pdf]

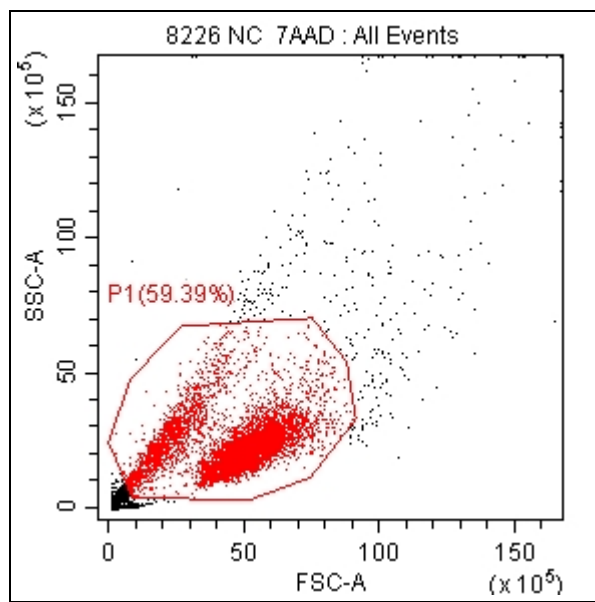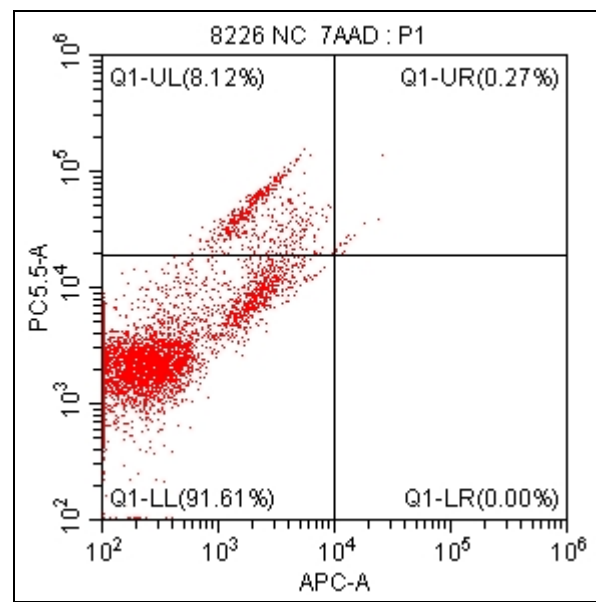

Supplement: Supplementary file 4 [file DataSheet2.ZIP › cell apoptosis/8226/8226 NC 7AAD.pdf]

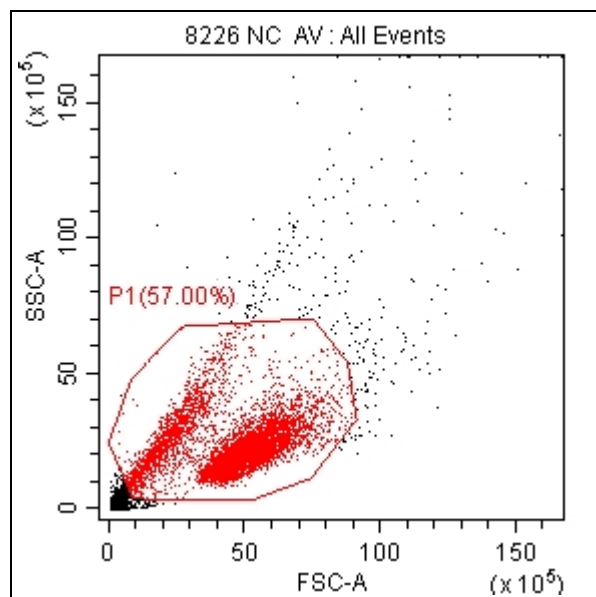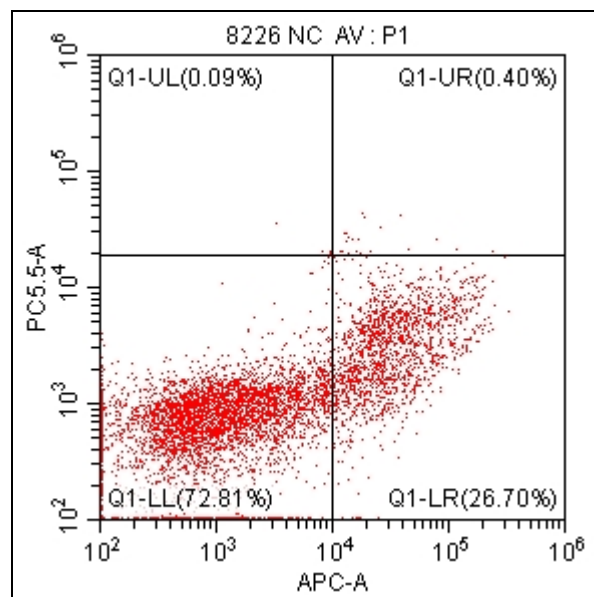

Supplement: Supplementary file 4 [file DataSheet2.ZIP › cell apoptosis/8226/8226 NC AV.pdf]

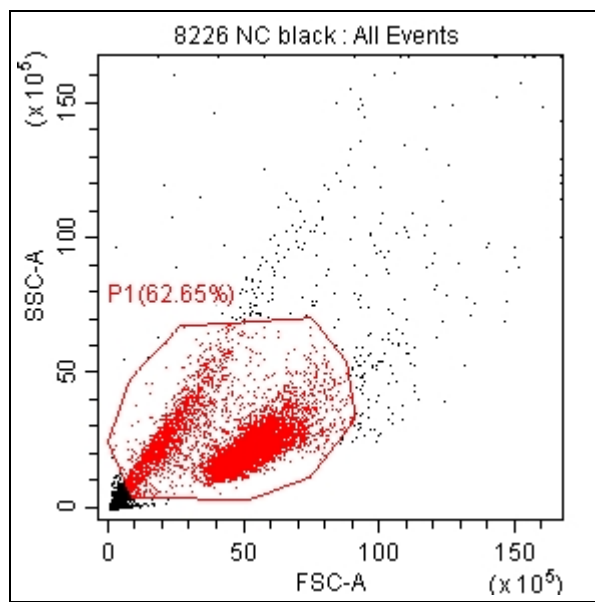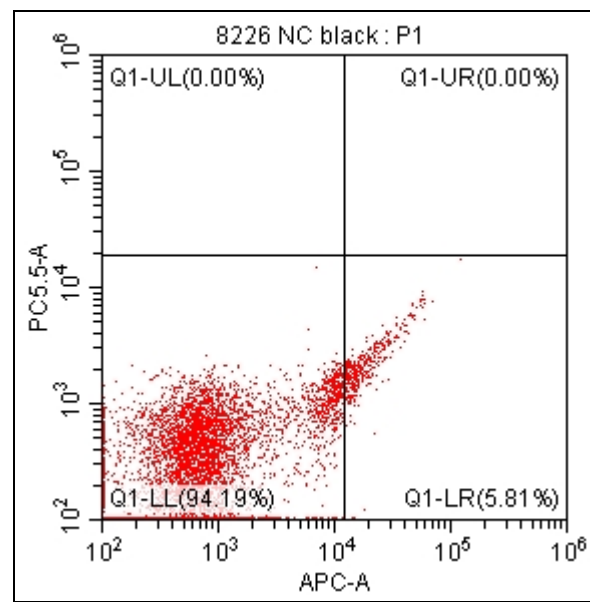

Supplement: Supplementary file 4 [file DataSheet2.ZIP › cell apoptosis/8226/8226 NC blank.pdf]

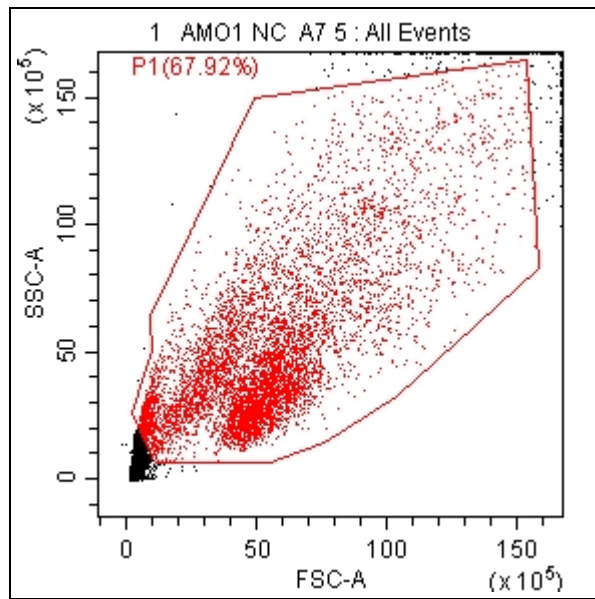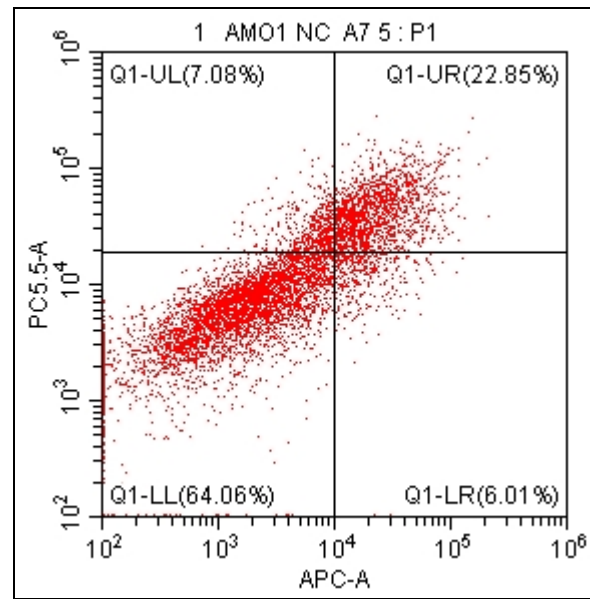

Supplement: Supplementary file 4 [file DataSheet2.ZIP › cell apoptosis/AMO1/1 AMO1 NC A7.pdf]

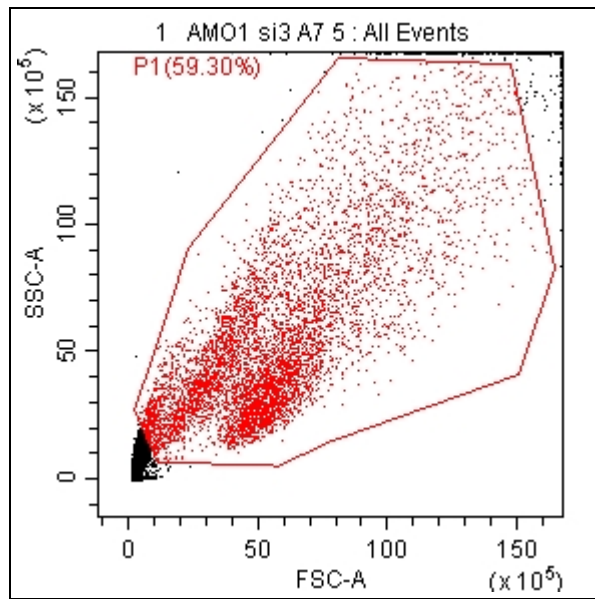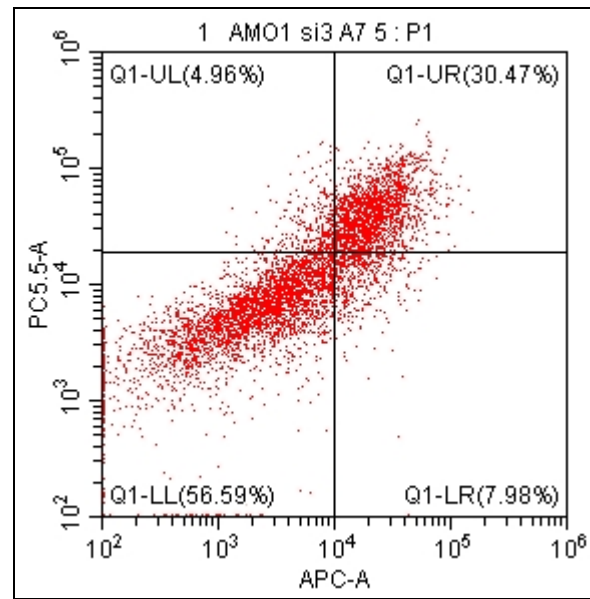

Supplement: Supplementary file 4 [file DataSheet2.ZIP › cell apoptosis/AMO1/1 AMO1 si3 A7.pdf]

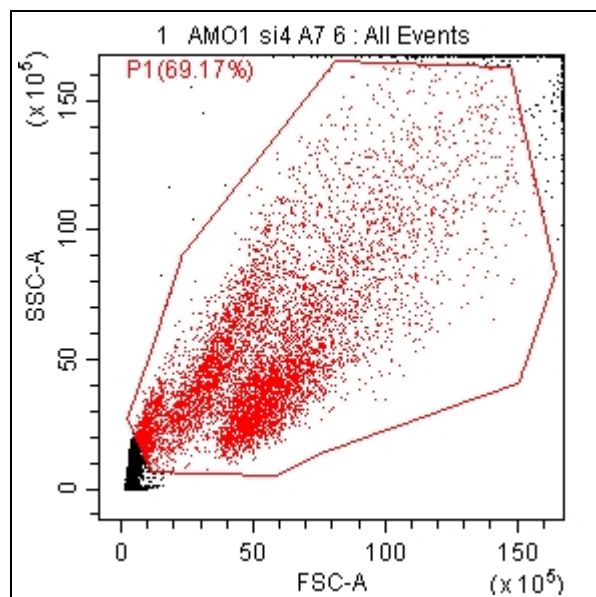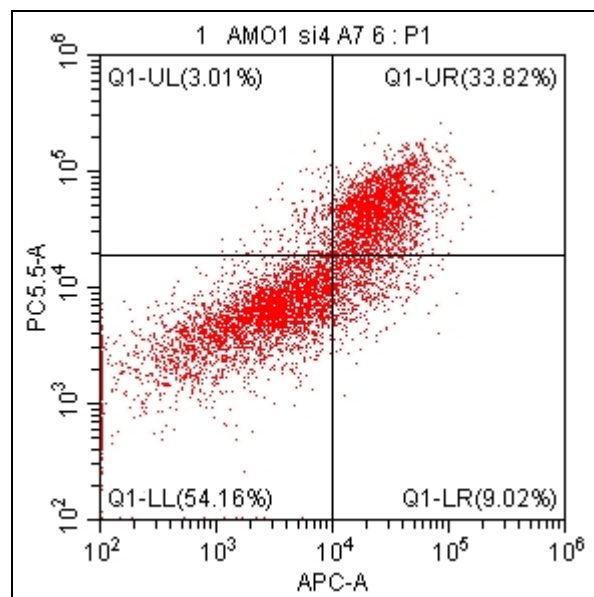

Supplement: Supplementary file 4 [file DataSheet2.ZIP › cell apoptosis/AMO1/1 AMO1 si4 A7 6.pdf]

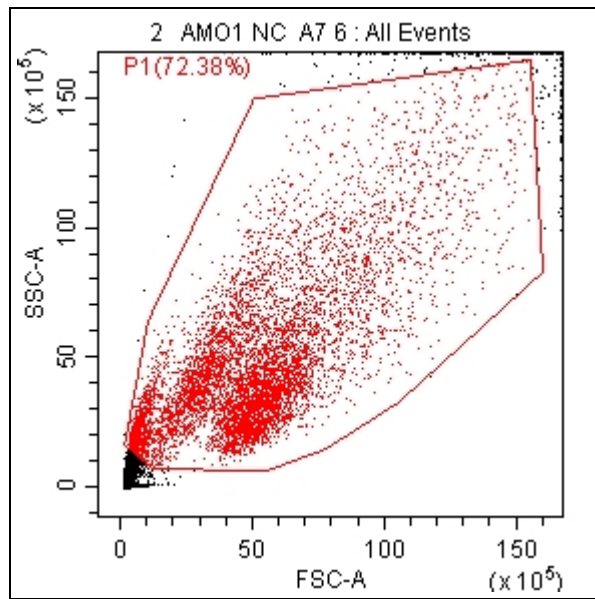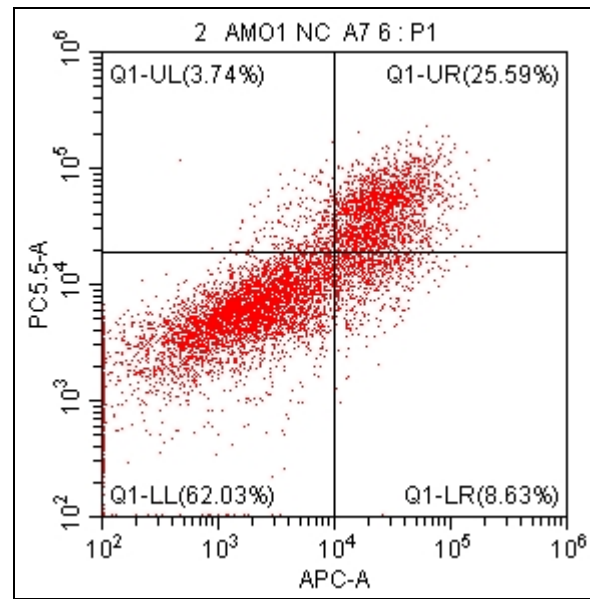

Supplement: Supplementary file 4 [file DataSheet2.ZIP › cell apoptosis/AMO1/2 AMO1 NC A7 6.pdf]

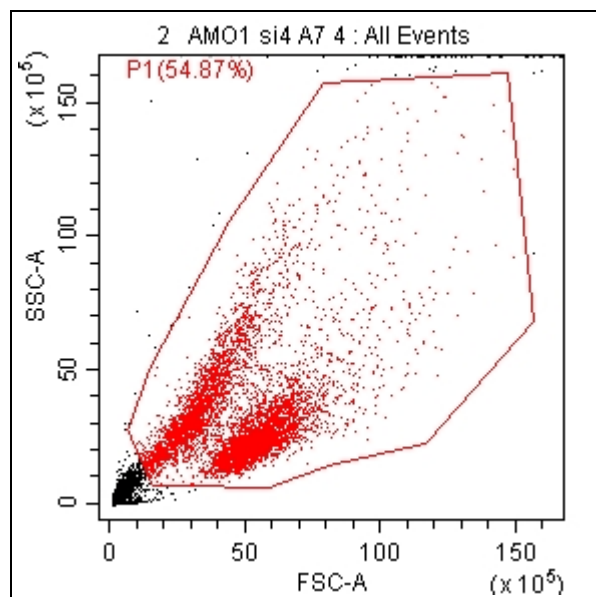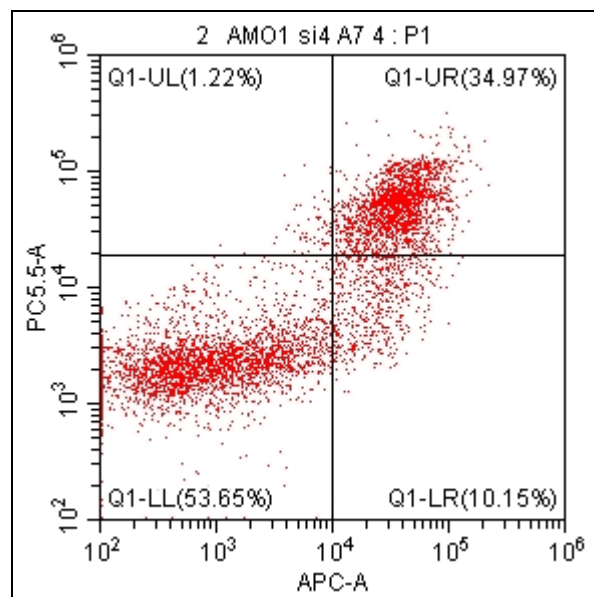

Supplement: Supplementary file 4 [file DataSheet2.ZIP › cell apoptosis/AMO1/2 AMO1 si4 A7 4.pdf]

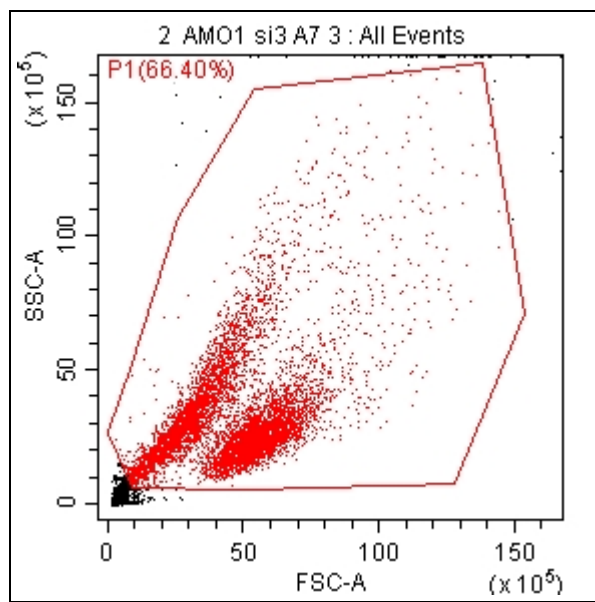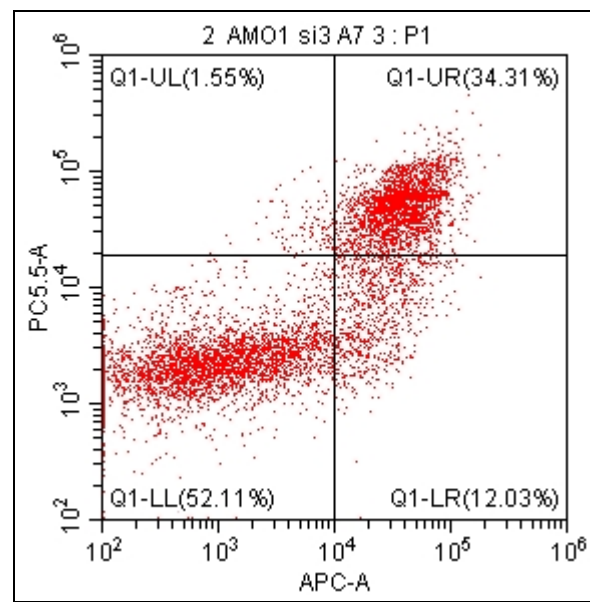

Supplement: Supplementary file 4 [file DataSheet2.ZIP › cell apoptosis/AMO1/2 AMO1 si3 A7 3.pdf]

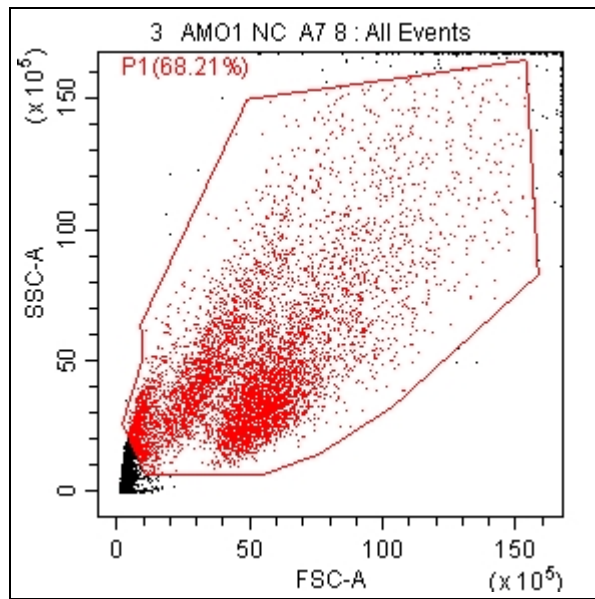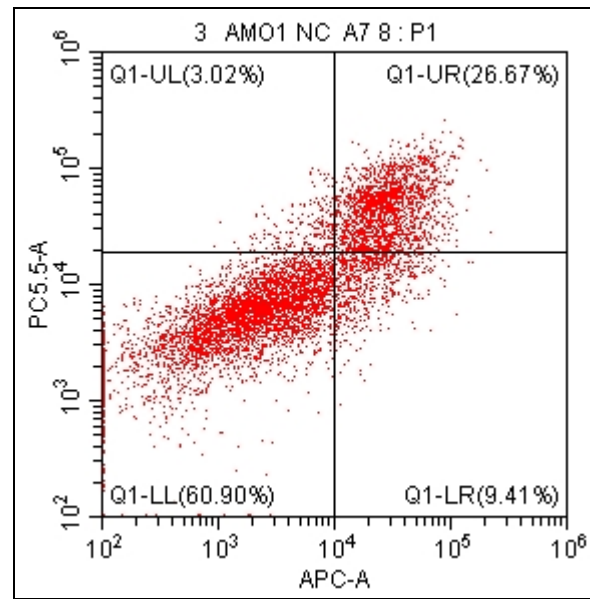

Supplement: Supplementary file 4 [file DataSheet2.ZIP › cell apoptosis/AMO1/3 AMO1 NC A7 8.pdf]

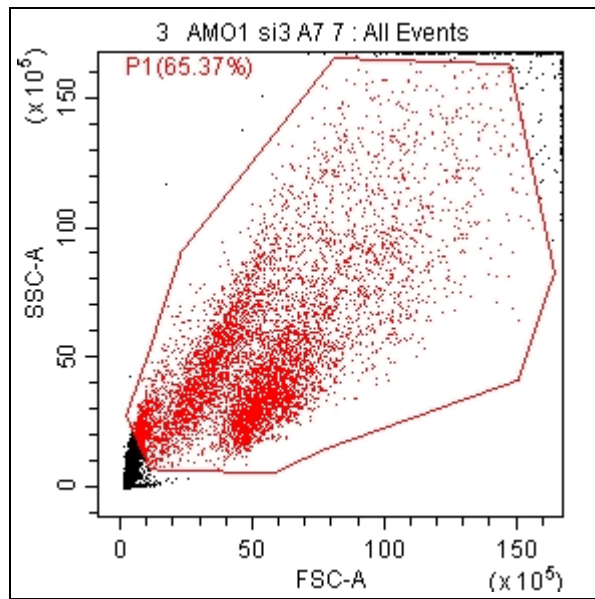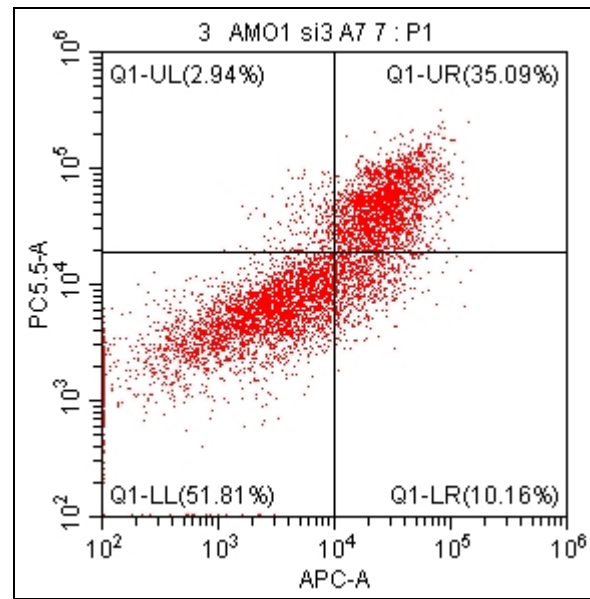

Supplement: Supplementary file 4 [file DataSheet2.ZIP › cell apoptosis/AMO1/3 AMO1 si3 A7 7.pdf]

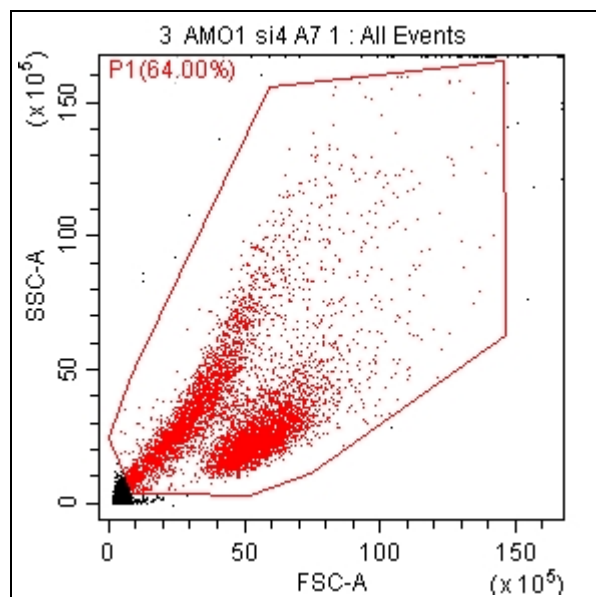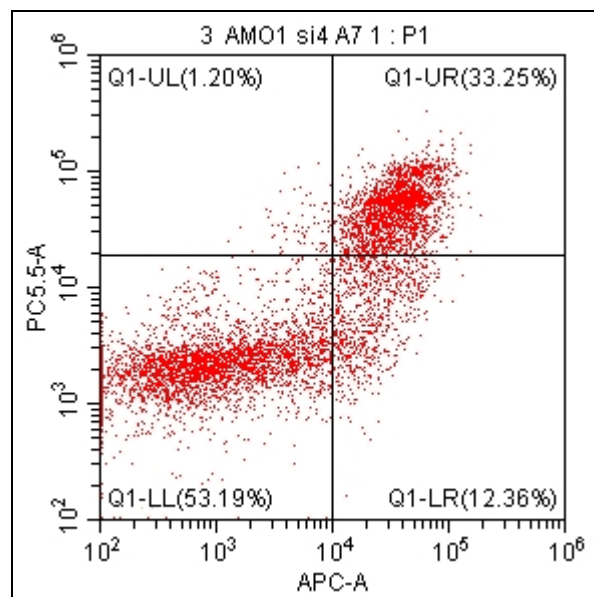

Supplement: Supplementary file 4 [file DataSheet2.ZIP › cell apoptosis/AMO1/3 AMO1 si4 A7 1.pdf]

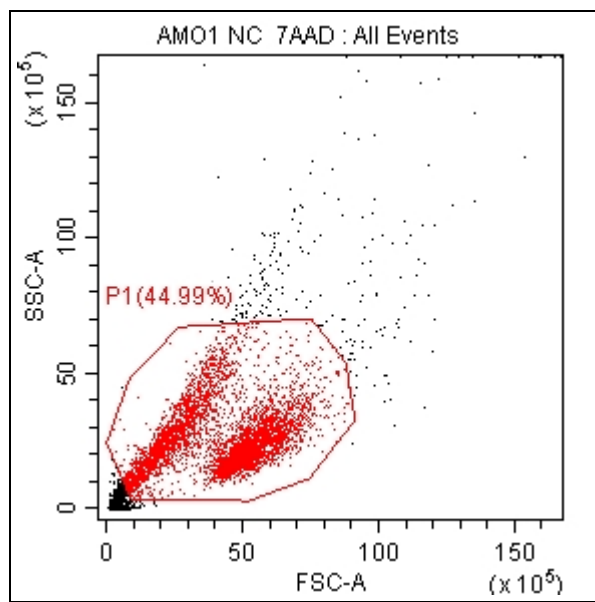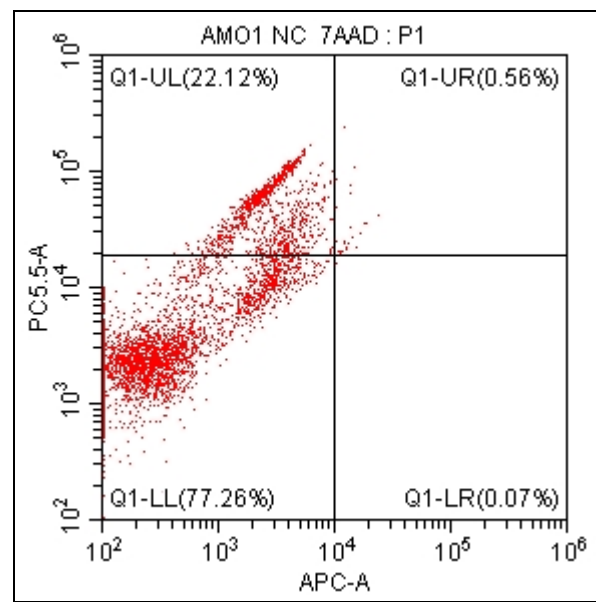

Supplement: Supplementary file 4 [file DataSheet2.ZIP › cell apoptosis/AMO1/AMO1 NC 7AAD.pdf]

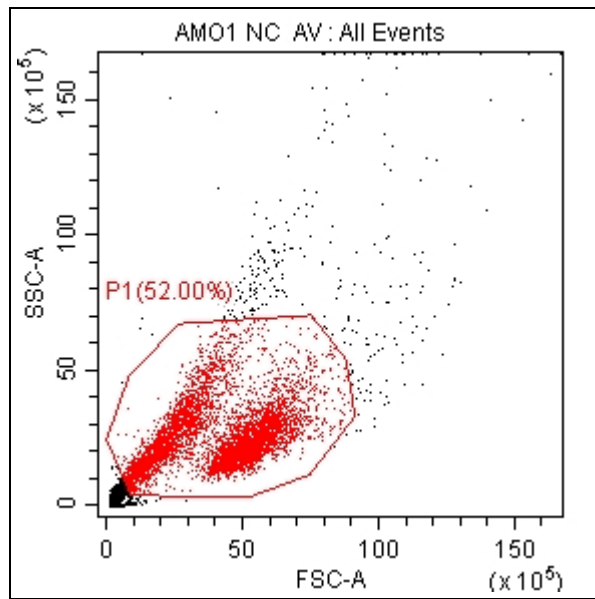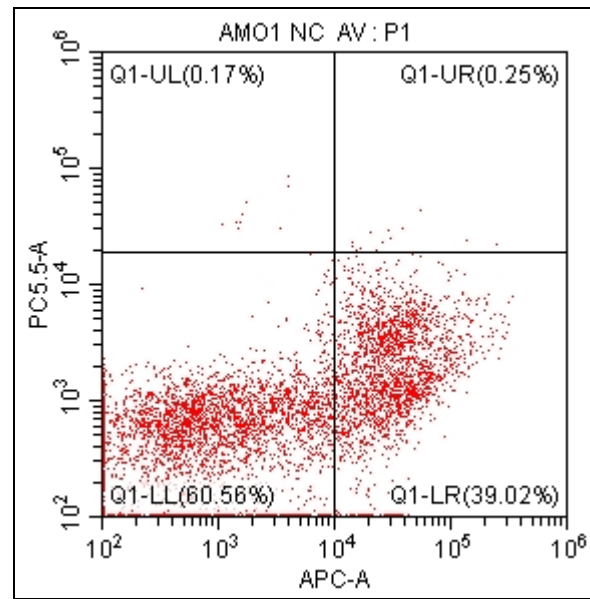

Supplement: Supplementary file 4 [file DataSheet2.ZIP › cell apoptosis/AMO1/AMO1 NC AV.pdf]

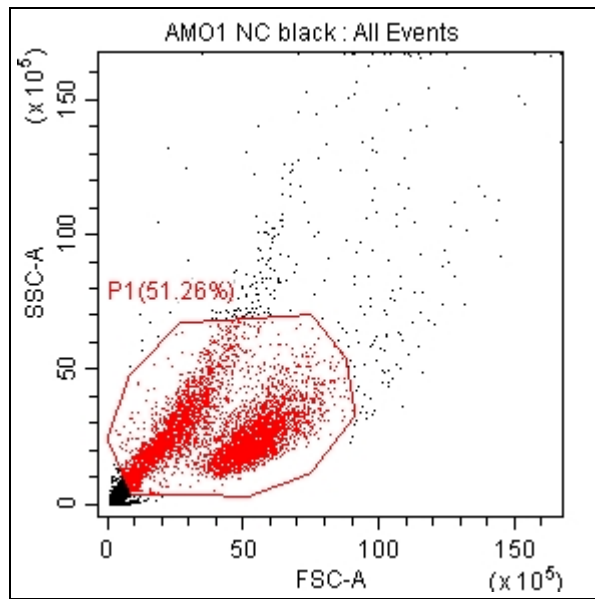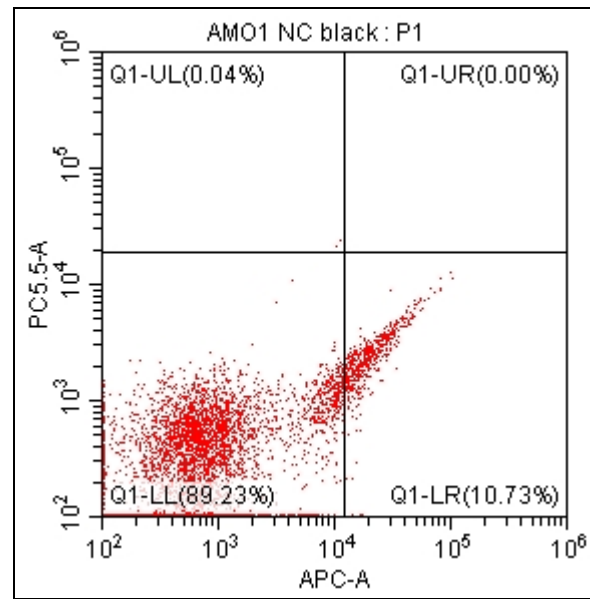

Supplement: Supplementary file 4 [file DataSheet2.ZIP › cell apoptosis/AMO1/AMO1 NC blank.pdf]
